# Dynamics of Metal–Metal Bond Dissociation in Pd–Pd and Ni–Ni Complexes: Reorganization and Redistribution Reactions of the Metalloradicals

**DOI:** 10.1002/anie.202514965

**Published:** 2025-09-04

**Authors:** Tim Bruckhoff, Vincenz J. Kohler, Felix Braun, Joachim Ballmann, Lutz H. Gade

**Affiliations:** ^1^ Anorganisch‐Chemisches Institut Ruprecht‐Karls‐Universität Heidelberg Im Neuenheimer Feld 276 69120 Heidelberg Germany

**Keywords:** Metalloradicals, Metal–metal bonds, Nickel, Palladium, T‐shaped complexes

## Abstract

Dinuclear M(I)–M(I) complexes (M = Ni, Pd) may serve as stable reservoir forms for highly reactive mononuclear metalloradicals, which are of interest as potential catalytic species. However, their dissociation dynamics as well as the factors governing monomer stabilization remain incompletely understood. This study investigates the influence of steric bulk and residual ligand flexibility within a PNP pincer framework on the homolytic dissociation behavior of unsupported Ni(I)–Ni(I) and Pd(I)–Pd(I) dimers. Utilizing an *^i^* ^Pr^PNP pincer ligand, direct evidence of accessible reversible homolytic cleavage in such Pd(I) species has been obtained by NMR and EPR spectroscopy. A kinetic and thermodynamic analysis, coupled with DFT modeling, allowed detailed examination of the dissociation process, including geometric influences before and after cleavage on the dissociation barrier. For nickel, a T‐shaped [(*^i^* ^Pr^PNP)Ni^I^] monomer was isolated and fully characterized. Reduced steric inter‐ligand repulsion for an ethyl‐substituted PNP pincer yielded stable unsupported dimers for both metals. The two homodinuclear complexes underwent thermal and photochemical redistributions giving the first unbridged heterobimetallic Ni(I)–Pd(I) dimer. Thus, ligand sterics and flexibility critically tune the M–M bond strength and dissociation kinetics. Conformational adaptability lowers activation barriers for radical dissociation, while geometric relaxation stabilizes monomers, enabling controlled access to open‐shell species relevant in catalytic reactions.

## Graphical abstract

PNP‐stabilized dinuclear [Pd^I^]_2_ and [Ni^I^]_2_ complexes with unsupported metal–metal bonds may serve as reservoir forms for homolytically generated metalloradical species, and a quantitative investigation of the metal–metal bond dissociation dynamics has been achieved. Metalloradical fragment redistribution led to an unsupported heterodinuclear Ni–Pd dimer.

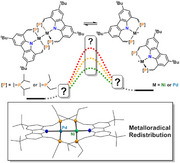

## Introduction

The role of nickel(I) and palladium(I) metalloradicals as reactive species in known as well as new chemical transformations has recently received increasing attention.^[^ ^1^, ^2^, ^3^, ^4^, ^5^, ^6^, ^7^, ^8^, ^9^, ^10^, ^11^, ^12^, ^13^, ^14^, ^15^, ^16^, ^17^, ^18^ ^]^ The isolation of such transition metal complexes with pronounced metal‐centered radical‐type reactivity enables the investigation of novel reactive patterns and their underlying mechanisms. While mononuclear nickel(I) compounds have been extensively studied,^[^ ^1^, ^4^ ^]^ the isolation of palladium(I) metalloradicals has proved more challenging due to their instability and resulting high reactivity. Consequently, the isolation and characterization of several mononuclear palladium(I) compounds has been reported only recently.^[^ ^14^, ^15^, ^16^, ^17^, ^18^, ^19^, ^20^, ^21^, ^22^ ^]^

On the other hand, dinuclear Pd(I) complexes featuring a metal–metal bond have been employed as reservoirs for the in situ generation of highly reactive Pd(0) monomers via redox disproportionation.^[^ ^23^, ^24^ ^]^ It is in this form that palladium(I) species have played an increasingly prominent role as precatalysts for a wide variety of organic transformations.^[^ ^24^ ^]^ Generally, a bridged metal–metal bond stabilizes the attractive interaction between the monometallic units significantly more effectively than an unsupported bond.^[^ ^24^, ^25^, ^26^ ^]^ The latter was first reported for palladium by Otsuka et al. in 1971 and structurally elucidated for an analogous complex by Balch et al. in 1983.^[^ ^27^, ^28^ ^]^ Moreover, for bridged [Pd^I^]_2_ dimers, direct dinuclear mechanisms in their interaction with substrates are favored over the dissociation into Pd(I) monomers, which was found to be energetically unlikely.^[^ ^24^, ^25^, ^29^, ^30^, ^31^, ^32^ ^]^ The accessibility of “palladoradicals” in solution thus would be expected to depend critically on the thermodynamic and kinetic stability of the dimeric state.^[^ ^33^, ^34^, ^35^, ^36^ ^]^ In fact, the only example for the reversible generation of a monomeric palladoradical from a dinuclear [Pd^I^]_2_ precursor has been reported by the Ozerov group who interpreted the mononuclear fragment as a Pd(0) complex coordinated by an oxidized ligand radical.^[^ ^26^ ^]^

Several factors render the dissociation of metal–metal bonds in polynuclear complexes favorable apart from intramolecular repulsion of sterically bulky complex fragments. Flexibility of the ancillary ligand not only offers steric relief—and thus stabilization—in the dinuclear form, it may also provide access to conformations from which dissociation can proceed via reduced activation barriers. In addition, metal–metal bond cleavage may be favored by structural reorganization and relaxation of the mononuclear species following the dissociation step.^[^ ^37^ ^]^

Our recent isolation of the PNP‐stabilized T‐shaped mononuclear [(*^t^* ^Bu^PNP)Pd^I^] metalloradical^[^ ^17^ ^]^ has prompted us to study the effect of reduced steric bulk on the stability of the mononuclear metalloradicals and dissociation dynamics of their dimeric reservoir forms [Pd^I^]_2_. In fact, the properties of the system have allowed us to monitor the dissociation dynamics of the metal–metal bonded form and thus investigate this type of process quantitatively for the first time. In this way, kinetic and thermodynamic parameters for such homolytic metal–metal bond dissociations were obtained. The analysis of the processes includes the effects of ligand dynamics on both the dissociation process itself and the stabilization of the resulting monomer units. Finally, the extension to their nickel analogues has provided additional insight and allowed crossover experiments between otherwise isostructural complexes.

## Results and Discussion

### Reversible Pd–Pd Bond Homolysis in [(*^i^* ^Pr^PNP)Pd]_2_

**Figure 1:**
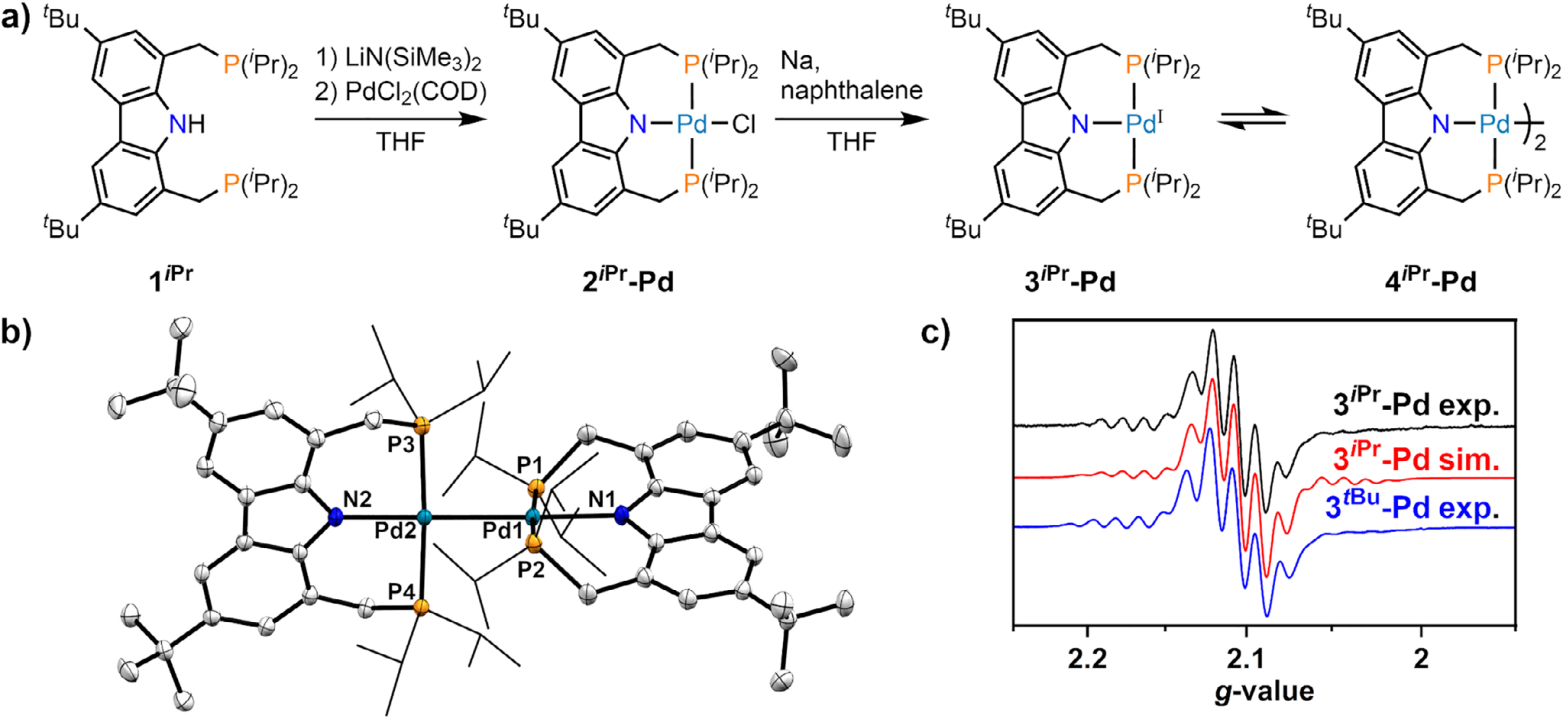
a) Synthesis of the homodinuclear palladium dimer [(*^i^* ^Pr^PNP)Pd]_2_ (**4^iPr^‐Pd**) starting with the protio ligand **1^iPr^**. b) Molecular structure of **4^iPr^‐Pd** in the crystal with thermal ellipsoids shown for 50% probability. Hydrogen atoms, two disordered co‐crystallized THF molecules and one disordered *^t^* Bu‐group are omitted for clarity. Selected bond lengths and angles in [Å] and [°] for Pd1–Pd2 2.6600(2); N1–Pd1 2.1878(19); N2–Pd2 2.1842(18); P1–Pd1 2.3365(6); P2–Pd1 2.3146(6); P3–Pd2 2.3192(6); P4–Pd2 2.3274(6); N1–Pd1–Pd2 177.50(6); P1–Pd1–P2 176.89(2); N2–Pd2–Pd1 178.29(5); P3–Pd2–P4 174.97(2). c) X‐band EPR spectrum of **3^iPr^‐Pd** in C_6_D_6_ at room temperature (black line) with the simulated EPR parameters (red line) and the previously reported spectrum of [(*^t^* ^Bu^PNP)Pd^I^]^[^ ^17^ ^]^ (**3^tBu^‐Pd**) for comparison (blue line).

Following the previously reported method,^[^ ^17^ ^]^ the chlorido precursor [(*^i^* ^Pr^PNP)PdCl] (**2^iPr^‐Pd**) was synthesized by deprotonating the protio ligand 3,6‐di‐*iso*‐propyl‐1,8‐bis((di‐*tert*‐butylphosphanyl)methyl)‐carbazole (*^i^* ^Pr^PNP, **1^iPr^**) with lithium bis(trimethylsilyl)amide (LiHMDS) and subsequent salt metathesis with PdCl_2_(COD). Reduction with 1.2 equiv of sodium and 20 mol% naphthalene yielded a diamagnetic product **4^iPr^‐Pd**, for which single crystal X‐ray diffraction confirmed its dimeric structure with an unsupported Pd–Pd bond (Figure 1a,b).^[^ ^38^ ^]^ The Pd–Pd bond length of 2.6600(2) Å is in the range of previously reported values for the [(PNP)Pd^I^] dimers described by Ozerov et al. (2.5758(4)–2.7167(2) Å).^[^ ^26^, ^39^ ^]^ Further structural analysis revealed nearly square‐planar coordination geometries at both palladium centers ($\tau_{4}^{\mathrm{Pd}1}$ = 0.04, $\tau_{4}^{\mathrm{Pd}2}$ = 0.05).^[^ ^40^ ^]^

At ambient temperature, a solution of the palladium dimer **4^iPr^‐Pd** was found to give rise to an EPR signal (X‐band, Figure 1c), indicating reversible homolytic cleavage of the Pd–Pd bond and formation of the Pd^I^ metalloradical. The spectral features closely resembled those previously recorded for the mononuclear [(*^t^* ^Bu^PNP)Pd^I^] complex.^[^ ^17^ ^]^ Simulation of the EPR spectrum of **3^iPr^‐Pd** gave an isotropic *g*‐value of *g*_iso_ = 2.104 along with hyperfine coupling (HFC) interactions with two phosphorus atoms (*I* = ½, *A*_iso_ = 54.9 MHz), one nitrogen atom (*I* = 1, *A*_iso_ = 54.9 MHz), and the ^105^Pd nucleus (*I* = 5/2, 22.34%, *A*_iso_ = 119.8 MHz), consistent with data obtained for [(*^t^* ^Bu^PNP)Pd^I^]. Notably, the frozen solution at 6 K was found to be EPR silent, mirroring the greater thermodynamic stability of the diamagnetic dimer **4^iPr^‐Pd** compared to the monomer **3^iPr^‐Pd**.

### NMR Spectroscopy of the Palladium‐Radical 3^iPr^‐Pd

**Figure 2:**
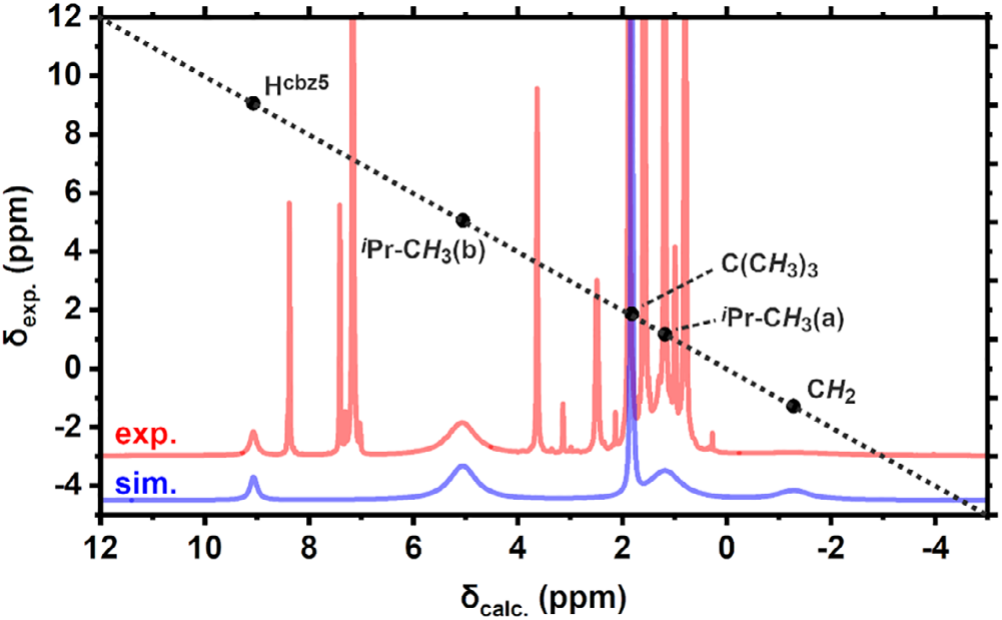
Correlation between the experimental (353 K, 600 MHz, C_6_D_6_, red line) and fitted (simulation, blue line) proton resonances of **3^iPr^‐Pd**. The black dotted line represents a perfect correlation. Fitted signal errors were determined to be <0.1 ppm. Additional (sharp) signals in the experimental spectrum correspond to the diamagnetic dimer **4^iPr^‐Pd** and solvent.

Remarkably, direct detection of the monomer **3^iPr^‐Pd** alongside dimer **4^iPr^‐Pd** in solution was possible by ^1^H NMR spectroscopy at temperatures above 0 °C. The complete identification and assignment of the paramagnetic proton resonances in **3^iPr^‐Pd** were achieved via simulation of a ^1^H NMR spectrum at 80 °C (see Figure 2) based on the relation:^[^ ^41^, ^42^, ^43^ ^]^

(1) $$\delta_{\mathrm{calc}}=\delta_{\mathrm{dia}}+\delta_{\mathrm{HF}}=\delta_{\mathrm{dia}}+\frac{1}{3}\mathrm{Tr}\left(A\chi \right)$$

The hyperfine tensors (**A**) were computed purely by DFT (B3LYP‐GD3(BJ)^[^ ^44^, ^45^, ^46^, ^47^, ^48^, ^49^ ^]^/SARC‐ZORA‐TZVP(Pd),^[^ ^50^ ^]^ ZORA‐Def2‐TZVP(‐f)^[^ ^51^ ^]^), whereas the CPCM(benzene)^[^ ^52^, ^53^ ^]^/TPSSh‐GD3(BJ)^[^ ^46^, ^47^, ^48^, ^49^, ^54^, ^55^ ^]^/Def2‐TZVP(Pd),^[^ ^51^ ^]^ 6‐311G(d,p)^[^ ^56^, ^57^ ^]^ level of theory, combined with an empirical correction, was used to model diamagnetic shifts (δ_dia_). The susceptibility tensor (**χ**) was iteratively optimized to minimize the root mean square deviations (RMSD) between calculated and experimental proton signals. Line broadening effects were quantified using estimated longitudinal electron relaxation times and rotational correlation times, which determined the relaxation rates (for more details on the NMR simulations, see Supporting Information: 10.1). On the other hand, severe line broadening precluded interpretable signals in the ^13^C NMR spectrum of **3^iPr^‐Pd**.

### Dynamics and Thermodynamics of the Metal–Metal Bond Dissociation

**Figure 3:**
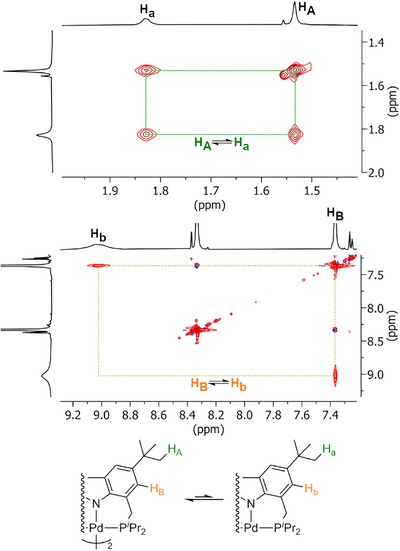
Direct observation of the Pd–Pd bond homolysis through exchange signals in 2D EXSY NMR (top: alkyl region, bottom: aromatic region, *t*_mix_ = 0.1 s).

The reversible bond homolysis could also be observed via 2D NMR exchange spectroscopy (2D EXSY NMR, Figure 3). Such an interchange between a metalloradical species and its dimer by NMR spectroscopy provides the second such example, the first having been reported recently for a Co–Co dimer.^[^ ^36^ ^]^ This allowed a convenient experimental determination of the Pd–Pd bond dissociation enthalpy (BDE) as well as bond dissociation free energy (BDFE) by monitoring concentration ratios of **3^iPr^‐Pd** and **4^iPr^‐Pd** at varying temperatures. At 80 °C, four out of seven expected monomer proton signals were found, with two exhibiting EXSY cross peaks to the dimer and thus enabling unambiguous assignment. Although the *tert*‐butyl groups on the monomer's carbazole moiety already appeared sharp at lower temperatures with cross correlation in EXSY, their partial overlap with other resonances made integration unreliable. Consequently, quantitative ratios were derived from less intense, broadened but well isolated, exchange‐coupled carbazole protons instead (cf. Supporting Information: 8.3). An experimental BDE of 24.06 ± 0.42 kcal mol^−1^, an entropy (Δ*S*°) of 62.20 ± 1.32 cal mol^−1^ K^−1^ and BDFE (298 K) of 5.53 ± 0.58 kcal mol^−1^ have been obtained. This is consistent with the DFT‐modeled data (CPCM(toluene)/r^2^SCAN‐3c^[^ ^58^, ^59^, ^60^ ^]^/Def2‐mTZVP,^[^ ^51^, ^58^, ^61^ ^]^ see Supporting Information: 10.6), namely a BDE of 27.5 kcal mol^−1^, an entropy of 70.0 kcal mol^−1^, and BDFE of 6.7 kcal mol^−1^, and in line with values found for other metal–metal bond homolysis.^[^ ^62^, ^63^, ^64^, ^65^, ^66^, ^67^, ^68^ ^]^

Frequently, these thermodynamic parameters are interpreted as direct indicators of the energy needed to break a bond or directly reflect its inherent strength. However, this assumption holds true only if the thermodynamic ground states involved in bond cleavage transition directly into one another without intermediates or transition states. As BDE and BDFE are state functions, they solely describe relative energies between thermodynamic minima and therefore neglect potential kinetic barriers or conformational rearrangements during dissociation.

**Figure 4:**
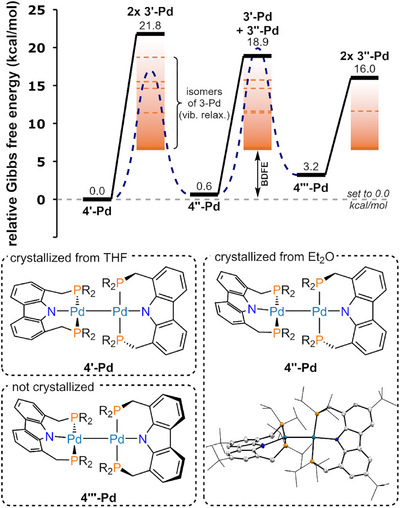
Relative Gibbs free energy profile (Δ*G*) of the direct [(^R^PNP)Pd^I^] (R = *i*Pr) fragment dissociation starting at three different conformers (**4′‐Pd**, **4′′‐Pd**, and **4′′′‐Pd**) for the palladium dimer (**4^iPr^‐Pd**). All compounds were computed as truncated structures in which the carbazole bound *^t^* Bu‐groups were replaced by H‐atoms. The metalloradical fragments are denoted as **3′‐Pd** for the twisted conformation and as **3′′‐Pd** for the seat conformation. The conformers of the dinuclear complex can transition into each other by ligand reorganization (blue line). Vibrational relaxation (orange) in each monomer results in the small BDFE (cf. Supporting Information: 10.7 and 10.8). The orientation of the *iso*‐propyl substituents in the solid‐state molecular structure of **4′′‐Pd** differs compared to a more ideal positioning found in silico (ΔG = 0.6 kcal mol^−1^), leading to a slightly higher relative energy (Δ*G* = 3.7 kcal mol^−1^) for the molecular structure in the crystal.

In fact, a computational study of the dissociation mechanism of **4^iPr^‐Pd** revealed that direct monomerization from its most stable dimeric ground state structure incurs a dissociation energy significantly higher than the experimentally determined BDFE (Figure 4). Although initial conformational preorganization can lower this barrier by enabling a more favorable dissociation pathway, the isomerization barriers were computed to be greater than the experimental BDFE. However, the high number of local energy minima due to the different orientations of the *iso*‐propyl substituents complicated the determination of the lowest energy transition states for each isomerization step and therefore only allowed for an estimate of an upper boundary for the activation barrier.

Upon changing the solvent for crystallization, a second conformer (analogous to **4′′‐Pd**) was observed with one Pd center exhibiting increased tetrahedral distortion ($\tau_{4}^{\mathrm{Pd}1}$ = 0.08, $\tau_{4}^{\mathrm{Pd}2}$ = 0.25, Figure 4) and an elongated Pd–Pd distance of 2.6752(4) Å. This form was found to be disfavored by 3.7 kcal mol^−1^ in solution in silico relative to the first conformer.

The conformational equilibration of **4^iPr^‐Pd** was reflected in the observation of only one ^31^P NMR resonance (*δ* = 9.9 ppm) at 295 K reflecting an effective D_2d_ symmetry on the NMR time scale in solution. Thus, the flexibility of the methylene bridge connecting the carbazole core and phosphine arms enables thermal interconversion between the conformers at room temperature with a relatively low‐lying energy barrier. Variable‐temperature ^1^H NMR established an isomerization barrier of 13.1 kcal mol^−1^, which is consistent with a DFT‐calculated (upper boundary) value of 17.0 kcal mol^−1^ (vide supra; see Supporting Information: 8.1 and 10.7 for details).

### Structural and Electronic Properties of T‐Shaped [(*^i^* ^Pr^PNP)Ni^I^]

**Figure 5:**
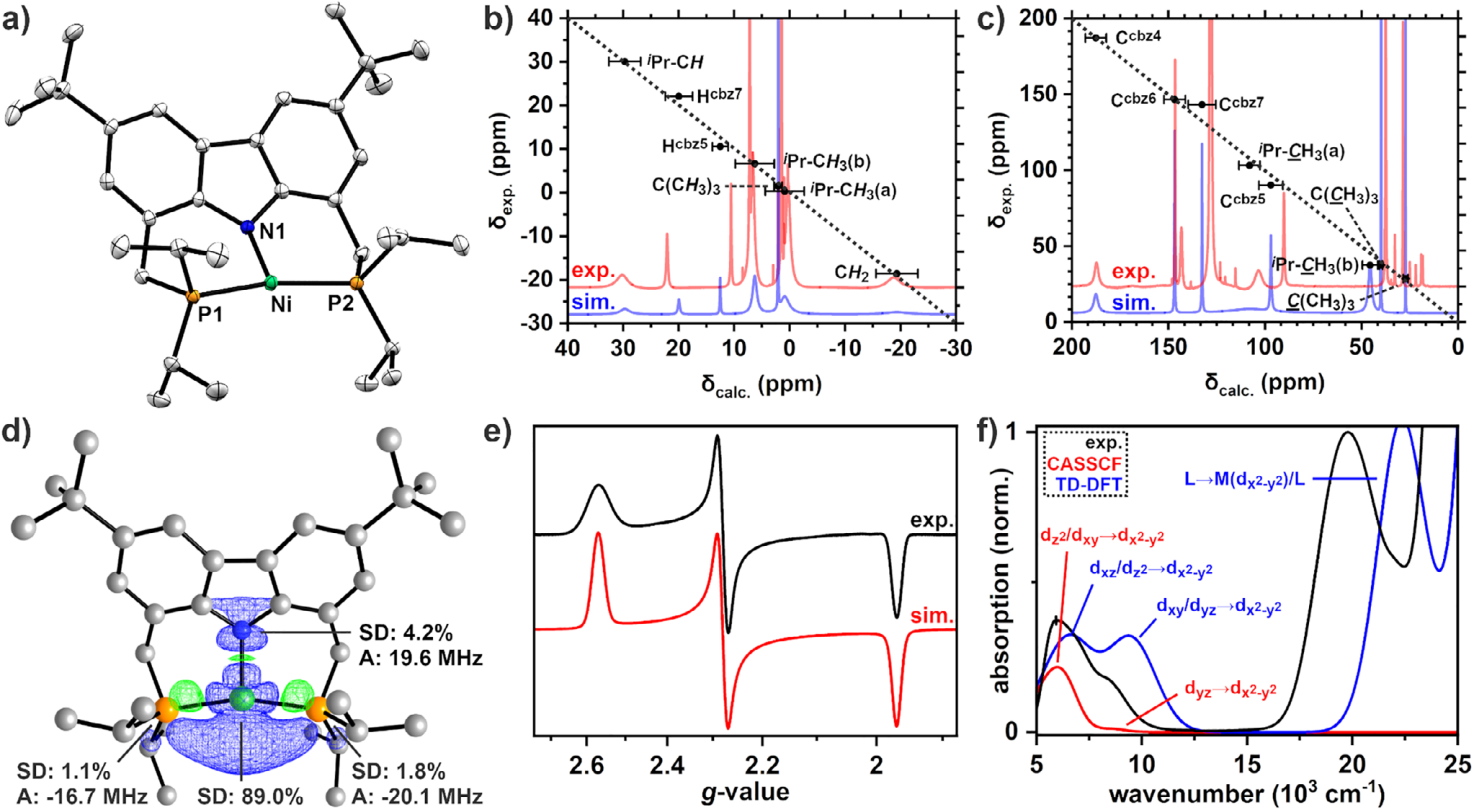
a) Molecular structure of **3^iPr^‐Ni** in the solid‐state with thermal ellipsoids shown at 50% probability level. Hydrogen atoms are omitted for clarity. Selected bond lengths and angles in [Å] and [°]: N1–Ni 1.9472(9); P1–Ni 2.2075(3), P2–Ni 2.1911(3); N1–Ni–P1 97.92(3); N1–Ni–P2 97.34(3); and P1–Ni–P2 164.477(12). b and c) Correlation between the experimental (red line) and fitted (simulation, blue line) (b) ^1^H NMR (C_6_D_6_, 600 MHz, 295 K) and (c) ^13^C NMR (C_6_D_6_, 151 MHz, 295 K) resonances of **3^iPr^‐Ni**. Fitted signal errors are depicted as error bars; the black dotted line represents a perfect correlation. d) Spin density (contour value = 0.001), Mulliken spin densities, and EPR hyperfine coupling constants (level of theory: B3PW91‐GD3(BJ)^[^ ^44^, ^46^, ^49^, ^71^ ^]^/ZORA‐def2‐TZVP(‐f)). e) X‐band EPR spectrum of **3^iPr^‐Ni** in 1‐methyl‐THF at 6 K (black line) with the simulated EPR parameters (red line). f) Normalized experimental UV/Vis‐NIR absorption spectrum of **3^iPr^‐Ni** in pentane (black line) illustrated in relation to TD‐DFT (blue line) and ab initio (SC‐NEVPT2^[^ ^72^, ^73^, ^74^ ^]^ corrected CASSCF, red line) computed absorption spectra. Absorption intensities (ε) of computed spectra were referenced against the extinction coefficient for the experimental absorption at 19 685 cm^−1^.

Replacing palladium with nickel while maintaining the *^i^* ^Pr^PNP ligand (**1*^i^* ^Pr^**) resulted in a different structural and electronic outcome for the corresponding Ni(I) complex. This difference arises from a smaller covalent radius of nickel and thus increased steric shielding of the metal center. The nickel congener **3^iPr^‐Ni** was prepared analogously to the palladium derivative via reaction with NiCl_2_(dme), giving the [(*^i^* ^Pr^PNP)NiCl] precursor as a green solid in 91% isolated yield. Subsequent reduction with 1.05 equiv sodium lead alloy afforded a paramagnetic complex as a pale red solid in 89% yield. The three‐coordinate and nearly T‐shaped structure of this compound was established by X‐ray diffraction (Figure 5a).

The distances between the nickel center and the PNP‐ligand donor atoms (Ni–P1 2.2075(3) Å, Ni–P2 2.1911(3) Å, and Ni–N1 1.9472(9) Å) are significantly shorter than those observed in mononuclear [(*^t^* ^Bu^PNP)Pd^I^] (Pd1–P1 2.2980(14) Å, Pd1–P2 2.3007(14) Å, and Pd1–N1 2.145(4) Å)^[^ ^17^ ^]^ or the chair‐conformer [(*^i^* ^Pr^PNP)Pd] moiety in **4^iPr^‐Pd** (Pd2–P3 2.3202(12) Å, Pd2–P4 2.33303(13) Å, and Pd2–N2 2.226(4) Å). This reduces the space between the *iso*‐propyl substituents, suppressing dimerization by increased shielding of the nickel center.

The T‐shaped nickel(I) complex **3^iPr^‐Ni** was further characterized by paramagnetic NMR (^1^H, ^13^C), EPR, and UV/Vis‐NIR spectroscopy. All proton and the majority of carbon resonances were detected and resolved in the NMR spectra at 295 K (Figure 5b,c). Assignments relied again on spectral simulations obtained by optimizing the susceptibility tensor and were complemented by analysis of deconvoluted signal integrals (for details see Supporting Information: 10.1). In this way, good agreement between theoretical and experimental shifts of a T‐shaped complex in solution has been achieved.

The nickel(I) complex remained monomeric upon reducing the temperature, leading to an EPR signal in a frozen solution with three broadened features, resulting from large g‐anisotropy (Figure 5e). Simulation of this signal based on a *S* = ½ configuration gave anisotropic *g*‐values of *g*_x_ = 2.57, *g*_y_ = 2.28 and *g*_z_ = 1.97. At room temperature, the broadened signal was fitted with an isotropic value of *g*_iso_ = 2.26, close to the mean value of the experimental anisotropic *g*‐values (cf. Figure S129 for isotropic spectrum). Hyperfine splittings were unresolved at both temperatures, precluding determination of HFC constants. Mulliken population analysis confirmed the metalloradical character of **3^iPr^‐Ni**, with 89% of the spin density residing on the nickel center (Figure 5d).

The UV/Vis‐NIR absorption spectrum displays weak absorption bands between 1000 and 2000 nm (5000–10 000 cm^−1^), assigned to d→d transitions according to time‐dependent DFT (TD‐DFT) and ab initio methods (Figure 5f). An experimental absorption maximum was determined at 1673 nm (5977 cm^−1^, ε_1673nm_ = 102 M^−1^ cm^−1^), with a shoulder at 1189 nm (8413 cm^−1^). Deconvolution of the band revealed three absorption maxima at 1189, 1464, and 1732 nm (8413, 6830, and 5773 cm^−1^) while TD‐DFT predicted four d→d transitions to be between 5000 and 10 000 cm^−1^ (1000–2000 nm). Modeling by CASSCF^[^ ^69^, ^70^ ^]^ suggested one of these transitions to be at lower energy (2076 cm^−1^, 4187 nm, $d_{\mathrm{xz}}\to d_{x^{2}-y^{2}}$) and thus outside the spectral window.

### Reducing the Steric Bulk of the Phosphine Groups to Stabilize M–M‐Bonding: Et_2_P‐Substitution in [(^Et^PNP)M]_2_ (M = Ni, Pd)

Previous studies of PNP‐stabilized monovalent mononuclear nickel complexes employed bulky *tert*‐butyl or *iso*‐propyl‐substituted phosphines to prevent dimerization.^[^ ^75^, ^76^, ^77^, ^78^, ^79^ ^]^ However, increased flexibility or decrease of the size of the phosphine substituents yielded bridged Ni(I) dimers via “overreach” of a phosphine unit to a second Ni center giving rise to either closed‐shell singlet^[^ ^80^ ^]^ or open‐shell triplet configurations.^[^ ^81^ ^]^ In contrast, metal(I) dimers bearing an unsupported metal–metal bond are rare for nickel complexes^[^ ^82^, ^83^, ^84^, ^85^, ^86^, ^87^ ^]^ but could act as reservoirs for mononuclear metalloradical species through homolytic cleavage of the M–M single bond akin to the dynamics observed for **4^iPr^‐Pd** (vide supra). In this way, sterically less hindered T‐shaped complexes might be accessible via reversible dissociation of such dimers.

**Figure 6:**
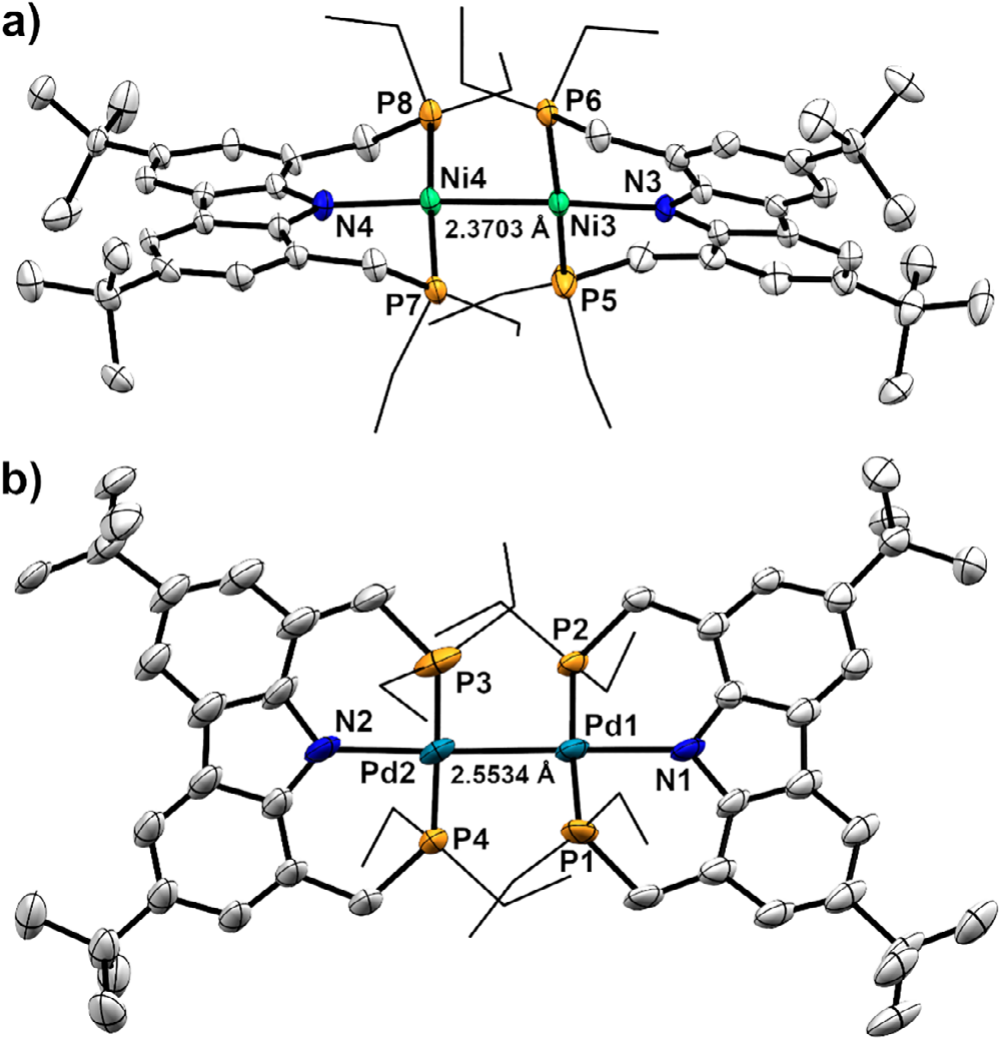
Molecular structures of a) **4^Et^‐Ni** showing one of two crystallographically independent conformers in the cell and b) **4^Et^‐Pd** (thermal ellipsoids at 50% probability). A third conformer for **4^Et^‐Ni** was obtained upon changing the solvent for crystallization (see Figure S149). Selected bond lengths and angles in [Å] and [°] for a) Ni3–Ni4 2.3703(14); N3–Ni3 1.987(5); P5–Ni3 2.194(2); P6–Ni3 2.170(2); N4–Ni4 1.983(5); P7–Ni4 2.181(2); P8–Ni4 2.178(2); N3–Ni3–Ni4 171.62(17); P5–Ni3–P6 171.20(9); N4–Ni4–Ni3 176.78(16); and P7–Ni4–P8 176.92(9) and b) Pd1–Pd2 2.5534(6); N1–Pd1 2.183(5); P1–Pd1 2.2675(18); P2–Pd1 2.2844(16); N2–Pd2 2.167(5); P3–Pd2 2.288(2); P4–Pd2 2.2901(17); N1–Pd1–Pd2 179.59(12); P1–Pd1–P2 175.66(6); N2–Pd2–Pd1 177.53(14); and P3–Pd2–P4 178.45(7).

To explore this approach for both nickel and palladium, the less bulky diethylphosphine‐substituted PNP ligand (**1^Et^**) was synthesized (see Supporting Information) and reacted with the corresponding precursors as described above to give [(^Et^PNP)NiCl] (**2^Et^‐Ni**) and [(^Et^PNP)PdCl] (**2^Et^‐Pd**) in excellent yields (90% and 94%). Reduction of the chlorido complexes **2^Et^‐M** (M = Ni, Pd) with sodium naphthalenide afforded diamagnetic dinuclear species, for which unsupported metal–metal bonds were confirmed by single crystal X‐ray diffraction (Figure 6). The ethyl‐substituted palladium dimer **4^Et^‐Pd** exhibits a Pd–Pd bond length of 2.5534(6) Å, which is thus 0.1066 Å shorter than that in the *iso*‐propyl analog **4^iPr^‐Pd**, reflecting the decreased steric repulsion between the two halves of the molecule. The metal–metal bond length in the (PNP)Pd dimer **4^Et^‐Pd** is in the range of other unbridged Pd–Pd complexes.^[^ ^26^, ^28^, ^39^, ^88^, ^89^, ^90^, ^91^, ^92^, ^93^, ^94^, ^95^, ^96^, ^97^, ^98^, ^99^, ^100^, ^101^, ^102^, ^103^, ^104^, ^105^, ^106^, ^107^ ^]^ Additionally, **4^Et^‐Ni** is the first example of an unbridged Ni(I) dimer stabilized by a PNP ligand, with a Ni–Ni distance between 2.3228(4) Å and 2.3827(14) Å (outer limits of the Ni–Ni bond lengths measured in three crystallographically independent conformers), that is similar to the shortest among other known unbridged nickel dimers.^[^ ^82^, ^83^, ^84^, ^85^, ^86^, ^87^ ^]^

Both the shorter M–M distance in **4^Et^‐Pd** and the stable Ni–Ni bonding in **4^Et^‐Ni** reflect the expected reduced repulsion between the non‐relaxed [(PNP)M] moieties. The greater M–M dissociation energies are also reflected in the absence of EPR signals for solutions at ambient temperature.

NMR spectroscopy revealed resolved ^1^H/^1^H coupling patterns for the palladium complex, both at ambient and elevated temperatures, whereas the nickel dimer showed no such couplings. In principle, this can be due to either fast ligand dynamics in **4^Et^‐Ni** at ambient temperature or thermal population of a low‐lying triplet state in the dimer. A conceivable exchange with an open‐shell mononuclear species and significant (detectable) accumulation of the latter is in contradiction with the absence of an EPR resonance. For **4^Et^‐Ni**, an increased line broadening and slight paramagnetic shifting observed at higher temperatures is thus consistent with the thermal population of an open‐shell (triplet) configuration (vide infra).

DFT calculations further substantiated these findings. For the palladium dimer, **4^Et^‐Pd**, a BDFE of 29.4 kcal mol^−1^ was calculated, while a lower BDFE of 9.9 kcal mol^−1^ was found for the nickel congener reflecting weaker metal–metal bonding. While this suggests sufficient thermal accessibility to enable the formation of the transient open‐shell metalloradical, its equilibrium concentration would be too low to be directly detectable.

Energy decomposition analyses^[^ ^108^ ^]^ (ETS‐NOCV; B3LYP‐TZ2P, D3, ZORA; details in Supporting Information: 10.10) of the dimers **4^iPr^‐Pd** and **4^Et^‐Pd**, fragmented into open‐shell [(PNP)Pd] metalloradicals, were performed to elucidate the origin of higher dimer stability with smaller phosphine substituents, i.e., lower monomeric radical stability. Although steric destabilization decreases slightly (−3.1 kcal mol^−1^) upon substituting *iso*‐propyl with ethyl and orbital interactions (primarily Pd–Pd bonding) are stabilized by −3.7 kcal mol^−1^ due to a shorter M–M distance, the stabilizing total intrinsic interaction energy (Δ*E*_int_) is higher for **4^iPr^‐Pd** (−67.7 versus −65.2 kcal mol^−1^). This arises from a significant loss of dispersion stabilization (about 9.3 kcal mol^−1^) in **4^Et^‐Pd**, contradicting expectations of a stronger interaction between the smaller Et‐substituted fragments. The experimentally observed overall increased stability of **4^Et^‐Pd** stems primarily from less deformation energy required during dimerization. Inter‐fragment repulsion causes more pronounced geometric distortion in the bulkier *^i^* Pr‐substituted dimer (**4^iPr^‐Pd**) relative to its relaxed monomers, leading to significant destabilization of the fragments and thus the dimeric state. Furthermore, the reduced electron‐donating properties of the phosphines upon changing substituents from *iso*‐propyl to ethyl intrinsically destabilize Pd–Pd orbital interactions by only 2.3 kcal mol^−1^. However, this minor effect is overcompensated (−6.0 kcal mol^−1^ stabilization) by geometric relaxation enabling a closer Pd–Pd distance, resulting in the net −3.7 kcal mol^−1^ orbital stabilization noted earlier.

**Figure 7:**
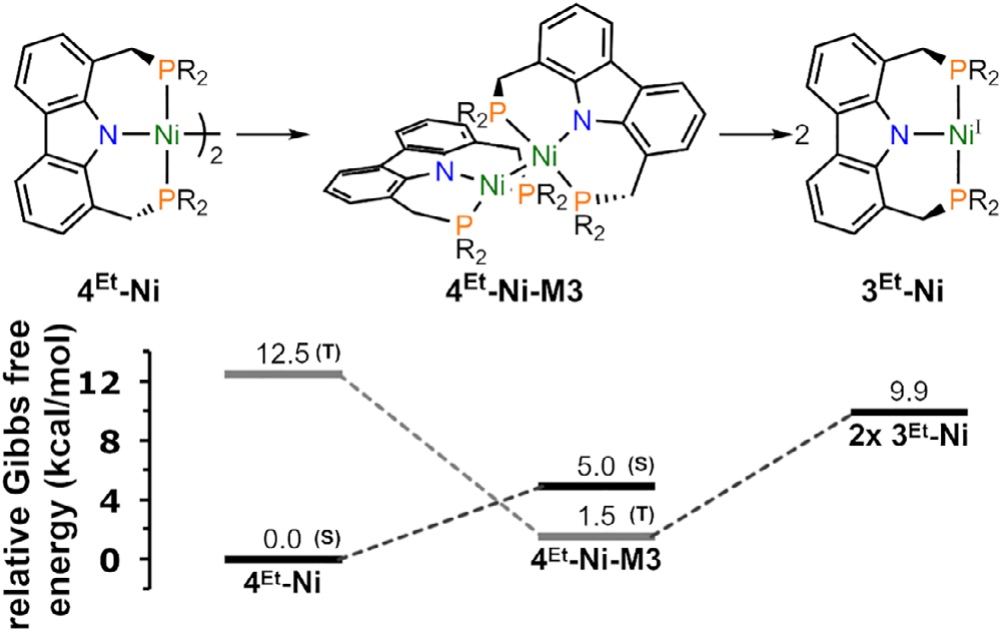
Relative Gibb's free energy pathway toward monomerization of the **4^Et^‐Ni** dimer computed at the r^2^SCAN‐3c/Def2‐mTZVP level of theory, connecting the dinuclear singlet ground state (s) of **4^Et^‐Ni** with a low‐lying triplet state (T) of **4^Et^‐Ni‐M3** and the monomer singlet ground state (**3^Et^‐Ni**). In this figure, the carbazole bound *tert*‐butyl groups were omitted for clarity (R = Et).

Furthermore, computational investigations into the dissociation mechanism have uncovered a dinuclear triplet intermediate (**4^Et^‐Ni‐M3**) that is lower in energy than the proposed T‐shaped monomeric species **3^Et^‐Ni** (Figure 7). Specifically, this triplet structure was calculated to lie 1.5 kcal mol^−1^ above the singlet ground state, suggesting its thermal population under ambient conditions. This additionally explains the observed temperature‐dependent line broadening and shifts of the proton resonances in the NMR spectra of **4^Et^‐Ni**. On the other hand, the corresponding triplet state for **4^Et^‐Pd** was found to be too high in energy (∆*G* = 26.4 kcal mol^−1^) relative to its closed‐shell ground state to be relevant under the conditions described above and consistent with the behavior of this species in solution.

### Photolytic Cleavage and Recombination of [(^Et^PNP)M]_2_ (M = Ni, Pd)

Apart for their thermally induced cleavage, the photolysis of metal–metal bonds may provide access to coordinatively unsaturated metal complex fragments. For symmetric, homodinuclear species these would be expected to possess metalloradical character. Irradiation into excited states with pronounced M–M‐σ* character would initiate such a photolytic fragmentation, giving rise to reactive molecules which either recombine rapidly to regenerate the original reactant or react with substrates present in the reaction mixture. UV–vis spectroscopy revealed absorption maxima at 349 nm (**4^Et^‐Ni**) and 383 nm (**4^Et^‐Pd**) for these dimers, which partially originate from excitations into the M–M‐σ* orbital based on TD‐DFT modeling. Irradiation within this range of wavelengths was thus expected to weaken the M–M‐σ‐bond. However, no paramagnetic species were detected by NMR after irradiating the homodinuclear [(^Et^PNP)M]_2_ (M = Ni or Pd) dimers at 390 nm, nor by in situ EPR during irradiation. This indicated that in the absence of trapping reagents or reactive substrates recombination is fast. Consequently, transient metalloradical species did not accumulate sufficiently at room temperature to be spectroscopically observable under these conditions.

**Figure 8:**
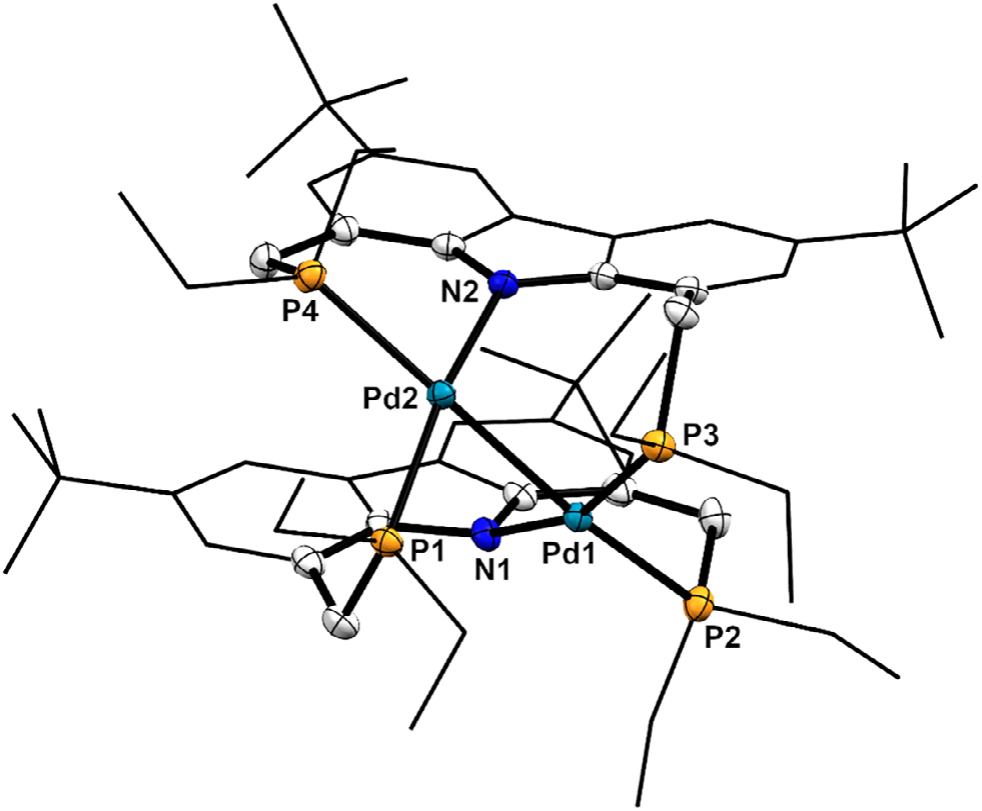
Solid‐state molecular structure of a PNP‐bridged constitutional isomer of **4^Et^‐Pd**. Thermal ellipsoids are depicted with 50% probability and hydrogen atoms and one disordered *^t^* Bu‐group are omitted for clarity. Selected bond lengths and angles in [Å] and [°] for Pd1–Pd2 2.556; N1–Pd1 2.122; P2–Pd1 2.345; P3–Pd1 2.252; N2–Pd2 2.129; P1–Pd2 2.256; P4–Pd2 2.343; N1–Pd1–P3 161.06; P2–Pd1–Pd2 164.56; N2–Pd2–P1 162.91; and P4–Pd2‐Pd1 166.53.

Whereas complex fragments thus could not be detected during irradiation, this does not imply the absence of dynamic processes involving bond cleavage and recombination. This became apparent upon the isolation of single crystals of a new (metastable) form of [(^Et^PNP)Pd]_2_ (Figure 8), which grew from an unsaturated benzene solution of **4^Et^‐Pd** upon irradiation and which rapidly converted back to **4^Et^‐Pd** upon redissolution in benzene. A single crystal X‐ray structure analysis of this metastable form revealed a dinuclear molecular structure in which the PNP ligands bridge the two connected Pd atoms. One phosphine donor of each PNP pincer coordinates to one palladium center and the other to the opposing metal atom (Figure 8). The geometry at both palladium centers is slightly distorted square planar ($\tau_{4}^{\mathrm{Pd}1}$ = 0.24, $\tau_{4}^{\mathrm{Pd}2}$ = 0.22). This structure is related to previously characterized [(PNP)Ni^I^] and [(PNP)Pd^I^] dimers which involved 2,5‐bis(phosphinomethyl)pyrrolato pincers.^[^ ^80^, ^109^ ^]^

### Metalloradical Redistribution Reactivity Between Homodinuclear Complexes

**Figure 9:**
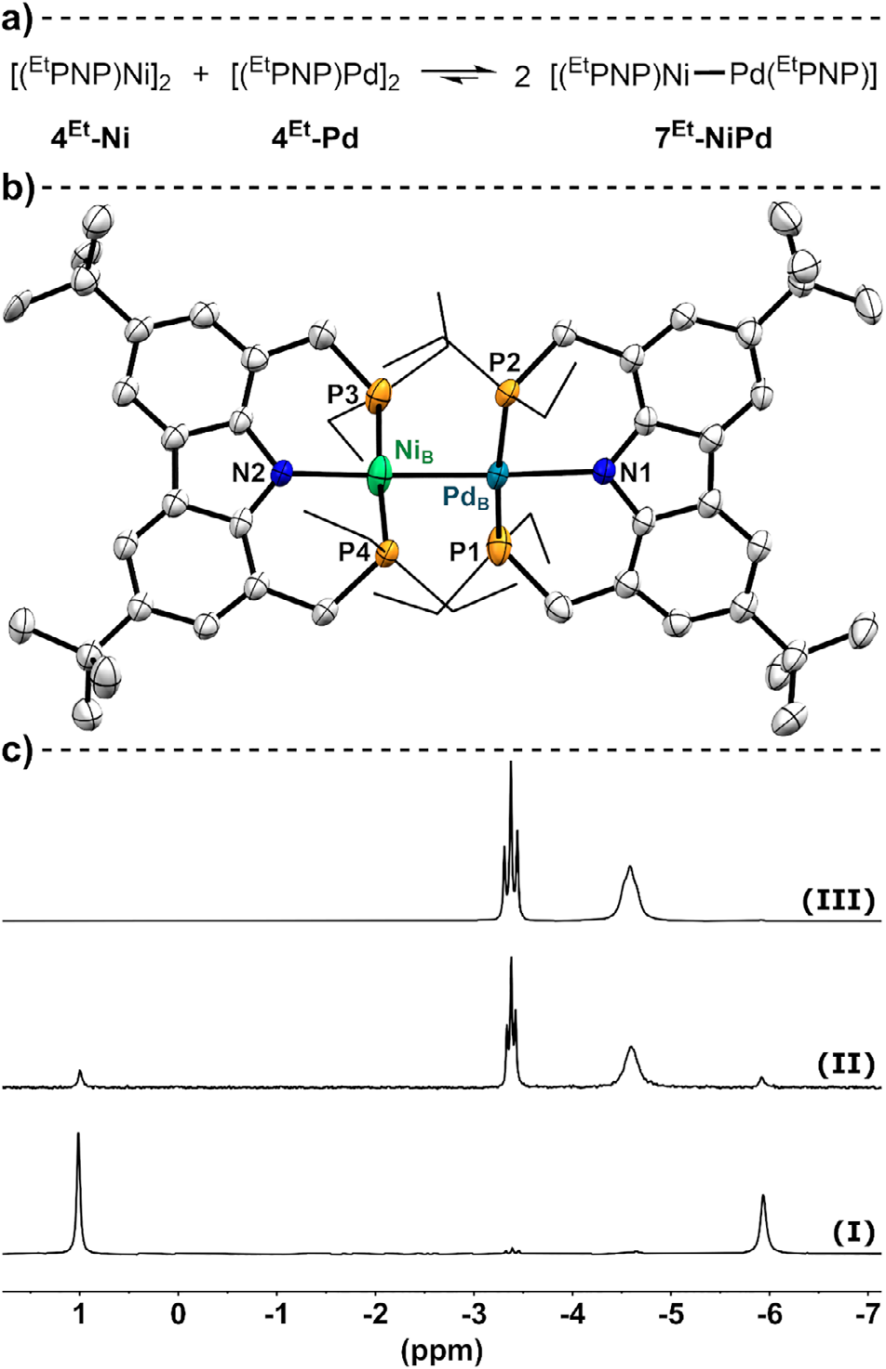
a) Reaction scheme of the reversible thermal metalloradical redistribution between the homodinuclear complexes **4^Et^‐Ni** and **4^Et^‐Pd**. b) Molecular structure of **7^Et^‐NiPd** determined by X‐ray diffraction with thermal ellipsoids at 50% probability level. Disorder of one *tert*‐butyl, three ethyl groups and the metal centers and hydrogen atoms are omitted for clarity. Selected bond length in [Å] for Ni_A_–Pd_A_ 2.486(8); Ni_B_–Pd_B_ 2.442(8). c) ^31^P‐NMR spectra (C_6_D_6_, 243 MHz, 295 K) of the reaction mixture of **4^Et^‐Ni** (1 eq.) and **4^Et^‐Pd** (1 equiv) directly after the addition (I), in thermal equilibrium (II) and directly after irradiation (III).

Reasoning that minimizing steric repulsion between the metal–ligand moieties would prioritize stability governed by intrinsic metal–metal bond strength, the sterically least hindered diethylphosphino‐PNP complexes **4^Et^‐Ni** and **4^Et^‐Pd** were combined (Figure 9a). At ambient temperature, slow conversion to a novel compound **7^Et^‐NiPd** was observed by ^31^P NMR spectroscopy over several days, a transformation which accelerated upon heating to 80 °C without reaching full completion. The reduced symmetry between the two (PNP)M‐complex moieties in the heterodinuclear reaction product was reflected in the observation of two ^31^P NMR resonances of equal intensity for this species which represent the two distinct molecular units (Figure 9c). Additionally, a small degree of decomposition into the respective hydrido complexes occurred under prolonged exposure to elevated temperatures as previously observed for the isolated [(^tBu^PNP)Pd^I^] metalloradical.^[^ ^17^ ^]^

Given the observed ratio of the heterodinuclear complex [(^Et^PNP)Ni–Pd(^Et^PNP)] (**7^Et^‐NiPd**) in equilibrium with the homodinuclear reactants, as determined by integration of the ^31^P‐NMR resonances, the heterodinuclear species was estimated to be at least 1.7 kcal mol^−1^ more stable (per molecule **7^Et^‐NiPd**) than its homodinuclear analogs **4^Et^‐Ni** and **4^Et^‐Pd**. This corresponds well to the DFT‐modeled stabilization of the heterodimetallic complex by a value of ∆*G* = −0.6 kcal mol^−1^ (CPCM(toluene)/r^2^SCAN‐3c/Def2‐mTZVP with ΔΔ*E*_int_ from EDA, vide infra).

To determine the origin of the enhanced stability of complex **7^Et^‐NiPd** relative to its homodinuclear precursors, energy decomposition analyses were again performed. The total steric interactions in **7^Et^‐NiPd** are less destabilizing than those in **4^Et^‐Ni** (mainly owing to diminished Pauli repulsion) but equally more destabilizing with respect to **4^Et^‐Pd** (due to weaker electrostatic contributions). Consequently, the net steric energy change relative to the reactants is nearly neutral. While the Ni–Pd orbital interaction strength equals that of Ni–Ni in **4^Et^‐Ni**, it is less stabilized compared to Pd–Pd in **4^Et^‐Pd**, and thus leads to a destabilization of 3.2 kcal mol^−1^ relative to the combined educts. Dispersion interactions are comparable between **4^Et^‐Ni** and **7^Et^‐NiPd**. However, in the latter, they provide greater stabilization than in **4^Et^‐Pd** (−6.0 kcal mol^−1^). This results in a total intrinsic interaction stabilization of ΔΔ*E*_int_ = −3.2 kcal mol^−1^ for **7^Et^‐NiPd** relative to the homodinuclear complexes. Theoretical quantification of potential stability contributions arising from bond polarity was attempted but precluded due to inconsistent partial charges obtained by different charge analysis methods.

The new product displayed two triplet signals in its ^31^P NMR spectrum, which exhibited cross‐peaks in ^31^P/^31^P correlation spectroscopy (COSY) with a ^3^ *J*‐coupling constant of 10.5 Hz. This signal and *J*‐coupling pattern represents two sets of two chemically inequivalent phosphorus nuclei and thus the reduced symmetry of **7^Et^‐NiPd**, a heterodinuclear dimer with an unsupported Ni–Pd bond, compared to the reactants from which it was formed. The heterodimetallic composition was further confirmed by mass spectrometry (LIFDI‐MS, *m*/*z* 1128.5).

Additional evidence for the formation of the heterodimetallic complex **7^Et^‐NiPd** containing an unsupported Ni–Pd bond was obtained by X‐ray diffraction of single crystals obtained from the reaction mixture (Figure 9b). However, in view of the approximate 1:1 metal disorder of nickel and palladium between the two sites due to crystal symmetry, the determined average Ni–Pd bond length of 2.464(6) Å needs to be interpreted with some care. We note that treating the data as arising from mixture of cocrystallized (and disordered) homodimers [(^Et^PNP)Ni]_2_ and [(^Et^PNP)Pd]_2_ would yield Ni–Ni and Pd–Pd distances of 2.292 and 2.640 Å, respectively, which deviates significantly from those previously determined for **4^Et^‐Ni** and **4^Et^‐Pd**, thus ruling out such a scenario in the case at hand. We note that—to our knowledge—complex **7^Et^‐NiPd** represents the first example of a heterobimetallic Ni–Pd system with an unsupported metal–metal bond.

To reduce structural disorder, crossover experiments were attempted between the pairs **3^iPr^‐Ni** and **4^iPr^‐Pd**, **3^iPr^‐Ni** and **4^Et^‐Pd** as well as **4^Et^‐Ni** and **4^iPr^‐Pd**. Reactivity was observed only for the latter mixture. This reaction, however, was accompanied by metal and ligand scrambling, prompting ongoing investigation into its mechanism.

## Conclusion

This study demonstrates the decisive influence of steric ligand parameters along with residual intramolecular flexibility on the stability and dissociation behavior of unsupported M(I)–M(I) dimers (M = Ni, Pd). As expected, reduced phosphine sterics (Et versus *^i^* Pr) stabilize the metal–metal bond by diminishing steric repulsion and thus lead to favored dimer formation. Nevertheless, the equilibrium between monomeric and dimeric species remains dynamic, as evidenced by NMR studies on the [(*^i^* ^Pr^PNP)Pd]_2_ complex.

We have shown that steric modification of the PNP ligand enables precise tuning of metal–metal bond strength while preserving dynamic equilibria. Metal radical dissociation pathways are kinetically facilitated by ligand conformational flexibility without drastically destabilizing the dimeric ground state. While the rigid backbone maintains steric strain at the Pd–Pd bond, conformational adaptation of the side arms facilitates energetically favorable dissociation. This effect arises from a pre‐organized monomer geometry within the dimer state and is followed by subsequent geometrical relaxation.

Computational investigations of the [(^Et^PNP)Ni]_2_ dimer reveal an unexpectedly accessible triplet state. This twisted dimer structure might represent an additional (reactive) intermediate in the dissociation pathway and could mediate alternative patterns of reactivity that are distinct from those of the open‐shell monomers. These findings may provide the foundation for designing transition metal catalysts with controllable radical activity.

## Supporting Information

The authors have cited additional references within the Supporting Information.^[^ ^110^, ^111^, ^112^, ^113^, ^114^, ^115^, ^116^, ^117^, ^118^, ^119^, ^120^, ^121^, ^122^, ^123^, ^124^, ^125^, ^126^, ^127^, ^128^, ^129^, ^130^, ^131^, ^132^, ^133^, ^134^, ^135^, ^136^, ^137^, ^138^, ^139^, ^140^, ^141^, ^142^, ^143^, ^144^, ^145^, ^146^, ^147^, ^148^, ^149^, ^150^, ^151^, ^152^, ^153^, ^154^, ^155^, ^156^, ^157^, ^158^, ^159^, ^160^, ^161^, ^162^, ^163^, ^164^, ^165^, ^166^, ^167^, ^168^, ^169^ ^]^

## Author Contributions

Conceptualization: TB, LHG. Methodology: TB. Investigation: TB (lead), VJK, FB, JB (crystallography). Formal analysis: TB. Visualization: TB. Writing: TB and LHG. Project administration: LHG. Funding acquisition: LHG. All authors have read and agreed to the published version of the manuscript.

## Conflict of Interests

The authors declare no conflict of interest.

## Supporting information



Supporting Information

Supporting Information

## Data Availability

The data that support the findings of this study are available in the Supporting Information of this article.
