# Supplementary material for: Dynamics of Metal–Metal Bond Dissociation in Pd–Pd and Ni–Ni Complexes: Reorganization and Redistribution Reactions of the Metalloradicals
Source: Angew Chem Int Ed Engl. 2025 Sep 4;64(44):e202514965. doi: 10.1002/anie.202514965 (PMC12559469; doi:10.1002/anie.202514965)
Supplement: Supplementary file 1 — Supporting Information [file ANIE-64-e202514965-s001.pdf]

Supporting Information

Dynamics of Metal–Metal Bond Dissociation in Pd–Pd and Ni–Ni Complexes: Reorganization and Redistribution Reactions of the Metalloradicals

Tim Bruckhoff, Vincenz J. Kohler, Felix Braun, Joachim Ballmann and Lutz H. Gade*

Anorganisch-Chemisches Institut, Universität Heidelberg, Im Neuenheimer Feld 276, 69120 Heidelberg, Germany.

Table of Contents

1) Experimental Procedures ... 3

1.1) General Remarks ... 3

1.2) Ligand Synthesis and Characterization Data ... 4

1.3) Precursor Synthesis and Characterization Data ... 5

1.4) Metal(I) (T-shaped & homodinuclear) Synthesis and Characterization Data ... 7

1.5) Metal(I) (heterodinuclear) Synthesis and Characterization Data ... 9

2) NMR spectra ... 10

3) EPR spectroscopy ... 73

4) UV-VIS spectroscopy ... 75

5) X-ray characterization data ... 80

6) IR spectroscopy ... 95

7) Kinetic and Thermodynamic Measurements ... 101

8.1) First Isomerization Barrier of 4^{iPr}-Pd ... 101

8.2) Thermodynamics of a Low-Energy Conformer of 4^{iPr}-Pd ... 102

8.3) Thermodynamics of the Reversible Pd–Pd Bond Dissociation in $[(^{iPr}PNP)Pd]_2$ ... 103

8.4) Exchange Kinetics from Line Broadening Analysis for the Reversible Bond Dissociation in $[(^{iPr}PNP)Pd]_2$ ... 106

8.5) Thermodynamics of the Equilibrium between 7^{Et}-NiPd and 4^{Et}-Pd & 4^{Et}-Ni ... 107

9) Variable Temperature NMR experiments of 4^{Et}-Ni and 4^{Et}-Pd ... 108

10) Computational Details ... 110

10.1) NMR Simulations for T-shaped Pd(I) and Ni(I) complexes ... 111

10.2) EPR results ... 123

10.3) CASSCF results (3^{iPr}-Ni) ... 124

10.4) UV/Vis ... 127

10.5) Analysis of experimentally found conformations of 4^{iPr}-Pd ... 134

10.6) Modeling Results for the Dissociation of Homodinuclear Dimers ... 135

10.7) Isomerization Mechanism of 4^{iPr}-Pd ... 136

10.8) Monomerizations starting at 4’-Pd, 4’’-Pd, 4’’’-Pd ... 140

10.9) Triplet Structures ... 142

10.10) Energy Decomposition Analysis (EDA) ... 144

11) References ... 146

1) Experimental Procedures

1.1) General Remarks

All manipulations were performed under dry and oxygen-free argon using standard Schlenk techniques or within a glovebox unless otherwise specified, using oven-dried glassware. All solvents were dried and distilled prior to use. Benzene was refluxed over potassium, toluene was dried extensively over potassium for several days at room temperature. Solvents including C_6D_6, toluene-d_8 (tol-d_8), tetrahydrofuran-d_8 (thf-d_8) tetrahydrofuran (THF), 2-methyltetrahydrofuran, and *n*-pentane were dried for multiple days over sodium. Triethylamine (NEt_3) and dichloromethane-d_2 (DCM-d_2) were dried with calcium hydride. All solvents subsequently were degassed by three freeze-pump-thaw cycles (after distillation) and stored under argon within glass ampules sealed with Teflon plugs. Anhydrous diethyl ether (Et_2O), dichloromethane (DCM), and *n*-hexane were obtained anhydrous from Honeywell and purified *via* an MBraun Solvent Purification System (SPS 800) incorporating activated alumina columns. These solvents were similarly stored in argon-filled ampules. Water was degassed prior to use.

The protioligand (^{iPr}PNP)H,[1] tetraethyldiphosphane disulfide ($Et_4P_2S_2$),[2] and diethylphosphane[3] were synthesized according to (modified) literature procedures. All other chemicals were purchased from commercial suppliers and used without further purification. Solid substances, however, underwent drying under vacuum followed by storage under argon prior to use.

Kessil PR160L-390nm and Kessil PR160L-370nm lamps were used for light irradiation (λ = 390 or 370 nm).

Samples for nuclear magnetic resonance (NMR) spectroscopy were prepared within an inert atmosphere utilising 5 mm Wilmad J. Young Tubes. One-dimensional and two-dimensional ^{1}H, ^{13}C, and ^{31}P NMR spectra were recorded using Bruker Avance II (400 MHz) or Bruker Avance III (600 MHz) spectrometers. Chemical shifts are reported in parts per million (ppm) and referenced to residual proton and carbon signals of the respective undeuterated solvent.[4] Coupling constants (*J*) are given in Hertz (Hz). ^{31}P NMR spectra were externally referenced to $P(OMe)_3$ (141.0 ppm with respect to 85 % H_3PO_4 at 0.0 ppm). Signal multiplicities are abbreviated as follows: singlet (s), doublet (d), triplet (t), quartet (q), quintet (quint), heptet (h), combinations thereof (e.g., dt for doublet of triplets), and multiplet (m). Higher-order multiplets arise due to virtual coupling, occurring when the chemical shift difference between coupled nuclei is smaller than their mutual coupling constant ($\Delta\nu < J$) with additional coupling of further nuclei to one of them. This effect was particularly observed in symmetric compounds synthesized within this work, featuring chemically equivalent phosphorus atoms where Δν approaches zero while $J_{P\text{-}P}$ and $J_{X\text{-}P}$ couplings are present. These higher-order multiplets are consistently denoted as “m”. However, where such a multiplet superficially resembles a simpler multiplicity (e.g., triplet), its notation includes both “m” and an apparent pseudo-multiplet prefix (p), such as m[pt] for a signal resembling a pseudotriplet. Coupling constants ($J_{H\text{-}H}$, $J_{H\text{-}P}$, $J_{C\text{-}P}$, $J_{P\text{-}P}$) were determined through iterative fitting of simulated spectra to the experimental data using the daisy module within TopSpin 4.1.3. It is important to note that while $J_{H\text{-}H}$, $J_{H\text{-}P}$ and $J_{C\text{-}P}$ could be determined with good precision, large $J_{P\text{-}P}$ coupling constants (> 50 Hz), which do not produce additional spectral features but only affect relative intensity maxima within a multiplet, were prone to a high error. Consequently, such large $J_{P\text{-}P}$ values are reported as approximate (≈) and represent the value used in simulation. Only smaller $J_{P\text{-}P}$ values generating resolvable spectral features were determinable with good precision and are reported without approximation.

To the best of our knowledge, a detailed analysis of the NMR spectra of dethylphosphane has not yet been reported in the literature. We provide such analysis here as part of its synthesis.

Spectral resolution enhancement was sometimes achieved by modifying window functions (WDW) applied to the Free Induction Decay (FID) before Fourier transformation. Specific parameters including function type, line broadening factor (LB), and Gaussian position (GB where applicable) are detailed within the corresponding figure captions of the NMR spectra. Unless stated otherwise, spectra were processed using an exponential window function with LB values of 0.1 Hz for ^{1}H, 0.6 Hz for ^{13}C, and 1.0 Hz for ^{31}P.

NOESY spectra were recorded using relaxation delays between 1.9 and 2.4 s and mixing times of 0.3 s (**2^{iPr}-Pd**, **4^{Et}-Pd**) or 0.5 s (all other).

Mass spectrometry analyses were conducted at the Department of Organic Chemistry at the University of Heidelberg on either a JEOL AccuTOF GCx mass spectrometer (Liquid Injection Field Desorption Ionisation – LIFDI) or a Bruker ApexQe FT-ICR instrument with electrospray ionisation (ESI). Elemental analyses were performed at the Department of Inorganic Chemistry, University of Heidelberg, using an Elementar varia MICRO cube analyser. X-band electron paramagnetic resonance (EPR) spectra were recorded under inert argon atmosphere utilising a Bruker Elexsys E500 EPR Spectrometer with a B-E25 electromagnet operating at 6 K or a Bruker ESP 300E instrument measuring at room temperature (295 K) and were simulated with the EasySpin v5.2.35 program in MATLAB. Ultraviolet-visible-near infrared (UV/Vis-NIR) spectra were recorded with a Varian Cary 5000 spectrophotometer at room temperature employing a scan rate of 600 nm/min and data point intervals of 1.0 nm. Infrared spectra were recorded on either an Agilent Cary 630 FT-IR spectrometer (ATR) within an argon-filled glovebox or a Bruker Alpha FT-IR instrument.

1.2) Ligand Synthesis and Characterization Data

Synthesis of 1,1,2,2-tetraethyldiphosphane 1,2-disulfide ($Et_4P_2S_2$)

In a 4 L three-necked flask equipped with a dropping funnel and KPG-stirrer, EtMgBr (400 mL, 3 M in Et2O, 1.20 mol, 3.0 eq.) was diluted with Et_2O (200 mL). A solution of $PSCl_3$ (40.6 mL, 392 mmol, 1.0 eq) in Et_2O (60 mL) was added dropwise over 2 h at 0 °C. The reaction mixture warmed to ambient temperature and stirred overnight. The mixture was carefully quenched with H_2O (200 mL), followed by addition of H_2SO_4 (15 mL) diluted in H_2O (400 mL). Stirring continued for further 6 h, before the aqueous layer was extracted under ambient atmosphere with Et_2O (2 x 300 mL). The combined organic phases were washed with H_2O (200 mL), dried over anhydrous $MgSO_4$, filtered, and concentrated *in vacuo*. The crude product was precipitated from the resulting yellow oil by the addition of H_2O (100 mL), washed with H_2O (300 mL), and dried *in vacuo*. Recrystallization from hot methanol (400 mL) afforded the product as a colorless crystalline powder. Concentrating the mother liquor and cooling to -40 °C yielded a second crop increasing the total yield to 69% (32.7 g, 135 mmol). The NMR data matched the literature report.[5]

Synthesis of $HPEt_2$

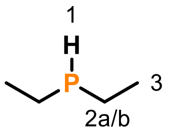

A mixture of $Et_4P_2S_2$ (15.0 g, 61.9 mmol, 1.0 eq.), $(^nBu)_3P$ (29.9 mL, 121 mmol, 2.0 eq.) and H_2O (1.12 g, 61.9 mmol, 1.0 eq.) was combined in a distillation apparatus equipped with a Vigreux column. The mixture was heated to 160 °C, and the product was isolated by distillation over 2 h while gradually increasing the temperature from 160 °C to 200 °C. A stationary vacuum was applied during the final 30 min. The product was obtained as a colorless liquid (4.13, 45.8 mmol, 49 %). **^{1}H NMR** (thf-d_8, 600 MHz, 295 K): δ [ppm] = 2.96 (dtt, $^1J_{H1\text{-}P}$ = 190 Hz, $^3J_{H1\text{-}H2b}$ = 7.06 Hz, $^3J_{H1\text{-}H2a}$ = 6.50 Hz, 1H, H_1), 1.59 (dqd, $^2J_{H2a\text{-}H2b}$ = 13.80 Hz, $^3J_{H2a\text{-}H3}$ = 7.75 Hz, $^3J_{H2a\text{-}H1}$ = 6.50 Hz, 2H, H_{2a}), 1.49 (dqdd, $^2J_{H2a\text{-}H2b}$ = 13.8 Hz, $^3J_{H2b\text{-}H3}$ = 7.61 Hz, $^3J_{H2b\text{-}H1}$ = 7.06 Hz, $^2J_{H2b\text{-}P}$ = 4.71 Hz, 2H, H_{2b}), 1.10 (ddd, $^3J_{H3\text{-}P}$ = 13.4 Hz, $^3J_{H3\text{-}H2a}$ = 7.75 Hz, $^3J_{H3\text{-}H2b}$ = 7.61 Hz, 6H, H_3); **^{1}H{^{31}P} NMR** (thf-d_8, 600 MHz, 295 K): δ [ppm] = 2.96 (tt, $^3J_{H1\text{-}H2b}$ = 7.06 Hz, $^3J_{H1\text{-}H2a}$ = 6.50 Hz, 1H, H_1), 1.59 (dqd, $^2J_{H2a\text{-}H2b}$ = 13.8 Hz, $^3J_{H2a\text{-}H3}$ = 7.75 Hz, $^3J_{H2a\text{-}H1}$ = 6.50 Hz, 2H, H_{2a}), 1.49 (dqd, $^2J_{H2a\text{-}H2b}$ = 13.8 Hz, $^3J_{H2b\text{-}H3}$ = 7.61 Hz, $^3J_{H2b\text{-}H1}$ = 7.06 Hz, 2H, H_{2b}), 1.10 (dd, $^3J_{H3\text{-}H2a}$ = 7.75 Hz, $^3J_{H3\text{-}H2b}$ = 7.61 Hz, 6H, H_3); **^{13}C NMR** (thf-d_8, 151 MHz, 295 K): δ [ppm] = 13.5 (d, $^1J_{C\text{-}P}$ = 9.9 Hz, 2C, C_2), 12.7 (d, $^2J_{C\text{-}P}$ = 10.0 Hz, 2C, C_3); **^{31}P NMR** (thf-d_8, 243 MHz, 295 K): δ [ppm] = 56.9 (dht, $^1J_{H1\text{-}P}$ = 190 Hz, $^3J_{H3\text{-}P}$ = 13.4 Hz, $^2J_{H2b\text{-}P}$ = 4.71 Hz, 1P); **^{31}P{^{1}H} NMR** (thf-d_8, 243 MHz, 295 K): δ [ppm] = 56.9 (s, 1P).

Synthesis of $[(^{Et}PNP)H_3]Br_2$

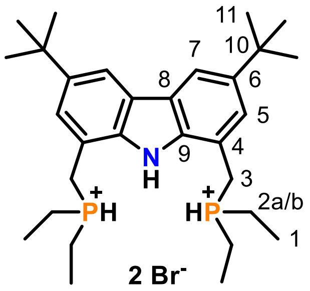

A solution of diethylphosphane (3.14 g, 34.8 mmol, 2.7 eq.) in DCM (12 mL) was added dropwise to a stirred solution of 1,8-bis(bromomethyl)-3,6-di-*tert*-butyl-9*H*-carbazole (6.00 g, 12.9 mmol, 1.0 eq.) in DCM (150 mL) over 40 min. The color changed from red-black to pale orange during the addition. After removal of the solvent under reduced pressure, the solid crude product was washed with *n*-pentane (25 mL) and Et_2O (10 mL). The product was dried *in vacuo* and obtained as a colorless powder (7.35 g, 11.4 mmol, 88%). **^{1}H NMR** (CD_2Cl_2, 600 MHz, 295 K): δ [ppm] = 11.94 (s, 1H, N**H**), 8.17 (dttt[pds], $^1J_{H\text{-}P}$ = 517 Hz, $^3J_{H\text{-}H3}$ = 6.97 Hz, $^3J_{H\text{-}H2b}$ = 5.19 Hz, $^3J_{H\text{-}H2a}$ = 2.97 Hz, 2H, P**H**), 8.10 (dd, $^6J_{H\text{-}P}$ = 2.38 Hz, $^4J_{H\text{-}H}$ = 1.85 Hz, 2H, H_7), 7.37 (dd, $^4J_{H\text{-}P}$ = 2.79 Hz, $^4J_{H\text{-}H}$ = 1.85 Hz, 2H, H_5), 4.38 (dd, $^2J_{H\text{-}P}$ = 15.6 Hz, $^3J_{H\text{-}H3}$ = 6.97 Hz, 4H, H_3), 2.51 (ddqd, $^2J_{H2a\text{-}H2b}$ = 15.5 Hz, $^2J_{H\text{-}P}$ = 13.4 Hz, $^3J_{H2a\text{-}H1}$ = 7.75 Hz, $^3J_{P\mathbf{H}\text{-}H2a}$ = 2.97 Hz, 4H, H_{2a}), 2.35 (ddqd, $^2J_{H2a\text{-}H2b}$ = 15.5 Hz, $^2J_{H\text{-}P}$ = 13.7 Hz, $^3J_{H2b\text{-}H1}$ = 7.77 Hz, $^3J_{P\mathbf{H}\text{-}H2b}$ = 5.19 Hz, 4H, H_{2b}), 1.44 (s, 18H, H_{11}), 1.29 (ddd, $^3J_{H\text{-}P}$ = 19.7 Hz, $^3J_{H2a\text{-}H1}$ = $^3J_{H2b\text{-}H1}$ = 7.77 Hz, 7.75 Hz, 12H, H_1); **^{1}H{^{31}P} NMR** (CD_2Cl_2, 600 MHz, 295 K): δ [ppm] = 11.94 (s, 1H, N**H**), 8.17 (ttt[ps]), $^3J_{H\text{-}H3}$ = 6.97 Hz, $^3J_{H\text{-}H2b}$ = 5.19 Hz, $^3J_{H\text{-}H2a}$ = 2.97 Hz, 2H, P**H**), 8.10 (d, $^4J_{H\text{-}H}$ = 1.85 Hz, 2H, H_7), 7.37 (d, $^4J_{H\text{-}H}$ = 1.85 Hz, 2H, H_5), 4.38 (d, $^3J_{H\text{-}H3}$ = 6.97 Hz, 4H, H_3), 2.51 (dqd, $^2J_{H2a\text{-}H2b}$ = 15.5 Hz, $^3J_{H2a\text{-}H1}$ = 7.75 Hz, $^3J_{H\text{-}H2a}$ = 2.97 Hz, 4H, H_{2a}), 2.35 (dqd, $^2J_{H2a\text{-}H2b}$ = 15.5 Hz, $^3J_{H2b\text{-}H1}$ = 7.77 Hz, $^3J_{H\text{-}H2b}$ = 5.19 Hz, 4H, H_{2b}), 1.44 (s, 18H, H_{11}), 1.29 (dd, $^3J_{H2b\text{-}H1}$ = 7.77 Hz, $^3J_{H2a\text{-}H1}$ = 7.75 Hz, 12H, H_1); **^{13}C NMR** (CD_2Cl_2, 151 MHz, 295 K): δ [ppm] = 143.8 (d, $^4J_{C\text{-}P}$ = 3.0 Hz, 2C, C_6), 138.0 (d, $^3J_{C\text{-}P}$ = 4.4 Hz, 2C, C_9), 125.6 (d, $^3J_{C\text{-}P}$ = 6.3 Hz, 2C, C_5), 124.5 (d, $^4J_{C\text{-}P}$ = 2.3 Hz, 2C, C_8), 117.4 (d, $^5J_{C\text{-}P}$ = 3.7 Hz, 2C, C_7), 111.1 (d, $^2J_{C\text{-}P}$ = 8.7 Hz, 2C, C_4), 35.0 (s, 2C, C_{10}), 32.0 (s, 6C, C_{11}), 23.7 (d, $^1J_{C\text{-}P}$ = 44.2 Hz, 2C, C_3), 10.7 (d, $^1J_{C\text{-}P}$ = 47.3 Hz, 4C, C_2), 6.8 (d, $^2J_{C\text{-}P}$ = 5.9 Hz, 4C, C_1); **^{31}P NMR** (CD_2Cl_2, 243 MHz, 295 K): δ [ppm] = 7.3 (dhttdd, $^1J_{H\text{-}P}$ = 517 Hz, $^3J_{H\text{-}P}$ = 19.7 Hz, $^2J_{H3\text{-}P}$ = 15.6 Hz, $^2J_{H2b\text{-}P}$ = 13.7 Hz, $^2J_{H2a\text{-}P}$ = 13.4 Hz, $^4J_{H\text{-}P}$ = 2.79 Hz, $^6J_{H\text{-}P}$ = 2.38 Hz, 2P); **^{31}P{^{1}H} NMR** (CD_2Cl_2, 243 MHz, 295 K): δ [ppm] = 7.32 (s, 2P). **IR** [cm^{-1}]: ṽ = 3124, 3120, 3116, 3028, 3024, 3020, 2999, 2950, 2904, 2865, 2802, 2751, 2745, 1594, 1493, 1481, 1458, 1442, 1403, 1396, 1362, 1323, 1306, 1284, 1261, 1240, 1230, 1202, 1174, 1146, 1089, 1047, 1016, 924, 872, 842, 799, 752, 722, 659; **MS** (LIFDI, DMC): calc. for $[C_{30}H_{49}Br_2NP_2]^+$: 643.17, found: 483.28 ($[M\text{-}(HBr)_2]^+$, $[C_{30}H_{47}NP_2]^+$, theor: 483.32). **EA**: Anal. calc. for $C_{30}H_{49}Br_2NP_2$ (645.48 g/mol): C 55.82, H 7.65, N 2.17. Found: C 56.78, H 7.72, N 2.12.

Synthesis of (^{Et}PNP)H (1^{Et})

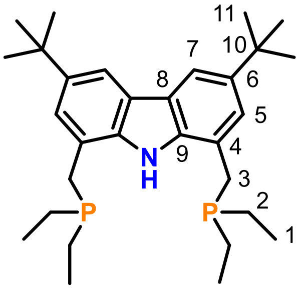

NEt_3 (15.5 mL, 110 mmol, 10 eq.) was added dropwise to a stirred solution of [(^{Et}PNP)H_3]Br_2 (7.10 g, 11.0 mmol, 1.0 eq.) in DCM (100 mL) over 20 min. The color of the solution changed from orange-red to yellow with formation of a colorless precipitate. After stirring for 1 h, the solvent was removed under reduced pressure. The solid was extracted with *n*-pentane (3 x 30 mL) and filtered through a pad of Celite®. Concentrating the filtrate to ~65 mL and crystallization at -40 °C afforded the product in a first crop. Further concentrating the mother liquor and re-cooling yielded a second crop. The collected crystals were dried *in vacuo* yielding the product as a colorless solid (4.41 g, 9.12 mmol, 83 %). Single crystals suitable for X-ray diffraction were grown from a saturated *n*-pentane solution at -40 °C. **^{1}H NMR** (C_6D_6, 600 MHz, 295 K): δ [ppm] = 9.11 (t, $J_{H\text{-}P}$ = 4.0 Hz, 1H, N**H**), 8.22 (m, 2H, H_7), 7.38 (m, 2H, H_5), 2.96 (m, 4H, H_3), 1.48 (s, 18H, H_{11}), 1.28 (q, $^{3}J_{H\text{-}H}$ = 7.67 Hz, 8H, H_2), 0.95 (m, $|^{2}J_{P\text{-}P}|$ = 8 Hz [$\Delta\nu_{P\text{-}P}$ = 0.005 ppm], $^{3}J_{H\text{-}P}$ = 14.4 Hz, $^{3}J_{H\text{-}H}$ = 7.67 Hz, 12H, H_1); **^{1}H{^{31}P} NMR** (C_6D_6, 600 MHz, 295 K): δ [ppm] = 9.11 (s, 1H, N**H**), 8.22 (d, $^{4}J_{H\text{-}H}$ = 1.60 Hz, 2H, H_7), 7.38 (d, $^{4}J_{H\text{-}H}$ = 1.60 Hz, 2H, H_5), 2.96 (s, 4H, H_3), 1.48 (s, 18H, H_{11}), 1.28 (q, $^{3}J_{H\text{-}H}$ = 7.67 Hz, 8H, H_2), 0.95 (t, $^{3}J_{H\text{-}H}$ = 7.67 Hz, 12H, H_1); **^{13}C NMR** (C_6D_6, 151 MHz, 295 K): δ [ppm] = 142.4 (s, 2C, C_6), 138.0 (m[pt], $|^{2}J_{P\text{-}P}|$ = 8 Hz [$\Delta\nu_{P\text{-}P}$ = 0 ppm], $J_{C\text{-}P}$ = 3.2 Hz, 2C, C_9), 124.6 (s, 2C, C_8), 124.4 (m[pt], $|^{2}J_{P\text{-}P}|$ = 8 Hz [$\Delta\nu_{P\text{-}P}$ = 0 ppm], $^{3}J_{C\text{-}P}$ = 4.5 Hz, 2C, C_5), 120.7 (m[pt], $|^{2}J_{P\text{-}P}|$ = 8 Hz [$\Delta\nu_{P\text{-}P}$ = 0 ppm], $J_{C\text{-}P}$ = 4.5 Hz, 2C, C_4), 114.4 (s, 2C, C_7), 34.5 (s, 2C, C_{10}), 32.0 (s, 6C, C_{11}), 31.7 (m, $|^{2}J_{P\text{-}P}|$ = 8 Hz [$\Delta\nu_{P\text{-}P}$ = 0.045 ppm], $^{1}J_{C\text{-}P}$ = 16.0 Hz, 2C, C_3), 19.5 (m, $|^{2}J_{P\text{-}P}|$ = 8 Hz [$\Delta\nu_{P\text{-}P}$ = 0.035 ppm], $^{1}J_{C\text{-}P}$ = 13.0 Hz, 4C, C_2), 9.6 (m, $|^{2}J_{P\text{-}P}|$ = 8 Hz [$\Delta\nu_{P\text{-}P}$ = 0.003 ppm], $^{2}J_{C\text{-}P}$ = 13.5 Hz, 4C, C_1); **^{31}P NMR** (C_6D_6, 243 MHz, 295 K): δ [ppm] = -24.4 (m, 2P), due to missing coupling information no interpretation of this multiplet was possible; **^{31}P{^{1}H} NMR** (C_6D_6, 243 MHz, 295 K): δ [ppm] = -24.4 (s, 2P); **IR** [cm^{-1}]: ṽ = 3394, 2953, 2931, 2904, 2871, 1592, 1492, 1481, 1455, 1440, 1412, 1393, 1379, 1361, 1321, 1301, 1244, 1201, 1188, 1144, 1135, 1036, 1028, 1009, 987, 977, 945, 935, 928, 896, 864, 853, 831, 810, 770, 746, 732, 713, 701, 672; **UV-VIS** [nm] (in *n*-pentane): 344 (ε = 5403 $M^{-1}cm^{-1}$), 330 (ε = 5443 $M^{-1}cm^{-1}$), 297 (ε = 23329 $M^{-1}cm^{-1}$) , 287 (ε = 11801 $M^{-1}cm^{-1}$) , 266 (ε = 19869 $M^{-1}cm^{-1}$); **MS** (LIFDI, *n*-pentane): calc. for $[C_{30}H_{47}NP_2]^+$: 483.32, found: 483.28. **EA**: Anal. calc. for $C_{30}H_{47}NP_2$ (483.66 g/mol): C 74.50, H 9.80, N 2.90. Found: C 74.54, H 10.06, N 3.05.

1.3) Precursor Synthesis and Characterization Data

Synthesis of complex [(^{iPr}PNP)PdCl] (2^{iPr}-Pd)

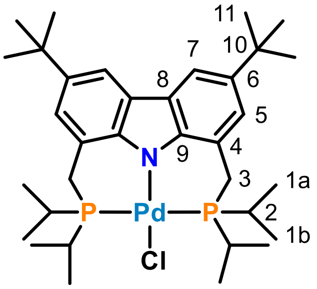

A solution of lithium bis(trimethylsilyl)amide (97%, 345 mg, 2.00 mmol, 1.08 eq.) in THF (8 mL) was added dropwise to a stirred solution of (^{iPr}PNP)H (1.0 g, 1.85 mmol, 1.0 eq.) in THF (4 mL) at room temperature. The yellow reaction mixture was diluted with additional THF (8 mL) and stirred for 10 min. Dichloro(1,5-cyclooctadiene)palladium(II) (99%, 566 mg, 1.96 mmol, 1.06 eq.) was added in portions, followed by dilution with further THF (8 mL). The deep red-orange mixture was stirred for 14 h at room temperature in the dark and then irradiated with blue light (λ = 390 nm) while stirring for an additional 24 h. After removal of the solvent under reduced pressure, the residue was dissolved in toluene (40 mL) and filtered through a pad of Celite®. The pad was rinsed with toluene (20 mL). The solvent of the filtrate was evaporated under reduced pressure, and the crude product washed with *n*-pentane (5 x 6 mL). The product was obtained after drying *in vacuo* as a bright orange powder (1.10 g, 1.62 mmol, 87%). Single crystals suitable for X-ray diffraction were grown from a saturated Et_2O solution at -40 °C. **^{1}H NMR** (C_6D_6, 600 MHz, 295 K): δ [ppm] = 8.36 (d, $^{4}J_{H\text{-}H}$ = 2.00 Hz, 2H, H_7), 7.24 (d, $^{4}J_{H\text{-}H}$ = 2.00 Hz, 2H, H_5), 2.87 (m[pt], $|^{2}J_{P\text{-}P}|$ ≈ 165 Hz, $^{2}J_{H\text{-}P}$ = 4.9 Hz, 4H, H_3), 2.35 (m, $|^{2}J_{P\text{-}P}|$ ≈ 165 Hz, $^{3}J_{H\text{-}H1b}$ = 7.34 Hz, $^{3}J_{H\text{-}H1a}$ = 6.89 Hz, $^{2}J_{H\text{-}P}$ = 6.60 Hz, 4H, H_2), 1.56 (s, 18H, H_{11}), 1.32 (m[pq], $|^{2}J_{P\text{-}P}|$ ≈ 165 Hz, $^{3}J_{H\text{-}P}$ = 15.5 Hz, $^{3}J_{H\text{-}H}$ = 7.34 Hz, 12H, H_{1b}), 0.90 (m[pq], $|^{2}J_{P\text{-}P}|$ ≈ 165 Hz, $^{3}J_{H\text{-}P}$ = 14.0 Hz, $^{3}J_{H\text{-}H}$ = 6.89 Hz, 12H, H_{1a}); **^{1}H{^{31}P} NMR** (C_6D_6, 600 MHz, 295 K): δ [ppm] = 8.36 (d, $^{4}J_{H\text{-}H}$ = 2.00 Hz, 2H, H_7), 7.24 (d, $^{4}J_{H\text{-}H}$ = 2.00 Hz, 2H, H_5), 2.87 (s, 4H, H_3), 2.35 (h, $^{3}J_{H\text{-}H1b}$ = 7.34 Hz, $^{3}J_{H\text{-}H1a}$ = 6.89 Hz, 4H, H_2), 1.56 (s, 18H, H_{11}), 1.32 (d, $^{3}J_{H\text{-}H}$ = 7.34 Hz, 12H, H_{1b}), 0.90 (d, $^{3}J_{H\text{-}H}$ = 6.89 Hz, 12H, H_{1a}); **^{13}C{^{1}H} NMR** (C_6D_6, 151 MHz, 295 K): δ [ppm] = 148.4 (m[pt], $|^{2}J_{P\text{-}P}|$ ≈ 165 Hz, $^{3}J_{C\text{-}P}$ = 8.0 Hz, 2C, C_9), 139.6 (s, 2C, C_6), 126.6 (s, 2C, C_8), 123.7 (m[pt], $|^{2}J_{P\text{-}P}|$ ≈ 165 Hz, $^{3}J_{C\text{-}P}$ = 6.7 Hz, 2C, C_5), 119.7 (s, 2C, C_4), 115.3 (s, 2C, C_7), 34.3 (s, 2C, C_{10}), 32.1 (s, 6C, C_{11}), 24.4 (m[pt], $|^{2}J_{P\text{-}P}|$ ≈ 165 Hz, $^{1}J_{C\text{-}P}$ = 23.1 Hz, 4C, C_2), 19.9 (m[pt], $|^{2}J_{P\text{-}P}|$ ≈ 165 Hz, $^{1}J_{C\text{-}P}$ = 18.5 Hz, 4C, C_3), 19.4 (m[pt], $|^{2}J_{P\text{-}P}|$ ≈ 165 Hz, $^{2}J_{C\text{-}P}$ = 3.0 Hz, 4C, C_{1b}), 17.7 (s, 4C, C_{1a}); **^{31}P NMR** (C_6D_6, 243 MHz, 295K): δ [ppm] = 57.3 (m[ps], 2P); **IR** [cm^{-1}]: ṽ = 2951, 2897, 2864, 1582, 1473, 1426, 1389, 1360, 1321, 1282, 1265, 1226, 1201, 1187, 1177, 1144, 1098, 1018, 935, 867, 857, 812, 731, 653, 645, 593, 577, 499, 474, 437; **UV-VIS** [nm] (in thf): 466 (ε = 1915 $M^{-1}cm^{-1}$), 375 (ε = 4276 $M^{-1}cm^{-1}$), 361 (ε = 4439 $M^{-1}cm^{-1}$), 311 (ε = 15157 $M^{-1}cm^{-1}$); **MS** (LIFDI, thf): calc. for $[C_{34}H_{54}ClNP_2Pd]^+$: 679.25, found: 679.25. The expected isotopic pattern agrees well with the measurement. **EA**: Anal. calc. for $C_{34}H_{54}ClNP_2Pd$ (680.63 g/mol): C 60.00, H 8.00, N 2.06. Found: C 60.2, H 8.15, N 2.21.

Synthesis of complex [(^{iPr}PNP)NiCl] (2^{iPr}-Ni)

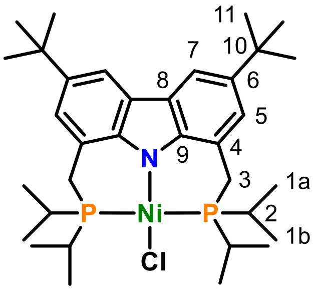

A solution of lithium bis(trimethylsilyl)amide (97%, 201.3 mg, 1.17 mmol, 1.05 eq.) in THF (5 mL) was added dropwise to a stirred solution of (^{iPr}PNP)H (600.0 mg, 1.11 mmol, 1.00 eq.) in THF (15 mL) at room temperature. After stirring for 15 minutes, dichloro(dimethoxyethane)nickel (264.0 mg, 1.20 mmol, 1.08 eq.) was added in portions, resulting in an immediate formation of a green solution. The mixture was stirred overnight at room temperature, filtered through Celite®, and concentrated under reduced pressure. The dry residue was dissolved in toluene, filtered through a pad of Celite® before the solvent was removed *in vacuo*. Washing the crude product with *n*-pentane (3 x 2 mL) afforded the product as a dark green solid (643 mg, 1.02 mmol, 91%). Single crystals suitable for X-ray diffraction were obtained from a saturated toluene/ *n*-pentane solution at -40 °C. **^{1}H NMR** (C_6D_6, 600 MHz, 295 K): δ [ppm] = 8.28 (d, $^4J_{H\text{-}H}$ = 2.10 Hz, 2H, H_7), 7.17 (s.i., d, $^4J_{H\text{-}H}$ = 2.10 Hz, 2H, H_5), 2.68 (m[pt], $|^2J_{P\text{-}P}| \approx 200$ Hz, $^2J_{H\text{-}P}$ = 7.00 Hz, 4H, H_3), 2.19 (m, $|^2J_{P\text{-}P}| \approx 200$ Hz, $^3J_{H\text{-}H1b}$ = 7.28 Hz, $^3J_{H\text{-}H1a}$ = 7.05 Hz, $^2J_{H\text{-}P}$ = 6.92 Hz, 4H, H_2), 1.54 (s, 18H, H_{11}), 1.47 (m[pq], $|^2J_{P\text{-}P}| \approx 200$ Hz, $^3J_{H\text{-}P}$ = 15.5 Hz, $^3J_{H\text{-}H}$ = 7.28 Hz, 12H, H_{1b}), 1.07 (m[pq], $|^2J_{P\text{-}P}| \approx 200$ Hz, $^3J_{H\text{-}P}$ = 13.4 Hz, $^3J_{H\text{-}H}$ = 7.05 Hz, 12H, H_{1a}); **^{1}H{^{31}P} NMR** (C_6D_6, 600 MHz, 295 K): δ [ppm] = 8.28 (d, $^4J_{H\text{-}H}$ = 2.1 Hz, 2H, H_7), 7.17 (s.i., d, $^4J_{H\text{-}H}$ = 2.1 Hz, 2H, H_5), 2.68 (s, 4H, H_3), 2.19 (qq[ph], $^3J_{H\text{-}H1b}$ = 7.28 Hz, $^3J_{H\text{-}H1a}$ = 7.05 Hz, 4H, H_2), 1.54 (s, 18H, H_{11}), 1.47 (d, $^3J_{H\text{-}H}$ = 7.28 Hz, 12H, H_{1b}), 1.07 (d, $^3J_{H\text{-}H}$ = 7.05 Hz, 12H, H_{1a}); **^{13}C{^{1}H} NMR** (C_6D_6, 151 MHz, 295 K): δ [ppm] = 148.6 (m[pt], $|^2J_{P\text{-}P}| \approx 200$ Hz, $^3J_{C\text{-}P}$ = 10.1 Hz, 2C, C_9), 139.8 (s, 2C, C_6), 127.4 (s, 2C, C_8), 122.9 (m[pt], $|^2J_{P\text{-}P}| \approx 200$ Hz, $^3J_{C\text{-}P}$ = 6.85 Hz, 2C, C_5), 119.6 (s, 2C, C_4), 114.9 (s, 2C, C_7), 34.3 (s, 2C, C_{10}), 32.1 (s, 6C, C_{11}), 24.2 (m[pt], $|^2J_{P\text{-}P}| \approx 200$ Hz, $^1J_{C\text{-}P}$ = 23.4 Hz, 4C, C_2), 19.9 (m[pt], $|^2J_{P\text{-}P}| \approx 200$ Hz, $^2J_{C\text{-}P}$ = 3.1 Hz, 4C, C_{1b}), 19.0 (m[pt], $|^2J_{P\text{-}P}| \approx 200$ Hz, $^1J_{C\text{-}P}$ = 15.2 Hz, 4C, C_3), 17.7 (s, 4C, C_{1a}); **^{31}P NMR** (C_6D_6, 243 MHz, 295 K): δ [ppm] = 38.4 (m[ps], 2P); **IR** [cm^{-1}]: ṽ = 2948, 2900, 2866, 1577, 1476, 1457, 1428, 1390, 1360, 1319, 1282, 1252, 1215, 1185, 1159, 1137, 1126, 1096, 1036, 928, 883, 865, 854, 820, 779, 742, 663; **UV-VIS** [nm] (in *n*-pentane): 629 (ε = 507 $M^{-1}cm^{-1}$), 439 (ε = 1375 $M^{-1}cm^{-1}$), 382 (ε = 4630 $M^{-1}cm^{-1}$), 343 (ε = 8075 $M^{-1}cm^{-1}$), 313 (ε = 18996 $M^{-1}cm^{-1}$), 284 (ε = 30375 $M^{-1}cm^{-1}$); **HR-MS** (ESI, DCM/MeOH): calc. for $[C_{34}H_{54}ClNNiP_2]^+$: 631.2773, found: 632.2851 (M-H$^+$). The expected isotopic pattern agrees well with the measurement. **EA**: Anal. calc. for $C_{34}H_{54}ClNNiP_2$ (632.90 g/mol): C 64.52, H 8.60, N 2.21. Found: C 64.72, H 8.84, N 1.96.

Synthesis of complex [(^{Et}PNP)PdCl] (2^{Et}-Pd)

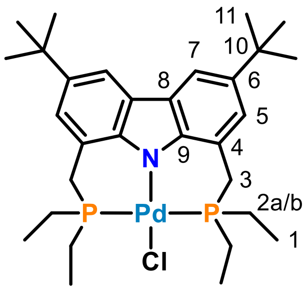

A solution of lithium bis(trimethylsilyl)amide (97%, 425 mg, 2.47 mmol, 1.10 eq.) in THF (5 mL) was added dropwise to a stirring solution of (^{Et}PNP)H (1.09 g, 2.24 mmol, 1.0 eq.) in THF (15 mL), whereby the reaction mixture turned orange. After stirring for 20 minutes, dichloro(1,5-cyclooctadiene)palladium(II) (99%, 679 mg, 2.36 mmol, 1.05 eq.) was added in portions resulting in a dark red solution, which was stirred overnight at room temperature. The solvent was removed under reduced pressure and the remaining solid taken up in toluene (30 mL). The solution was filtered through Celite® and the solvent was removed again. The crude product was washed with *n*-pentane (10 mL) affording the product as a red-orange powder (1.31 g, 2.10 mmol, 94%). Single crystals suitable for X-Ray diffraction were obtained by cooling a saturated toluene solution to -40 °C. **^{1}H NMR** (C_6D_6, 400 MHz, 295 K): δ [ppm] = 8.43 (d, $^4J_{H\text{-}H}$ = 1.90 Hz, 2H, H_7), 7.17 (d, $^4J_{H\text{-}H}$ = 1.90 Hz, 2H, H_5), 2.75 (m[pt], $|^2J_{P\text{-}P}| \approx 130$ Hz, $^2J_{H\text{-}P}$ = 5.50 Hz, 4H, H_3), 1.79 (m, $|^2J_{P\text{-}P}| \approx 130$ Hz, $^2J_{H\text{-}H}$ = 14.4 Hz, $^3J_{H\text{-}H}$ = 7.65 Hz, $^2J_{H\text{-}P} \approx 6.21$ Hz, 4H, H_{2b}), 1.55 (s, 18H, H_{11}), 1.39 (m, $|^2J_{P\text{-}P}| \approx 130$ Hz, $^2J_{H\text{-}H}$ = 14.4 Hz, $^3J_{H\text{-}H}$ = 7.65 Hz, $^2J_{H\text{-}P} \approx 6.81$ Hz, 4H, H_{2a}), 1.01 (m[pquint], $|^2J_{P\text{-}P}| \approx 130$ Hz, $^3J_{H\text{-}P}$ = 16.7 Hz, $^3J_{H\text{-}H}$ = 7.65 Hz, 12H, H_1); **^{1}H{^{31}P} NMR** (C_6D_6, 600 MHz, 295 K): δ [ppm] = 8.43 (d, $^4J_{H\text{-}H}$ = 1.90 Hz, 2H, H_7), 7.17 (d, $^4J_{H\text{-}H}$ = 1.90 Hz, 2H, H_5), 2.75 (s, 4H, H_3), 1.79 (dq, $^2J_{H\text{-}H}$ = 14.4 Hz, $^3J_{H\text{-}H}$ = 7.65 Hz, 8H, H_{2b}), 1.55 (s, 18H, H_{11}), 1.39 (dq, $^2J_{H\text{-}H}$ = 14.4 Hz, $^3J_{H\text{-}H}$ = 7.65 Hz, 8H, H_{2a}), 1.01 (t, $^3J_{H\text{-}H}$ = 7.65 Hz, 12H, H_1); **^{13}C{^{1}H} NMR** (C_6D_6, 151 MHz, 295 K): δ [ppm] = 148.0 (m[pt], $|^2J_{P\text{-}P}| \approx 130$ Hz, $^3J_{C\text{-}P}$ = 9.0 Hz, 2C, C_9), 139.7 (s, 2C, C_6), 126.8 (s, 2C, C_8), 123.7 (m[pt], $|^2J_{P\text{-}P}| \approx 130$ Hz, $^3J_{C\text{-}P}$ = 7.0 Hz, 2C, C_5), 119.1 (s, 2C, C_4), 115.4 (s, 2C, C_7), 34.4 (s, 2C, C_{10}), 32.1 (s, 2C, C_{11}), 23.2 (m[pt], $|^2J_{P\text{-}P}| \approx 130$ Hz, $^1J_{C\text{-}P}$ = 22.5 Hz, 4C, C_3), 16.4 (m[pt], $|^2J_{P\text{-}P}| \approx 130$ Hz, $^1J_{C\text{-}P}$ = 27.0 Hz, 4C, C_2), 8.2 (s, 2C, C_1); **^{31}P NMR** (C_6D_6, 162 MHz, 295 K): δ [ppm] = 42.4 (ps, 2P); **IR** [cm^{-1}]: ṽ = 2956, 2904, 2878, 1569, 1480, 1457, 1424, 1394, 1379, 1360, 1319, 1282, 1260, 1219, 1189, 1137, 1100, 1047, 1029, 1006, 954, 932, 857, 827, 794, 753, 731, 704; **UV-VIS** [nm] (in THF): 455 (ε = 1881 $M^{-1}cm^{-1}$), 360 (ε = 4735 $M^{-1}cm^{-1}$), 310 (ε = 14930 $M^{-1}cm^{-1}$), 274 (ε = 36167 $M^{-1}cm^{-1}$); **MS** (LIFDI, thf): calc. for $[C_{30}H_{46}ClNP_2Pd]^+$: 623.18, found: 625.25. The expected isotopic pattern agrees well with the measurement. **EA**: Anal. calc. for $C_{30}H_{46}ClNP_2Pd$ (624.52 g/mol): C 57.70, H 7.42, N 2.24. Found: C 57.29, H 7.55, N 2.26.

Synthesis of complex [(^{Et}PNP)NiCl] (2^{Et}-Ni)

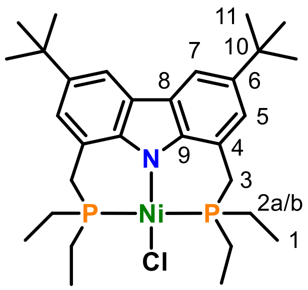

A solution of lithium bis(trimethylsilyl)amide (97%, 763 mg, 4.47 mmol, 1.08 eq.) in THF (10 mL) was added dropwise to a stirring solution of (^{Et}PNP)H (2.0 g, 4.14 mmol, 1.0 eq.) in THF (20 mL) at room temperature. After stirring for 10 minutes, dichloro(dimethoxyethane)nickel (98%, 993 mg, 4.38 mmol, 1.06 eq.) was added as a solid, resulting in a color change from orange to deep red that changed to deep green upon stirring overnight (18 h). The reaction mixture was filtered through a pad of Celite® after which this Celite® pad was rinsed with THF (5-10 mL). The solvent was removed under reduced pressure, the residue dissolved in toluene (20 mL) and subsequently filtered through a Celite® pad again. This pad was rinsed with toluene until a colorless filtrate was obtained. The solvent was removed *in vacuo* followed by washing of the green crude product with *n*-pentane (3-4 x 10 mL). The product was obtained after drying *in vacuo* as a green solid (2.14767 g, 3.72 mmol, 90%). Single crystals suitable for X-Ray diffraction were

grown from a saturated Et_2O solution at -40 °C. **^{1}H NMR** (C_6D_6, 600 MHz, 295 K): δ [ppm] = 8.35 (d, $^4J_{H-H}$ = 2.30 Hz, 2H, H_7), 7.14 (d, $^4J_{H-H}$ = 2.30 Hz, 2H, H_5), 2.65 (m[pt], $|^2J_{P-P}|$ ≈ 138 Hz, $^2J_{H-P}$ = 7.30 Hz, 4H, H_3), 1.72 (m, $|^2J_{P-P}|$ ≈ 138 Hz, $^2J_{H-H}$ = 14.2 Hz, $^3J_{H-H}$ = 7.90 Hz, $^2J_{H-P}$ = 6.9 Hz, 4H, H_{2a}), 1.54 (s, 18H, H_{11}), 1.29 (m, $|^2J_{P-P}|$ ≈ 138 Hz, $^2J_{H-H}$ = 14.2 Hz, $^2J_{H-P}$ = 8.10 Hz, $^3J_{H-H}$ = 7.40 Hz, 4H, H_{2b}), 1.11 (m[pquint], $|^2J_{P-P}|$ ≈ 138 Hz, $^3J_{H-P}$ = 15.5 - 16.6 Hz, $^3J_{H-H}$ = 7.90 Hz, $^3J_{H-H}$ = 7.40 Hz, 12H, H_1); **^{1}H{^{31}P} NMR** (C_6D_6, 600 MHz, 295 K): δ [ppm] = 8.35 (d, $^4J_{H-H}$ = 2.30 Hz, 2H, H_7), 7.14 (d, $^4J_{H-H}$ = 2.30 Hz, 2H, H_5), 2.65 (s, 4H, H_3), 1.72 (dq, $^2J_{H-H}$ = 14.2 Hz, $^3J_{H-H}$ = 7.9 Hz, 4H, H_{2a}), 1.54 (s, 18H, H_{11}), 1.29 (dq, $^2J_{H-H}$ = 14.2 Hz, $^3J_{H-H}$ = 7.4 Hz, 4H, H_{2b}), 1.11 (dd[pt], $^3J_{H-H}$ = 7.9 Hz, $^3J_{H-H}$ = 7.4 Hz, 12H, H_1); **^{13}C{^{1}H} NMR** (C_6D_6, 151 MHz, 295 K): δ [ppm] = 147.7 (m[pt], $|^2J_{P-P}|$ ≈ 138 Hz, $^3J_{C-P}$ = 11.4 Hz, 2C, C_9), 139.8 (s, 2C, C_6), 127.6 (s.i., 2C, C_8), 122.9 (m[pt], $|^2J_{P-P}|$ ≈ 138 Hz, $^3J_{C-P}$ = 7.55 Hz, 2C, C_5), 119.1 (s, 2C, C_4), 115.0 (s, 2C, C_7), 34.3 (s, 2C, C_{10}), 32.1 (s, 6C, C_{11}), 22.0 (m[pt], $|^2J_{P-P}|$ ≈ 138 Hz, $^1J_{C-P}$ = 19.3 Hz, 2C, C_3), 16.0 (m[pt], $|^2J_{P-P}|$ ≈ 138 Hz, $^1J_{C-P}$ = 27.2 Hz, 4C, C_2), 8.1 (s, 4C, C_1); **^{31}P NMR** (C_6D_6, 243 MHz, 295 K): δ [ppm] = 24.1 (dhttt, $|^2J_{P-P}|$ ≈ 138 Hz, $^3J_{H-P}$ = 15.6-16.6 Hz, $^2J_{H-P}$ = 8.10 Hz, $^2J_{H-P}$ = 7.30 Hz, $^2J_{H-P}$ = 6.90 Hz); **^{31}P{^{1}H} NMR** (C_6D_6, 243 MHz, 295 K): δ [ppm] = 24.1 (s, 2P); **IR** [cm^{-1}]: ṽ = 2960, 2939, 2902, 2874, 1568, 1477, 1454, 1426, 1408, 1392, 1377, 1360, 1320, 1313, 1279, 1265, 1242, 1231, 1216, 1204, 1193, 1183, 1136, 1098, 1051, 1032, 1020, 1014, 1005, 999, 952, 935, 874, 863, 855, 837, 824, 811, 796, 790, 768, 751, 732, 699, 687, 658; **UV-VIS** [nm] (in *n*-pentane): 616 (ε = 453 $M^{-1}cm^{-1}$), 429 (ε = 1276 $M^{-1}cm^{-1}$), 381 (ε = 3724 $M^{-1}cm^{-1}$), 366 (ε = 3259 $M^{-1}cm^{-1}$), 326 (ε = 11520 $M^{-1}cm^{-1}$), 312 (ε = 17057 $M^{-1}cm^{-1}$), 284 (ε = 28485 $M^{-1}cm^{-1}$); **MS** (LIFDI, thf): calc. for $[C_{30}H_{46}ClNNiP_2]^+$: 575.21, found: 575.26. The expected isotopic pattern agrees well with the measurement. **EA**: Anal. calc. for $C_{30}H_{46}ClNNiP_2$ (576.80 g/mol): C 62.47, H 8.04, N 2.43. Found: C 62.19, H 8.03, N 2.49.

1.4) Metal(I) (T-shaped & homodinuclear) Synthesis and Characterization Data

Synthesis of complex [(^{iPr}PNP)Pd]$_2$ (4^{iPr}-Pd)

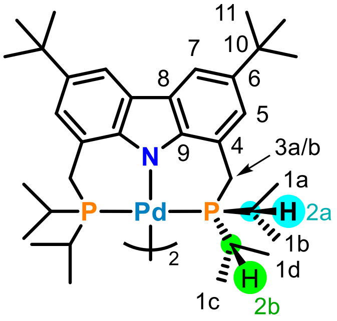

Complex **2^{iPr}-Pd** (1.00 g, 1.47 mmol, 1.0 eq.) and naphthalene (37.7 mg, 294 µmol, 0.2 eq.) were dissolved in THF (20 mL). This solution was rapidly added to a vigorously stirred suspension of sodium (40.5 mg, 1.76 mmol, 1.2 eq) in THF (5 mL), leading to a dark red mixture. After dilution with additional THF (10-20 mL), the reaction mixture was stirred overnight (~18 h) at room temperature. The solvent was removed under reduced pressure, and the dry residue dissolved in benzene (30 mL). After filtration through a pad of Celite® and subsequent rinsing of the Celite® pad with benzene (10 mL), the collected filtrate was evaporated to dryness *in vacuo*. The crude product was washed with *n*-pentane (5 x 8 mL) and subsequently with cold (-40 °C) Et_2O (3-5 mL) to afford the product after drying *in vacuo* as an orange powder (753 mg, 584 µmol, 79%). Single crystals suitable for X-ray diffraction were grown from either a saturated Et_2O or THF solution at -40 °C. **^{1}H NMR** (C_6D_6, 600 MHz, 295 K): δ [ppm] = 8.46 (d, $^4J_{H-H}$ = 1.90 Hz, 4H, H_7), 7.48 (d, $^4J_{H-H}$ = 1.90 Hz, 4H, H_5), 3.61 (bs, $\Delta v_{1/2}$ = 60 Hz, 8H, $H_{3a/b}$), 2.43 (bs, $\Delta v_{1/2}$ = 300 Hz, 8H, $H_{2a/b}$), 1.60 (s, 36H, H_{11}), 1.13 (bs, $\Delta v_{1/2}$ = 36 Hz, 24H, $H_{1b/d}$), 0.75 (bs, $\Delta v_{1/2}$ = 140 Hz, 24H, $H_{1a/c}$); **^{13}C{^{1}H} NMR** (C_6D_6, 151 MHz, 295K): δ [ppm] = 145.6 (s, 4C, C_9), 137.0 (s, 4C, C_6), 125.3 (s, 4C, C_8), 123.7 (m[pt], $^3J_{C-P}$ = 7.5 Hz, 4C, C_5), 118.5 (s, 4C, C_4), 115.1 (s, 4C, C_7), 34.3 (s, 4C, C_{10}), 32.4 (s, 12C, C_{11}), 24.8 (m[pt], $^3J_{C-P}$ = 16.5 Hz, 4C, C_3), 20.2 (s, 8C, $C_{1a/c}$), 18.6 (s, 4C, $C_{1b/d}$); **^{31}P NMR** (C_6D_6, 243 MHz, 295K): δ [ppm] = 9.9 (s, 2P); **^{1}H NMR** (tol-d_8, 600 MHz, 243 K): δ [ppm] = 8.44 (s, 4H, H_7), 7.48 (s, 4H, H_5), 3.80 (d, $^2J_{H-H}$ = 12.9 Hz, 4H, H_{3b}), 3.35 (d, $^2J_{H-H}$ = 12.9 Hz, 4H, H_{3a}), 2.87 (s, 4H, H_{2b}), 1.79 (s, 4H, H_{2a}), 1.63 (s, 36H, H_{11}), 1.25 (s, 12H, H_{1d}), 1.08 (s, 12H, H_{1c}), 0.94 (s, 12H, H_{1b}) , 0.30 (s, 12H, H_{1a}); **^{13}C{^{1}H} NMR** (tol-d_8, 151 MHz, 243K): δ [ppm] = 145.2 (s, 4C, C_9), 136.6 (s, 4C, C_6), 125.0 (s.i., 4C, C_8), 123.6 (s, 4C, C_5), 118.4 (s, 4C, C_4), 114.9 (s, 4C, C_7), 34.3 (s, 4C, C_{10}), 32.8 (s, 4C, C_{2b}), 32.3 (s, 12C, C_{11}), 25.2 (s, 4C, C_{2a}), 24.4 (s, 4C, C_3), 20.5 (s.i., s, 4C, C_{1a}), 19.7 (s, 4C, C_{1c}), 18.5 (s, 4C, C_{1d}), 17.9 (s, 4C, C_{1b}); **^{31}P NMR** (tol-d_8, 243 MHz, 243K): δ [ppm] = 9.6 (s, 4P); **IR** [cm^{-1}]: ṽ 2956, 2904, 2874, 1573, 1461, 1428, 1387, 1360, 1319, 1286, 1234, 1200, 1155, 1141, 1100, 1051, 1032, 936, 883, 865, 820, 786, 760, 738; **MS** (LIFDI, thf): calc. for $[C_{68}H_{108}N_2P_4Pd_2]^+$: 1288.55, found: 1288.53 (minor), 644.28 (major, [(^{iPr}PNP)Pd^I]). The expected isotopic pattern agrees well with the measurement. **EA**: Anal. calc. for $C_{68}H_{108}N_2P_4Pd_2$ (1290.36 g/mol): C 63.30, H 8.44, N 2.17. Found: C 63.60, H 8.83, N 2.19.

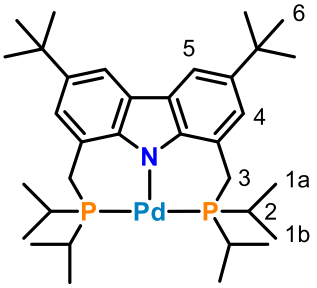

Concomitant detection of the mononuclear **[(^{iPr}PNP)Pd^I]** metalloradical (**3^{iPr}-Pd**) alongside the dimer (**4^{iPr}-Pd**) was possible at temperatures higher than 273 K. X-band **EPR** (295 K, C_6D_6) g = 2.104 (^{105}Pd(22.33%): 119.8 MHz; ^{31}P: 54.9 MHz; ^{14}N 54.9 MHz); **^{1}H NMR** (C_6D_6, 600 MHz, 353 K, deconvoluted): δ [ppm] = 9.07 ($\Delta v_{1/2}$ = 98 Hz, 2H, H_5), 5.07 ($\Delta v_{1/2}$ = 410 Hz, 12H, H_{1b}), 1.87 ($\Delta v_{1/2}$ = 16 Hz, 18H, H_6), 1.18 ($\Delta v_{1/2}$ = 443 Hz, 12H, H_{1a}), -1.28 ($\Delta v_{1/2}$ = 1761 Hz, 4H, H_3); **MS** (LIFDI, thf): calc. for $[C_{34}H_{54}NP_2Pd]^+$: 644.28, found: 644.28. The expected isotopic pattern agrees well with the measurement.

Synthesis of complex [(^{iPr}PNP)Ni(I)] (3^{iPr}-Ni)

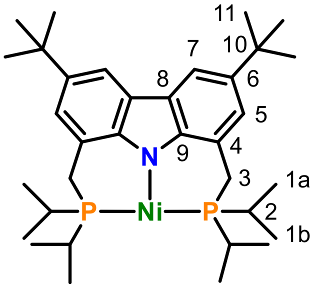

A solution of Complex **2^{iPr}-Ni** (300.0 mg, 473 µmol, 1.0 eq.) in THF (10 mL) was added to sodium-lead alloy (10 w%, 119.8 mg, 520 µmol, 1.1 eq.). The dark green reaction mixture was stirred overnight at room temperature, changing its color to red. The reaction mixture was filtered through Celite® after which the solvent of the filtrate was removed under reduced pressure. The residue was dissolved in toluene and filtered again through a pad of Celite®. The product was obtained after removal of the solvent *in vacuo* as a pale red solid (266.1 mg, 445 µmol, 89%). Single crystals suitable for X-ray diffraction were obtained from a saturated *n*-pentane solution at -40 °C. **^{1}H NMR** (C_6D_6, 600 MHz, 295 K, deconvoluted): δ [ppm] = 30.05 ($\Delta\nu_{1/2}$ = 1490 Hz, 4H, H_2), 22.06 ($\Delta\nu_{1/2}$ = 218 Hz, 2H, H_7), 10.55 ($\Delta\nu_{1/2}$ = 116 Hz, 2H, H_5), 6.62 ($\Delta\nu_{1/2}$ = 500 Hz, 12H, H_{1b}), 1.46 ($\Delta\nu_{1/2}$ = 17 Hz, 18H, H_{11}), 0.28 ($\Delta\nu_{1/2}$ = 622 Hz, 12H, H_{1a}), -18.61 ($\Delta\nu_{1/2}$ = 1688 Hz, 4H, H_3); **^{13}C{^{1}H} NMR** (C_6D_6, 151 MHz, 295K, deconvoluted): δ [ppm] = 187.2 ($\Delta\nu_{1/2}$ = 340 Hz, 2C, C_4), 146.5 ($\Delta\nu_{1/2}$ = 37 Hz, 2C, C_6), 143.3 ($\Delta\nu_{1/2}$ = 145 Hz, 2C, C_7), 103.2 ($\Delta\nu_{1/2}$ = 500 Hz, 4C, C_{1a}), 90.2 ($\Delta\nu_{1/2}$ = 92 Hz, 2C, C_5), 37.5 ($\Delta\nu_{1/2}$ = 58 Hz, 4C, C_{1b}), 37.5 ($\Delta\nu_{1/2}$ = 22 Hz, 2C, C_{11}), 28.5 ($\Delta\nu_{1/2}$ = 24 Hz, 2C, C_{10}); **IR** [cm^{-1}]: ṽ = 2948, 2922, 2892, 2863, 1569, 1457, 1424, 1383, 1360, 1308, 1278, 1226, 1185, 1159, 1129, 1096, 1040, 1021, 924, 876, 865, 850, 820, 734, 678; **UV-VIS** [nm] (in *n*-pentane): 1732 (ε = 98 $M^{-1}cm^{-1}$), 1464 (ε = 86 $M^{-1}cm^{-1}$), 1189 (ε = 48 $M^{-1}cm^{-1}$), 517 (ε = 258 $M^{-1}cm^{-1}$), 385 (ε = 6430 $M^{-1}cm^{-1}$), 363 (ε = 6433 $M^{-1}cm^{-1}$); **MS** (LIFDI, thf): calc. for $[C_{34}H_{54}NNiP_2]^+$: 596.31, found: 596.34. The expected isotopic pattern agrees well with the measurement. **EA**: Anal. calc. for $C_{34}H_{54}NNiP_2$ (597.45 g/mol): C 68.35, H 9.11, N 2.34. Found: C 68.00, H 9.56, N 2.28.

Synthesis of complex [(^{Et}PNP)Pd]$_2$ (4^{Et}-Pd)

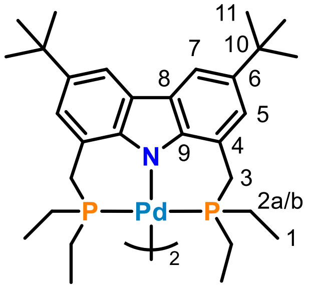

A suspension of sodium powder (25.1 mg, 1.09 mmol, 1.05 eq.) and naphthalene (13.3 mg, 104 µmol, 0.10 eq.) in THF (10 mL) was added dropwise to a cooled solution of **2^{Et}-Pd** (650 mg, 1.04 mmol, 1.0 eq.) in THF (10 mL) at -40 °C. The resulting dark brown mixture was stirred for three days at room temperature. The solvent was removed *in vacuo* and the residue was dissolved in benzene (40 mL). The solution was filtered through a pad of Celite® and the solvent of the collected filtrate was removed again. Washing the crude product with 1:1 Et_2O/ *n*-pentane (40 mL) yielded the product as an ocher-colored powder (320 mg, 543 µmol, 54%). Crystals suitable for X-ray diffraction were obtained by slow evaporation of a saturated toluene solution. **^{1}H NMR** (C_6D_6, 600 MHz, 295 K): δ [ppm] = 8.50 (d, $^4J_{H-H}$ = 1.90 Hz, 2H, H_7), 7.41 (d, $^4J_{H-H}$ = 1.90 Hz, 2H, H_5), 3.22 (s, 4H, H_3), 1.62 (s, 18H, H_{11}), 1.59 (dq, $^2J_{H-H}$ = 14.9 Hz, $^3J_{H-H}$ = 7.60 Hz, 4H, H_{2b}), 1.49 (dq, $^2J_{H-H}$ = 14.9 Hz, $^3J_{H-H}$ = 7.60 Hz, 4H, H_{2a}), 0.86 (m[pquint], $|^2J_{P-P}| \approx$ 130 Hz, $^3J_{H-P}$ = 14.0 Hz, $^3J_{H-H}$ = 7.60 Hz, 12H, H_1); **^{1}H{^{31}P} NMR** (C_6D_6, 600 MHz, 295 K): δ [ppm] = 8.50 (d, $^4J_{H-H}$ = 1.90 Hz, 2H, H_7), 7.41 (d, $^4J_{H-H}$ = 1.90 Hz, 2H, H_5), 3.22 (s, 4H, H_3), 1.62 (s, 18H, H_{11}), 1.59 (dq, $^2J_{H-H}$ = 14.9 Hz, $^3J_{H-H}$ = 7.60 Hz, 4H, H_{2b}), 1.49 (dq, $^2J_{H-H}$ = 14.9 Hz, $^3J_{H-H}$ = 7.60 Hz, 4H, H_{2a}), 0.86 (t, $^3J_{H-H}$ = 7.60 Hz, 12H, H_1); **^{13}C{^{1}H} NMR** (C_6D_6, 151 MHz, 295K): δ [ppm] = 145.0 (m[pt], $|^2J_{P-P}| \approx$ 130 Hz, $^3J_{C-P} \approx$ 5 Hz, 2C, C_9), 137.0 (s, 2C, C_6), 125.5 (s, 2C, C_8), 123.7 (m[pt], $|^2J_{P-P}| \approx$ 130 Hz, $^3J_{C-P} \approx$ 7 Hz, 2C, C_5), 117.8 (s, 2C, C_4), 115.3 (s, 2C, C_7), 34.4 (s, 2C, C_{10}), 32.4 (s, 6C, C_{11}), 28.2 (m[pt], $|^2J_{P-P}| \approx$ 130 Hz, $^1J_{C-P} \approx$ 19 Hz, 2C, C_3), 20.2 (m[pt], $|^2J_{P-P}| \approx$ 130 Hz, $^2J_{C-P} \approx$ 24 Hz, 4C, C_2), 7.8 (s, 4C, C_1); **^{31}P NMR** (C_6D_6, 243 MHz, 295K): δ [ppm] = 1.0 (m[ps], 2P); **IR** [cm^{-1}]: ṽ = 2957, 2903, 2875, 1566, 1475, 1461, 1426, 1391, 1377, 1360, 1315, 1282, 1230, 1191, 1140, 1102, 1026, 1006, 991, 981, 952, 936, 863, 853, 826, 789, 756, 746, 713, 682, 639, 610, 575, 563, 530, 511, 497, 447, 408; **UV-VIS** [nm] (in THF): 458 (ε = 2218 $M^{-1}cm^{-1}$), 399 (ε = 14534 $M^{-1}cm^{-1}$), 380 (ε = 15135 $M^{-1}cm^{-1}$), 360 (ε = 14673 $M^{-1}cm^{-1}$), 292 (ε = 53067 $M^{-1}cm^{-1}$); **MS** (LIFDI, thf): calc. for $[C_{60}H_{92}N_2P_4Pd]^+$: 1176.43, found: 1178.53. The expected isotopic pattern agrees well with the measurement. **EA**: Anal. calc. for $C_{60}H_{92}N_2P_4Pd_2$ (1178.15 g/mol): C 61.17, H 7.87, N 2.38. Found: C 61.37, H 7.84, N 2.56.

Synthesis of complex [(^{Et}PNP)Ni]$_2$ (4^{Et}-Ni)

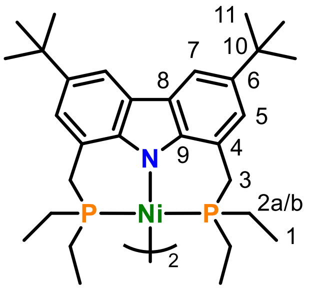

2^{Et}-Ni (1.00 g, 1.73 mmol, 1.0 eq.) was dissolved in THF (40 mL) and added to sodium powder (47.8 mg, 2.08 mmol, 1.2 eq). After addition of a solution of naphthalene (5.56 mg, 43.34 µmol, 0.025 eq) in THF (10 mL) to the mixture, the reaction was stirred at room temperature for 30 h. The solvent was removed under reduced pressure after which the residues were dissolved in toluene (30 mL) and filtered through a pad of Celite®. This Celite® pad was rinsed with additional toluene (20-40 mL) and the solvent of the collected filtrate removed *in vacuo*. The crude product was washed with a 1:5 Et_2O/ *n*-pentane mixture (5 mL) followed by *n*-pentane until washings became colorless. Subsequent removal of the solvent under reduced pressure afforded the product as a grey-greenish powder (756 mg, 698 µmol, 81%). Single crystals suitable for X-ray diffraction were grown at room temperature by slow evaporation of Et_2O or toluene/*n*-hexane solutions. **^{1}H NMR** (C_6D_6, 600 MHz, 295 K, deconvoluted): δ [ppm] = 8.37 ($\Delta\nu_{1/2}$ = 6.3 Hz, 4H, H_7), 7.38 ($\Delta\nu_{1/2}$ = 6.6 Hz, 4H, H_5), 3.35 ($\Delta\nu_{1/2}$ = 37 Hz, 8H, H_3), 1.91 ($\Delta\nu_{1/2}$ = 138 Hz, 8H, H_{2b}), 1.70 ($\Delta\nu_{1/2}$ = 62 Hz, 8H, H_{2a}), 1.58 ($\Delta\nu_{1/2}$ = 2.0 Hz, 36H, H_{11}), 0.86 ($\Delta\nu_{1/2}$ = 36 Hz, 24H, H_1); **^{13}C{^{1}H} NMR** (C_6D_6, 151 MHz, 295K): δ [ppm] = 145.5 (s, 4C, C_9), 137.4 (s, 4C, C_6), 126.1 (s, 4C, C_8), 123.3 (s, 4C, C_5), 117.9 (s, 4C, C_4), 114.9 (s, 4C, C_7), 34.3 (s, 4C, C_{10}), 32.3 (s, 12C, C_{11}), 26.4 (s, 4C, C_3), 20.0 (s, 8C, C_2), 8.37 (s, 8C, C_1); **^{31}P NMR** (C_6D_6, 243 MHz, 295K): δ [ppm] = -5.9 (s, 4P); **IR** [cm^{-1}]: ṽ = 2952, 2904, 2874, 1569, 1461, 1424, 1394, 1360, 1316, 1278, 1223, 1185, 1137, 1103, 1029, 988, 954, 865, 850, 820, 790, 775, 749, 712, 675, 656; **UV-VIS** [nm] (in THF): 697 (ε = 203 $M^{-1}cm^{-1}$), 557 (ε = 306 $M^{-1}cm^{-1}$), 420 (ε = 6764 $M^{-1}cm^{-1}$), 349 (ε = 29906 $M^{-1}cm^{-1}$), 290 (ε = 42201 $M^{-1}cm^{-1}$); **MS** (LIFDI, thf): calc. for $[C_{60}H_{92}N_2Ni_2P_4]^+$: 1080.49, found: 540.28 ([(^{Et}PNP)Ni^{I}], theor. 540.25). The expected isotopic pattern agrees well with the measurement.

1.5) Metal(I) (heterodinuclear) Synthesis and Characterization Data

Synthesis of complex [(^{Et}PNP)Pd-Ni(^{Et}PNP)] (7^{Et}-NiPd)

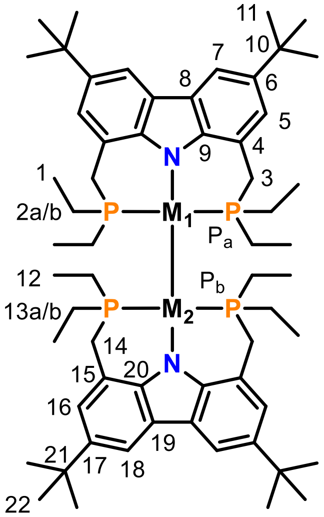

4^{Et}-Ni (10.0 mg, 9.24 µmol, 1.0 eq.) and **4^{Et}-Pd** (10.9 mg, 9.24 µmol, 1.0 eq.) were dissolved in C_6D_6 (0.5 mL). The solution was irradiated with light (λ = 390 nm) for 2 h. Removal of the solvent under reduced pressure afforded the product as a brown powder in quantitative yields. Single crystals suitable for X-ray diffraction were grown from a 1:3 benzene/*n*-pentane solution at -40°C. **^{1}H NMR** (C_6D_6, 600 MHz, 295 K): δ [ppm] = 8.47 (d, $^4J_{H\text{-}H}$ = 2.00 Hz, 2H, H_7), 8.40 (d, $^4J_{H\text{-}H}$ = 2.00 Hz, 2H, H_{18}), 7.39 (m, resonance 1: 7.391 ppm, d, $^4J_{H\text{-}H}$ = 2.00 Hz, 2H, H_{16}; resonance2: 7.394 ppm, d, $^4J_{H\text{-}H}$ = 2.00 Hz, 2H, H_5), 3.35 (s, 4H, H_{14}), 3.22 (m[pt], $|^2J_{P\text{-}P}|$ ≈ 230 Hz, $^2J_{H\text{-}P}$ = 5.30 Hz, 4H, H_3), 1.83-1.72 (m, resoncance 1: 1.793 ppm, m, $|^2J_{P\text{-}P}|$ ≈ 230 Hz, $^2J_{H\text{-}H}$ = 15.0 Hz, $^3J_{H\text{-}H}$ = 7.60 Hz, $^2J_{H\text{-}P}$ = 5.00 Hz, 4H, H_{2b}; resonance 2: 1.750 ppm, m, $^2J_{H\text{-}H}$ = 15.0 Hz, $^3J_{H\text{-}H}$ = 7.60 Hz, $J_{H\text{-}P}$ not resolved, 4H, H_{2a}), 1.61 (s. 18H, H_{11}), 1.59 (s, 18H, H_{22}), 1.56 (m, $^2J_{H\text{-}H}$ = 14.3 Hz, $^3J_{H\text{-}H}$ = 7.60 Hz, $^2J_{H\text{-}P}$ not resolved, 4H, H_{13b}), 1.49 (m, $^2J_{H\text{-}H}$ = 14.3 Hz, $^3J_{H\text{-}H}$ = 7.60 Hz, $^2J_{H\text{-}P}$ not resolved, 4H, H_{13a}), 0.99 (m[pquint], $|^2J_{P\text{-}P}|$ ≈ 230 Hz, $^3J_{H\text{-}P}$ = 14.0 Hz, $^3J_{H\text{-}H2b}$ = 7.60 Hz, $^3J_{H\text{-}H2a}$ = 7.60 Hz, 12H, H_1), 0.76 (m[pquint], $|^2J_{P\text{-}P}|$ ≈ 230 Hz, $^3J_{H\text{-}P}$ = 14.0 Hz, $^3J_{H\text{-}H13b}$ = 7.60 Hz, $^3J_{H\text{-}H13a}$ = 7.60 Hz, 12H, H_{12}); **^{1}H{^{31}P} NMR** (C_6D_6, 600 MHz, 295 K): δ [ppm] = 8.47 (d, $^4J_{H\text{-}H}$ = 2.00 Hz, 2H, H_7), 8.40 (d, $^4J_{H\text{-}H}$ = 2.00 Hz, 2H, H_{18}), 7.39 (m, resonance 1: 7.391 ppm, d, $^4J_{H\text{-}H}$ = 2.00 Hz, 2H, H_{16}; resonance2: 7.394 ppm, d, $^4J_{H\text{-}H}$ = 2.00 Hz, 2H, H_5), 3.35 (s, 4H, H_{14}), 3.22 (s, 4H, H_3), 1.83-1.72 (m, resoncance 1: 1.793 ppm, dq, $^2J_{H\text{-}H}$ = 15.0 Hz, $^3J_{H\text{-}H}$ = 7.60 Hz, 4H, H_{2b}; resonance 2: 1.750 ppm, dq, $^2J_{H\text{-}H}$ = 15.0 Hz, $^3J_{H\text{-}H}$ = 7.60 Hz, 4H, H_{2a}), 1.61 (s. 18H, H_{11}), 1.59 (s, 18H, H_{22}), 1.56 (m[dq], $^2J_{H\text{-}H}$ = 14.3 Hz, $^3J_{H\text{-}H}$ = 7.60 Hz, 4H, H_{13b}), 1.49 (m[dq], $^2J_{H\text{-}H}$ = 14.3 Hz, $^3J_{H\text{-}H}$ = 7.60 Hz, 4H, H_{13a}), 0.99 (t, $^3J_{H\text{-}H2a}$ = 7.60 Hz, $^3J_{H\text{-}H2b}$ = 7.60 Hz, 12H, H_1), 0.76 (t, $^3J_{H\text{-}H13b}$ = 7.60 Hz, $^3J_{H\text{-}H13a}$ = 7.60 Hz, 12H, H_{12}); **^{13}C{^{1}H} NMR** (C_6D_6, 151 MHz, 295K): δ [ppm] = 145.57 (m[pt], $|^2J_{P\text{-}P}|$ ≈ 230 Hz, $^3J_{C\text{-}P}$ = 8.1 Hz, 2C, C_{20}), 145.0 (m[pt], $|^2J_{P\text{-}P}|$ ≈ 230 Hz, $^3J_{C\text{-}P}$ = 4.8 Hz, 2C, C_9), 137.52 (s, 2C, C_{17}), 136.80 (s, 2C, C_6), 126.35 (s, 2C, C_{19}), 125.11 (s, 2C, C_8), 123.63 (m[pt], $|^2J_{P\text{-}P}|$ ≈ 230 Hz, $^3J_{C\text{-}P}$ = 7.65 Hz, 2C, C_5), 123.19 (m[pt], $|^2J_{P\text{-}P}|$ ≈ 230 Hz, $^3J_{C\text{-}P}$ = 7.57 Hz, 2C, C_{16}), 118.08 (s, 2C, C_{15}), 117.75 (s, 2C, C_4), 115.20 (s, 2C, C_7), 114.93 (s, 2C, C_{18}) 34.36 (s, 2C, C_{10}), 34.33 (s, 2C, C_{21}), 32.36 (s, 6C, C_{11}), 32.30 (s, 6C, C_{22}), 27.97 (m[pt], $|^2J_{P\text{-}P}|$ ≈ 230 Hz, $^1J_{C\text{-}P}$ = 21.8 Hz, 2C, C_3), 26.69 (m[pt], $|^2J_{P\text{-}P}|$ ≈ 230 Hz, $^1J_{C\text{-}P}$ = 17 Hz, 2C, C_{14}), 20.91 (bs, 4C, C_{13}), 19.64 (m[pt], $|^2J_{P\text{-}P}|$ ≈ 230 Hz, $^1J_{C\text{-}P}$ = 23 Hz, 4C, C_2), 8.17 (s, 4C, C_1), 8.00 (s, 4C, C_{12}); **^{31}P{^{1}H} NMR** (C_6D_6, 243 MHz, 295K): δ [ppm] = -3.4 (t, $^3J_{P\text{-}P}$ = 10.5 Hz, $2P_b$), -3.4 (ps, $\Delta v_{1/2}$ = 27 Hz, $^3J_{P\text{-}P}$ = 10.5 Hz, $2P_a$); **IR** [cm^{-1}]: ṽ = 2952, 2900, 2874, 1569, 1461, 1424, 1394, 1360, 1316, 1282, 1226, 1189, 1137, 1100, 1029, 950, 936, 865, 824, 790, 775, 749, 712, 678, 656; **UV-VIS** [nm] (in thf): 659 (ε = 172 $M^{-1}cm^{-1}$), 548 (ε = 630 $M^{-1}cm^{-1}$), 474 (ε = 1275 $M^{-1}cm^{-1}$), 401 (ε = 12089 $M^{-1}cm^{-1}$), 349 (ε = 21626 $M^{-1}cm^{-1}$), 294 (ε = 46822 $M^{-1}cm^{-1}$); **MS** (LIFDI, thf): calc. for $[C_{60}H_{92}N_2NiP_4Pd]^+$: 1128.46, found: 1128.53. The expected isotopic pattern agrees well with the measurement. **EA**: Anal. calc. for $C_{60}H_{92}N_2NiP_4Pd$ (1130.42 g/mol): C 63.75, H 8.20, N 2.48. Found: C 65.90, H 8.35, N 2.18.

2) NMR spectra

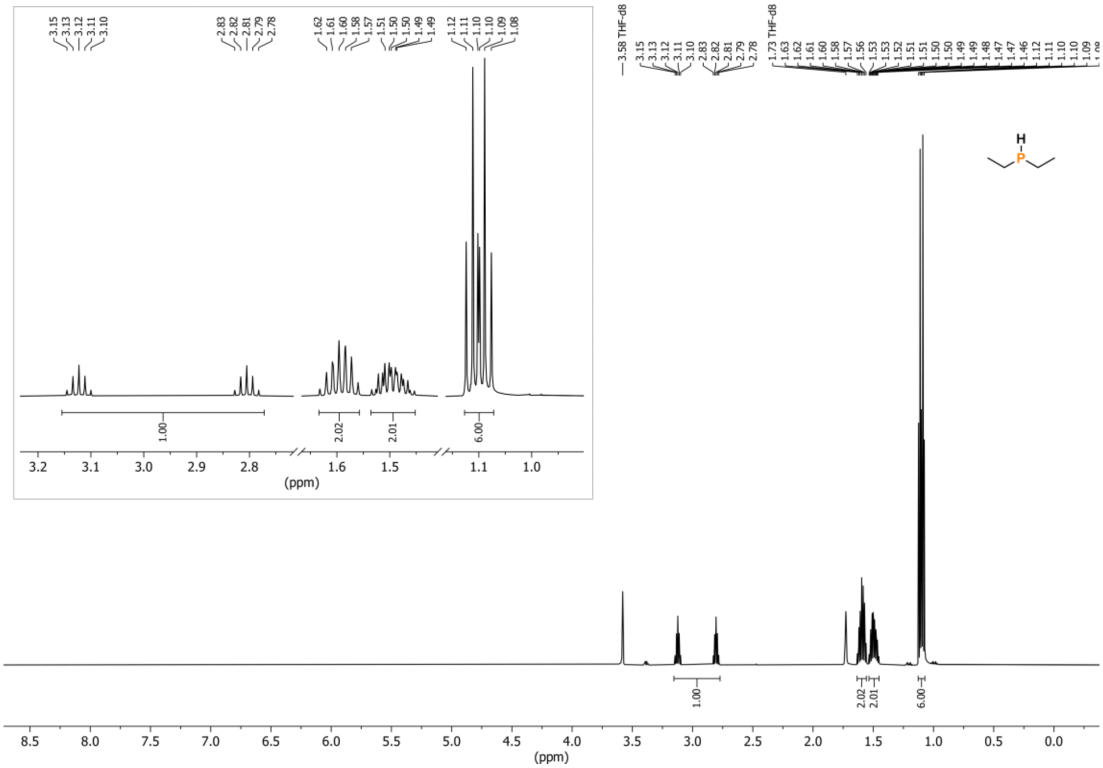

Figure S1. ^1H NMR spectrum (thf-d_8, 600 MHz, 295 K) of **HPEt₂**.

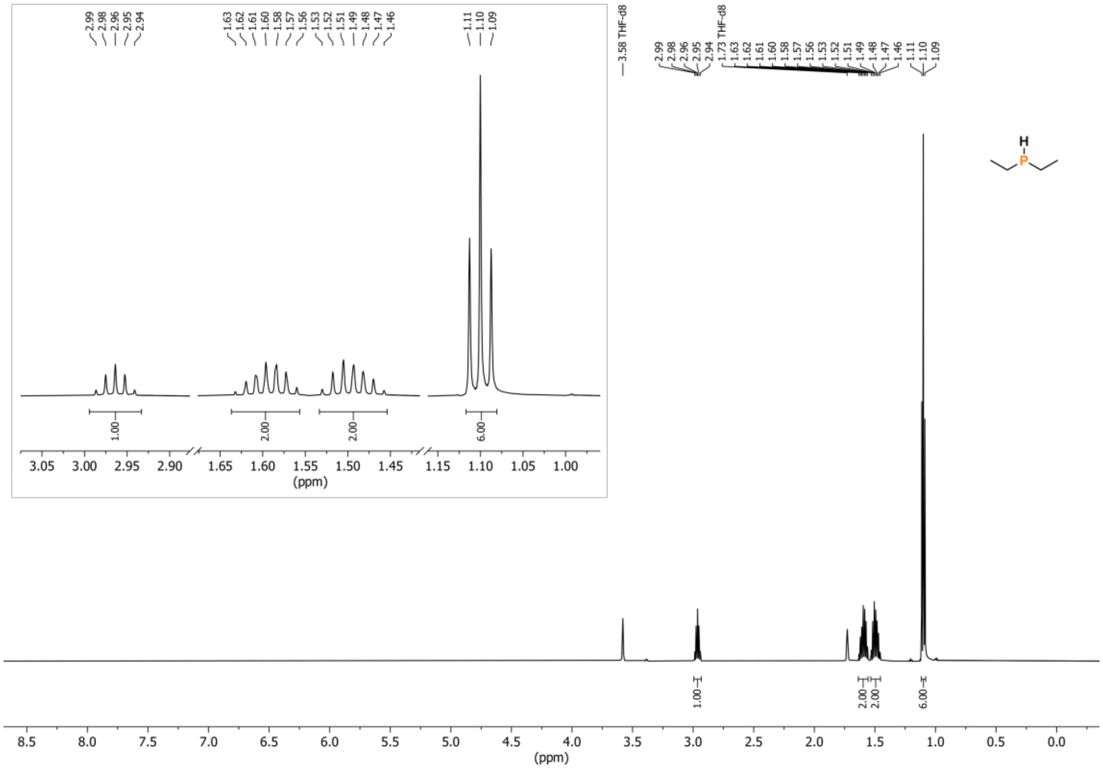

Figure S2. $^1H\{^{31}P\}$ NMR spectrum (thf-d_8, 600 MHz, 295 K) of **HPEt₂**.

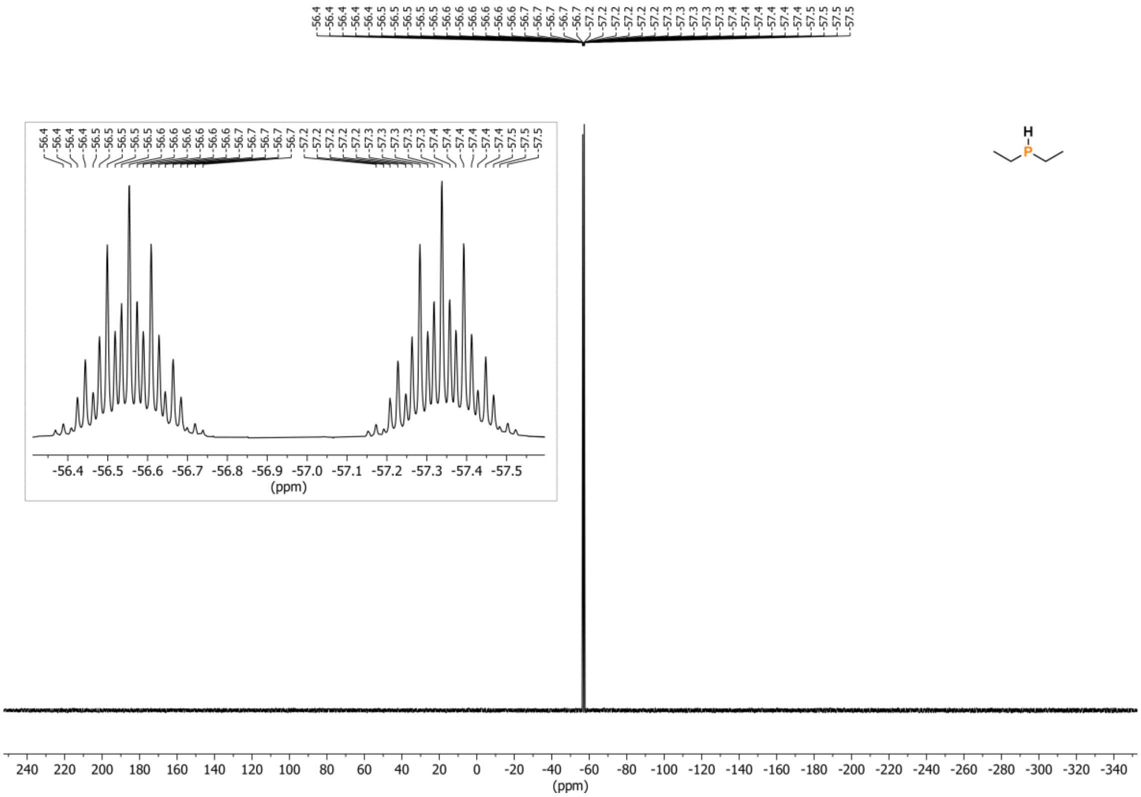

Figure S3. ^{31}P NMR spectrum (thf-d_8, 243 MHz, 295K) of **HPEt₂**.

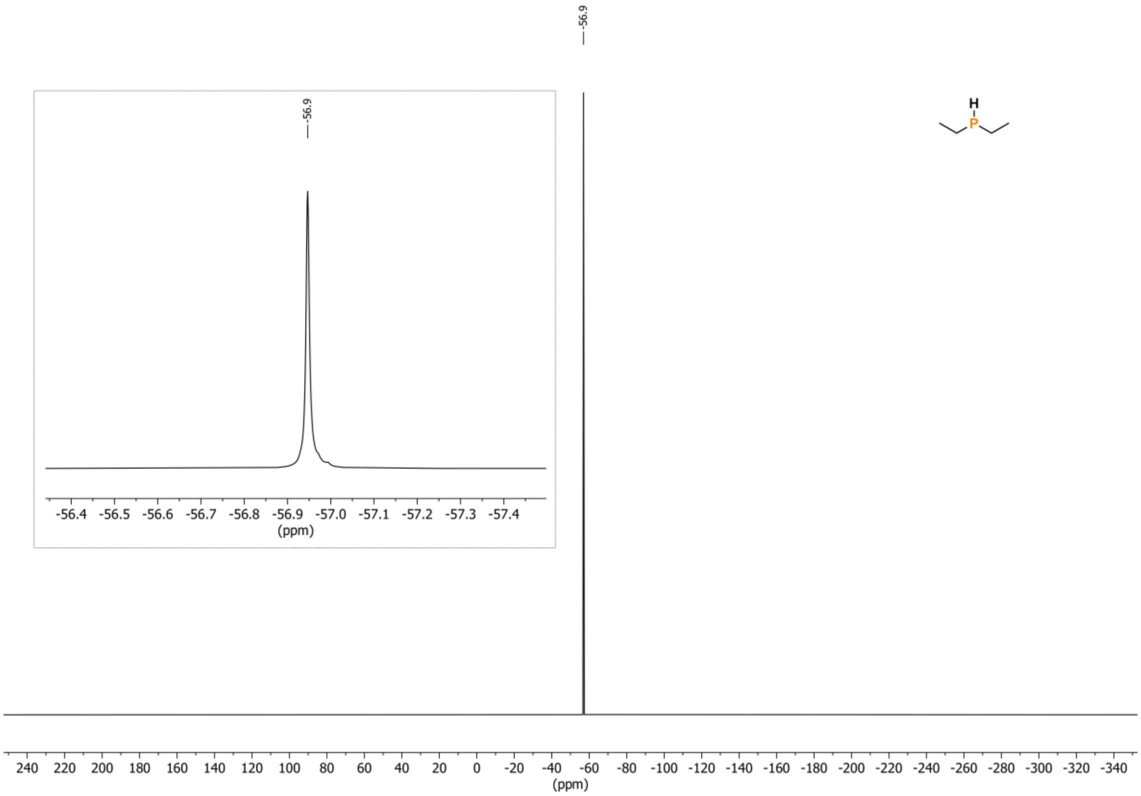

Figure S4. $^{31}P\{^1H\}$ NMR spectrum (thf-d_8, 243 MHz, 295K) of **HPEt$_2$**.

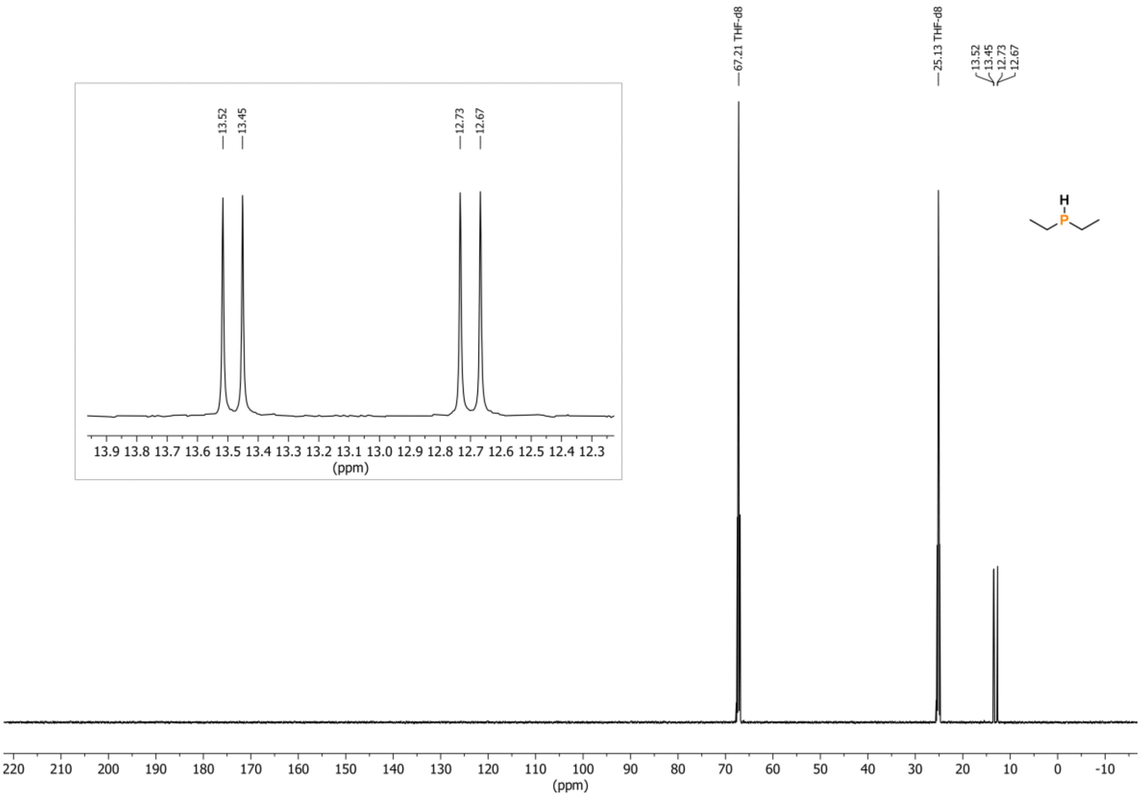

Figure S5. $^{13}C\{^{1}H\}$ NMR spectrum (thf-d_8, 151 MHz, 295 K) of **$HPEt_2$**.

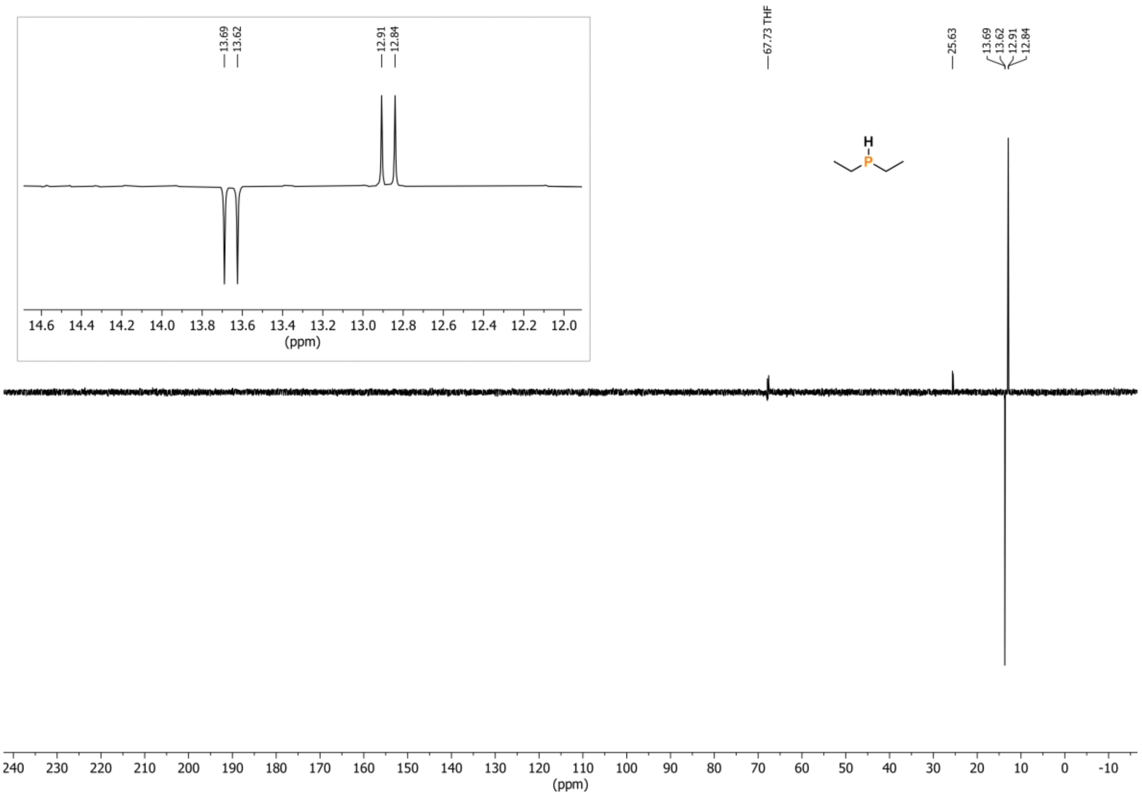

Figure S6. $^{13}C\{^{1}H\}$ DEPT-135 NMR spectrum (thf-d_8, 151 MHz, 295 K) of **$HPEt_2$**.

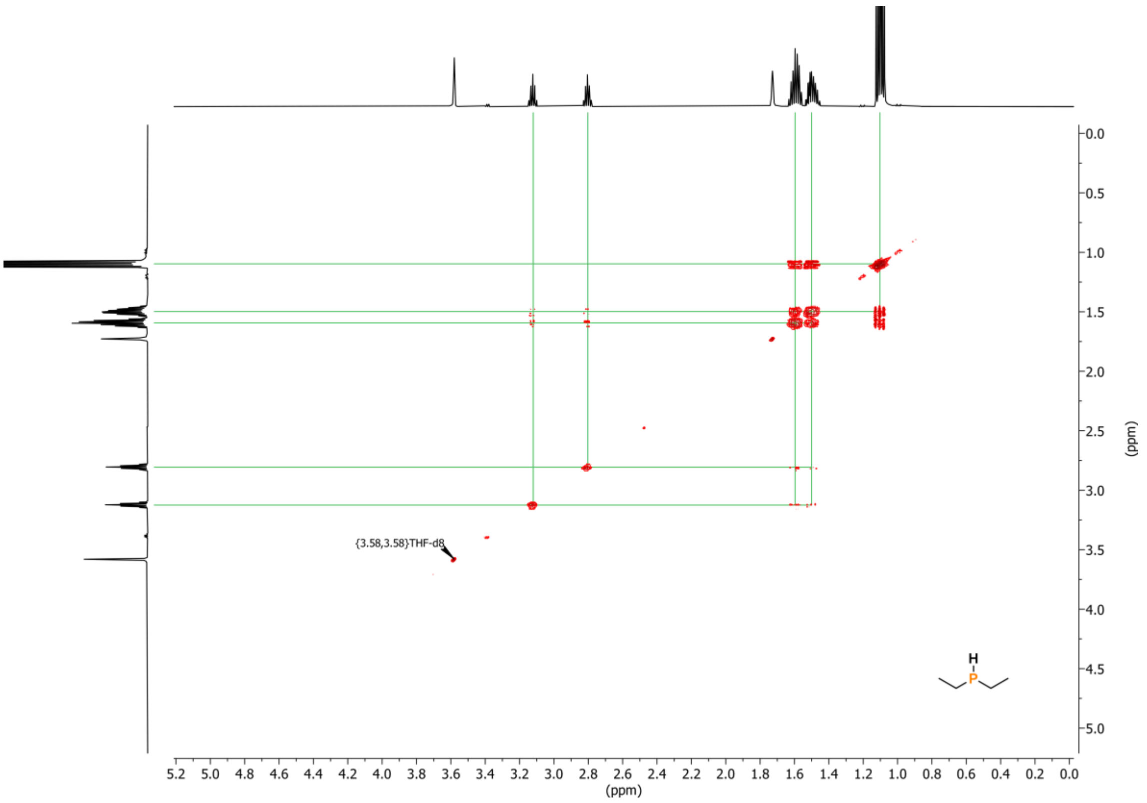

Figure S7. $^{1}H/^{1}H$ COSY NMR spectrum (thf-d_8, 600/600 MHz, 295 K) of **HPEt$_2$**.

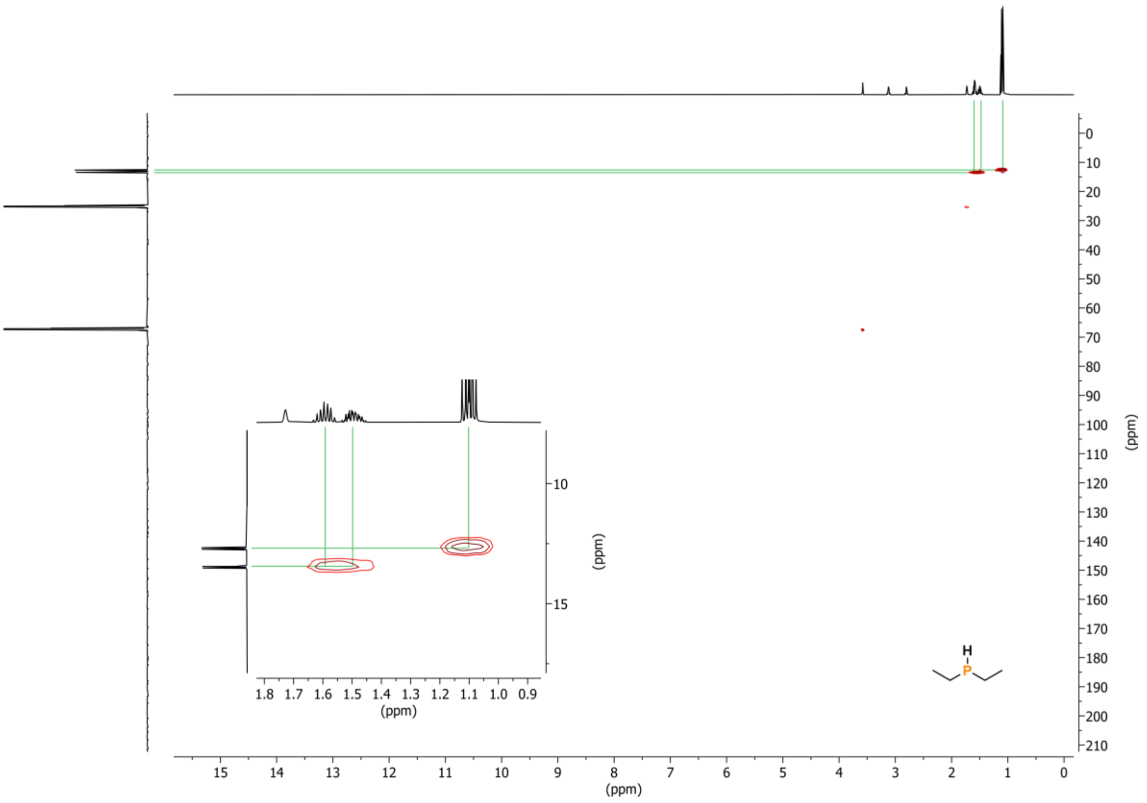

Figure S8. $^{1}H/^{13}C$ HSQC NMR spectrum (thf-d_8, 600/151 MHz, 295 K) of **HPEt$_2$**.

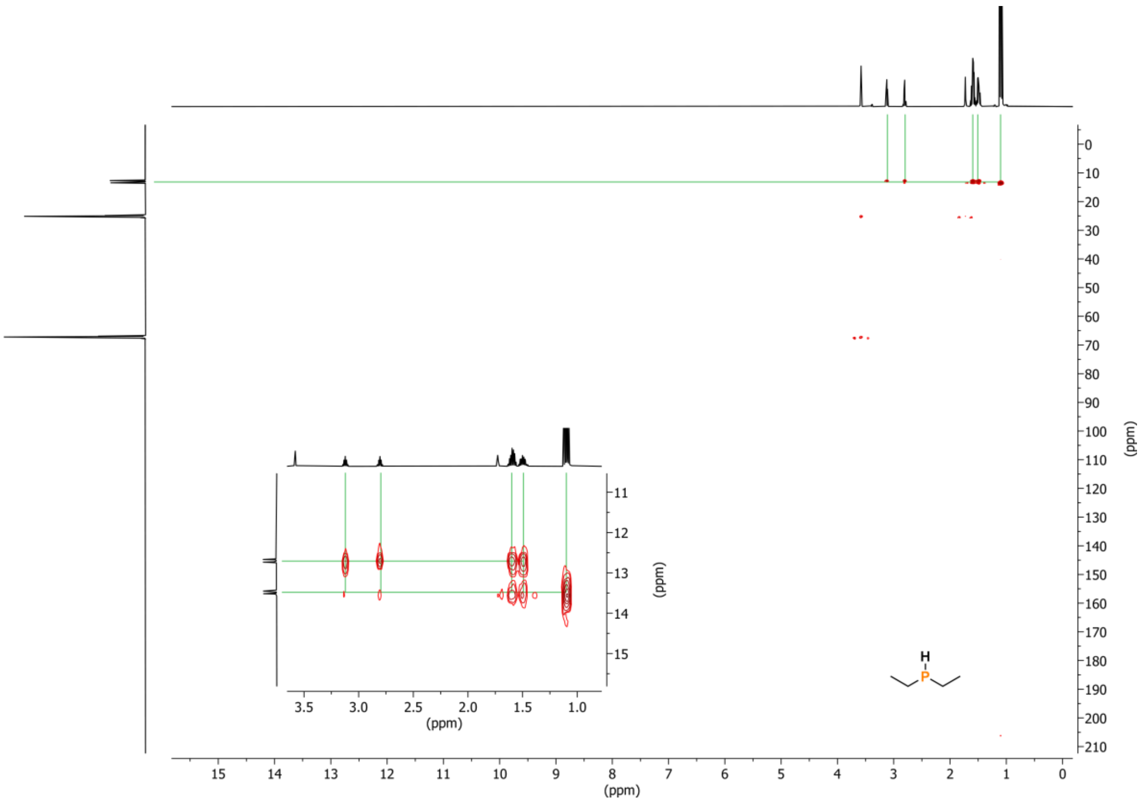

Figure S9. ${}^{1}H/{}^{13}C$ HMBC NMR spectrum (thf-d_8, 600/151 MHz, 295 K) of **$HPEt_2$**.

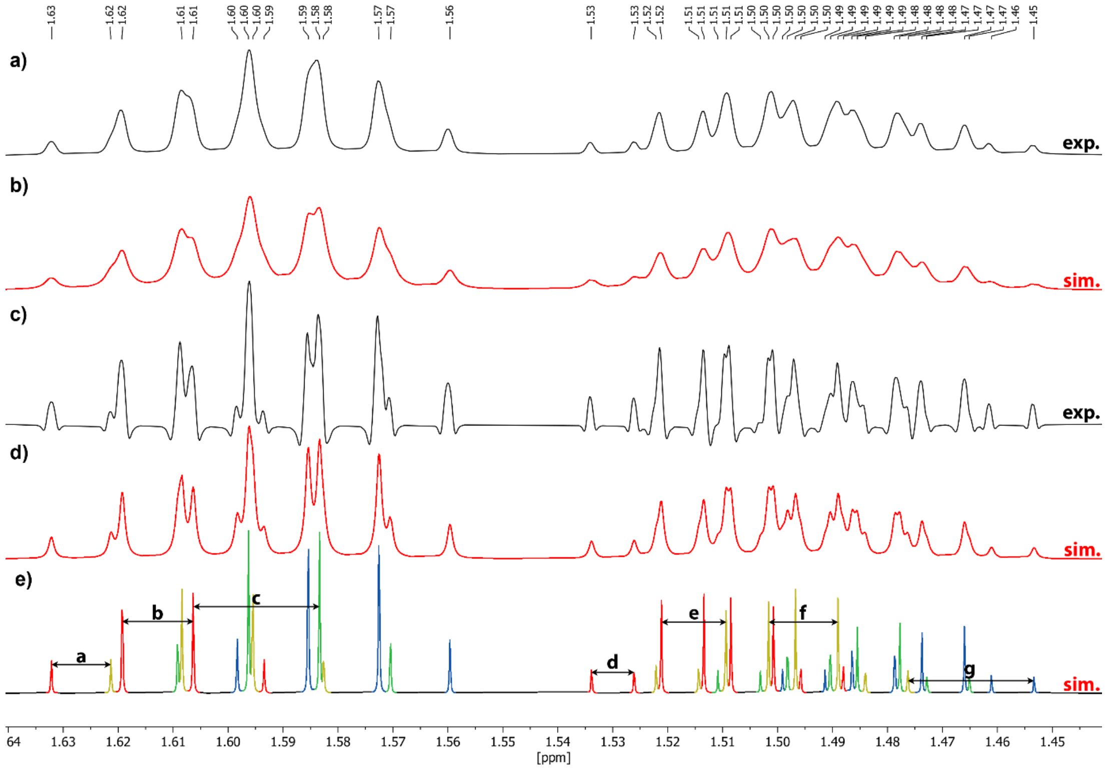

Figure S10. CH_2 region in the ${}^{1}H$ NMR spectrum (thf-d_8, 600 MHz, 295 K) of **$HPEt_2$**. a) Experimental spectrum with LB = 0.1 Hz and exponential window function; b) simulation with linewidth = 1.4 Hz; c) experimental spectrum with LB = -1.4 Hz, GB = 0.3 and gaussian window function; d) simulation with linewidth = 0.7 Hz; e) simulation with linewidth = 0.2 Hz, color-coded with a-g marking splittings.

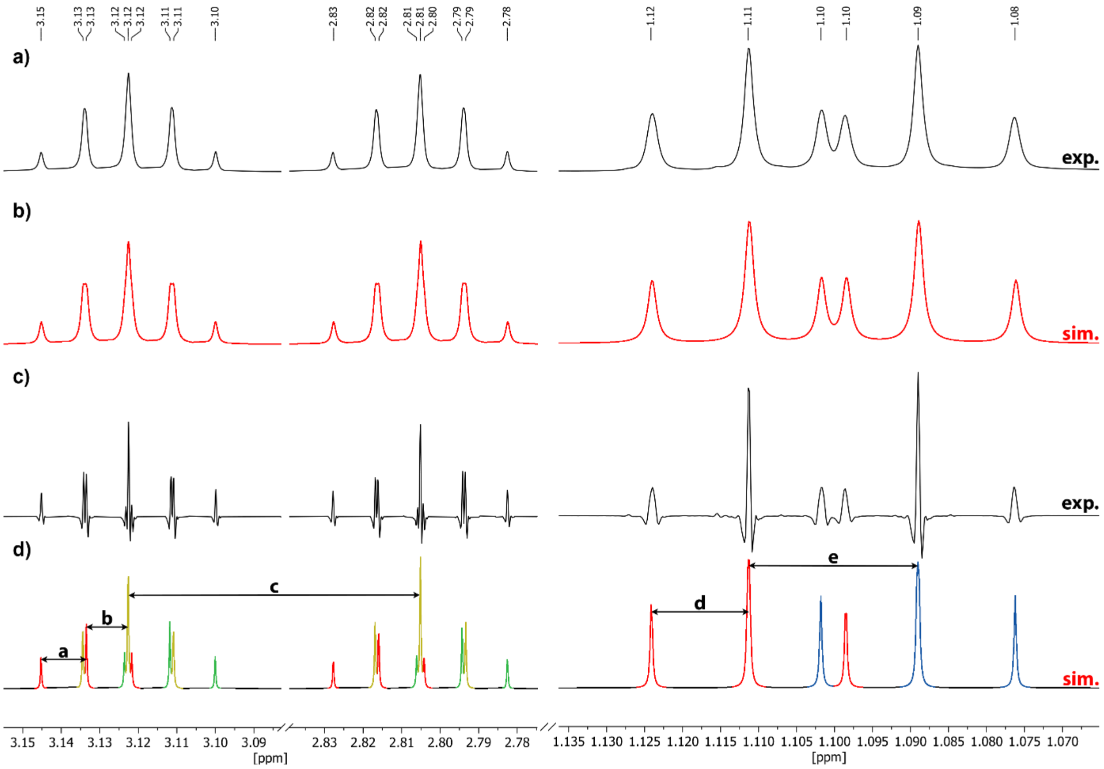

Figure S11. CH_3 region in the ^1H NMR spectrum (thf-d_8, 600 MHz, 295 K) of **HPEt$_2$**. a) Experimental spectrum with LB = 0.1 Hz; b) simulation with linewidth = 1.0 Hz; c) experimental spectrum with LB = -1.4 Hz, GB = 0.5 and gaussian window function; d) simulation with linewidth = 0.2 Hz, color-coded with a-e marking splittings.

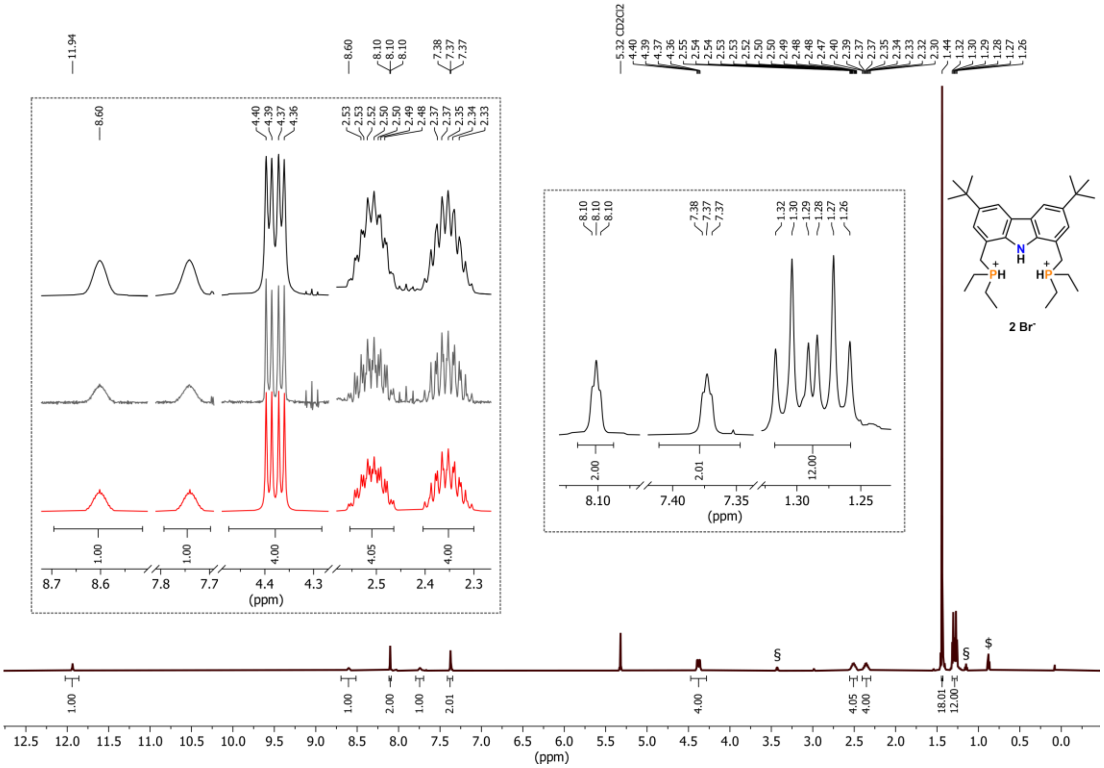

Figure S12. ^1H NMR spectrum (CD_2Cl_2, 600 MHz, 295 K) of **[(^{Et}PNP)H$_3$]Br$_2$**. The grey spectrum (left inset, middle) shows the experimental spectrum with LB = -2.7 Hz, GB = 0.1 and gaussian window function. Simulated spectrum in red. \$ = *n*-pentane, § = Et_2O.

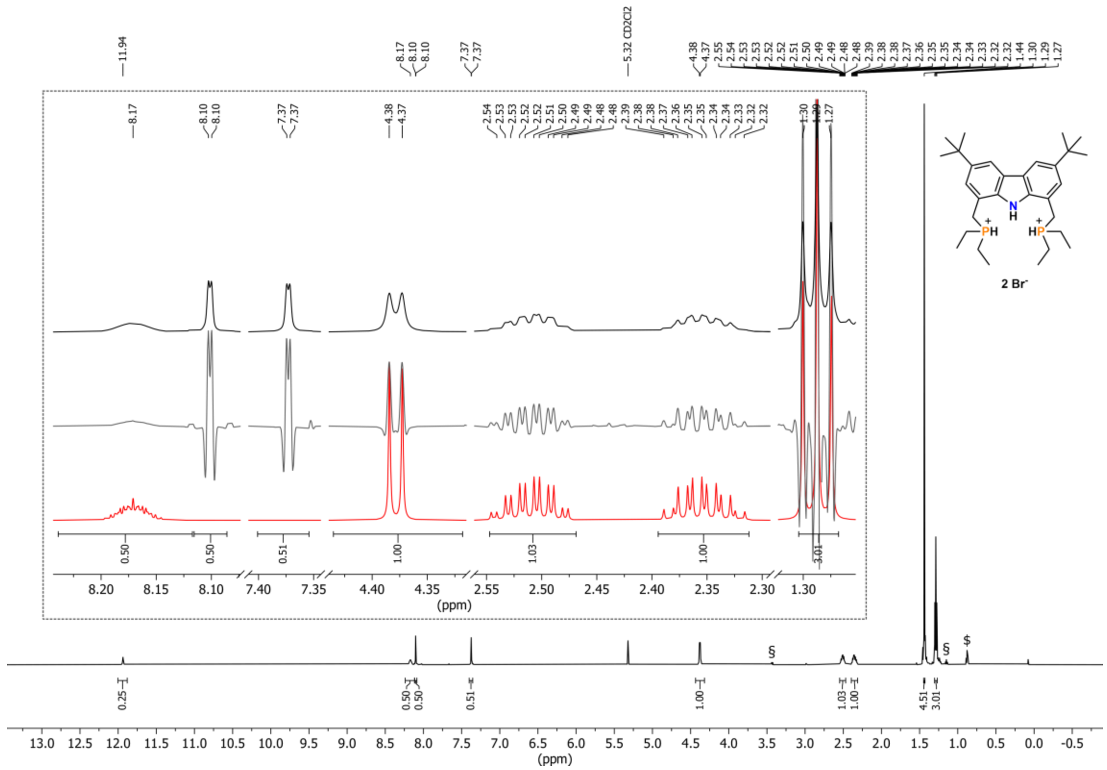

Figure S13. $^{1}H\{^{31}P\}$ NMR spectrum (CD_2Cl_2, 600 MHz, 295 K) of **[(^{Et}PNP)H$_3$]Br$_2$**. The grey spectrum (inset, middle) shows the experimental spectrum with LB = -5.3 Hz, GB = 0.2 and gaussian window function. Simulated spectrum in red. $ = *n*-pentane, § = Et_2O.

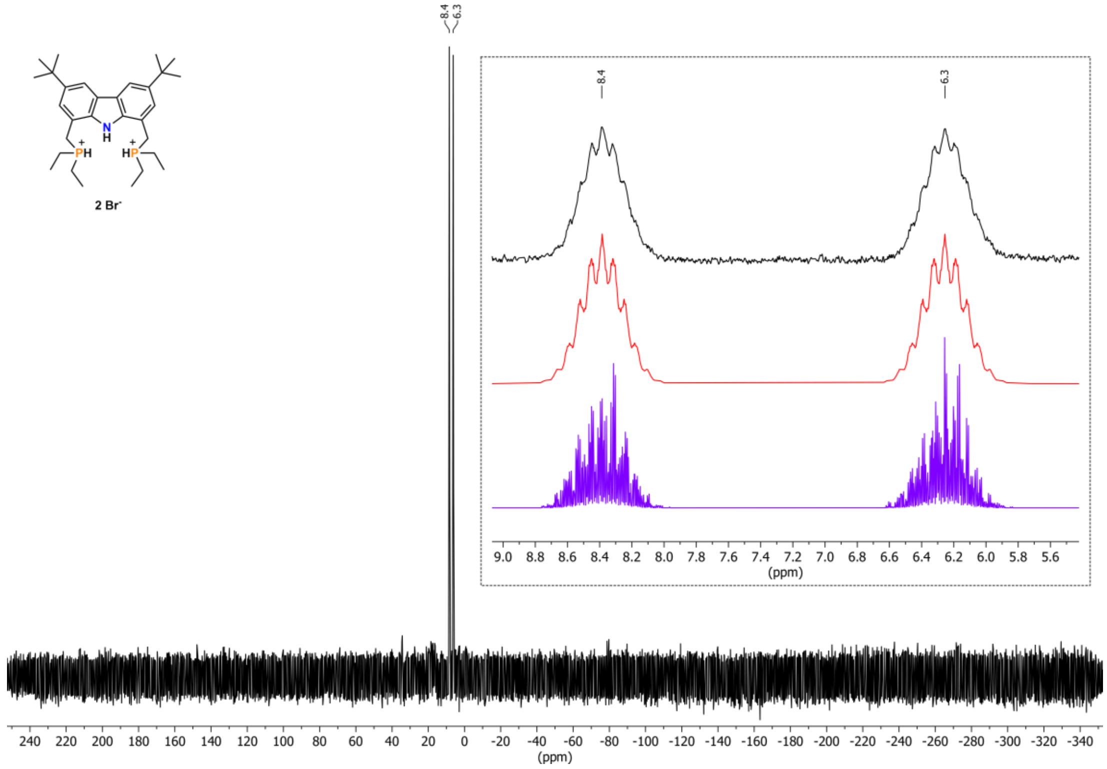

Figure S14. ^{31}P NMR spectrum (CD_2Cl_2, 243 MHz, 295K) of **[(^{Et}PNP)H$_3$]Br$_2$**. Simulated spectrum in red (linewidth = 3 Hz) and purple (linewidth = 0.1 Hz). Note: ^{31}P NMR was not included in the signal fitting procedure.

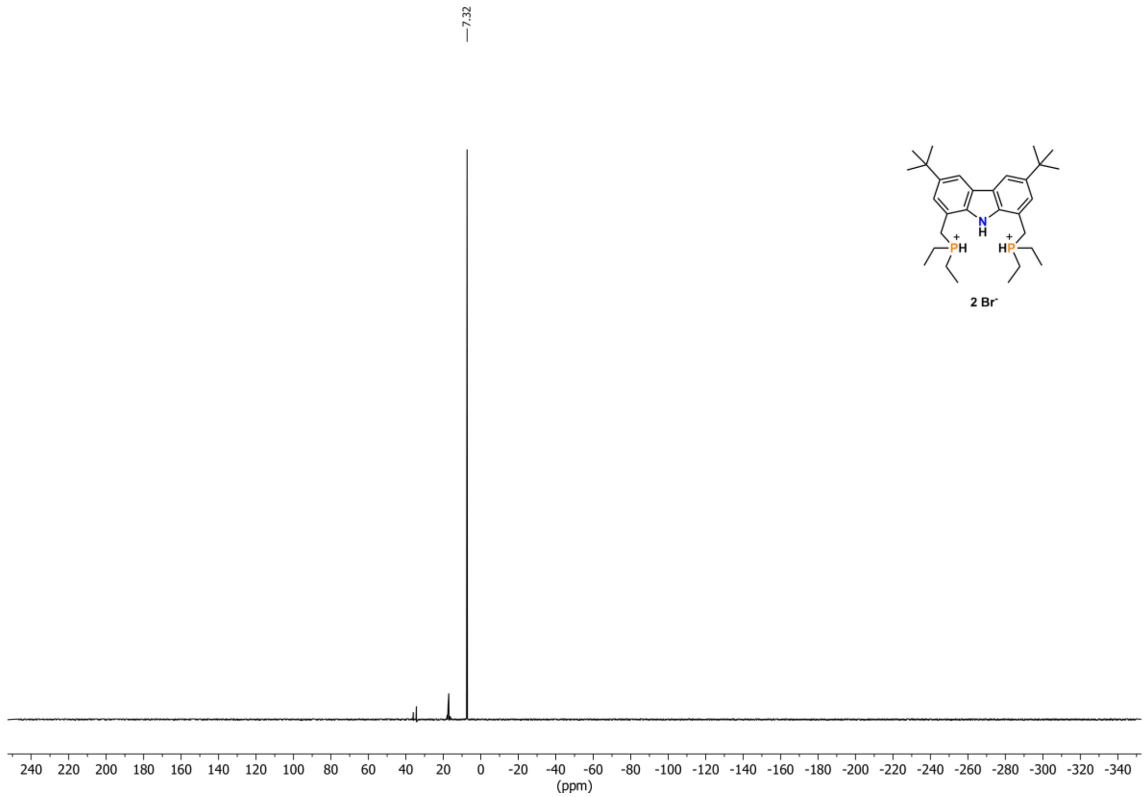

Figure S15. $^{31}P\{^{1}H\}$ NMR spectrum (CD_2Cl_2, 243 MHz, 295K) of **[(^{Et}PNP)H_3]Br_2** (minor unknown impurities).

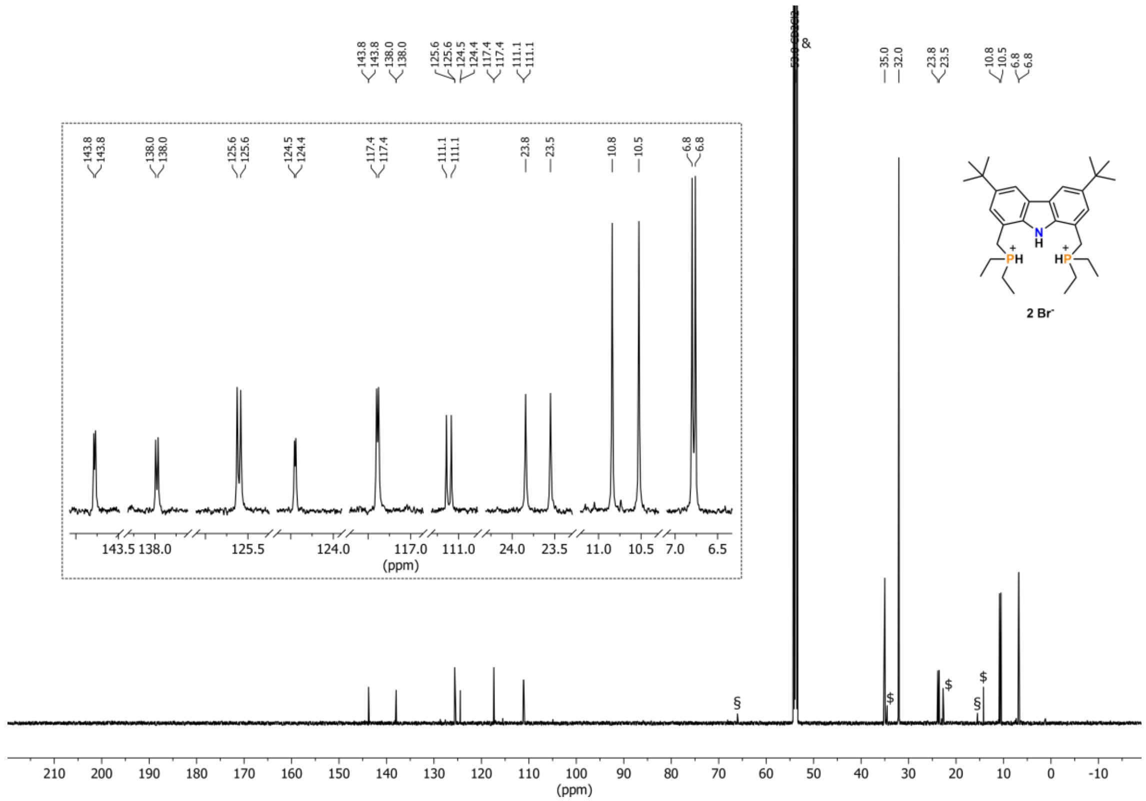

Figure S16. $^{13}C\{^{1}H\}$ NMR spectrum (CD_2Cl_2, 151 MHz, 295 K) of **[(^{Et}PNP)H_3]Br_2** ($ = *n*-pentane, § = Et_2O, & = CD_2Cl_2).

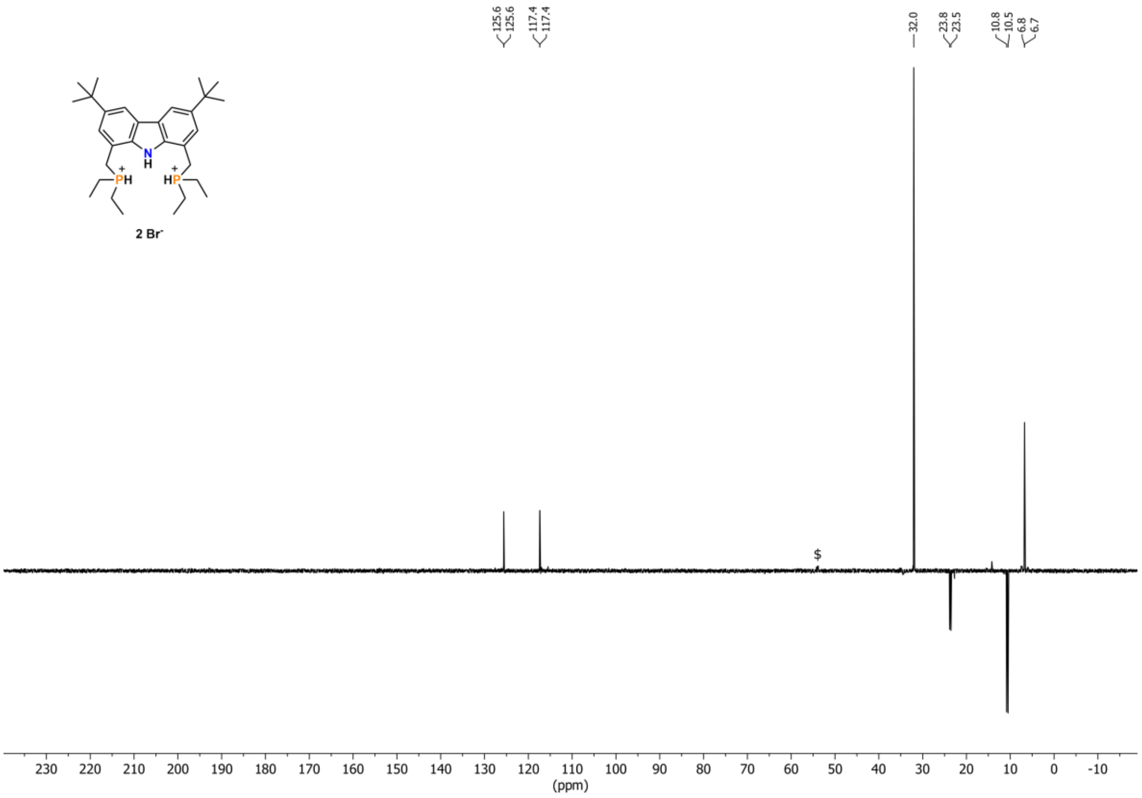

Figure S17. $^{13}C\{^{1}H\}$ DEPT-135 NMR spectrum (CD_2Cl_2, 151 MHz, 295 K) of **[(^{Et}PNP)H$_3$]Br$_2$** ($ = residual DCM).

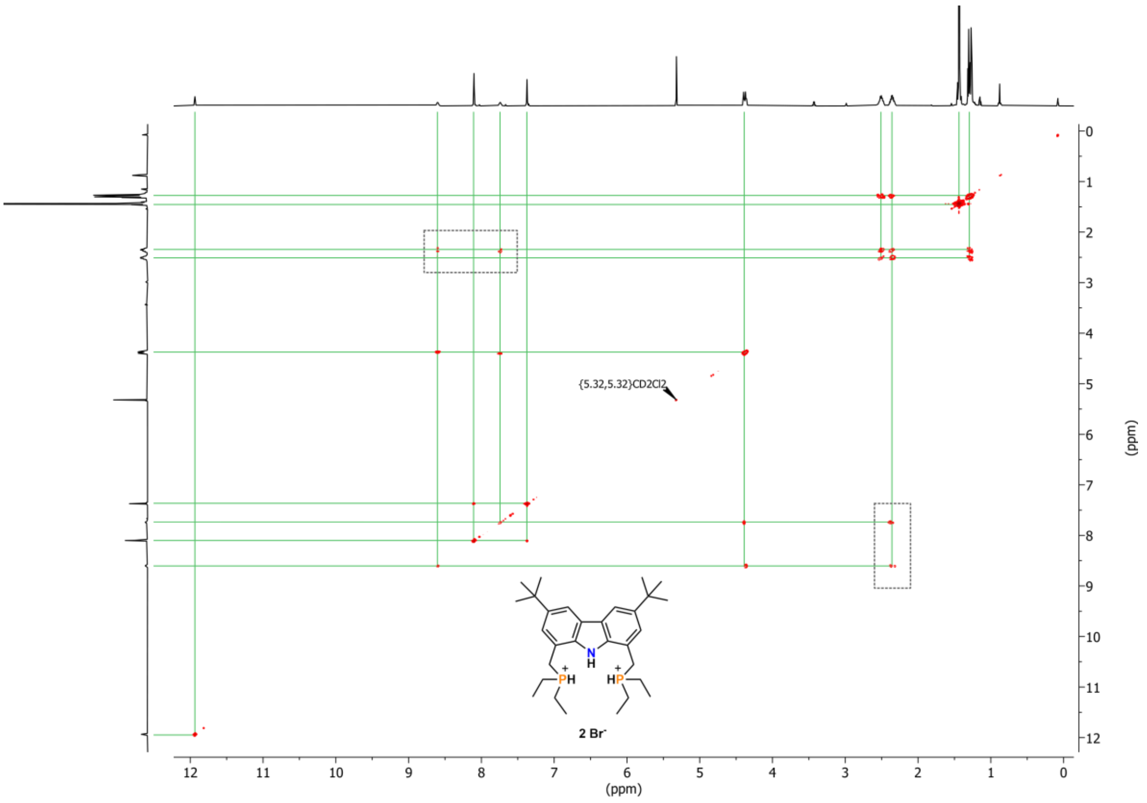

Figure S18. $^{1}H/^{1}H$ COSY NMR spectrum (CD_2Cl_2, 600/600 MHz, 295 K) of **[(^{Et}PNP)H$_3$]Br$_2$**. Due to a large dynamic range, the weaker signals were amplified in the inset.

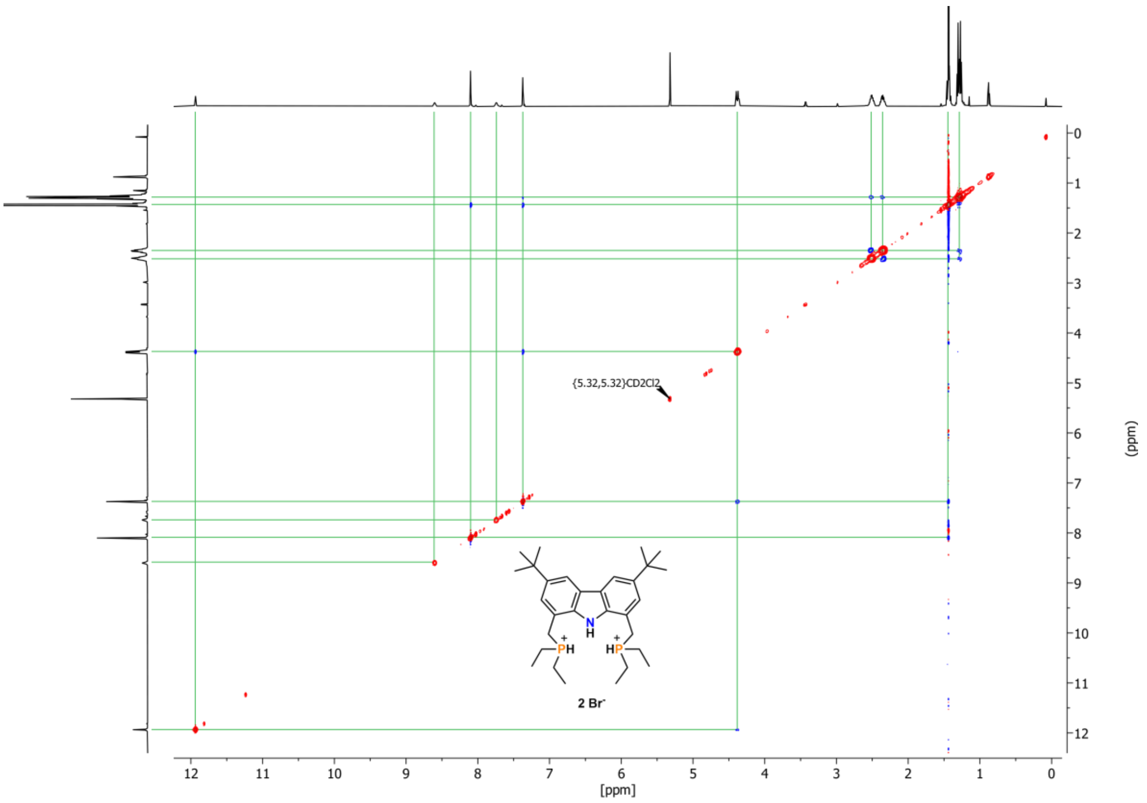

Figure S19. $^{1}H/^{1}H$ NOESY NMR spectrum (CD_2Cl_2, 600/600 MHz, 295 K) of **[(^{Et}PNP)H_3]Br_2**.

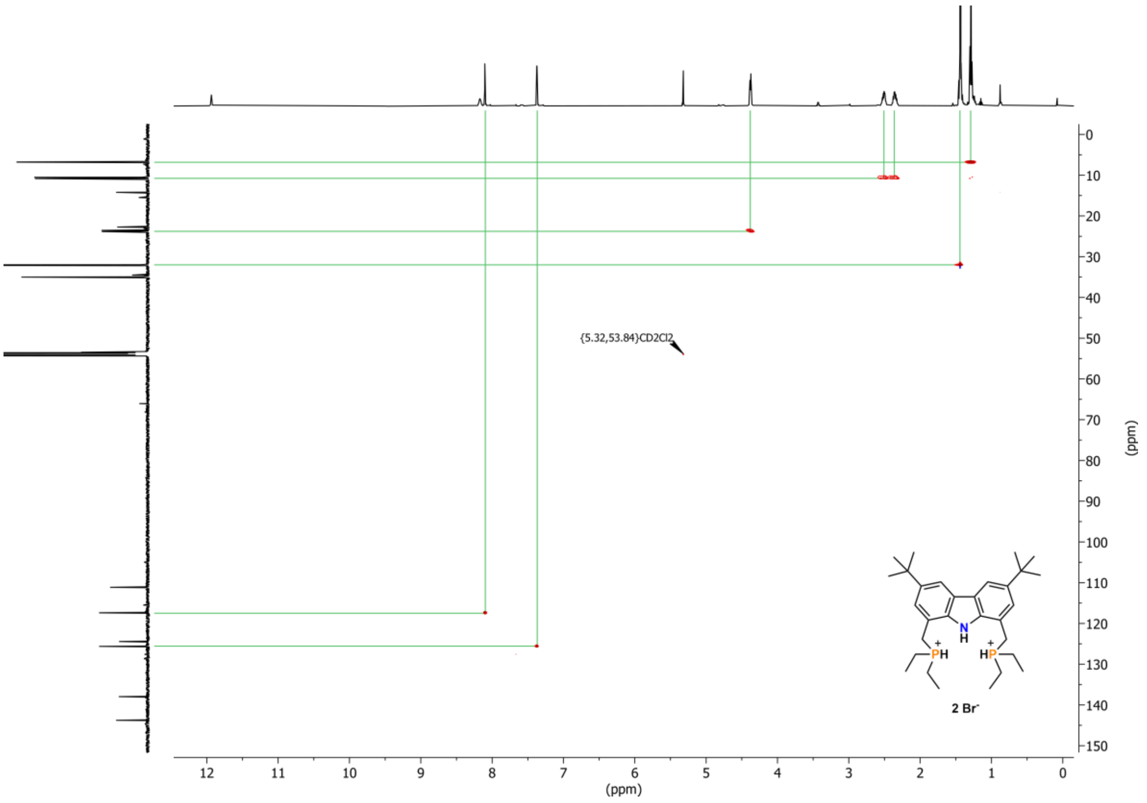

Figure S20. $^{1}H/^{13}C$ HSQC NMR spectrum (CD_2Cl_2, 600/151 MHz, 295 K) of **[(^{Et}PNP)H_3]Br_2**.

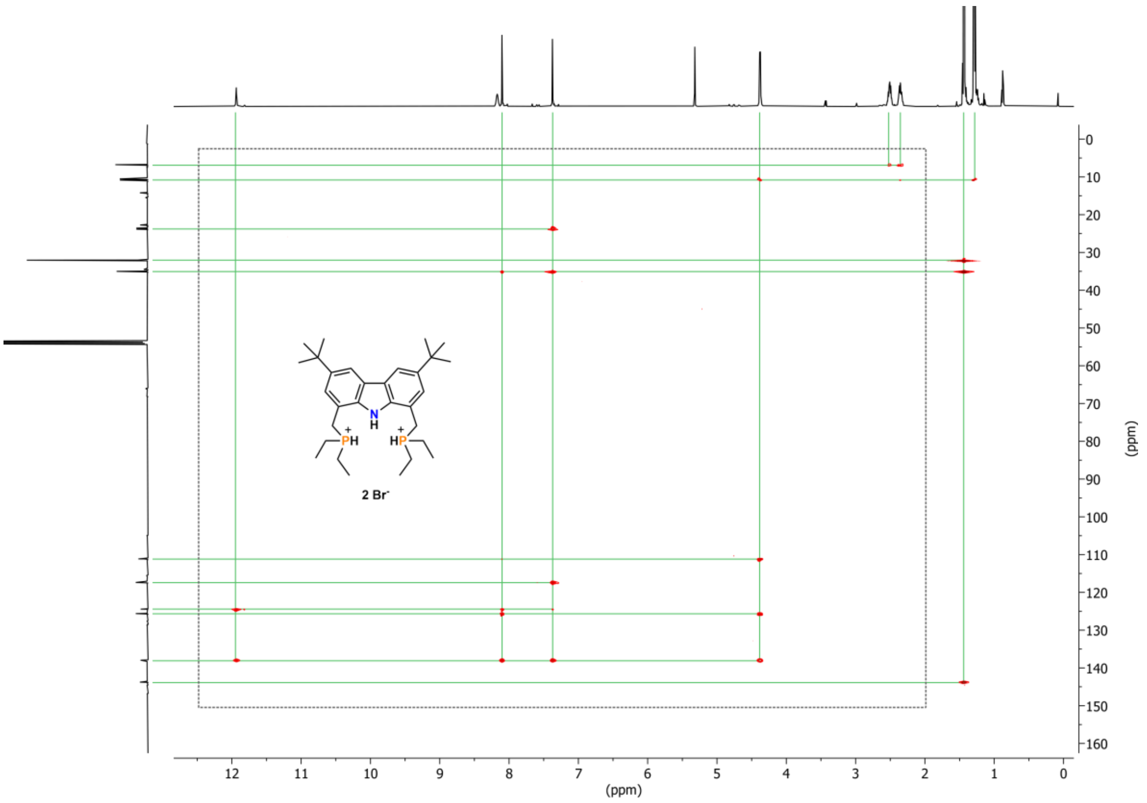

Figure S21. $^{1}H/^{13}C$ HMBC NMR spectrum (CD_2Cl_2, 600/151 MHz, 295 K) of **[(^{Et}PNP)H_3]Br_2**. Due to a large dynamic range, the weaker signals were amplified in the inset.

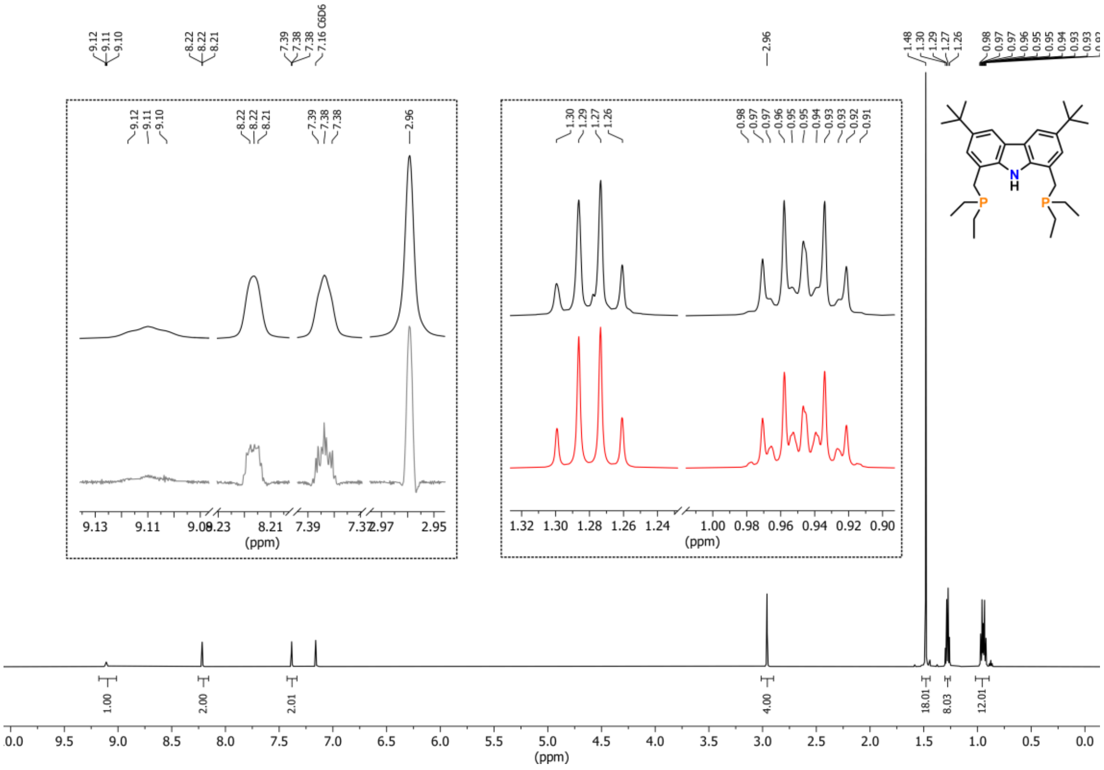

Figure S22. ^{1}H NMR spectrum (C_6D_6, 600 MHz, 295 K) of **(^{Et}PNP)H (1^{Et})**. The grey spectrum (inset left, bottom) shows the experimental spectrum with LB = -1.5 Hz, GB = 0.4 using a gaussian window function. Simulation in red.

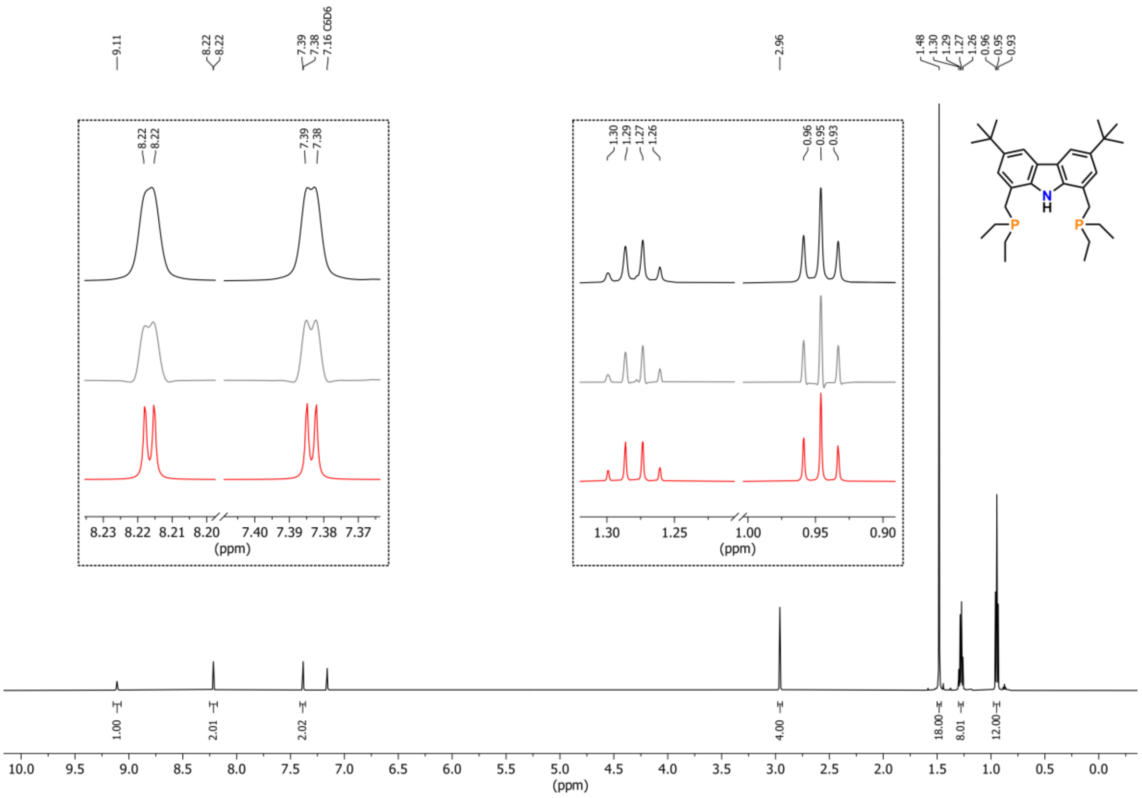

Figure S23. $^{1}H\{^{31}P\}$ NMR spectrum (C_6D_6, 600 MHz, 295 K) of **(^{Et}PNP)H (1^{Et})**. The grey spectra (insets, middle) show regions of the experimental spectrum with LB = -1.2 Hz, GB = 0.4 using a gaussian window function. Simulated multiplets are depicted in red.

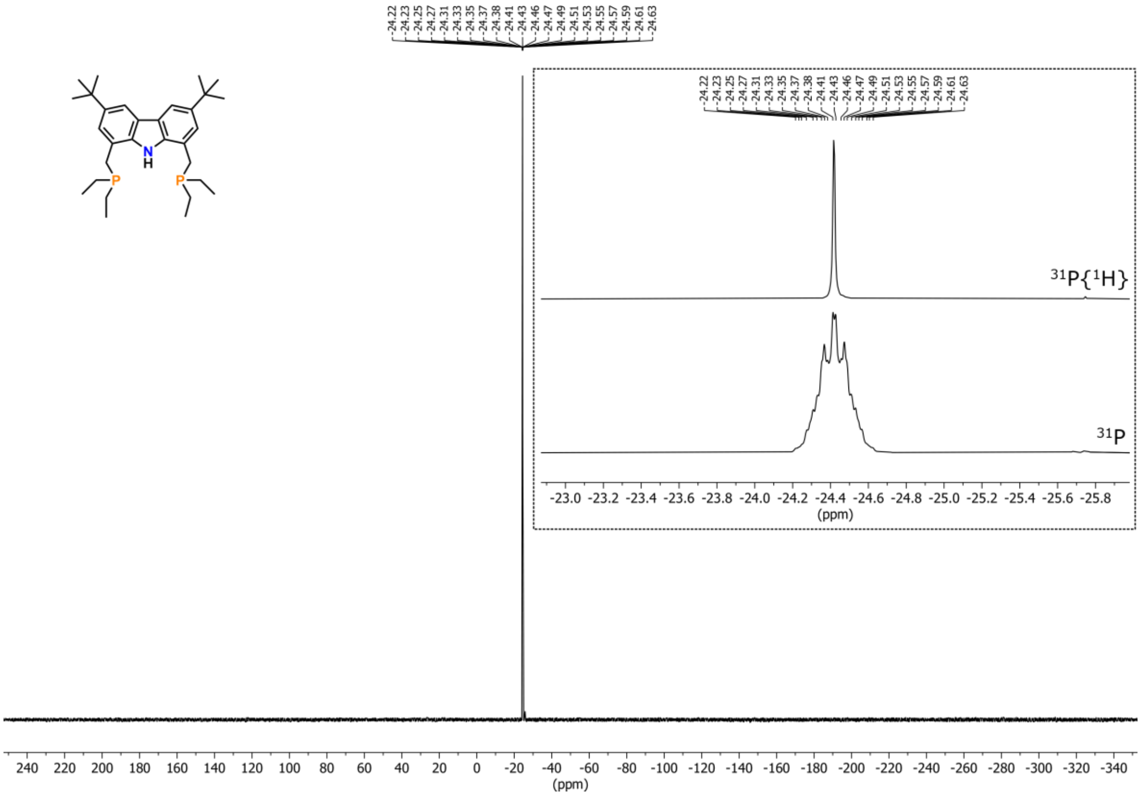

Figure S24. ^{31}P and $^{31}P\{^{1}H\}$ NMR spectrum (C_6D_6, 243 MHz, 295K) of **(^{Et}PNP)H (1^{Et})**.

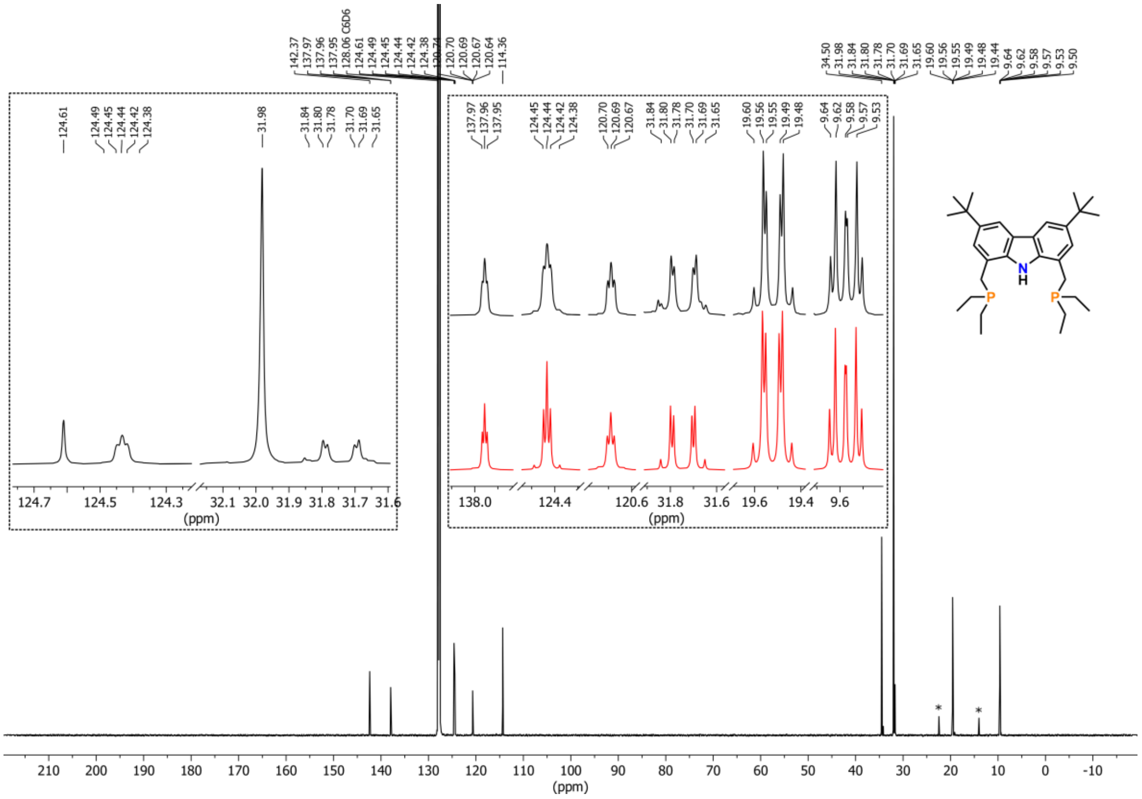

Figure S25. $^{13}C\{^1H\}$ NMR spectrum (C_6D_6, 151 MHz, 295 K) of **(^{Et}PNP)H (1^{Et})**. Simulated multiplets are depicted in red, * = *n*-pentane. The inset on the left shows carbon resonances with small relative chemical shifts for clarity.

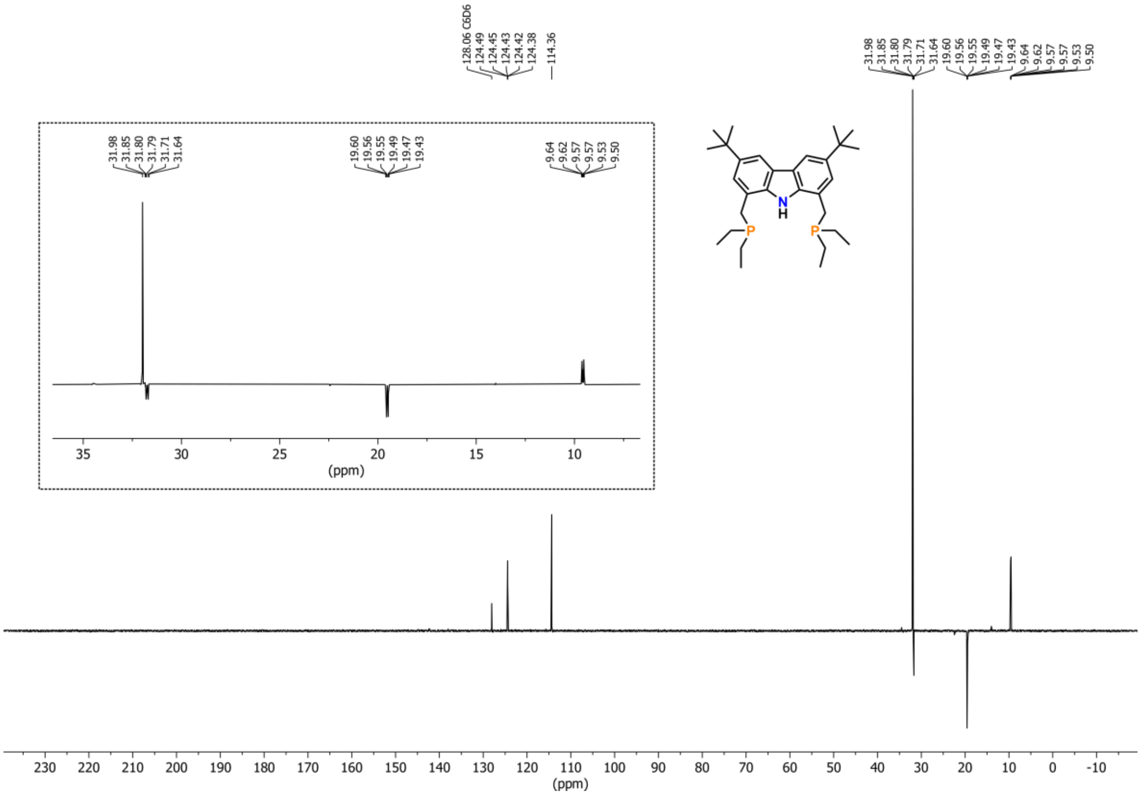

Figure S26. $^{13}C\{^1H\}$ DEPT-135 NMR spectrum (C_6D_6, 151 MHz, 295 K) of **(^{Et}PNP)H (1^{Et})**.

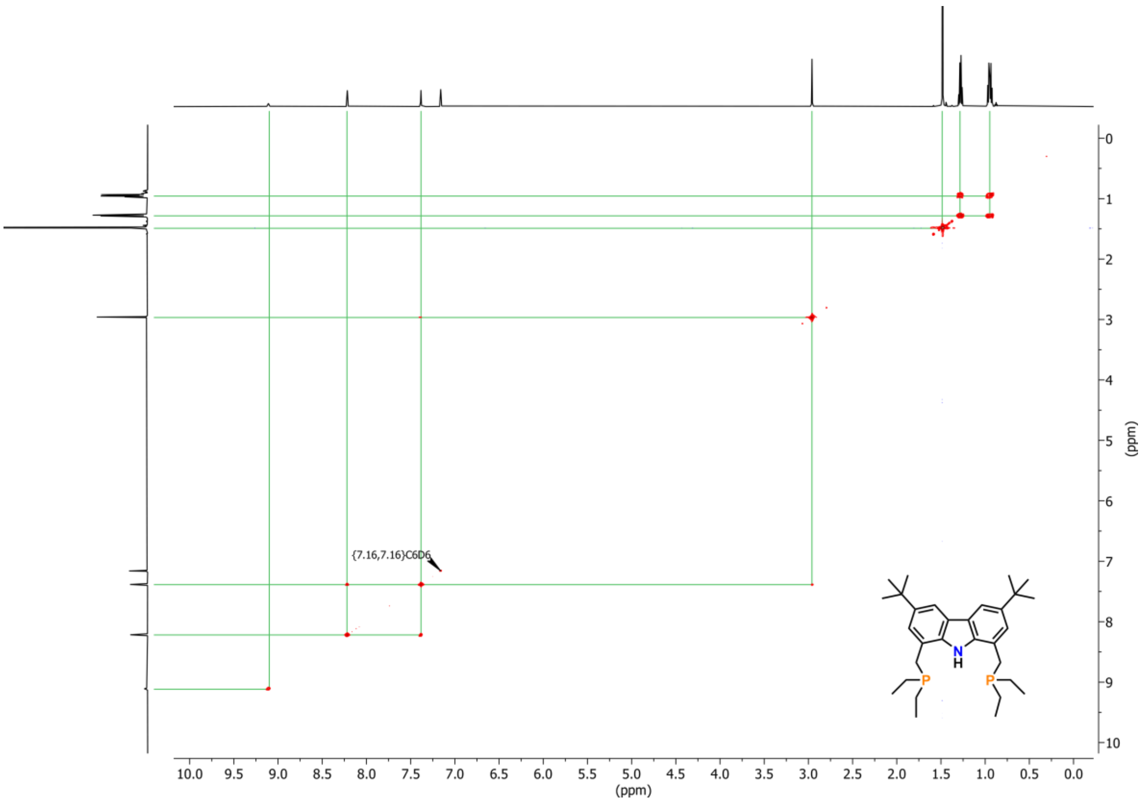

Figure S27. $^1H/^1H$ COSY NMR spectrum (C_6D_6, 600/600 MHz, 295 K) of **(^{Et}PNP)H (1^{Et})**.

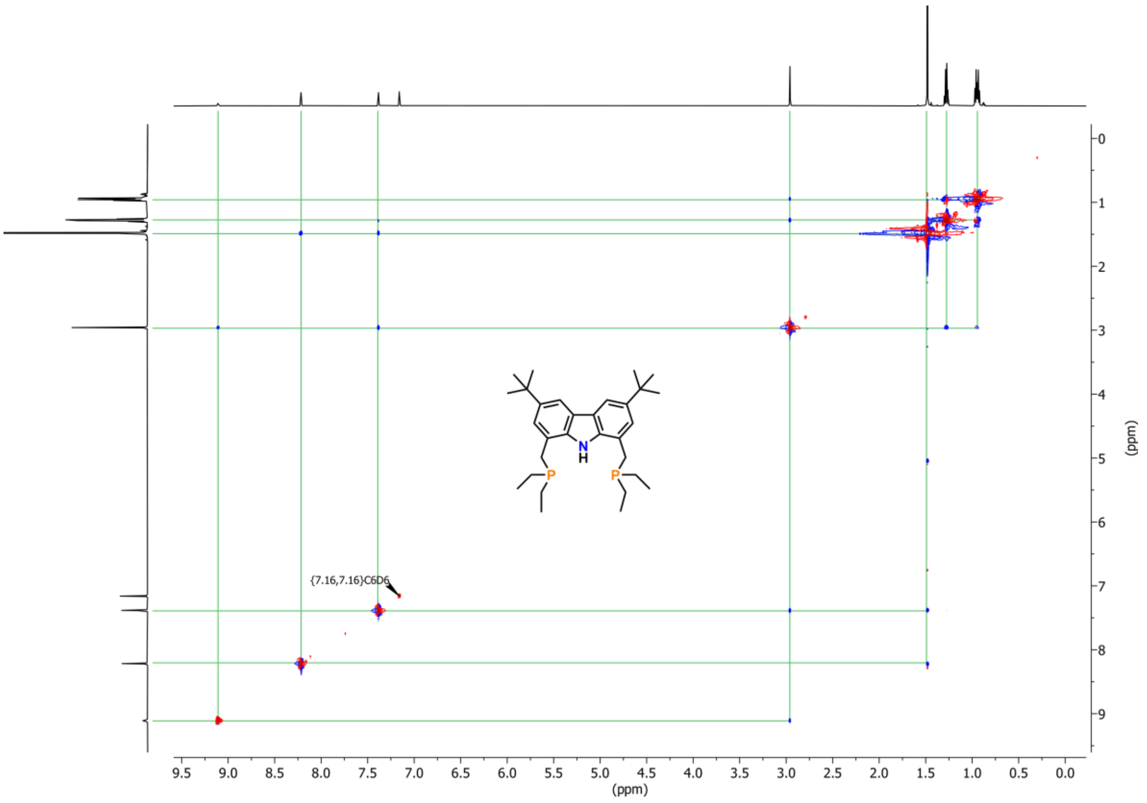

Figure S28. $^1H/^1H$ NOESY NMR spectrum (C_6D_6, 600/600 MHz, 295 K) of **(^{Et}PNP)H (1^{Et})**.

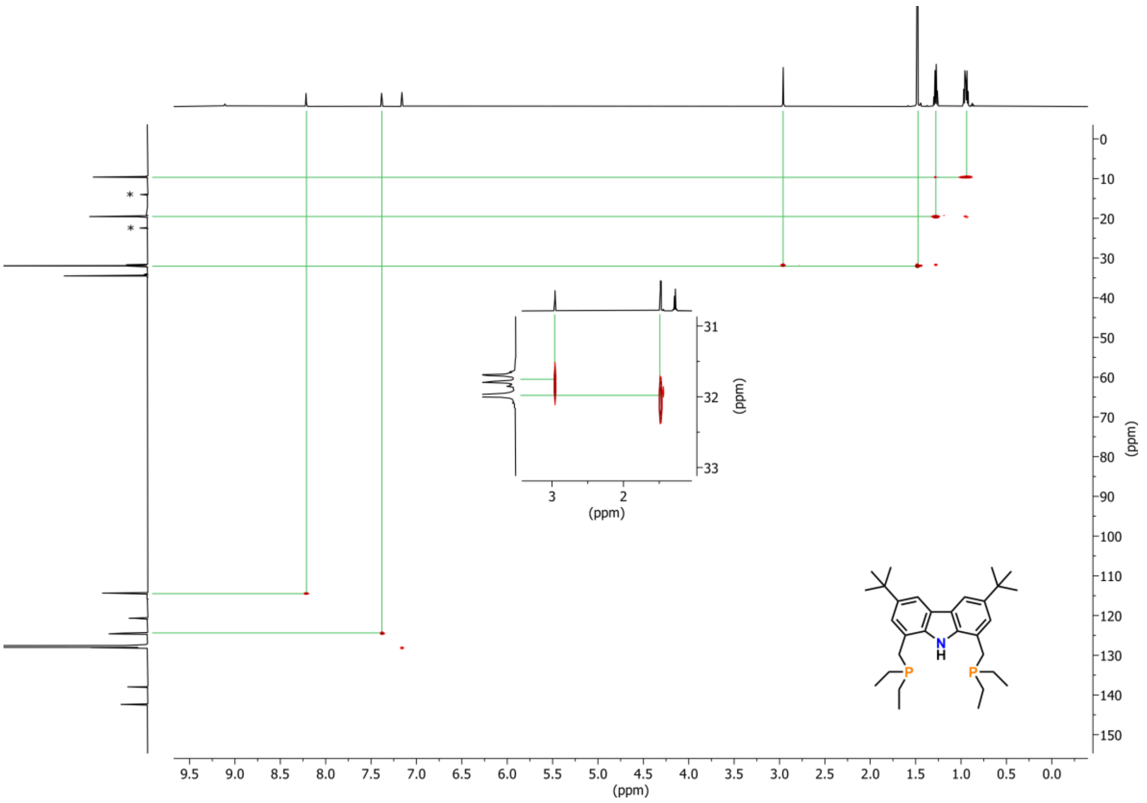

Figure S29. $^{1}H/^{13}C$ HSQC NMR spectrum (C_6D_6, 600/151 MHz, 295 K) of **(^{Et}PNP)H (1^{Et})** (* = *n*-pentane).

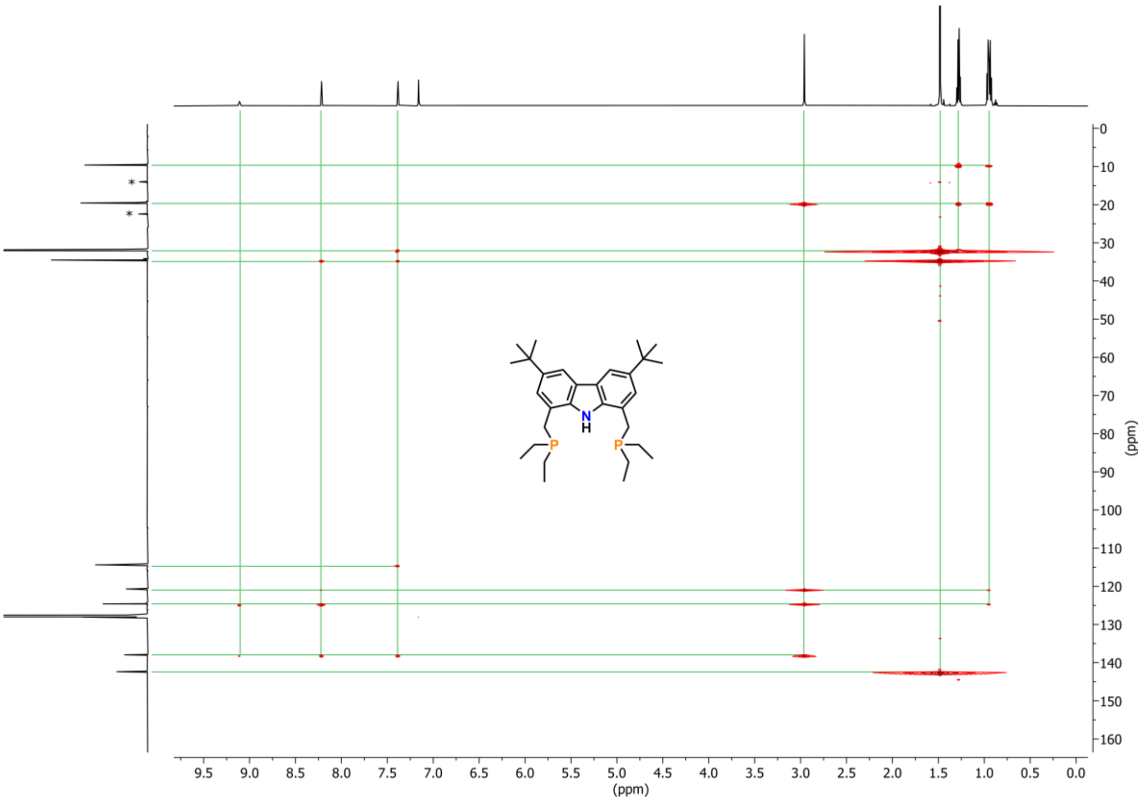

Figure S30. $^{1}H/^{13}C$ HMBC NMR spectrum (C_6D_6, 600/151 MHz, 295 K) of **(^{Et}PNP)H (1^{Et})** (* = *n*-pentane).

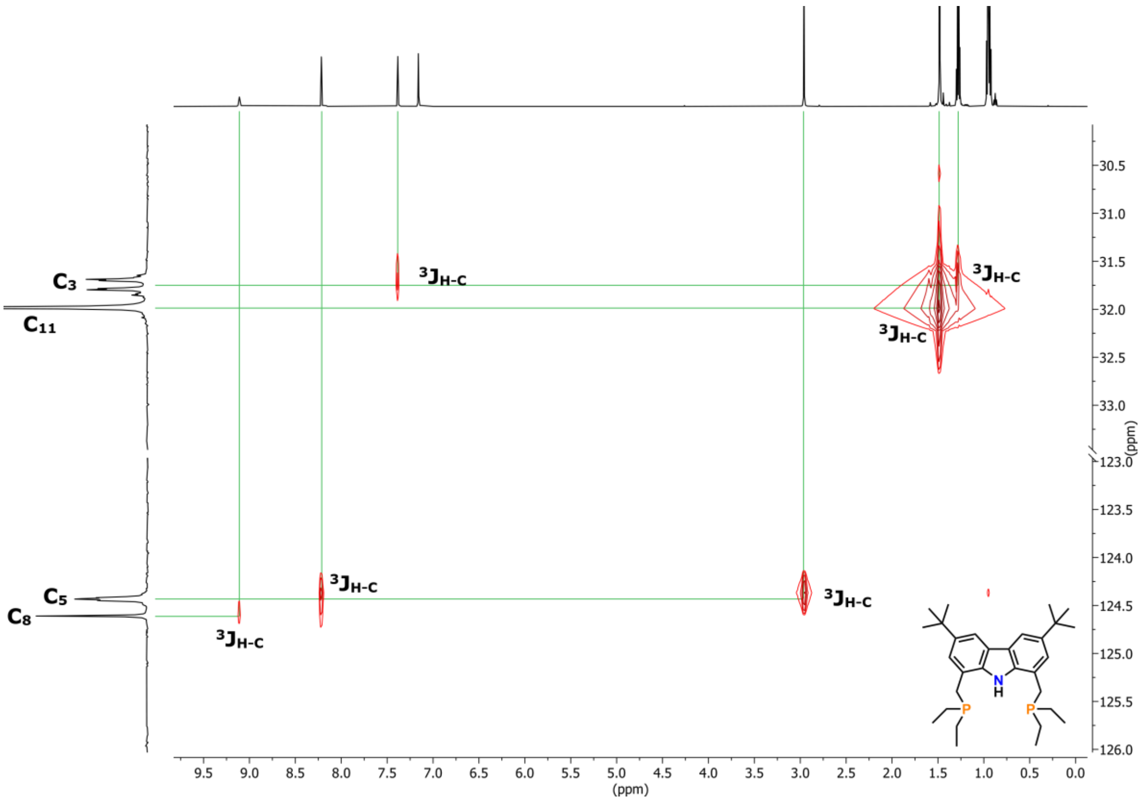

Figure S31. Selected region in the $^{1}H/^{13}C$ HMBC NMR spectrum (C_6D_6, 600/151 MHz, 295 K) of **(^{Et}PNP)H (1^{Et})** for clarity.

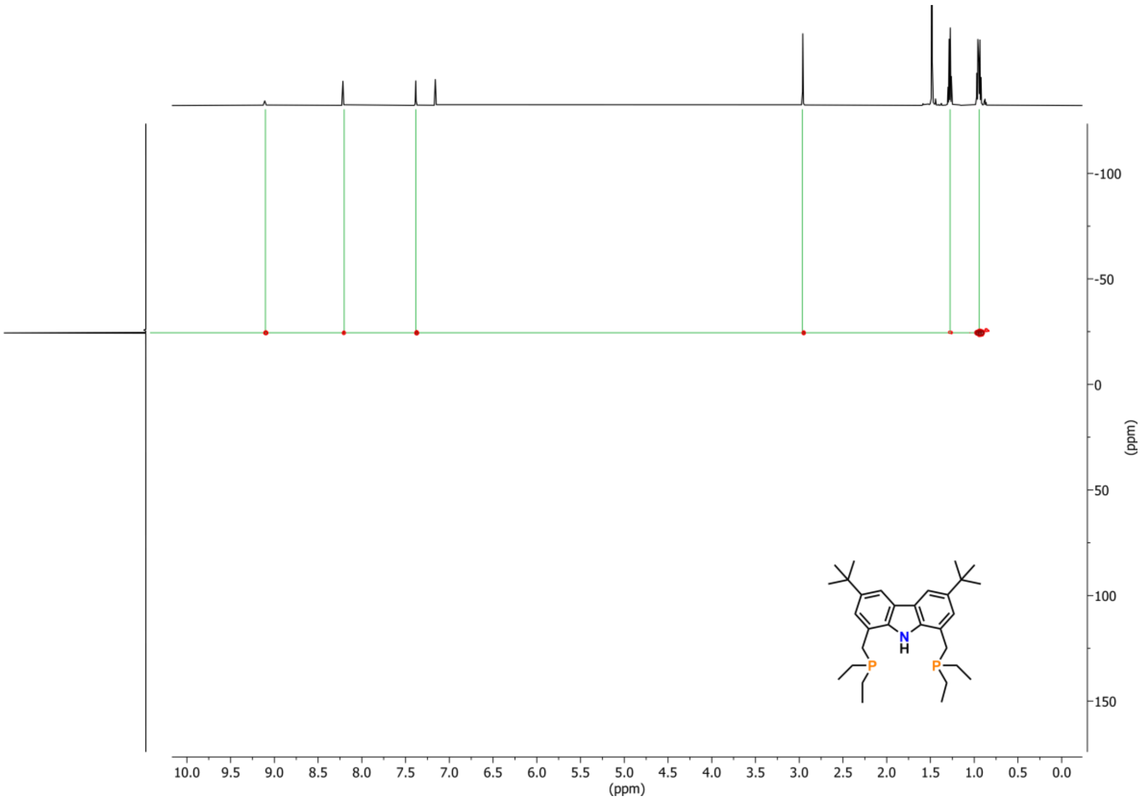

Figure S32. $^{1}H/^{31}P$ HMBC NMR spectrum (C_6D_6, 600/243 MHz, 295 K) of **(^{Et}PNP)H (1^{Et})**.

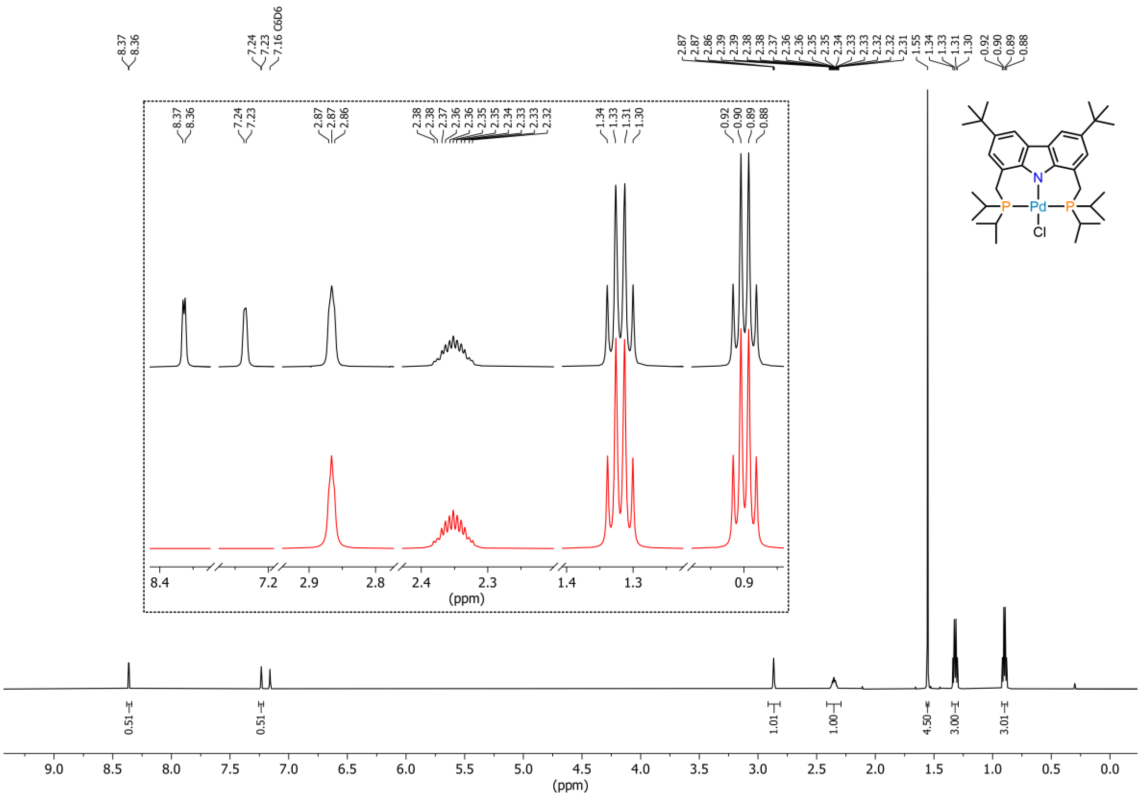

Figure S33. ^{1}H NMR spectrum (C_6D_6, 600 MHz, 295 K) of **[(^{iPr}PNP)PdCl] (2^{iPr}-Pd)**. Simulated multiplets are depicted in red.

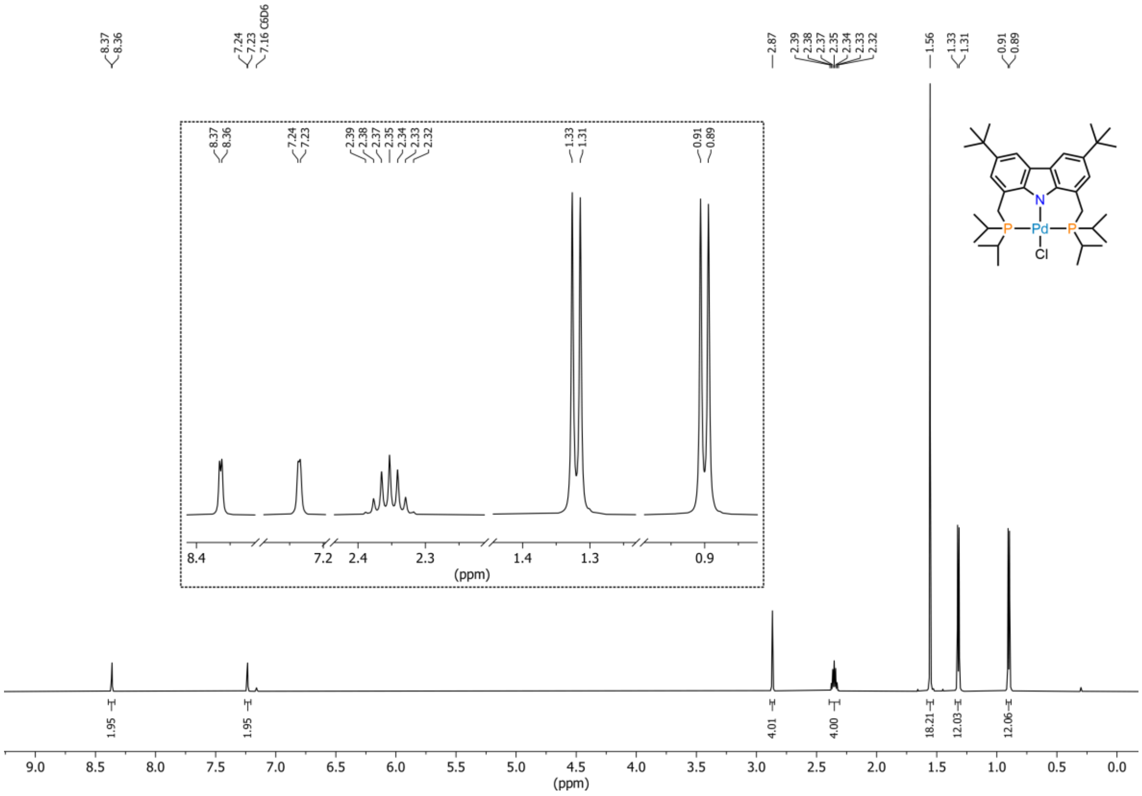

Figure S34. $^{1}H\{^{31}P\}$ NMR spectrum (C_6D_6, 600 MHz, 295 K) of **[(^{iPr}PNP)PdCl] (2^{iPr}-Pd)**.

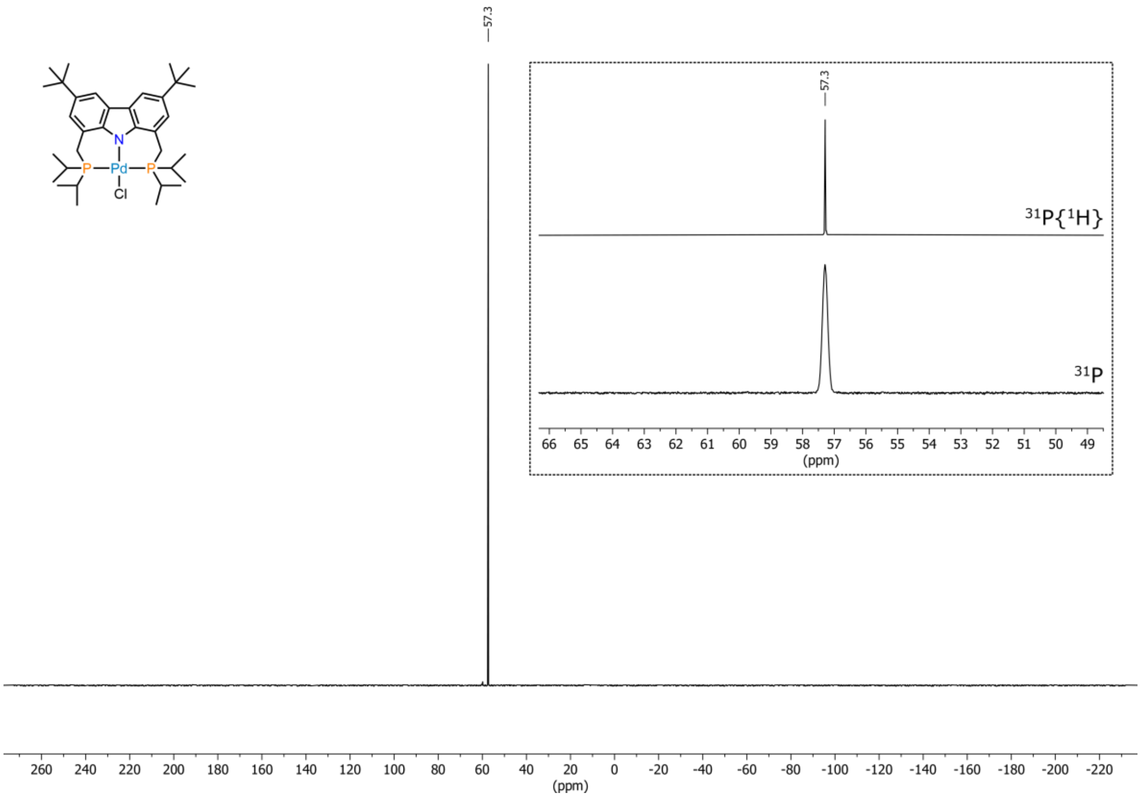

Figure S35. ^{31}P and $^{31}P\{^1H\}$ NMR spectrum (C_6D_6, 243 MHz, 295K) of **[(^{iPr}PNP)PdCl] (2^{iPr}-Pd)**.

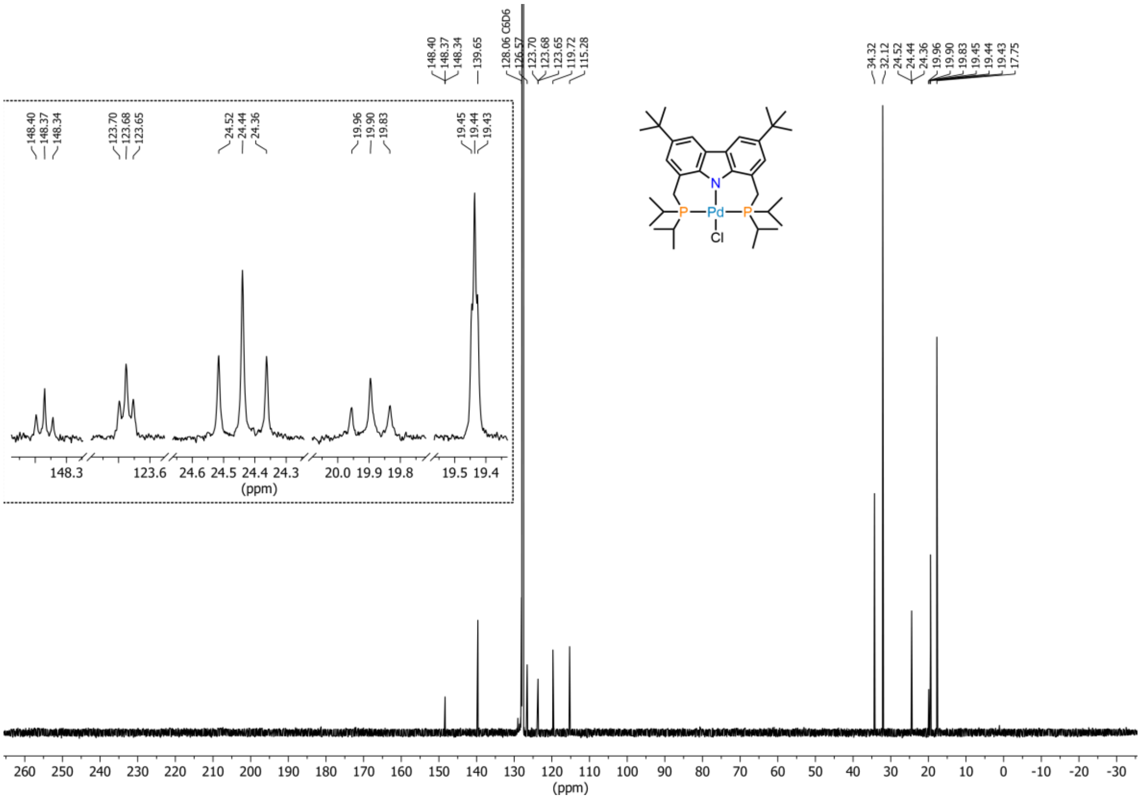

Figure S36. $^{13}C\{^1H\}$ NMR spectrum (C_6D_6, 151 MHz, 295 K) of **[(^{iPr}PNP)PdCl] (2^{iPr}-Pd)**.

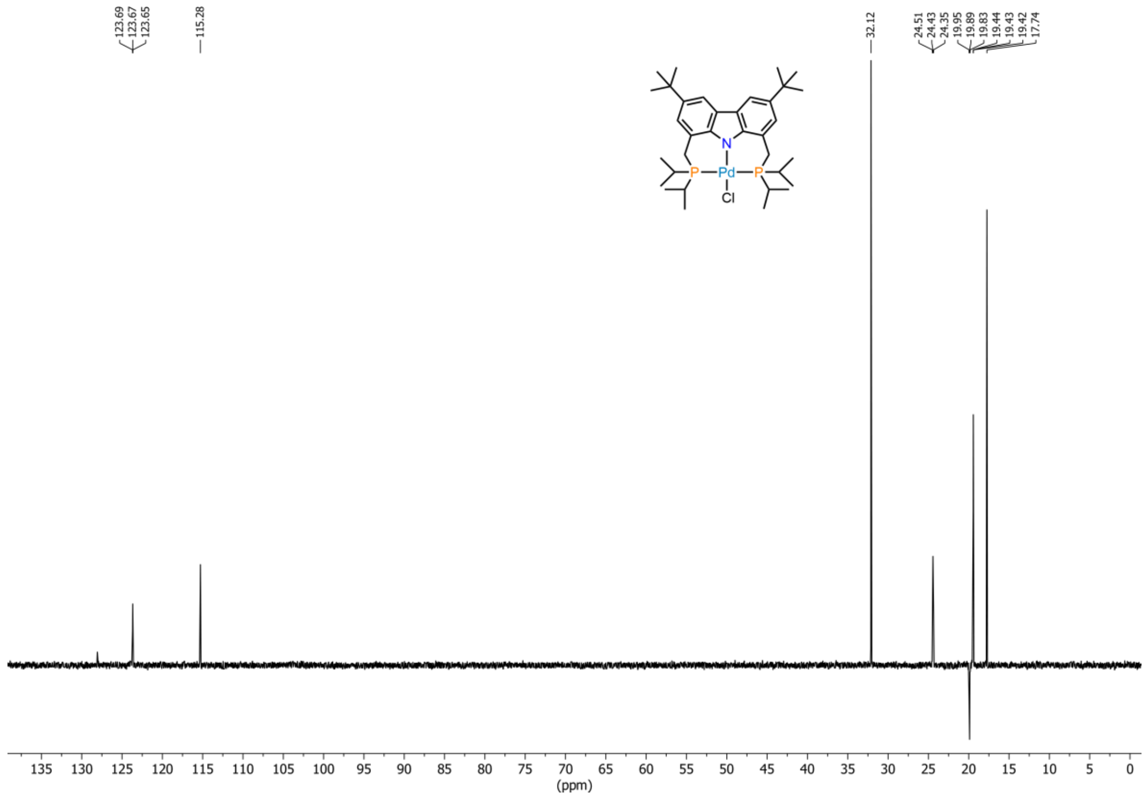

Figure S37. $^{13}C\{^{1}H\}$ DEPT-135 NMR spectrum (C_6D_6, 151 MHz, 295 K) of **[(^{iPr}PNP)PdCl] (2^{iPr}-Pd)**.

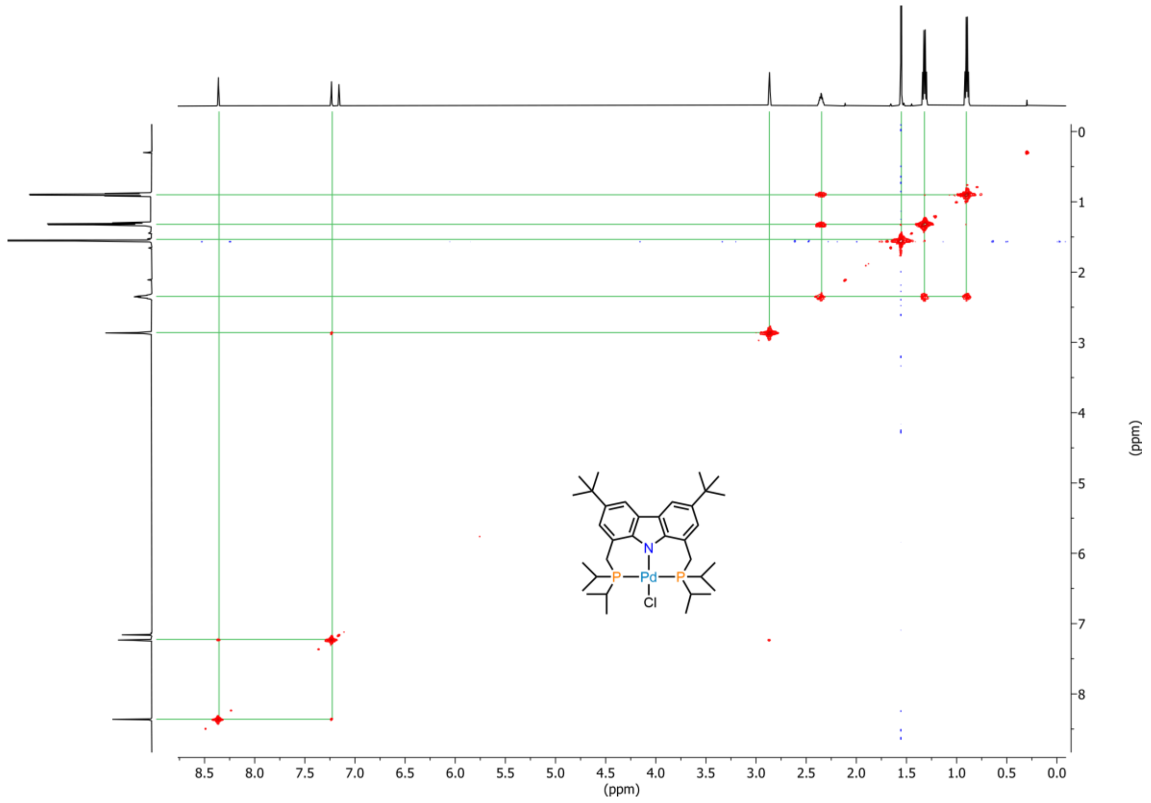

Figure S38. $^{1}H/^{1}H$ COSY NMR spectrum (C_6D_6, 600/600 MHz, 295 K) of **[(^{iPr}PNP)PdCl] (2^{iPr}-Pd)**.

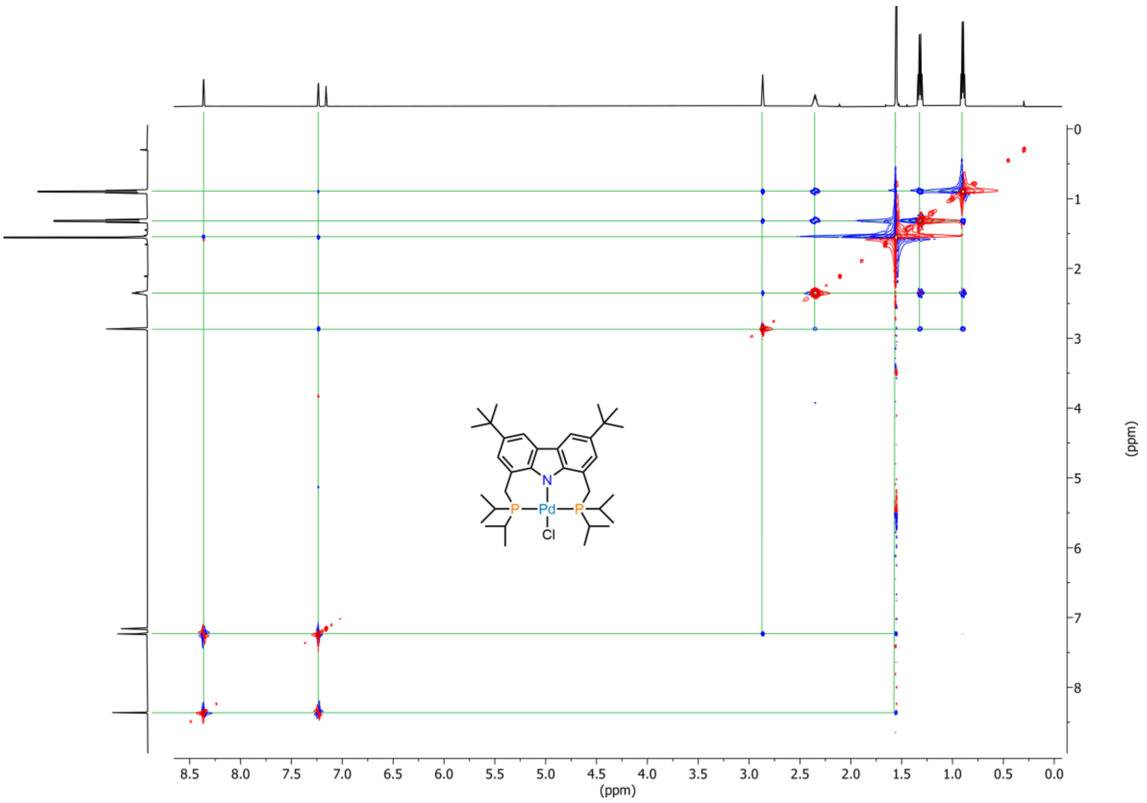

Figure S39. $^1H/^1H$ NOESY NMR spectrum (C_6D_6, 600/600 MHz, 295 K) of **[(^{iPr}PNP)PdCl] (2^{iPr}-Pd)**.

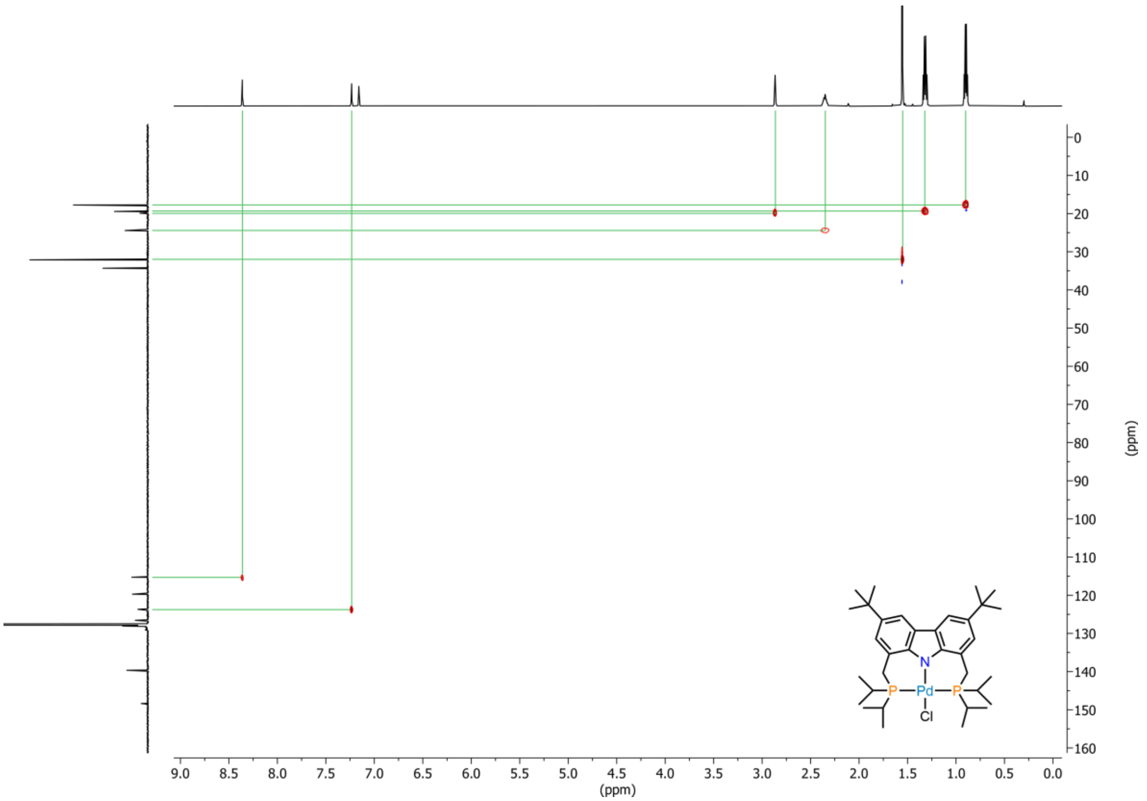

Figure S40. $^1H/^{13}C$ HSQC NMR spectrum (C_6D_6, 600/151 MHz, 295 K) of **[(^{iPr}PNP)PdCl] (2^{iPr}-Pd)**.

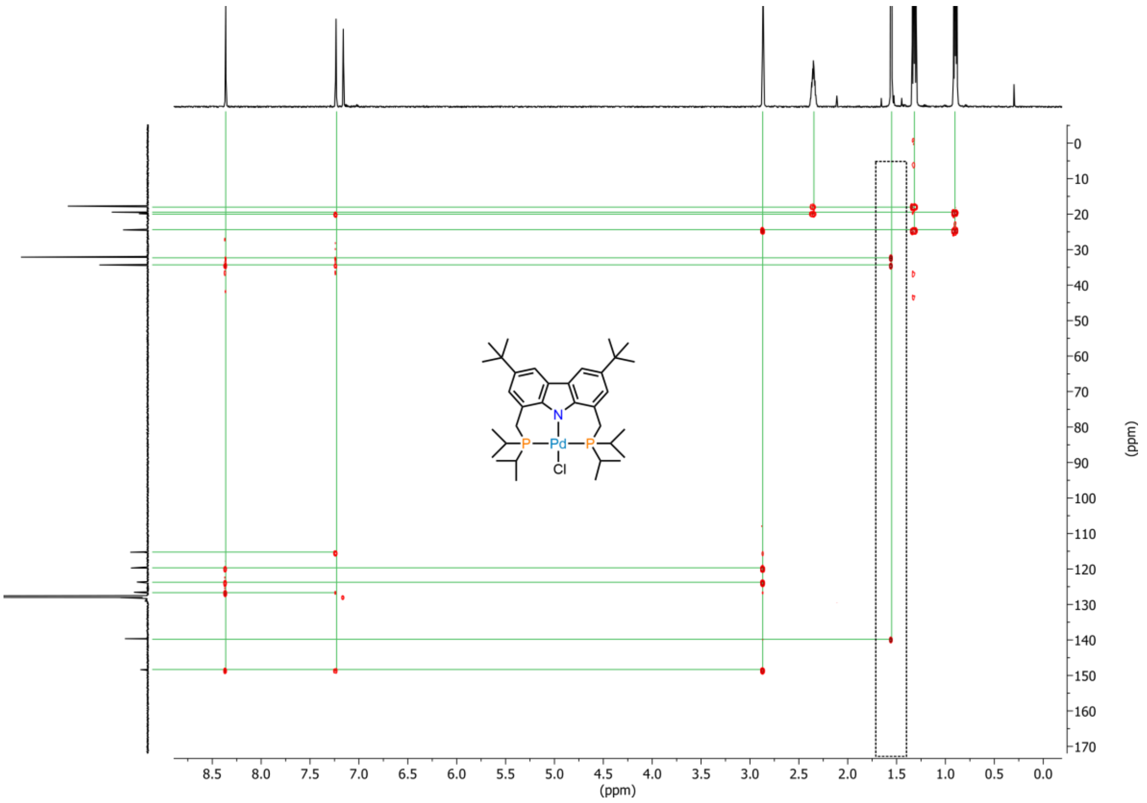

Figure S41. $^{1}H/^{13}C$ HMBC NMR spectrum (C_6D_6, 600/151 MHz, 295 K) of **[(^{iPr}PNP)PdCl] (2^{iPr}-Pd)**. Due to a large dynamic range, the signal intensities were decreased in the inset.

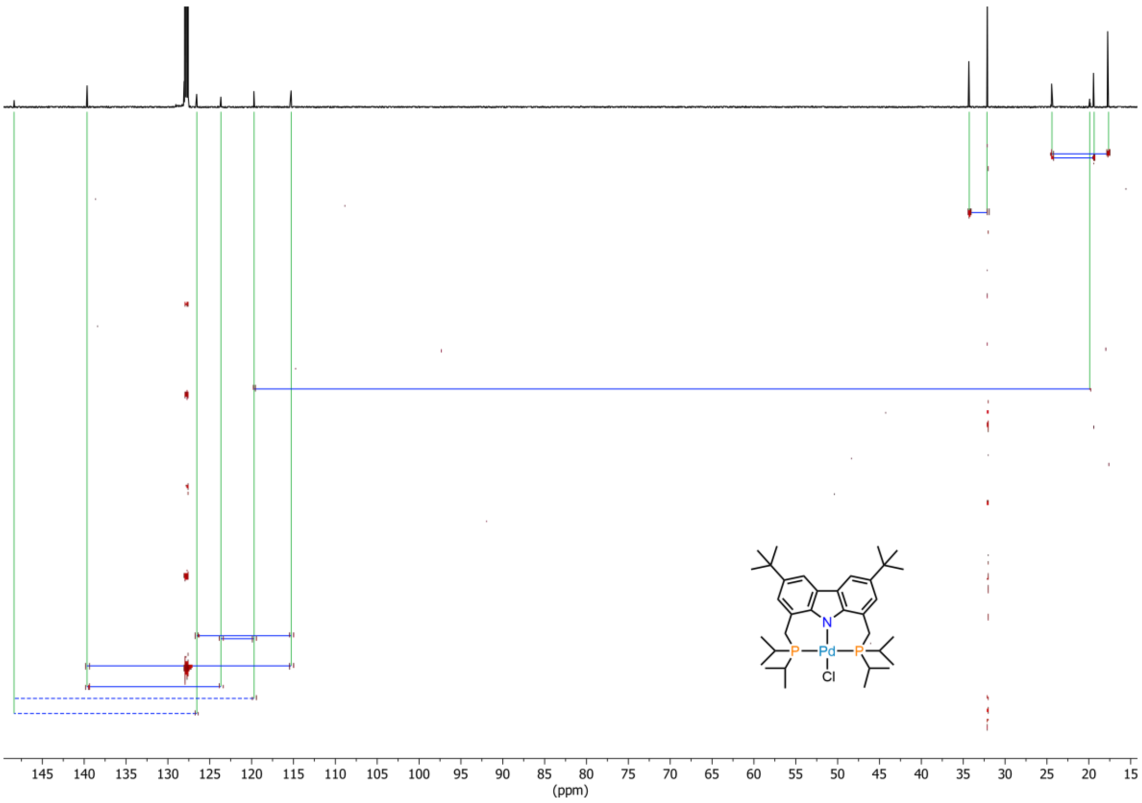

Figure S42. ^{13}C Inadequate NMR spectrum (C_6D_6, 151 MHz, 295 K) of **[(^{iPr}PNP)PdCl] (2^{iPr}-Pd)**.

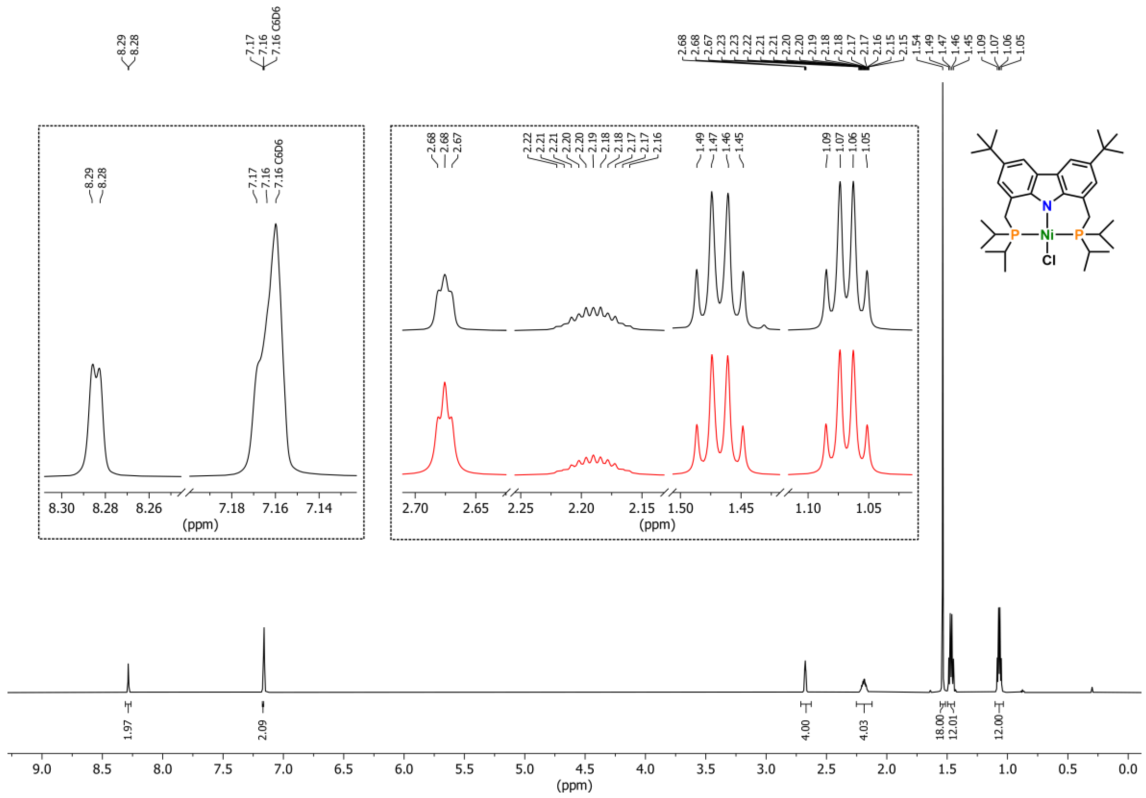

Figure S43. ^{1}H NMR spectrum (C_6D_6, 600 MHz, 295 K) of **[(^{iPr}PNP)NiCl] (2^{iPr}-Ni)**. Simulated multiplets are depicted in red.

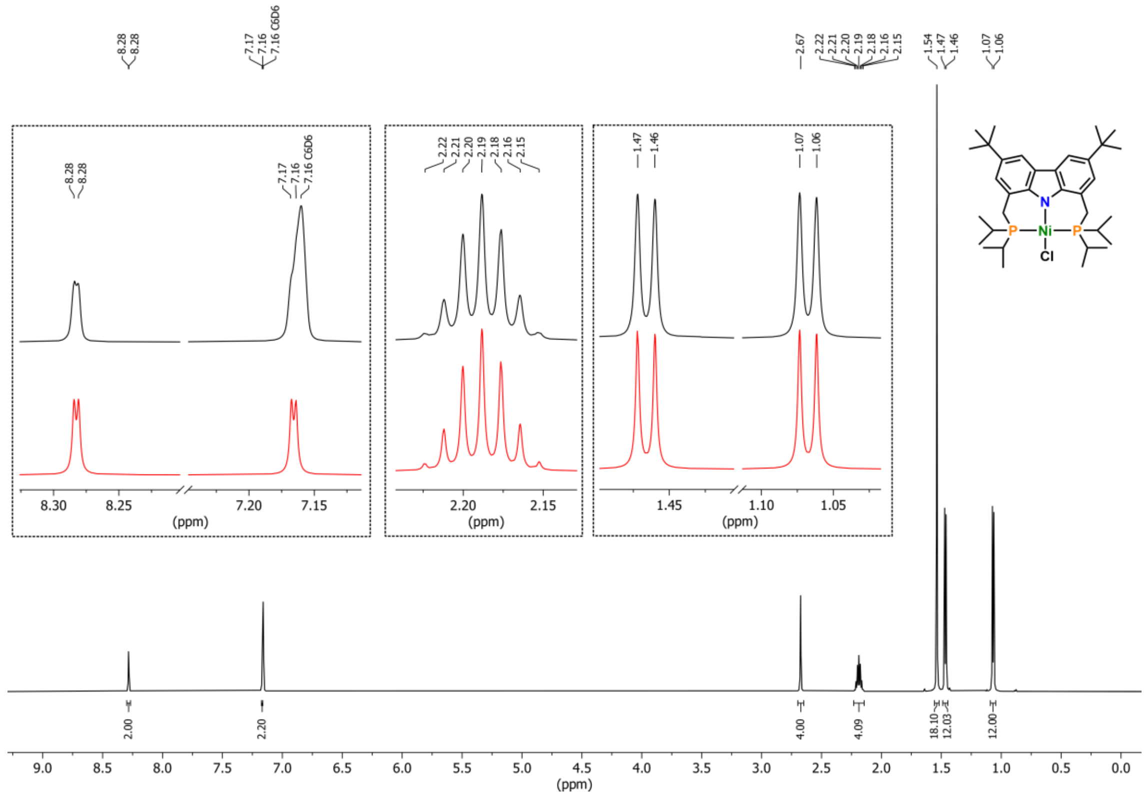

Figure S44. $^{1}H\{^{31}P\}$ NMR spectrum (C_6D_6, 600 MHz, 295 K) of **[(^{iPr}PNP)NiCl] (2^{iPr}-Ni)**. Simulated multiplets are depicted in red.

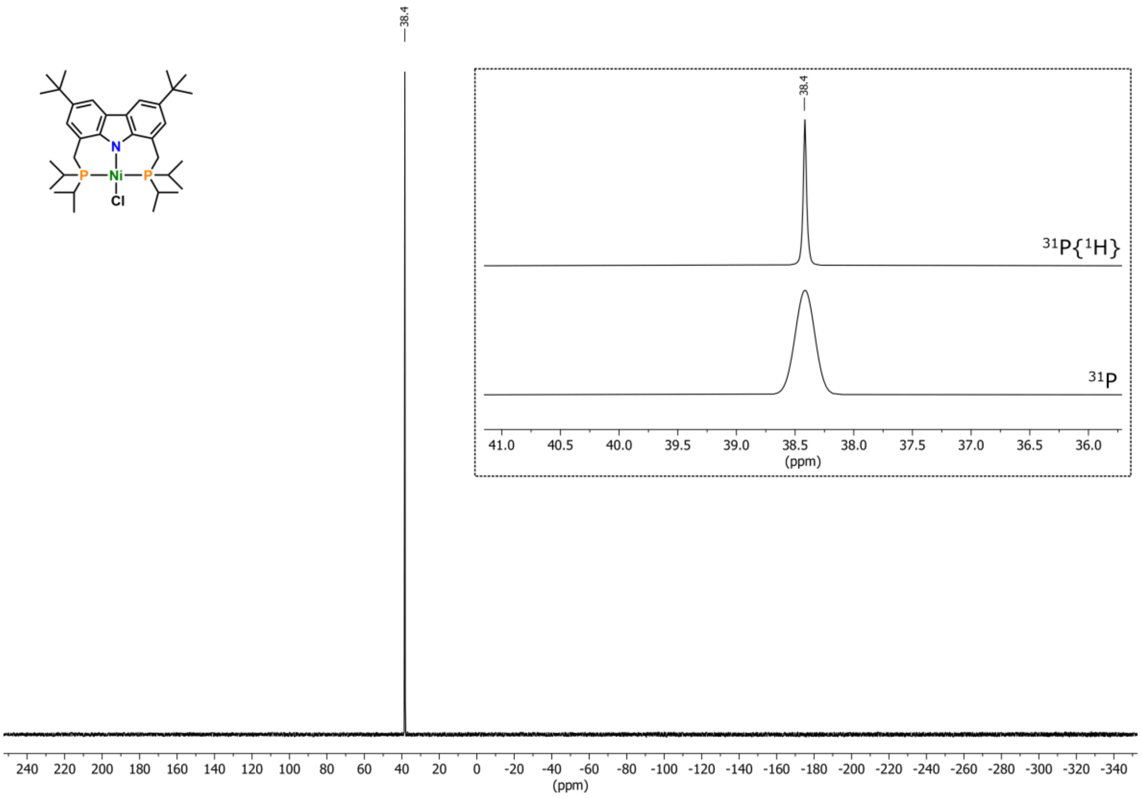

Figure S45. ^{31}P and $^{31}P\{^1H\}$ NMR spectrum (C_6D_6, 243 MHz, 295K) of **[(^{iPr}PNP)NiCl] (2^{iPr}-Ni)**.

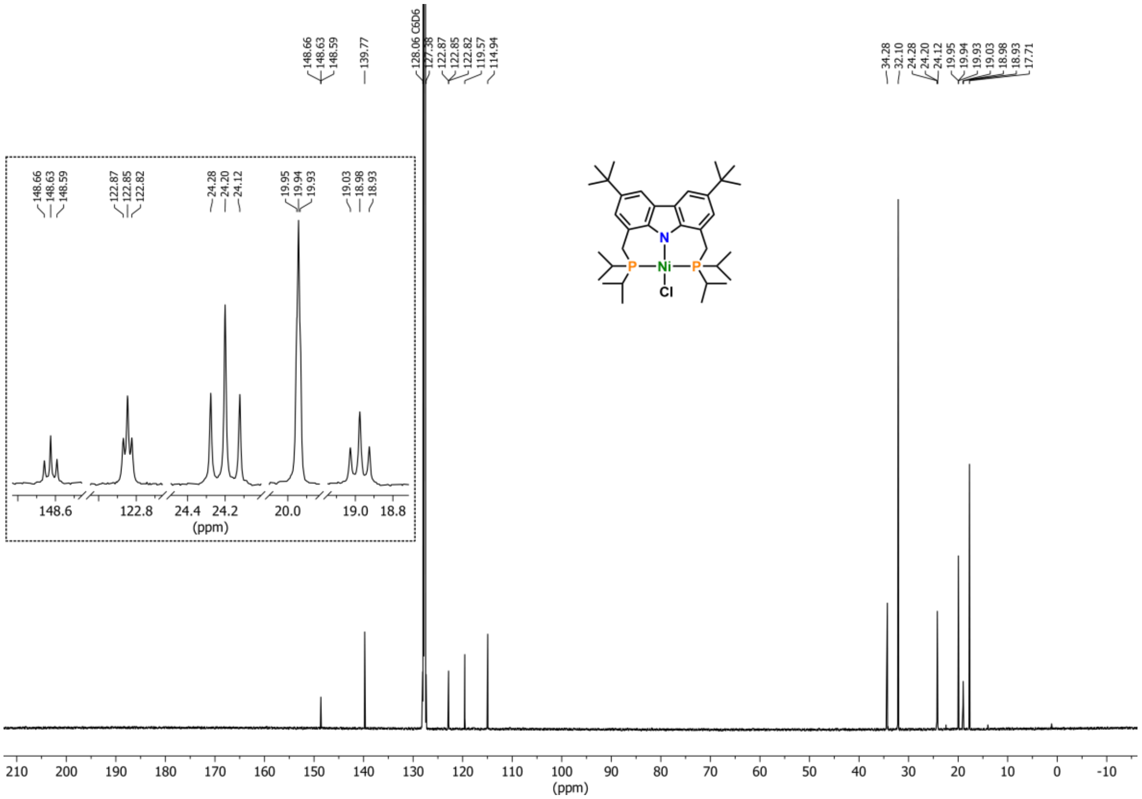

Figure S46. $^{13}C\{^1H\}$ NMR spectrum (C_6D_6, 151 MHz, 295 K) of **[(^{iPr}PNP)NiCl] (2^{iPr}-Ni)**.

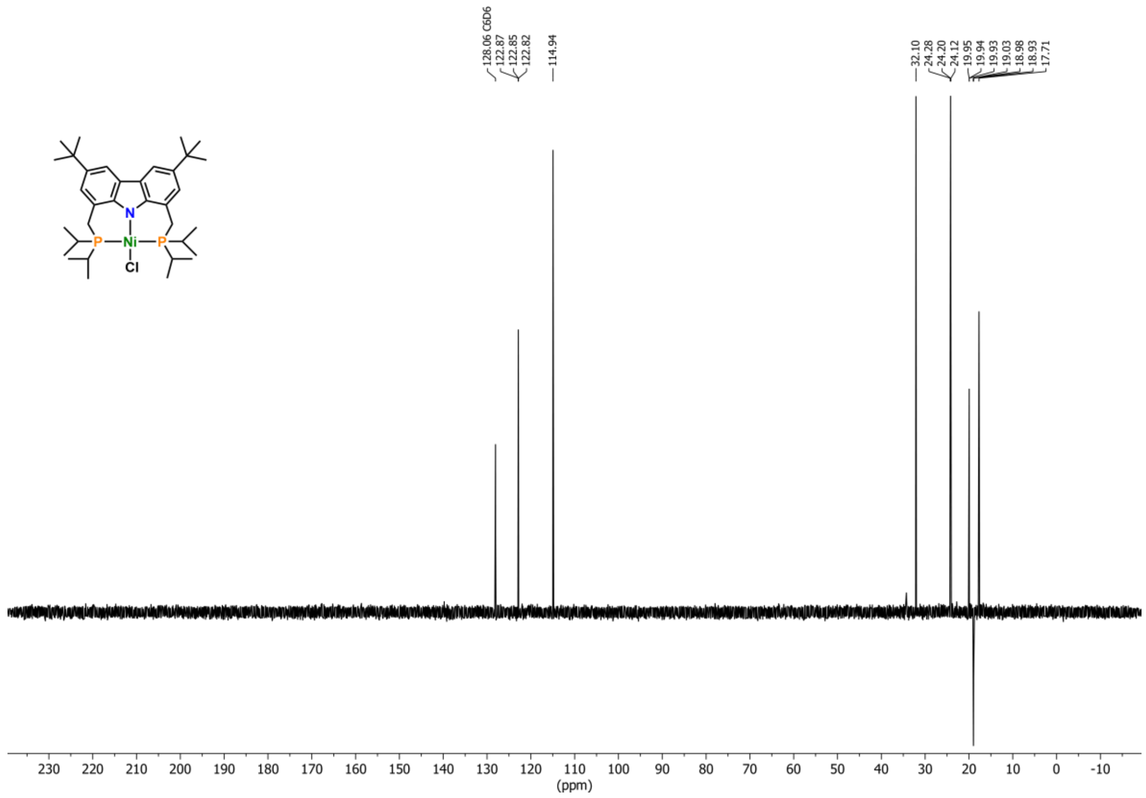

Figure S47. $^{13}C\{^{1}H\}$ DEPT-135 NMR spectrum (C_6D_6, 151 MHz, 295 K) of **[(^{iPr}PNP)NiCl] (2^{iPr}-Ni)**.

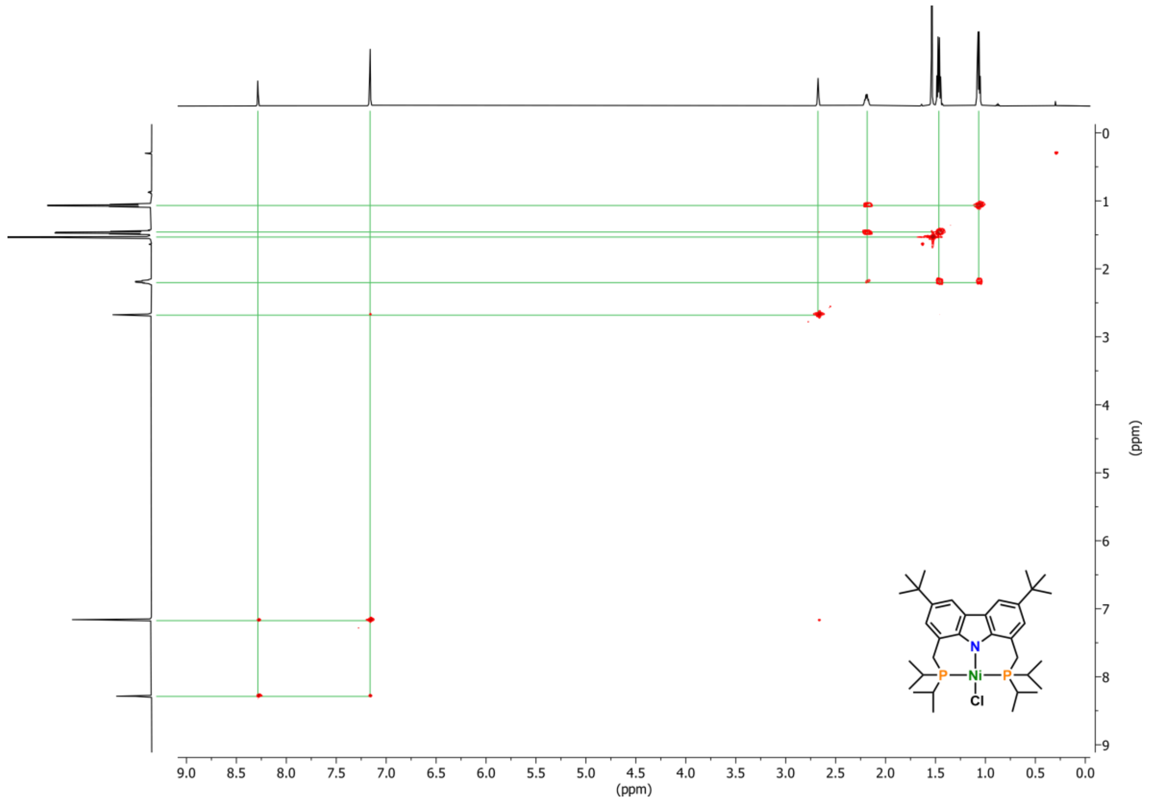

Figure S48. $^{1}H/^{1}H$ COSY NMR spectrum (C_6D_6, 600/600 MHz, 295 K) of **[(^{iPr}PNP)NiCl] (2^{iPr}-Ni)**.

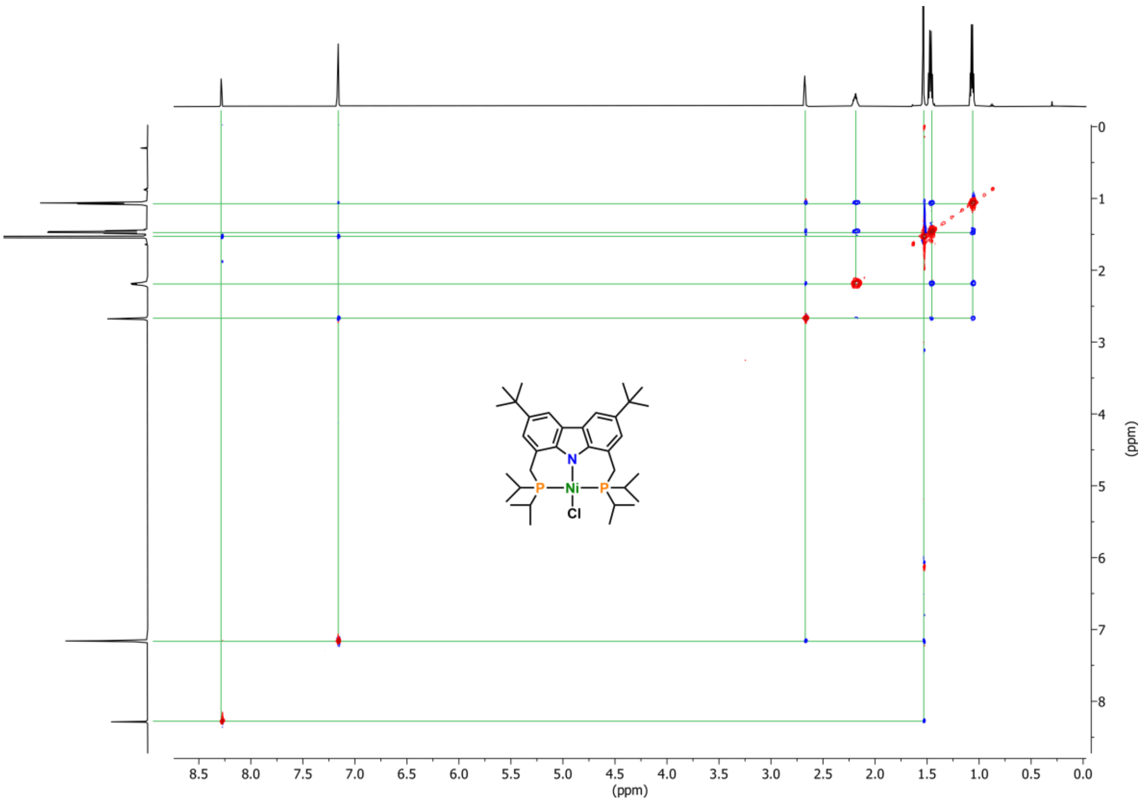

Figure S49. $^1H/^1H$ NOESY NMR spectrum (C_6D_6, 600/600 MHz, 295 K) of **[(^{iPr}PNP)NiCl] (2^{iPr}-Ni)**.

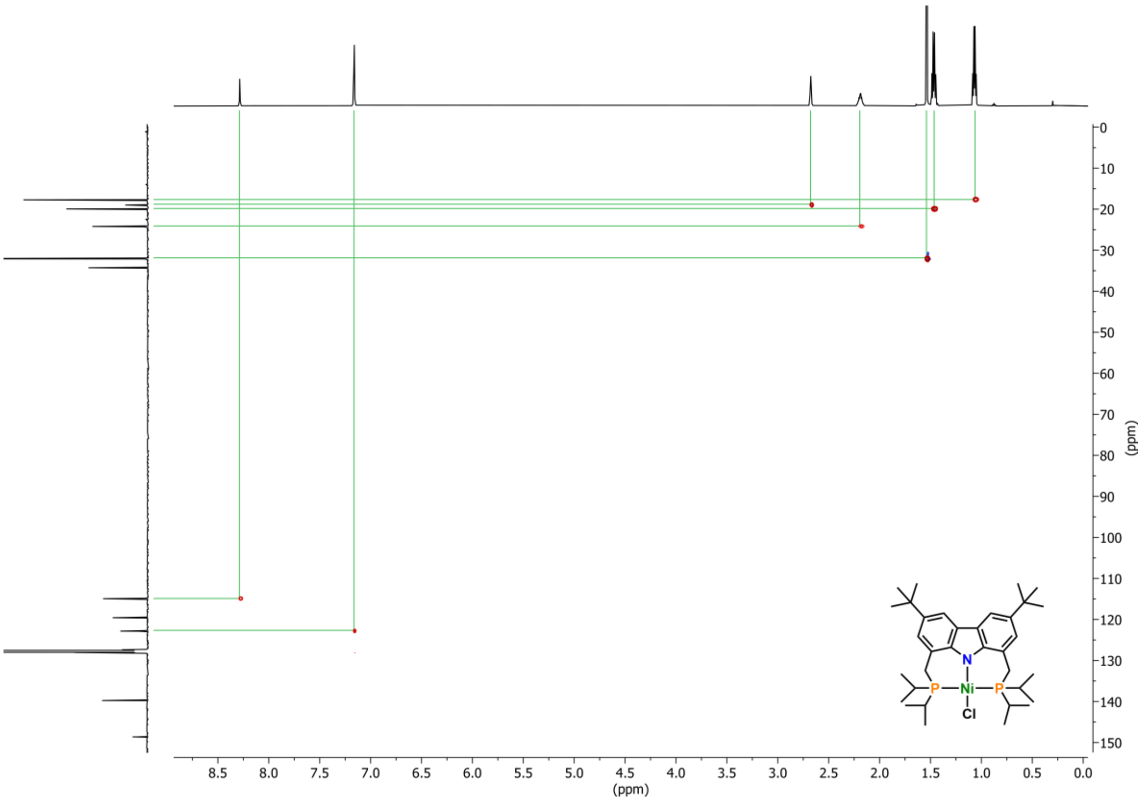

Figure S50. $^1H/^{13}C$ HSQC NMR spectrum (C_6D_6, 600/151 MHz, 295 K) of **[(^{iPr}PNP)NiCl] (2^{iPr}-Ni)**.

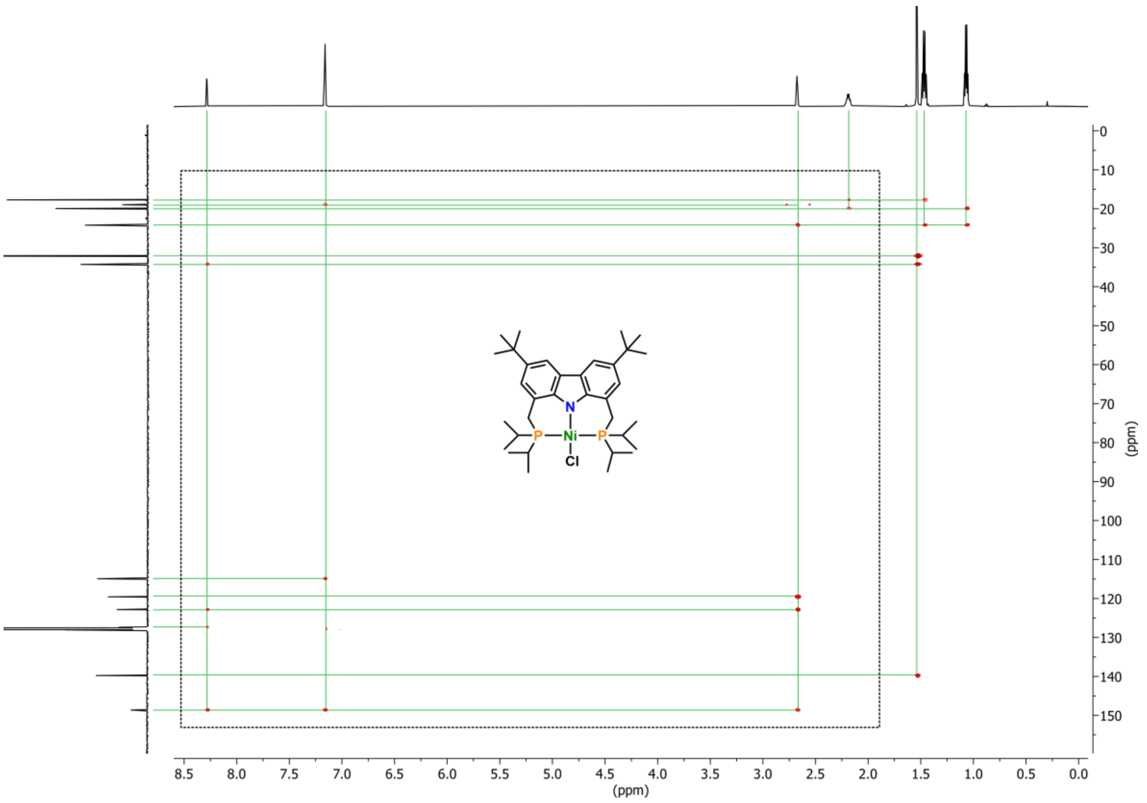

Figure S51. $^{1}H/^{13}C$ HMBC NMR spectrum (C_6D_6, 600/151 MHz, 295 K) of **[(^{iPr}PNP)NiCl] (2^{iPr}-Ni)**. Due to a large dynamic range, the weaker signals were amplified in the inset.

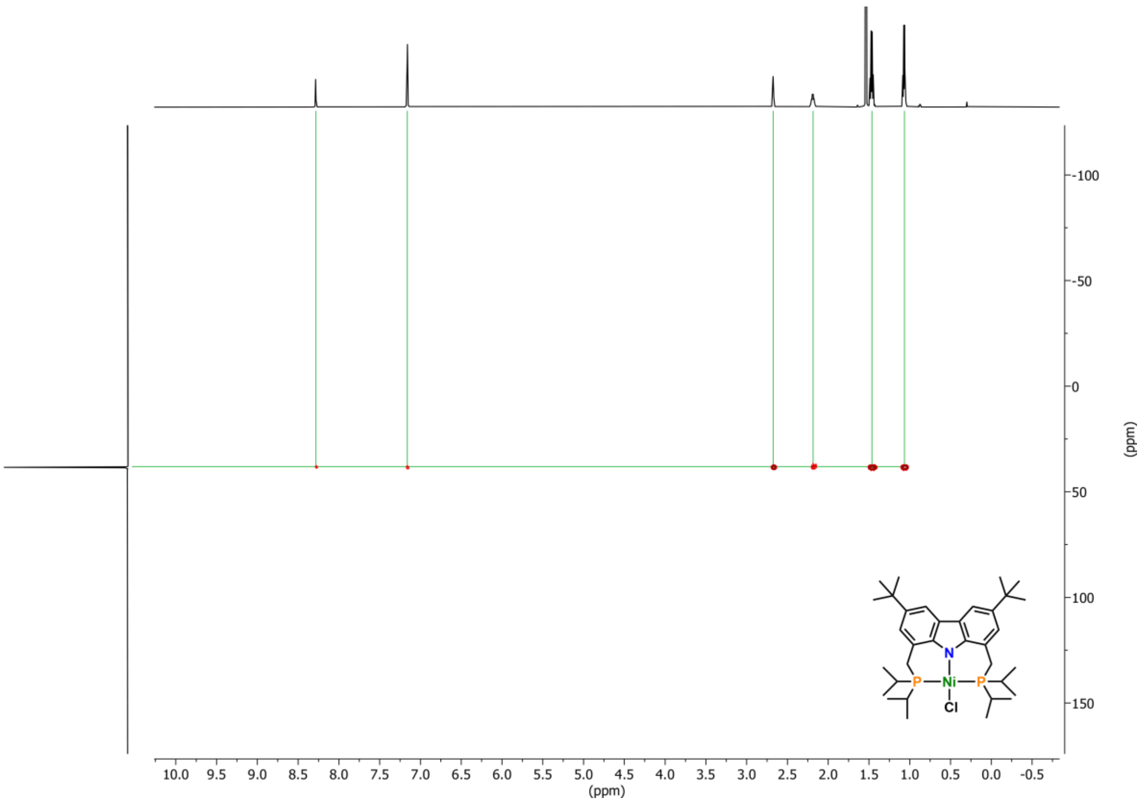

Figure S52. $^{1}H/^{31}P$ HMBC NMR spectrum (C_6D_6, 600/243 MHz, 295 K) of **[(^{iPr}PNP)NiCl] (2^{iPr}-Ni)**.

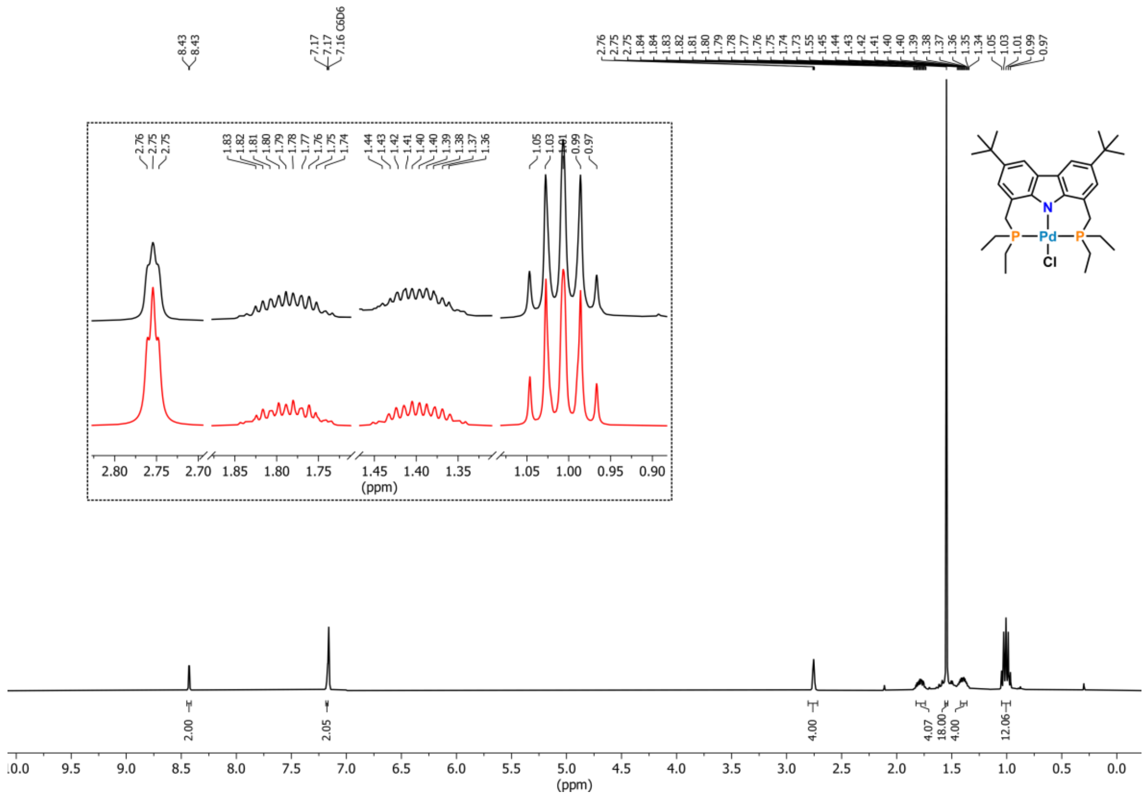

Figure S53. ^{1}H NMR spectrum (C_6D_6, 400 MHz, 295 K) of **[(^{Et}PNP)PdCl] (2^{Et}-Pd)**. Simulated multiplets are depicted in red.

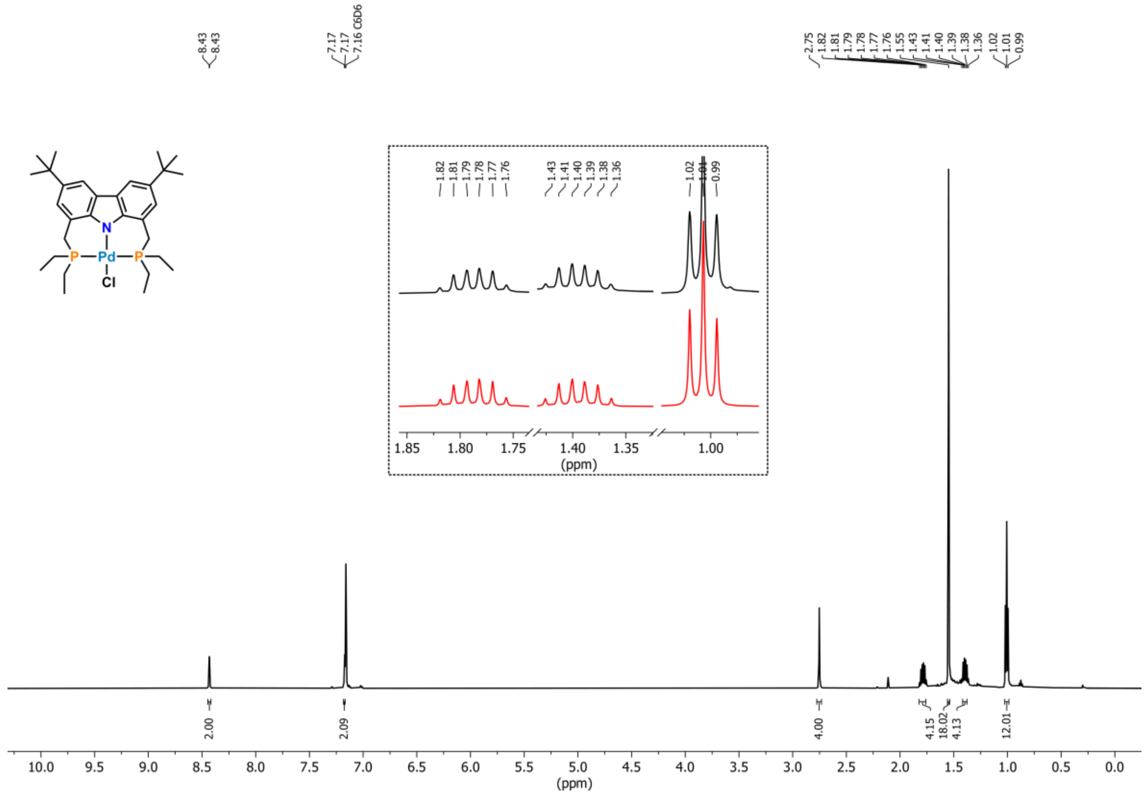

Figure S54. ^{1}H{^{31}P} NMR spectrum (C_6D_6, 600 MHz, 295 K) of **[(^{Et}PNP)PdCl] (2^{Et}-Pd)**. Simulated multiplets are depicted in red.

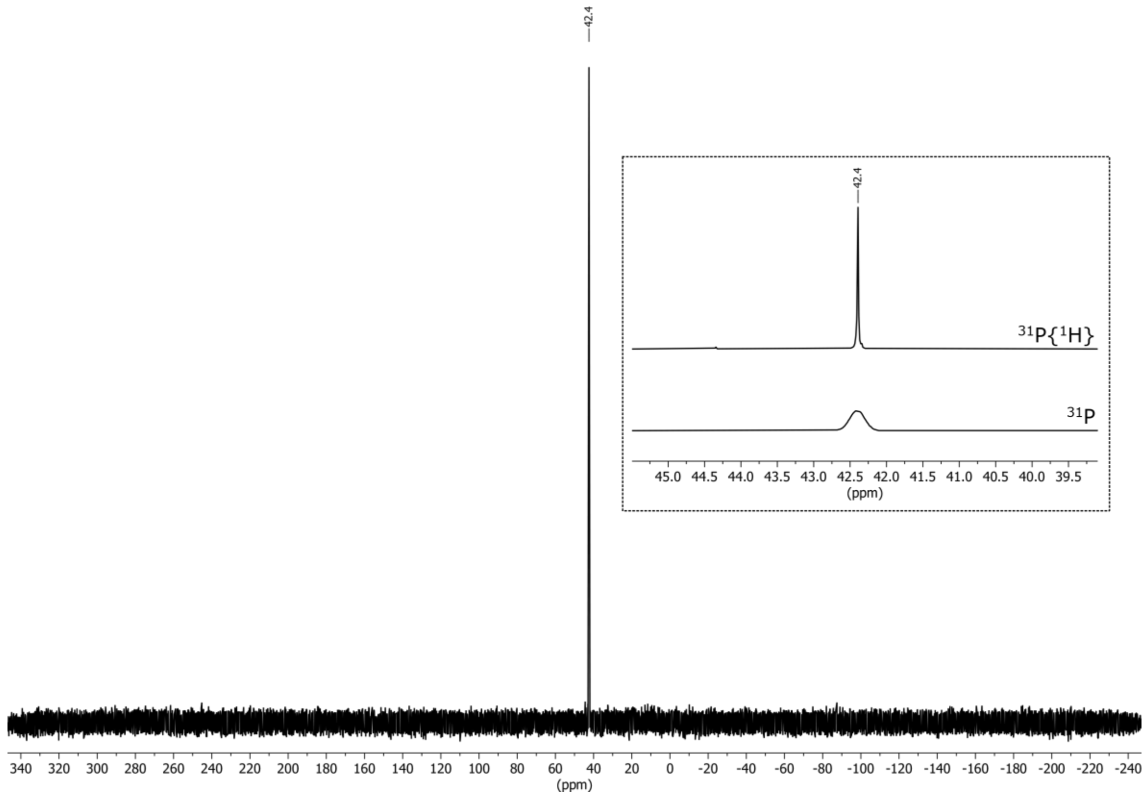

Figure S55. ^{31}P and $^{31}P\{^1H\}$ NMR spectrum (C_6D_6, 162 MHz, 295K) of **[(^{Et}PNP)PdCl] (2^{Et}-Pd)**.

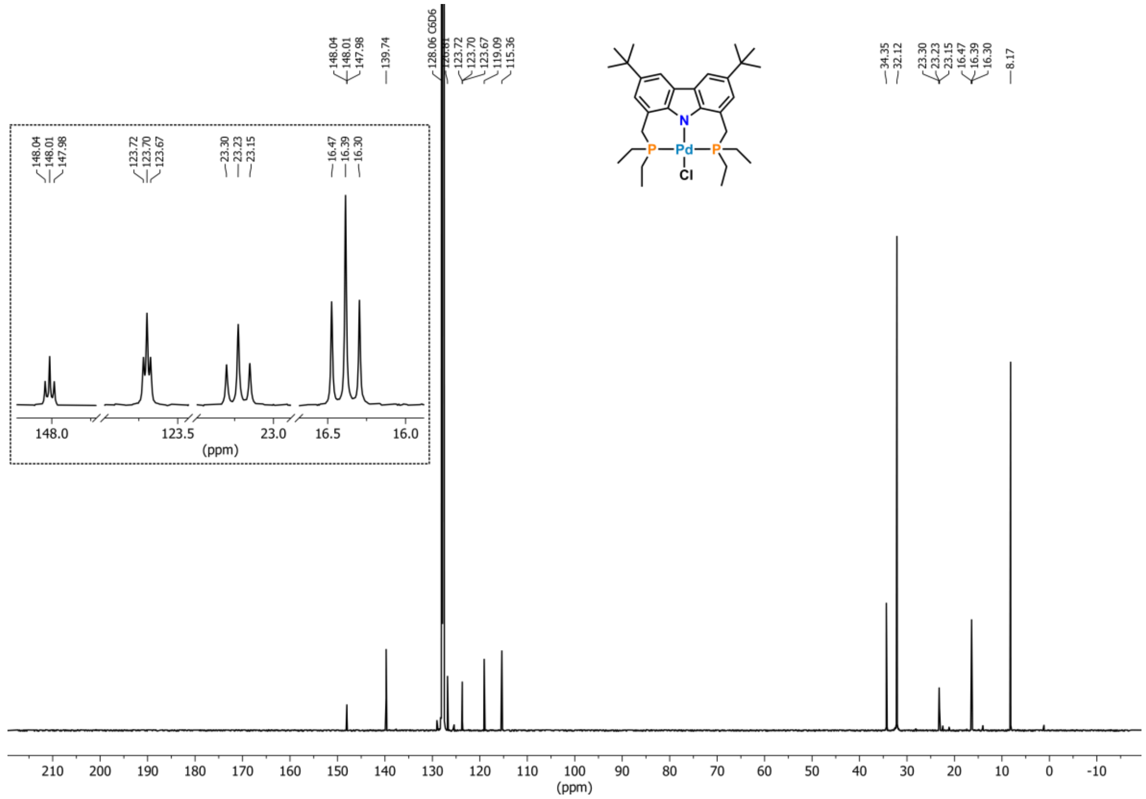

Figure S56. $^{13}C\{^1H\}$ NMR spectrum (C_6D_6, 151 MHz, 295 K) of **[(^{Et}PNP)PdCl] (2^{Et}-Pd)**.

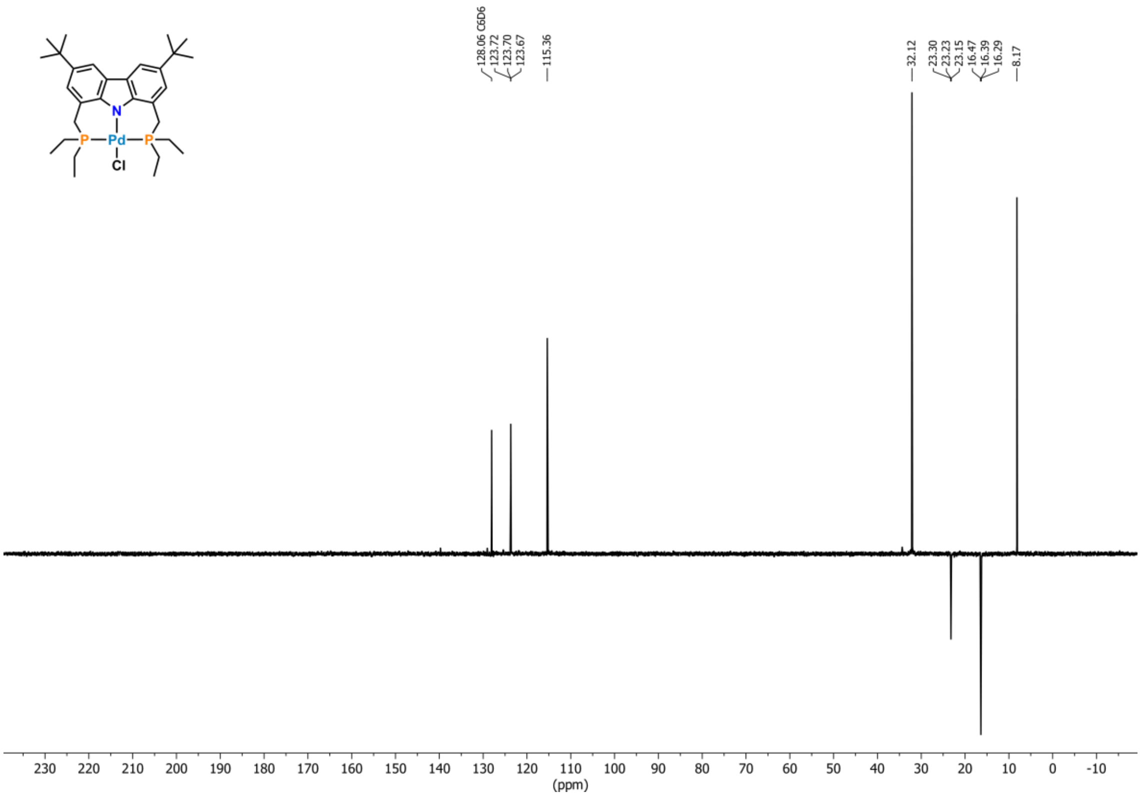

Figure S57. $^{13}C\{^{1}H\}$ DEPT-135 NMR spectrum (C_6D_6, 151 MHz, 295 K) of **[(^{Et}PNP)PdCl] (2^{Et}-Pd)**.

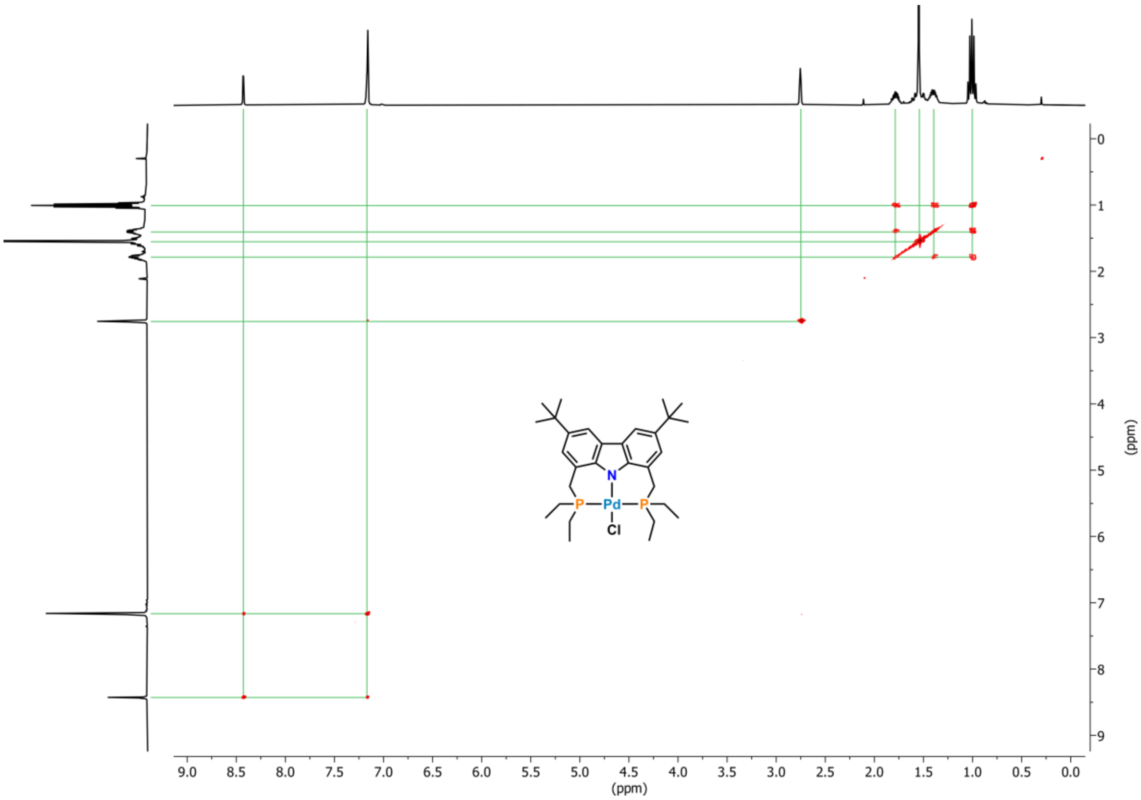

Figure S58. $^{1}H/^{1}H$ COSY NMR spectrum (C_6D_6, 600/600 MHz, 295 K) of **[(^{Et}PNP)PdCl] (2^{Et}-Pd)**.

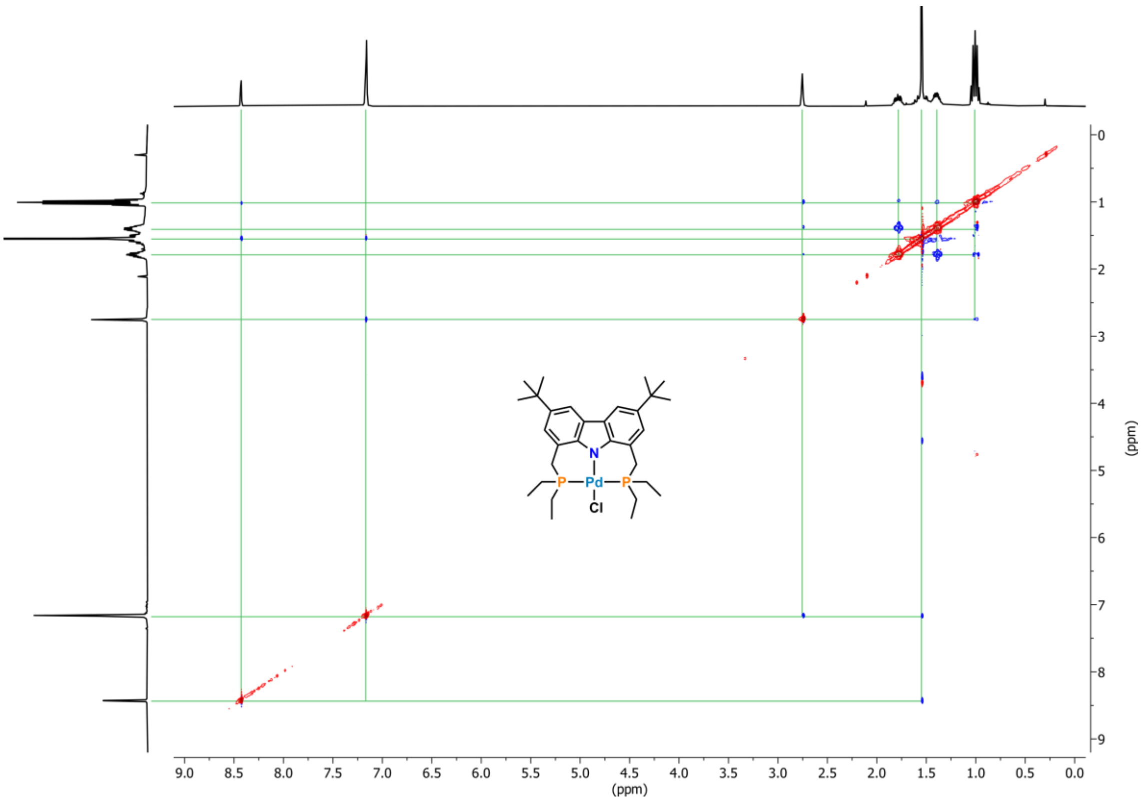

Figure S59. $^{1}H/^{1}H$ NOESY NMR spectrum (C_6D_6, 600/600 MHz, 295 K) of **[(^{Et}PNP)PdCl] (2^{Et}-Pd)**.

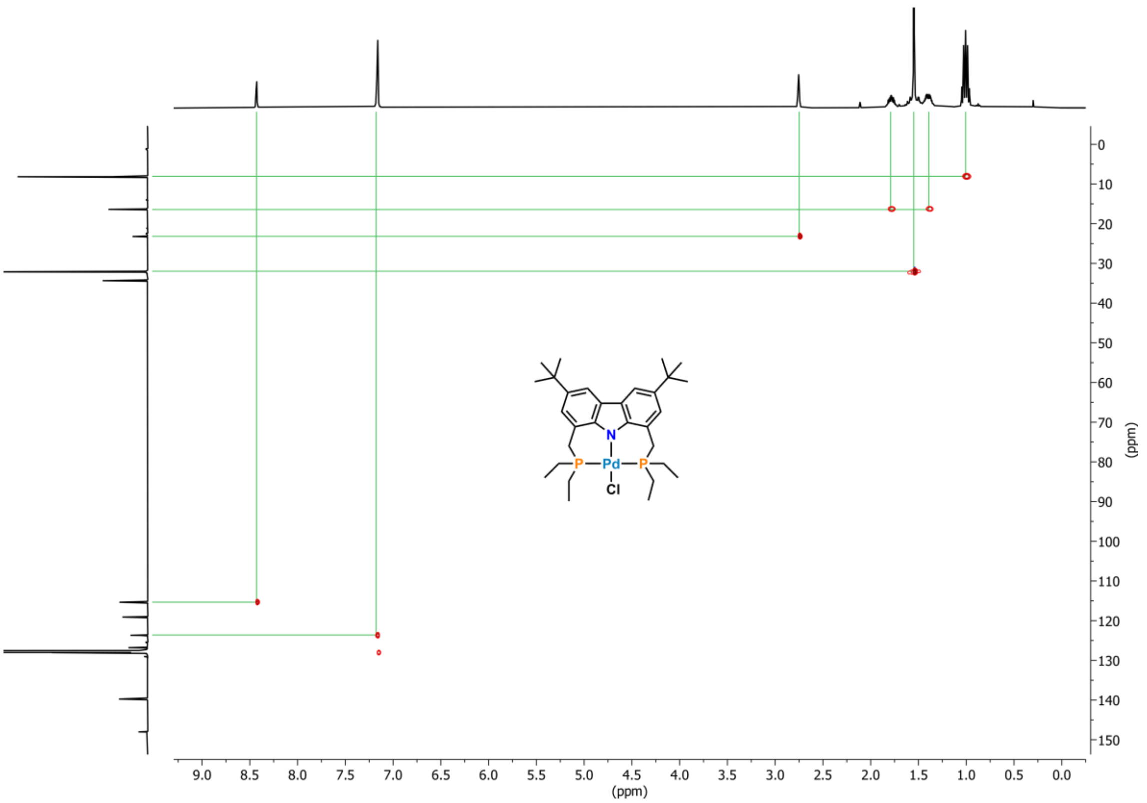

Figure S60. $^{1}H/^{13}C$ HSQC NMR spectrum (C_6D_6, 600/151 MHz, 295 K) of **[(^{Et}PNP)PdCl] (2^{Et}-Pd)**.

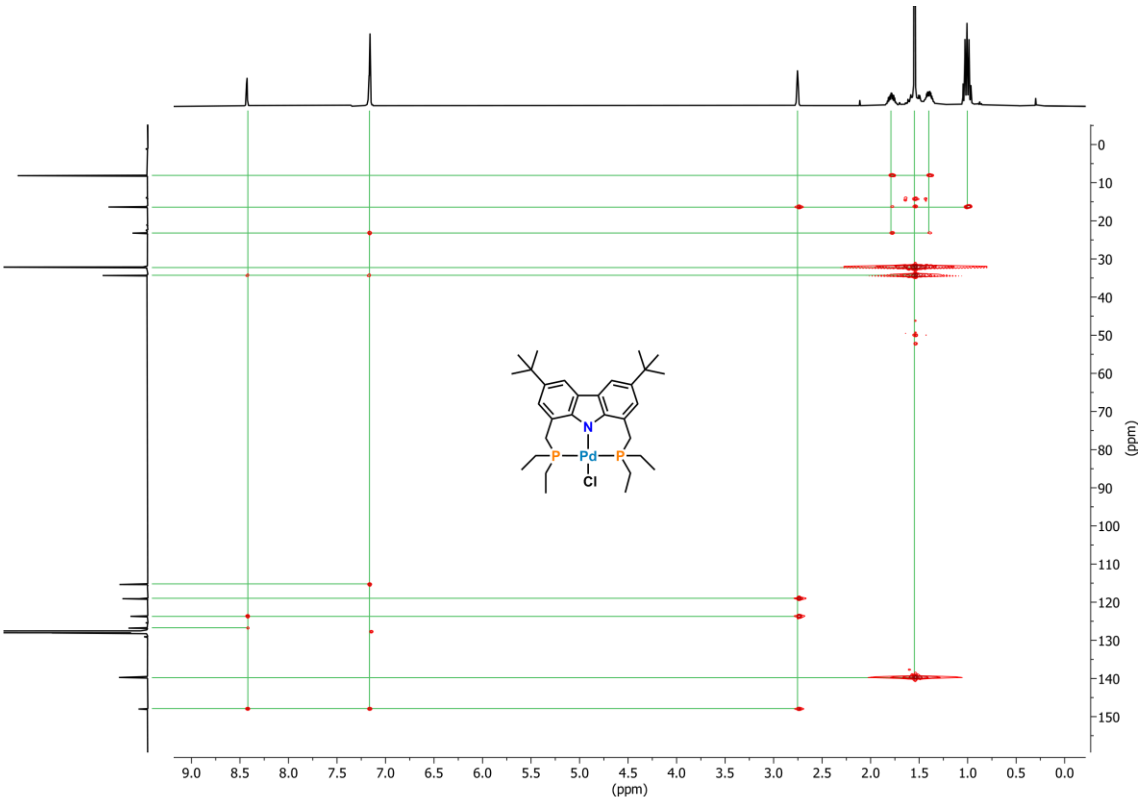

Figure S61. $^{1}H/^{13}C$ HMBC NMR spectrum (C_6D_6, 600/151 MHz, 295 K) of **[(^{Et}PNP)PdCl] (2^{Et}-Pd)**.

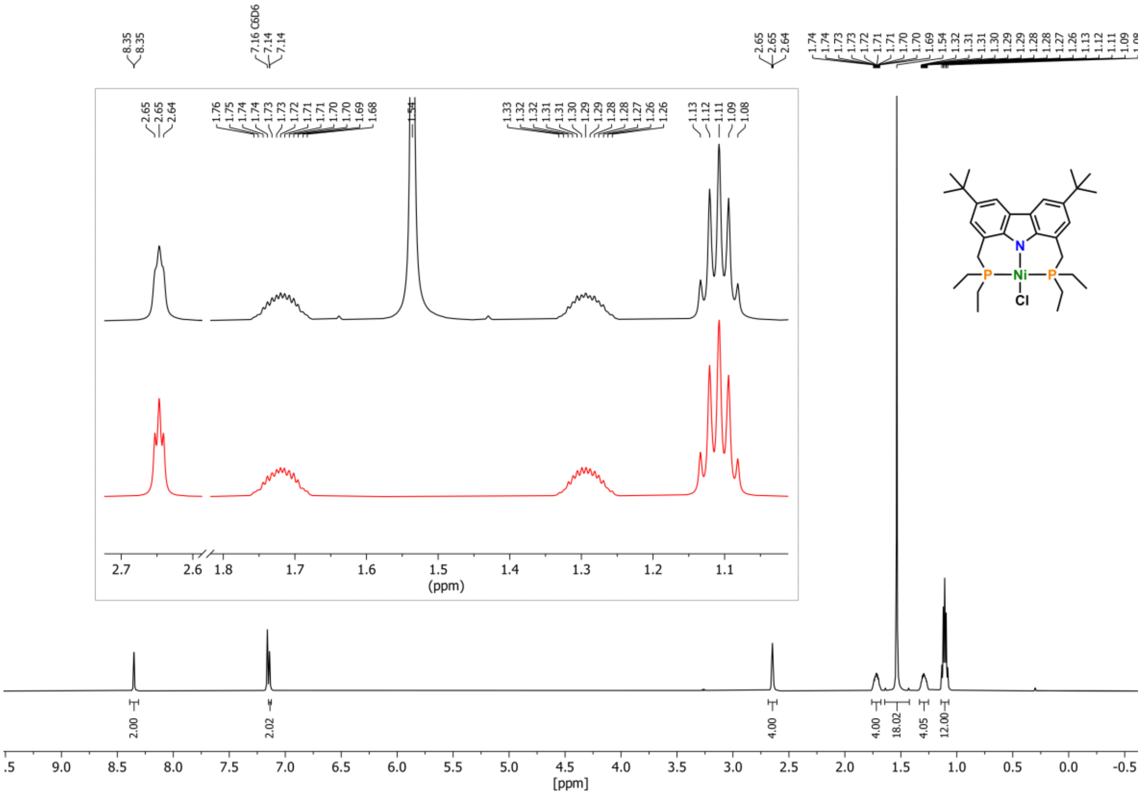

Figure S62. ^{1}H NMR spectrum (C_6D_6, 600 MHz, 295 K) of **[(^{Et}PNP)NiCl] (2^{Et}-Ni)**. Simulated multiplets are depicted in red.

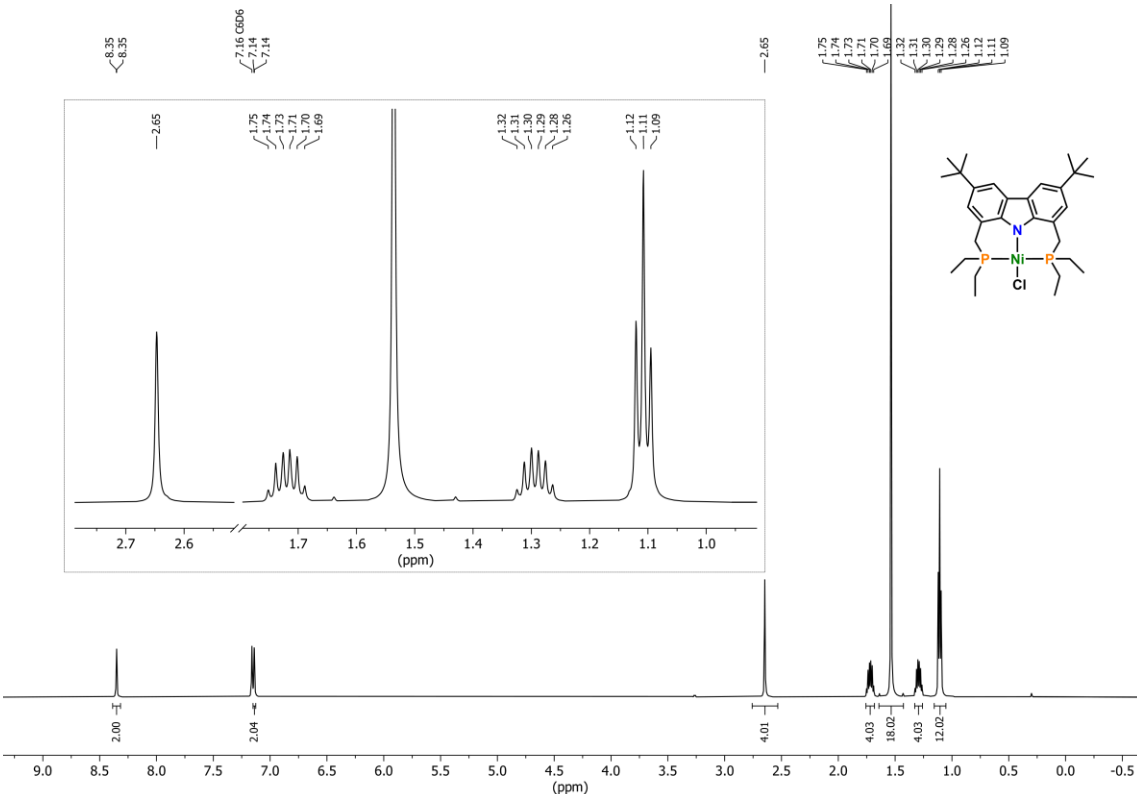

Figure S63. $^{1}H\{^{31}P\}$ NMR spectrum (C_6D_6, 600 MHz, 295 K) of **[(^{Et}PNP)NiCl] (2^{Et}-Ni)**.

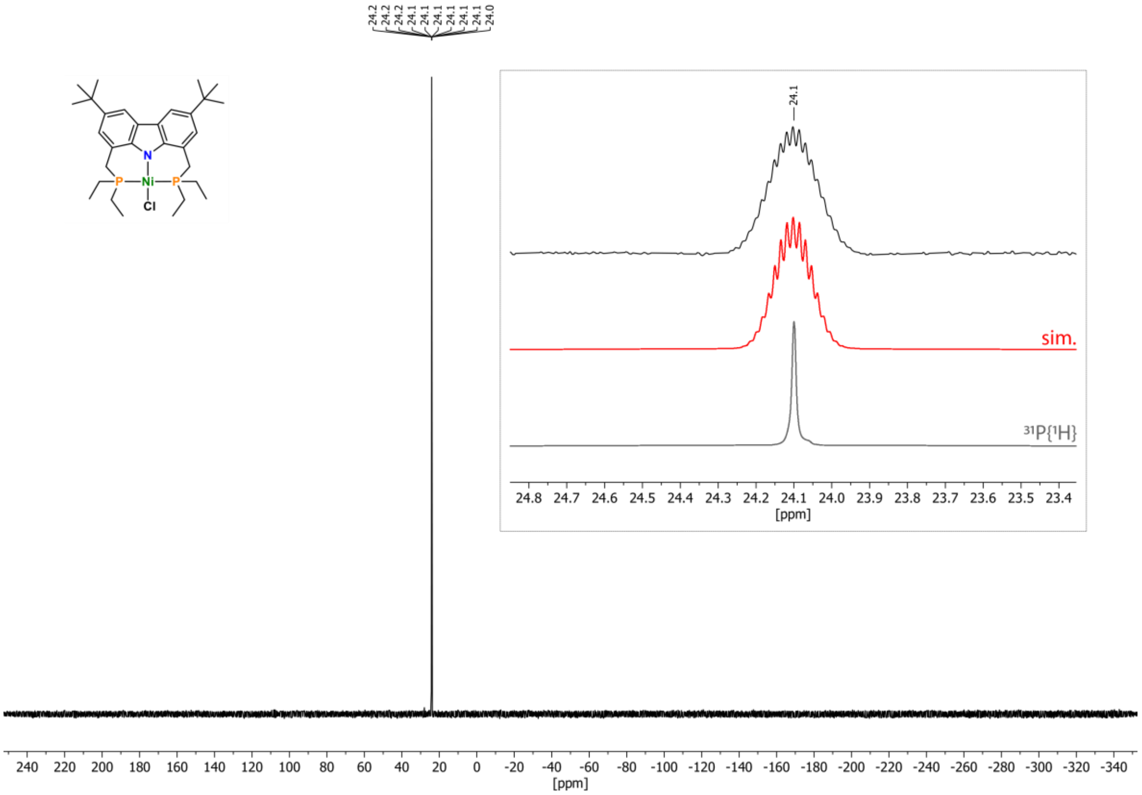

Figure S64. ^{31}P and $^{31}P\{^{1}H\}$ NMR spectrum (C_6D_6, 243 MHz, 295K) of **[(^{Et}PNP)NiCl] (2^{Et}-Ni)**. Inset with simulated spectrum in red.

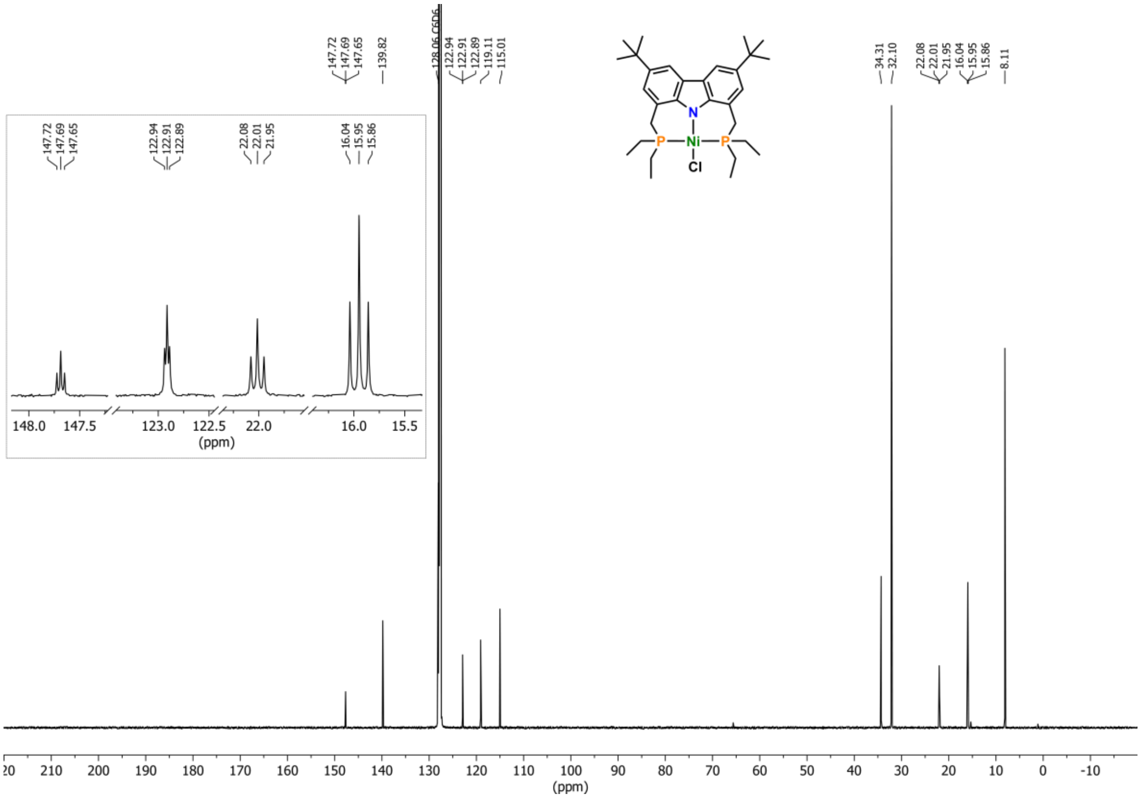

Figure S65. $^{13}C\{^{1}H\}$ NMR spectrum (C_6D_6, 151 MHz, 295 K) of **[(^{Et}PNP)NiCl] (2^{Et}-Ni)**.

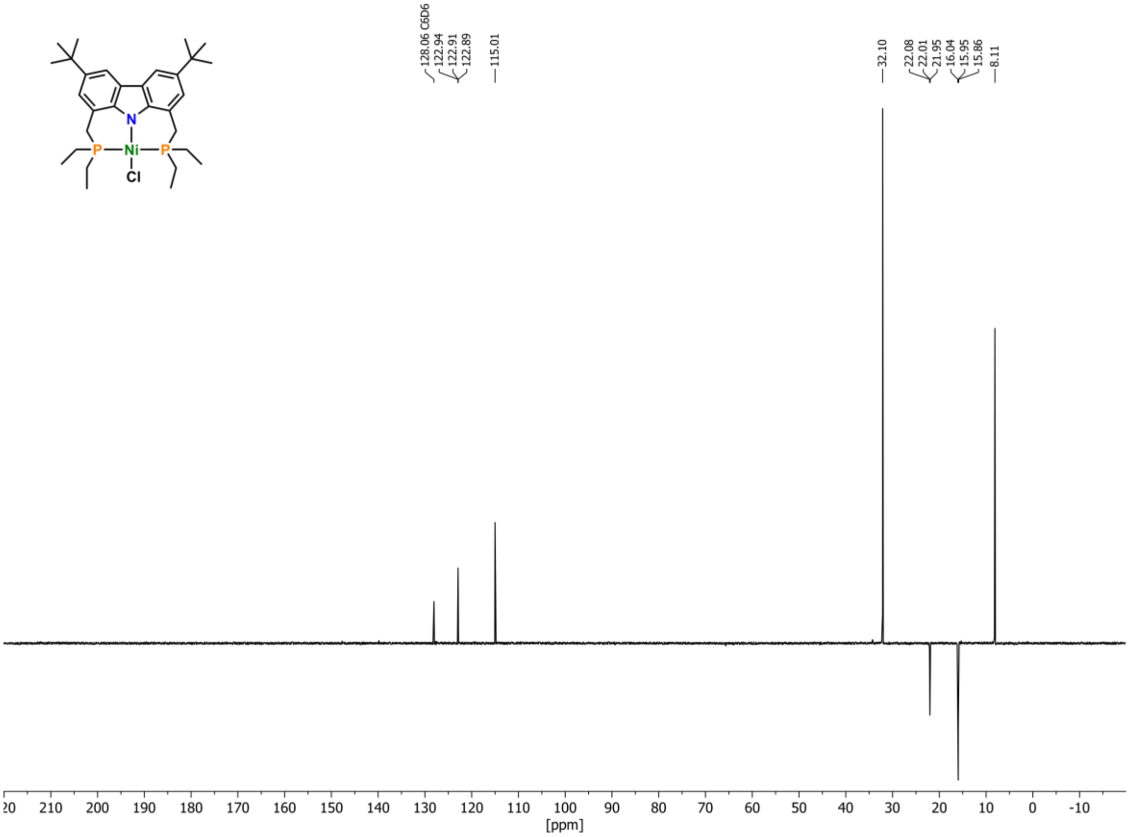

Figure S66. $^{13}C\{^{1}H\}$ DEPT-135 NMR spectrum (C_6D_6, 151 MHz, 295 K) of **[(^{iEt}PNP)NiCl] (2^{Et}-Ni)**.

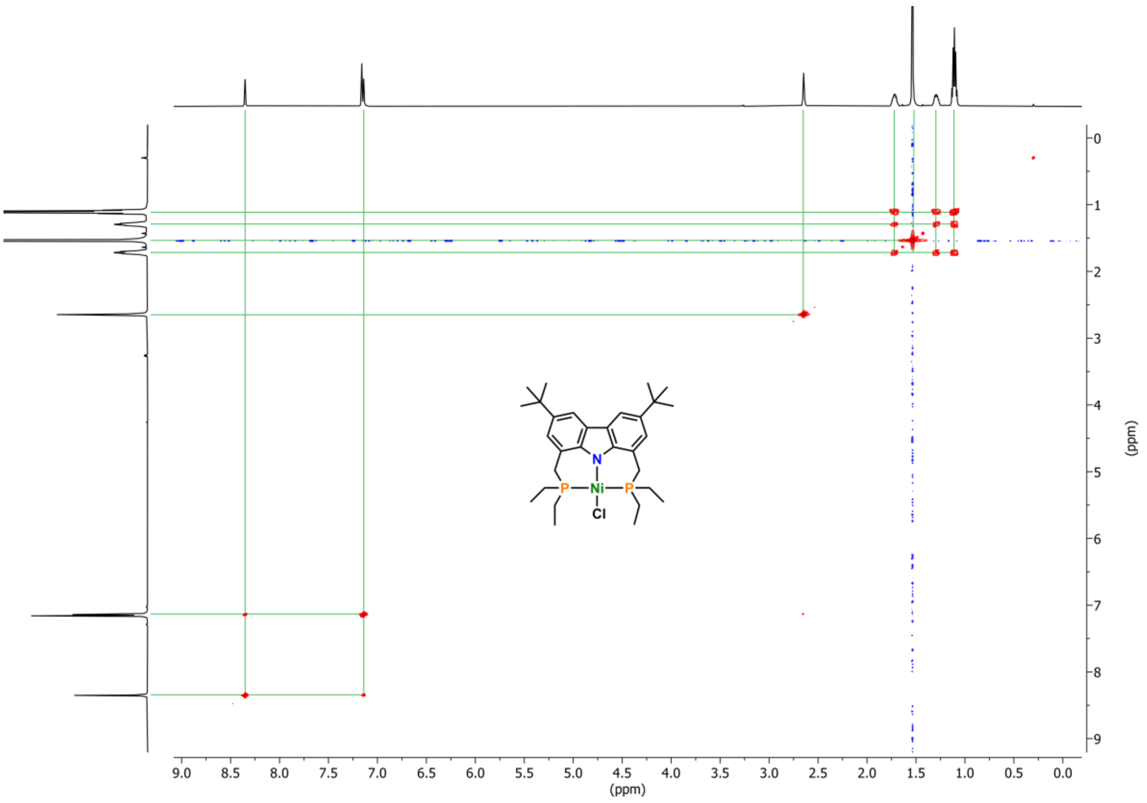

Figure S67. $^1H/^1H$ COSY NMR spectrum (C_6D_6, 600/600 MHz, 295 K) of **[(^{Et}PNP)NiCl] (2^{Et}-Ni)**.

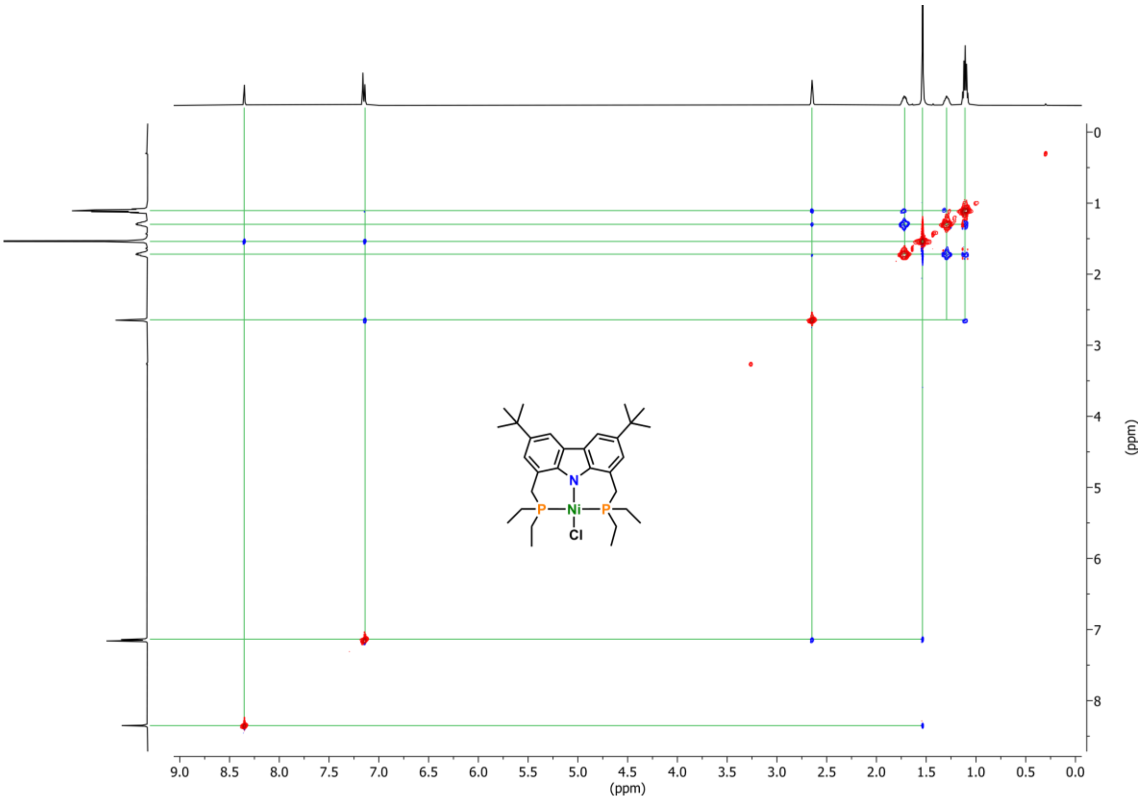

Figure S68. $^1H/^1H$ NOESY NMR spectrum (C_6D_6, 600/600 MHz, 295 K) of **[(^{Et}PNP)NiCl] (2^{Et}-Ni)**.

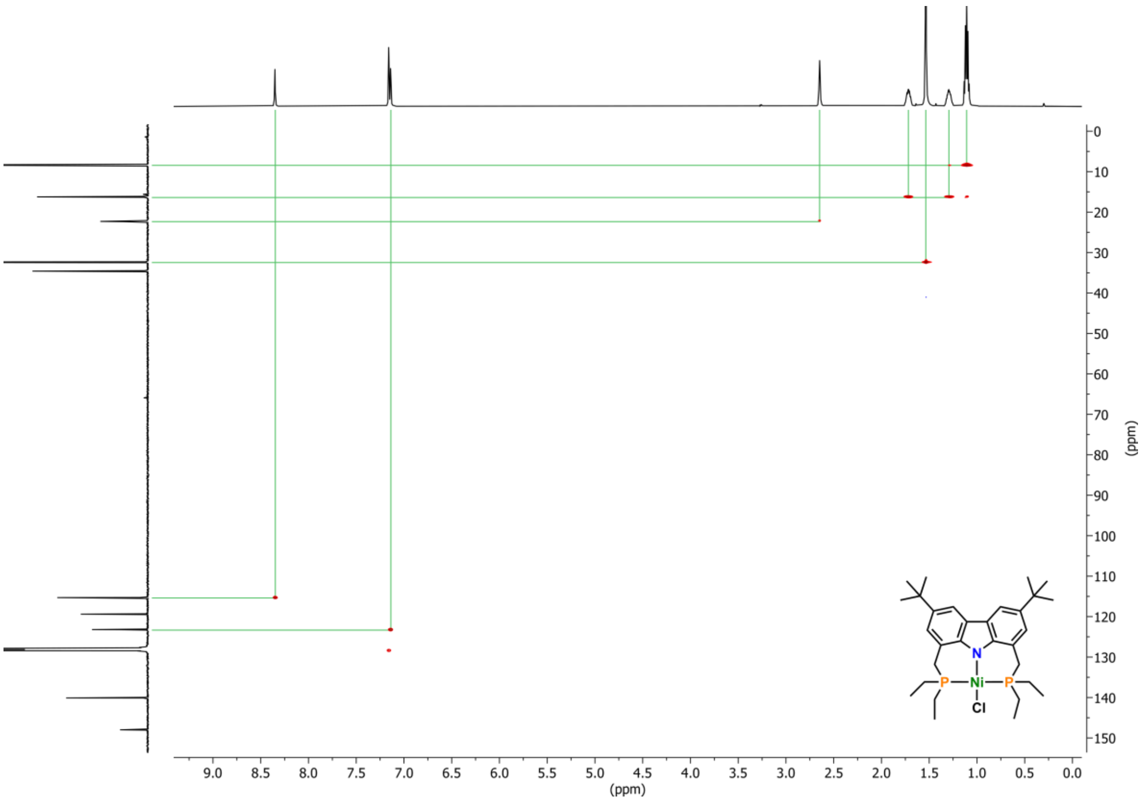

Figure S69. $^1H/^{13}C$ HSQC NMR spectrum (C_6D_6, 600/151 MHz, 295 K) of **[(^{Et}PNP)NiCl] (2^{Et}-Ni)**.

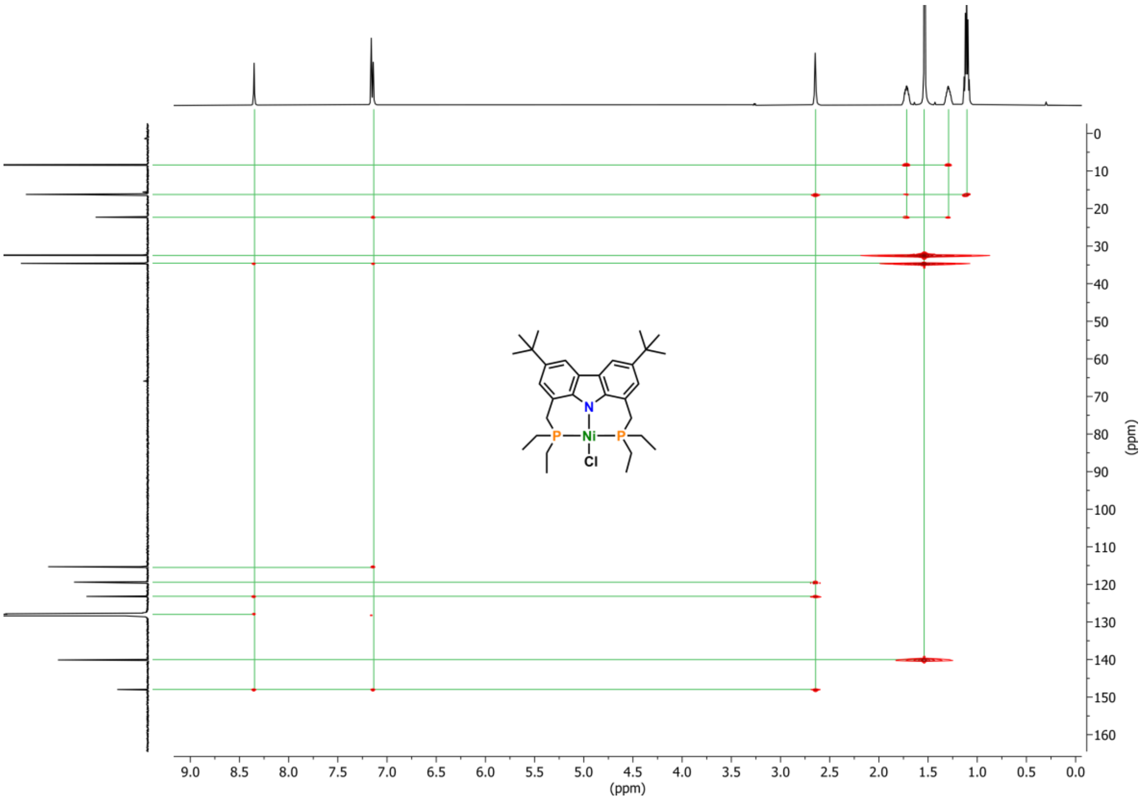

Figure S70. $^1H/^{13}C$ HMBC NMR spectrum (C_6D_6, 600/151 MHz, 295 K) of **[(^{Et}PNP)NiCl] (2^{Et}-Ni)**.

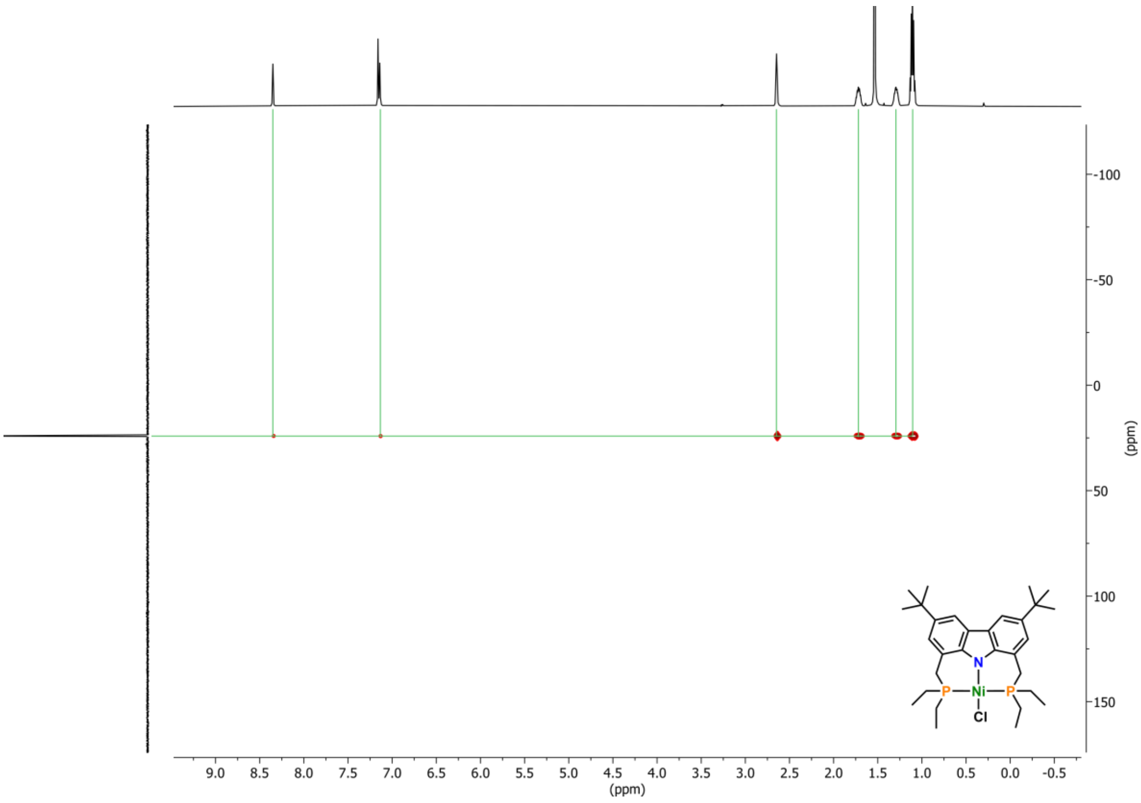

Figure S71. $^1H/^{31}P$ HMBC NMR spectrum (C_6D_6, 600/243 MHz, 295 K) of **[(^{Et}PNP)NiCl] (2^{Et}-Ni)**.

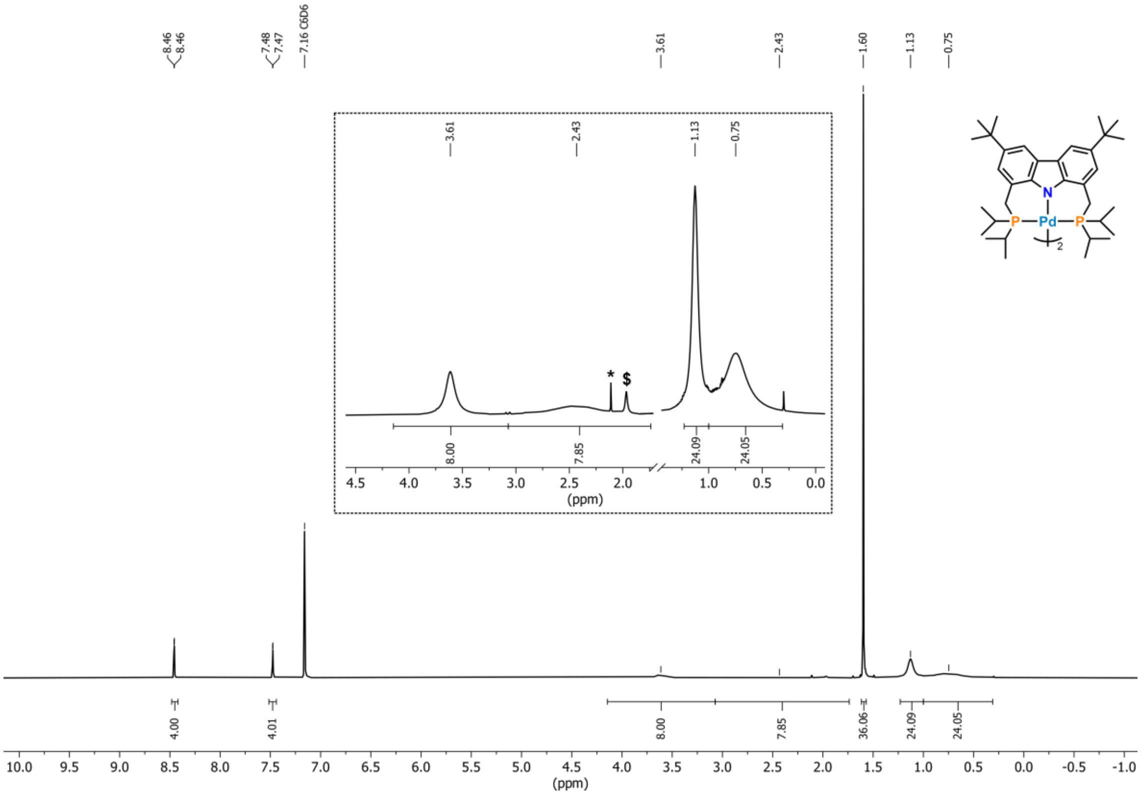

Figure S72. ^1H NMR spectrum (C_6D_6, 600 MHz, 295 K) of **[(^{iPr}PNP)Pd]$_2$ (4^{iPr}-Pd)** (* = toluene, $ = **3^{iPr}-Pd**).

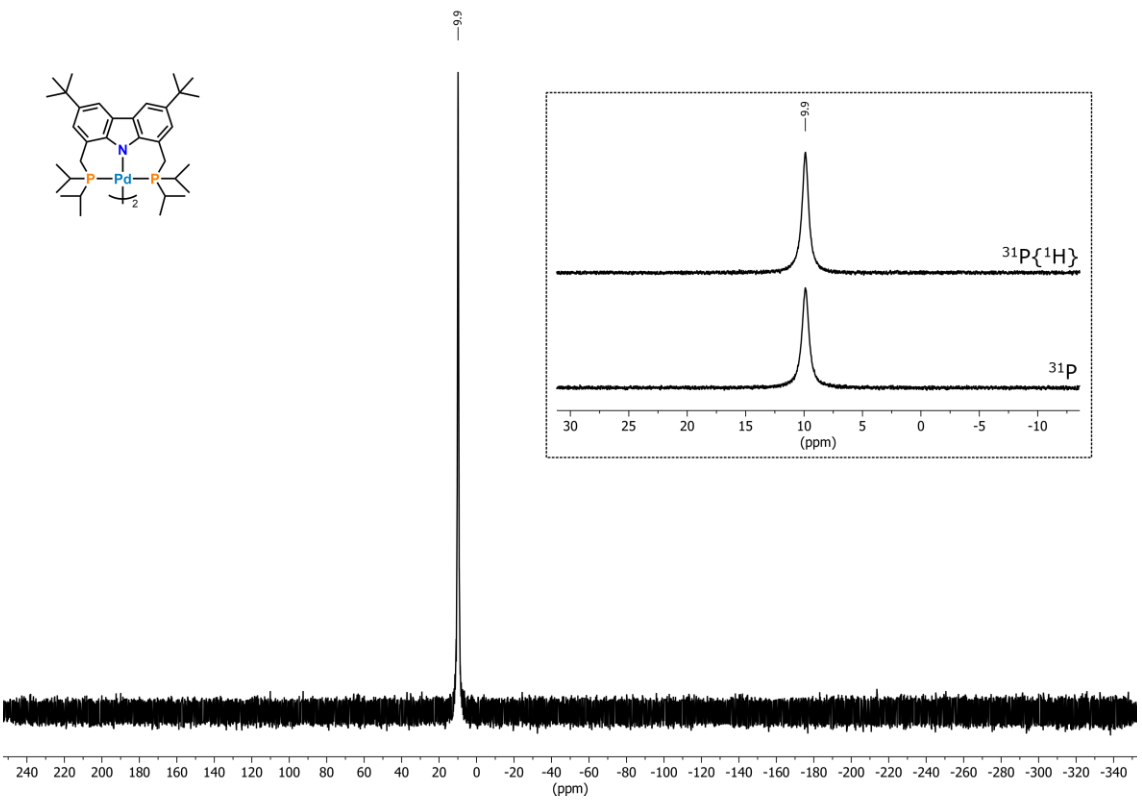

Figure S73. ^{31}P and $^{31}P\{^1H\}$ NMR spectrum (C_6D_6, 243 MHz, 295K) of **[(^{iPr}PNP)Pd]$_2$ (4^{iPr}-Pd)**.

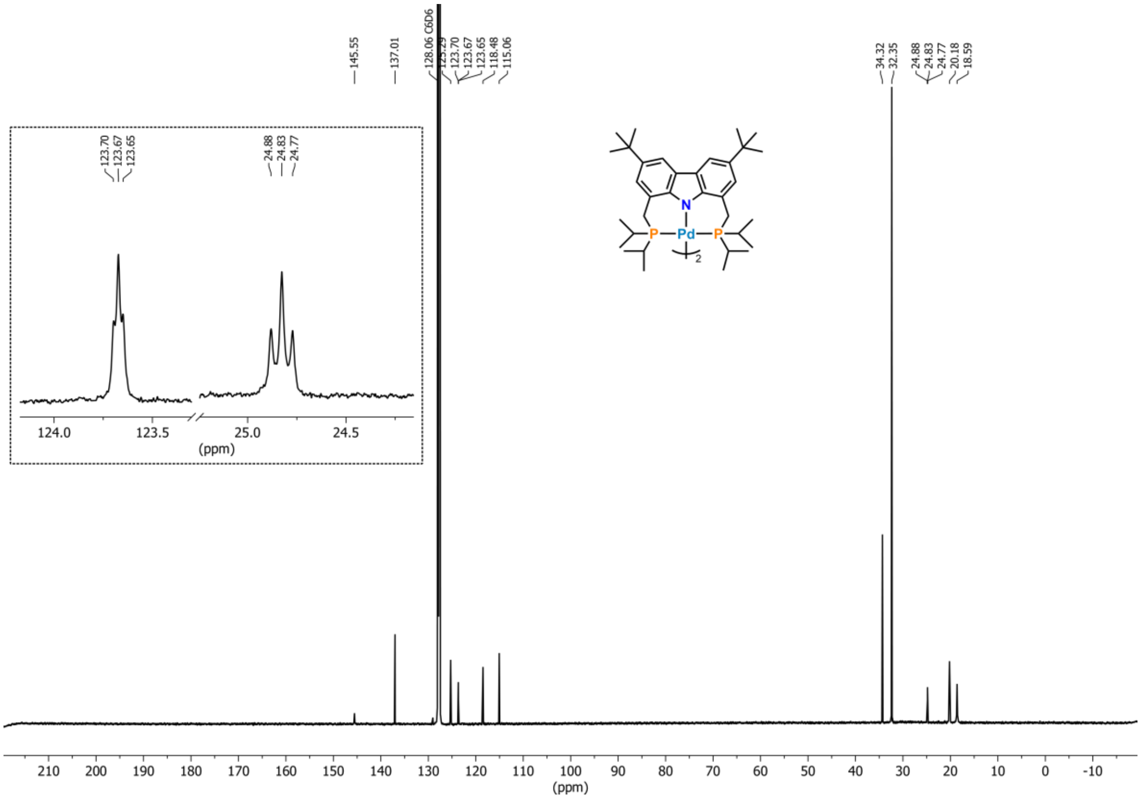

Figure S74. $^{13}C\{^1H\}$ NMR spectrum (C_6D_6, 151 MHz, 295 K) of **[(^{iPr}PNP)Pd]$_2$ (4^{iPr}-Pd)**.

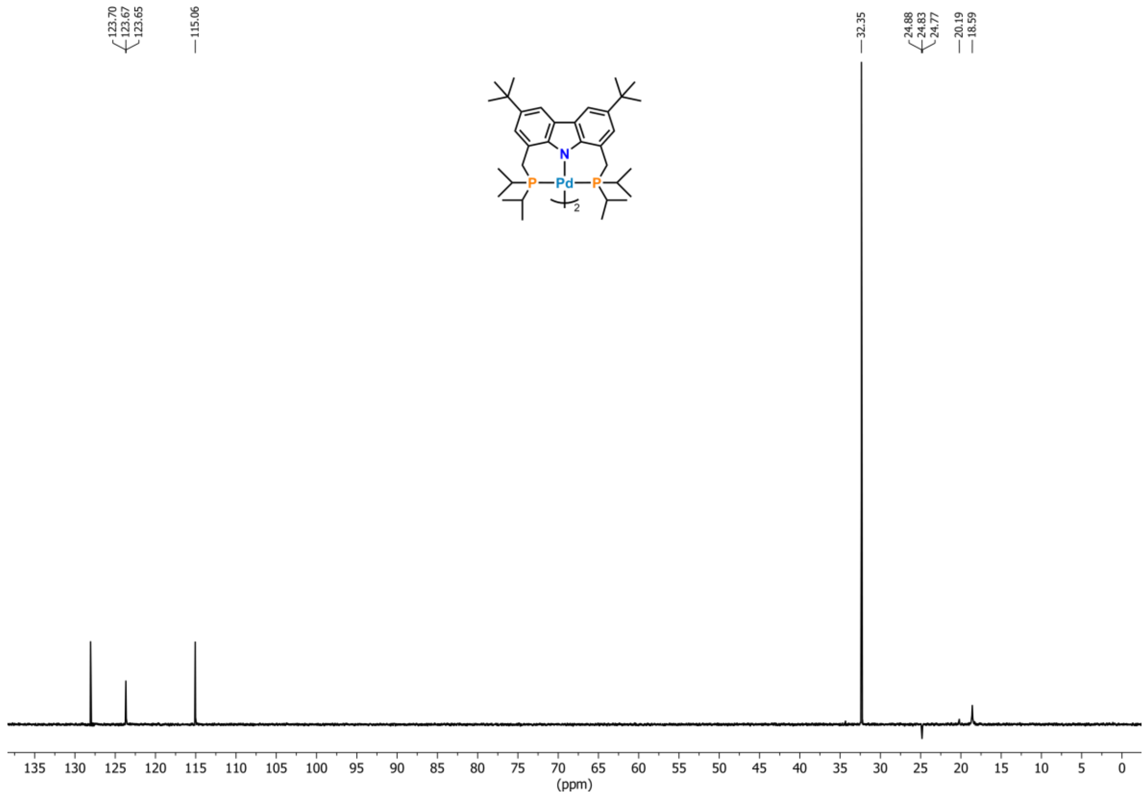

Figure S75. $^{13}C\{^{1}H\}$ DEPT-135 NMR spectrum (C_6D_6, 151 MHz, 295 K) of **[(^{iPr}PNP)Pd]$_2$ (4^{iPr}-Pd)**.

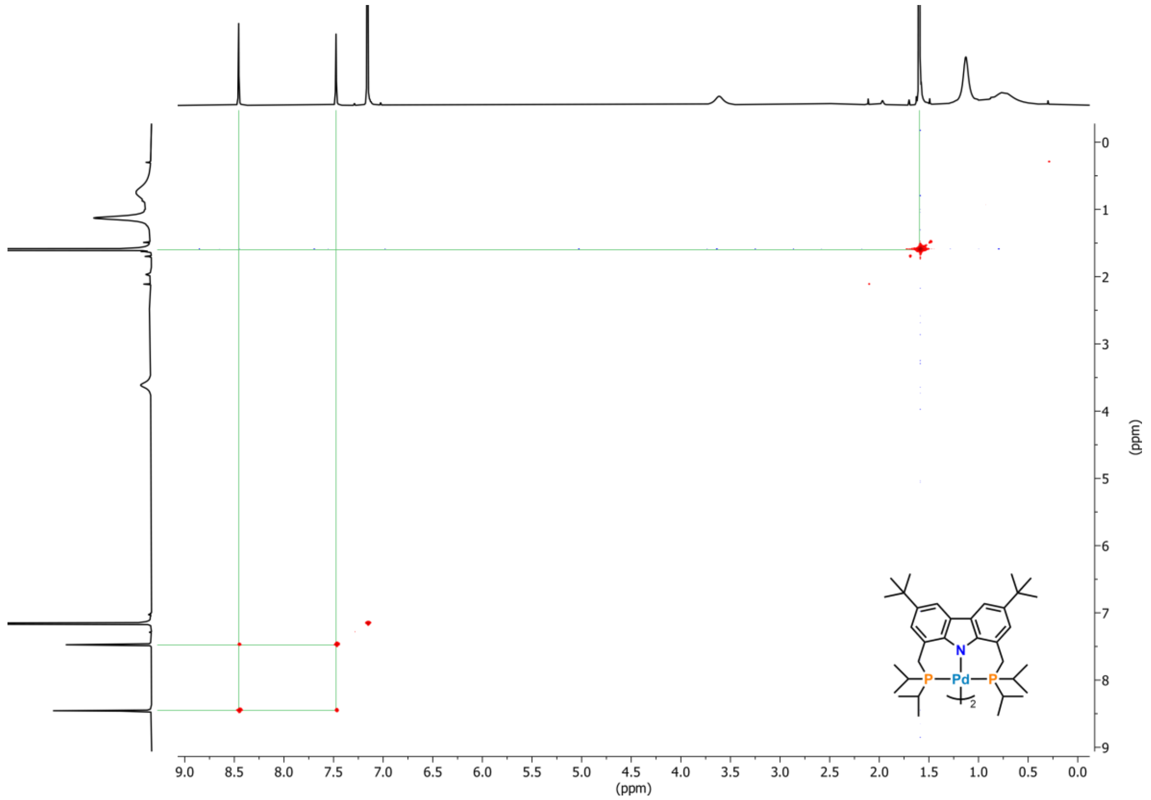

Figure S76. $^{1}H/^{1}H$ COSY NMR spectrum (C_6D_6, 600/600 MHz, 295 K) of **[(^{iPr}PNP)Pd]$_2$ (4^{iPr}-Pd)**.

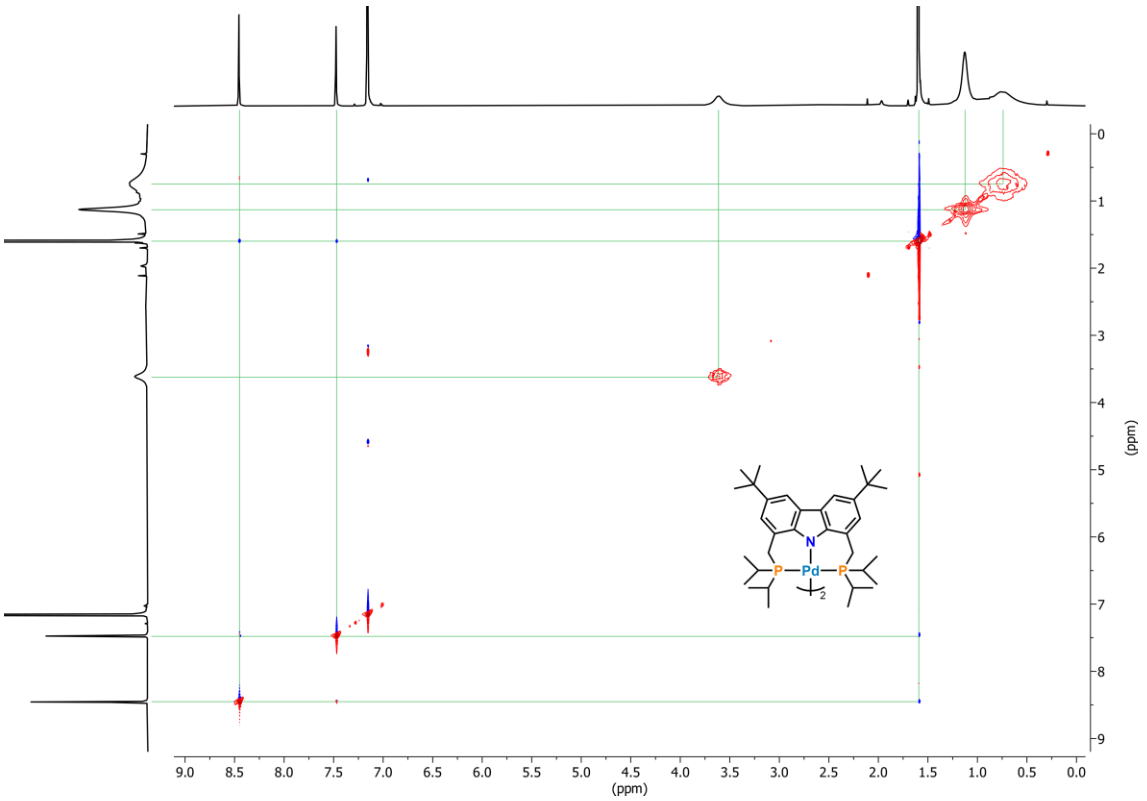

Figure S77. $^{1}H/^{1}H$ NOESY NMR spectrum (C_6D_6, 600/600 MHz, 295 K) of **[(^{iPr}PNP)Pd]$_2$ (4^{iPr}-Pd)**.

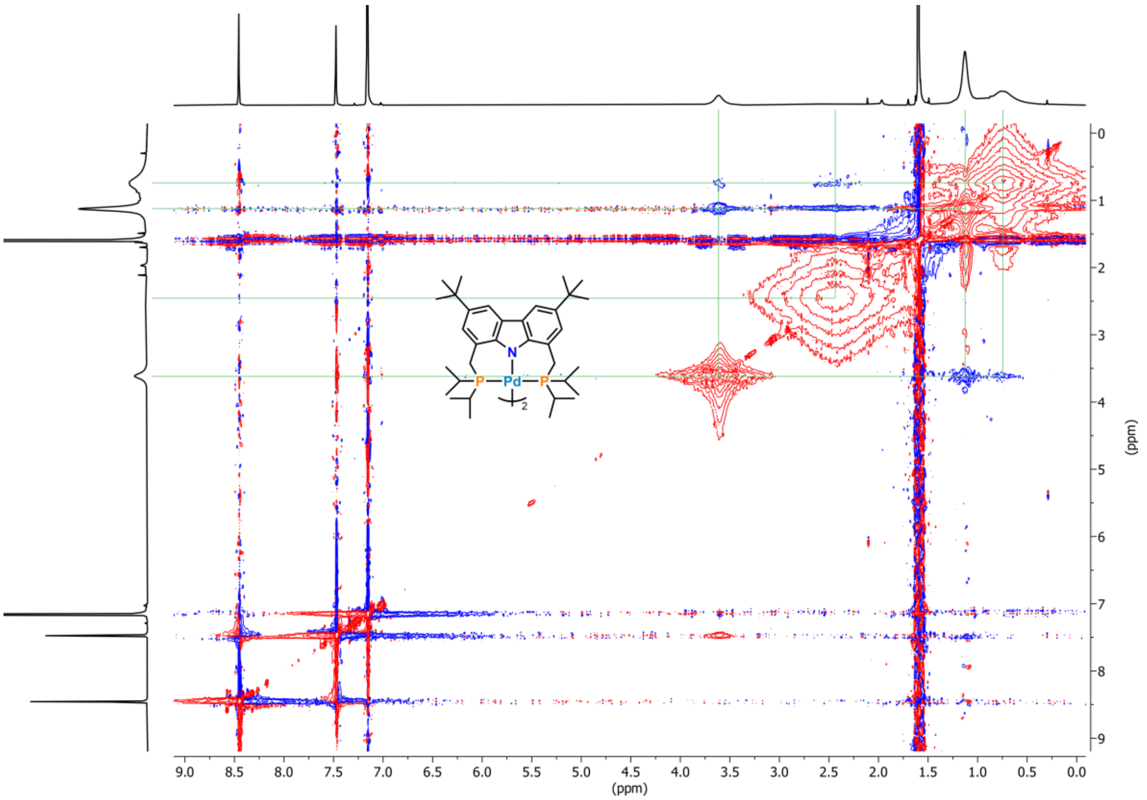

Figure S78. Amplified $^{1}H/^{1}H$ NOESY NMR spectrum (C_6D_6, 600/600 MHz, 295 K) of **[(^{iPr}PNP)Pd]$_2$ (4^{iPr}-Pd)**.

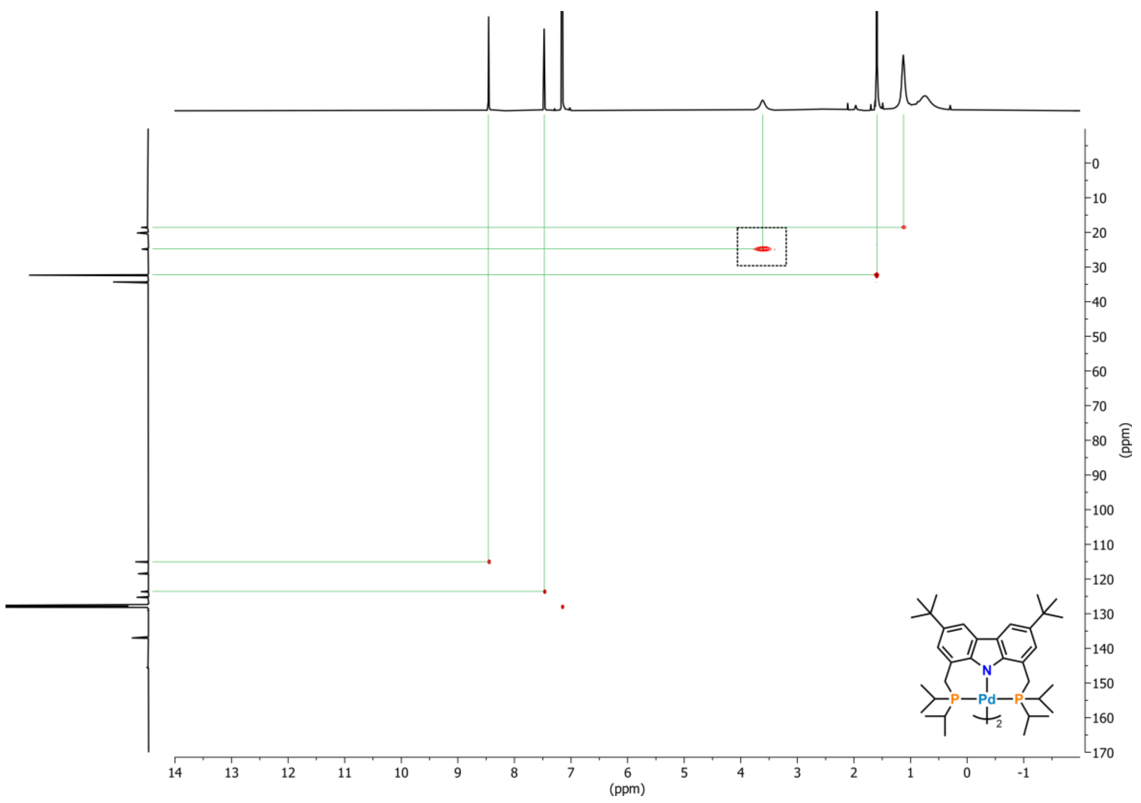

Figure S79. $^1H/^{13}C$ HSQC NMR spectrum (C_6D_6, 600/151 MHz, 295 K) of **[(^{iPr}PNP)Pd]$_2$ (4^{iPr}-Pd)**. Due to a large dynamic range, the weaker signal was amplified in the inset.

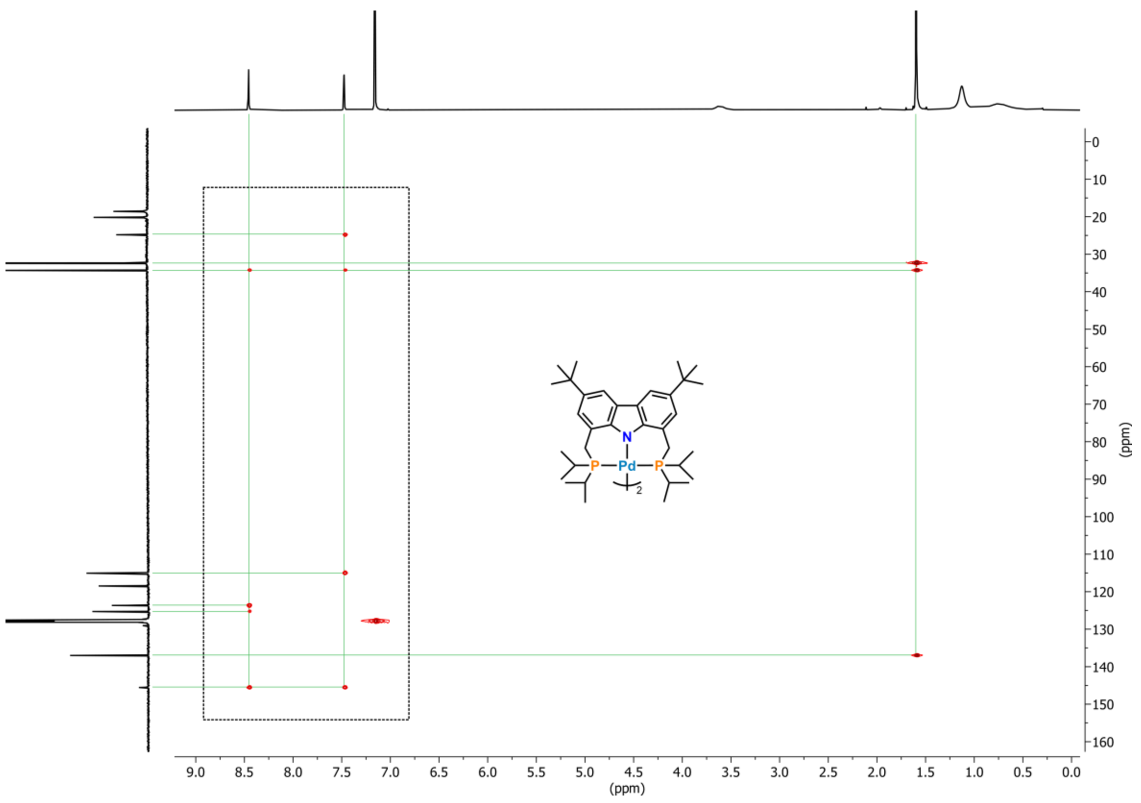

Figure S80. $^1H/^{13}C$ HMBC NMR spectrum (C_6D_6, 600/151 MHz, 295 K) of **[(^{iPr}PNP)Pd]$_2$ (4^{iPr}-Pd)**. Due to a large dynamic range, the weaker signals were amplified in the inset.

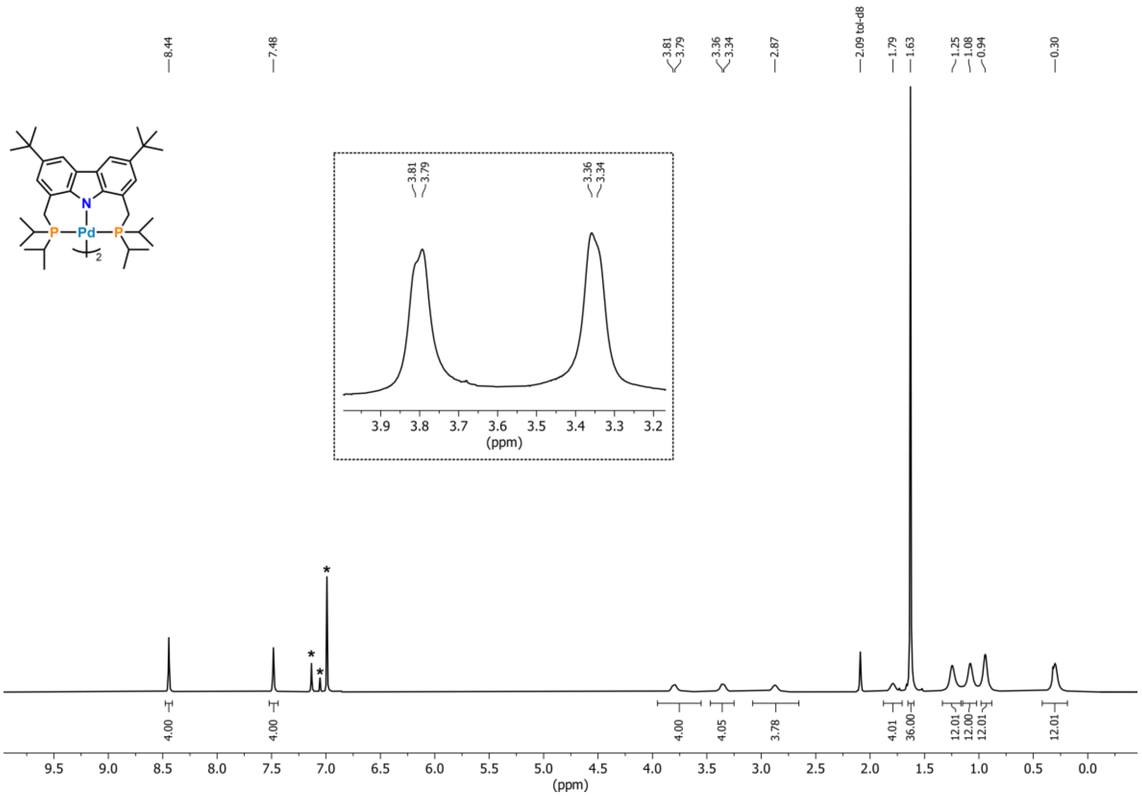

Figure S81. ^{1}H NMR spectrum (tol-d_8, 600 MHz, 243 K) of **[(^{iPr}PNP)Pd]$_2$ (4^{iPr}-Pd)** (* = tol-d_8).

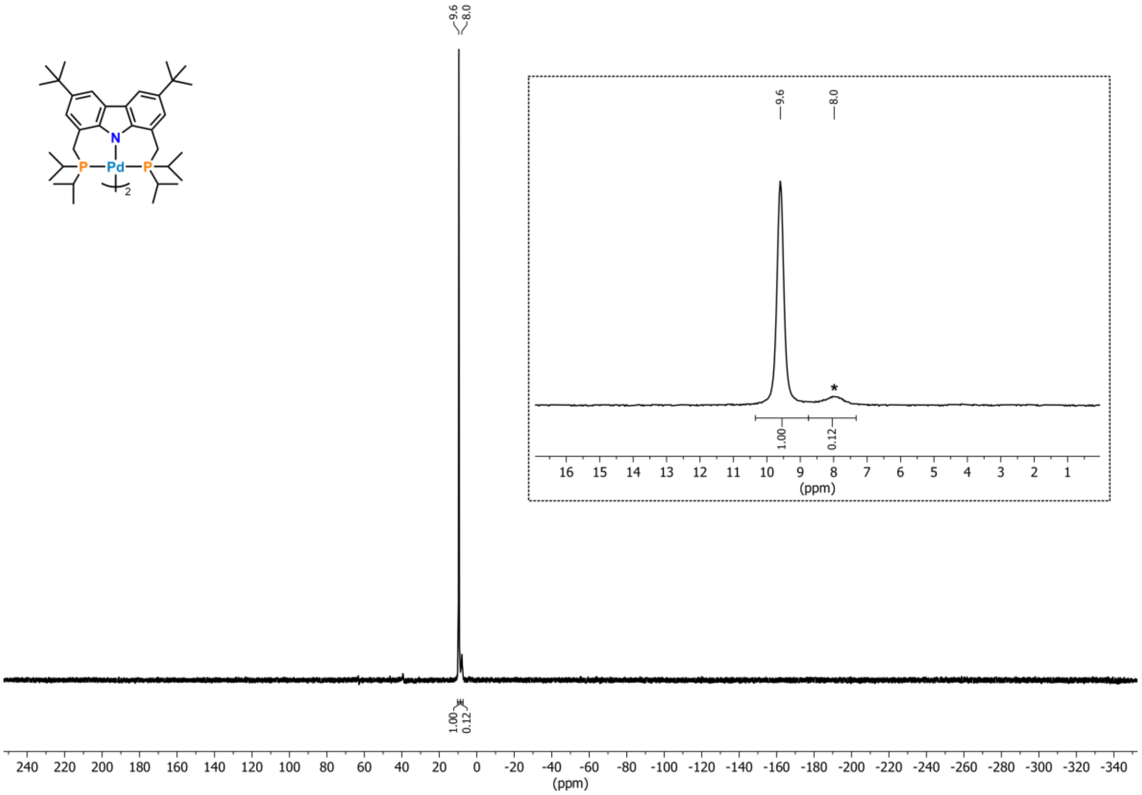

Figure S82. ^{31}P and $^{31}P\{^{1}H\}$ NMR spectrum (tol-d_8, 243 MHz, 243K) of **[(^{iPr}PNP)Pd]$_2$ (4^{iPr}-Pd)** (* = second isomer analog to **4''-Pd**, see main manuscript).

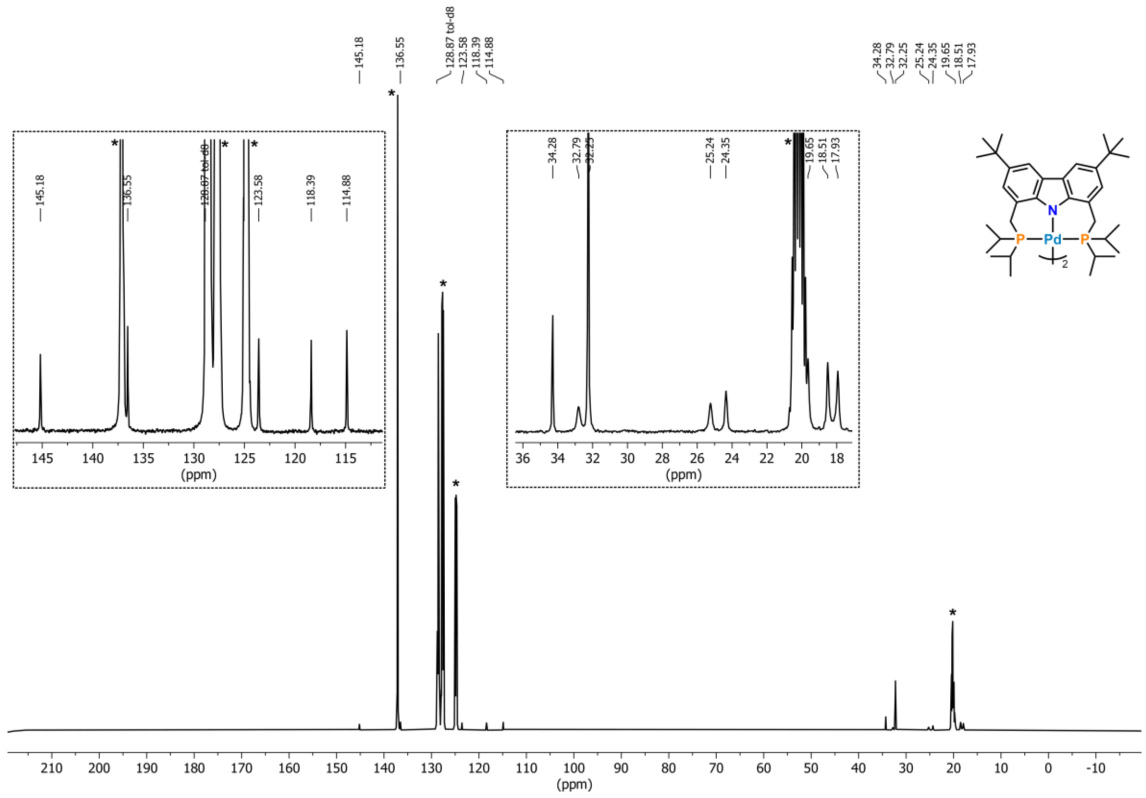

Figure S83. $^{13}C\{^{1}H\}$ NMR spectrum (tol-d_8, 151 MHz, 243 K) of **[(*^{iPr}*PNP)Pd]$_2$ (4^{iPr}-Pd)** (* = tol-d_8).

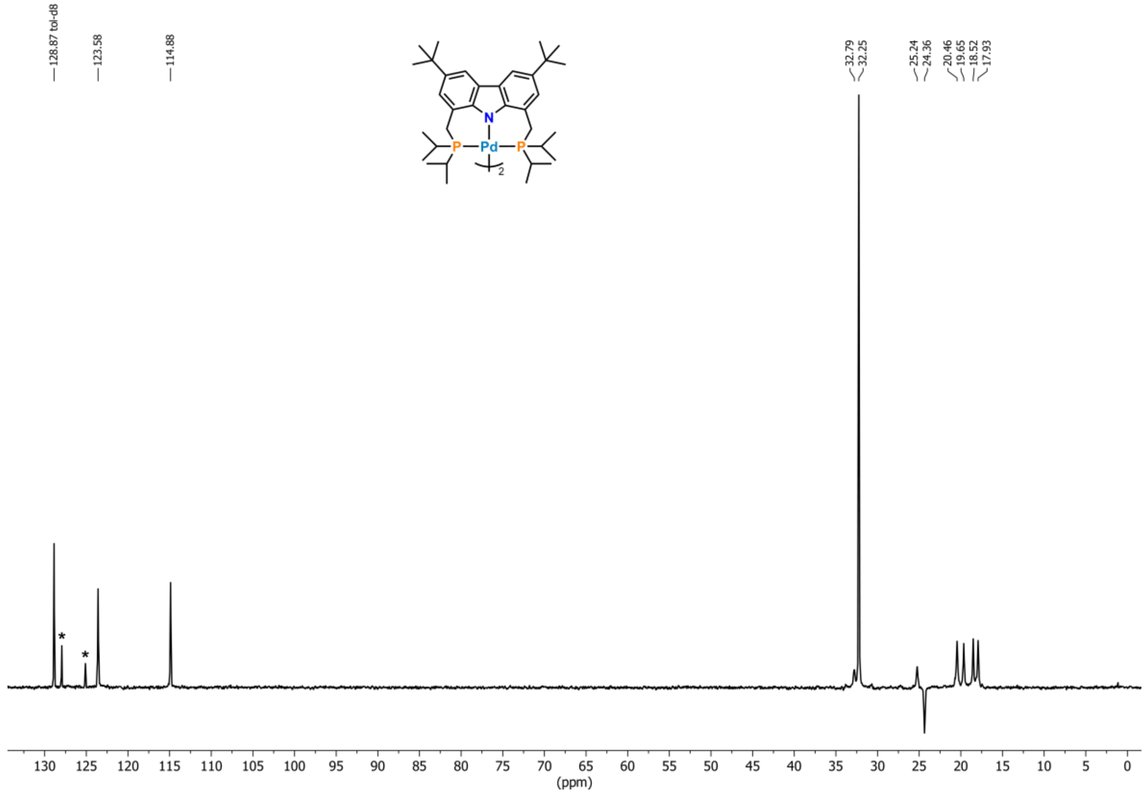

Figure S84. $^{13}C\{^{1}H\}$ DEPT-135 NMR spectrum (tol-d_8, 151 MHz, 243 K) of **[(*^{iPr}*PNP)Pd]$_2$ (4^{iPr}-Pd)** (* = tol-d_8).

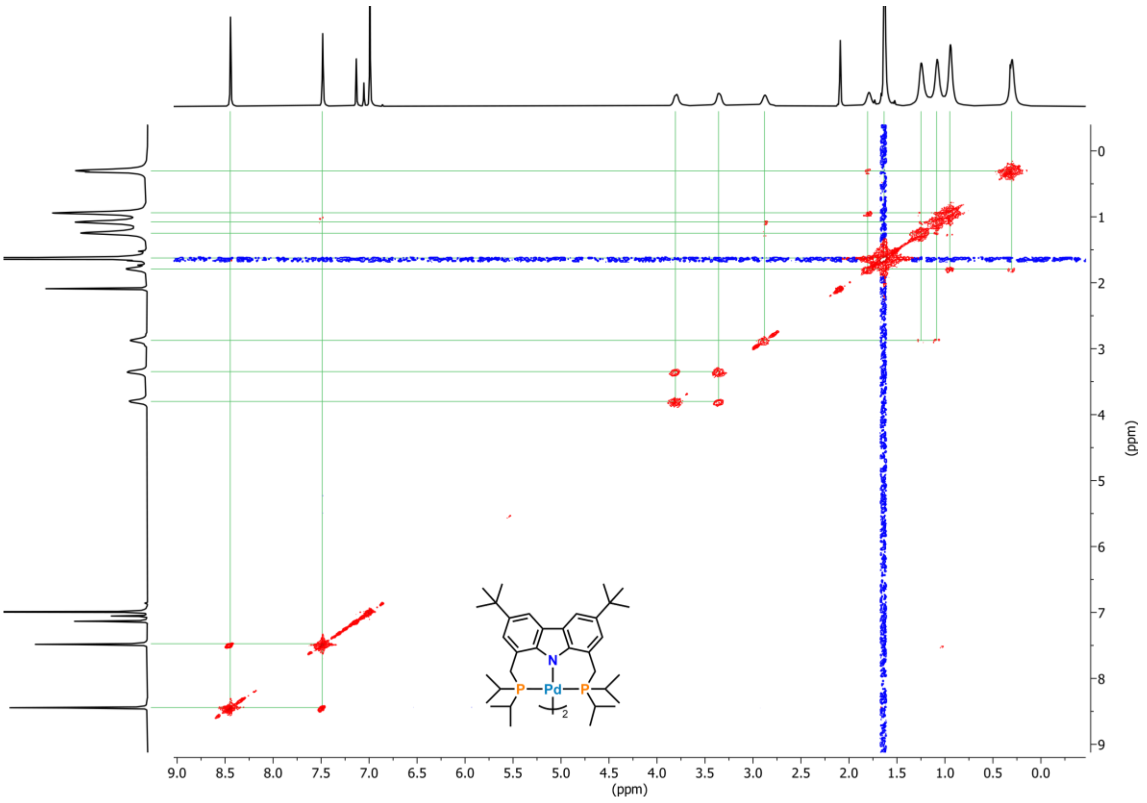

Figure S85. $^{1}H/^{1}H$ COSY NMR spectrum (tol-d_8, 600/600 MHz, 243 K) of **[(^{iPr}PNP)Pd]$_2$ (4^{iPr}-Pd)**.

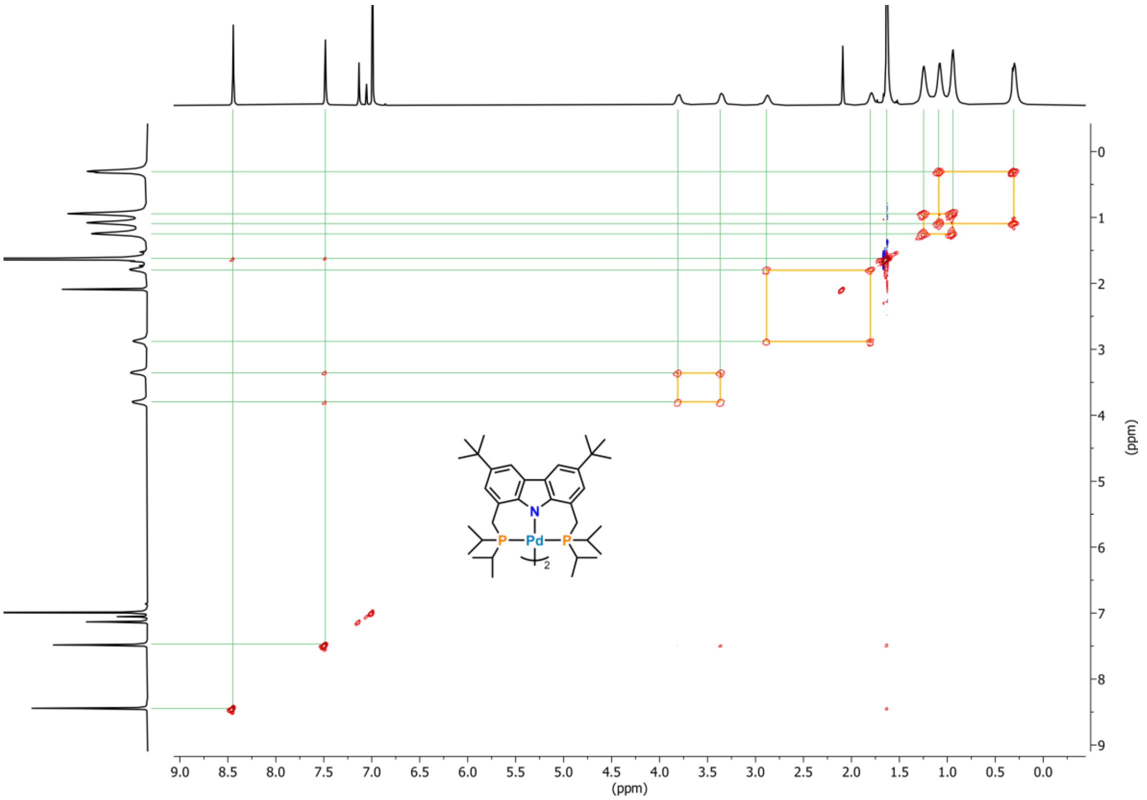

Figure S86. $^{1}H/^{1}H$ NOESY NMR spectrum (tol-d_8, 600/600 MHz, 243 K) of **[(^{iPr}PNP)Pd]$_2$ (4^{iPr}-Pd)** (in orange connected are exchange cross peaks).

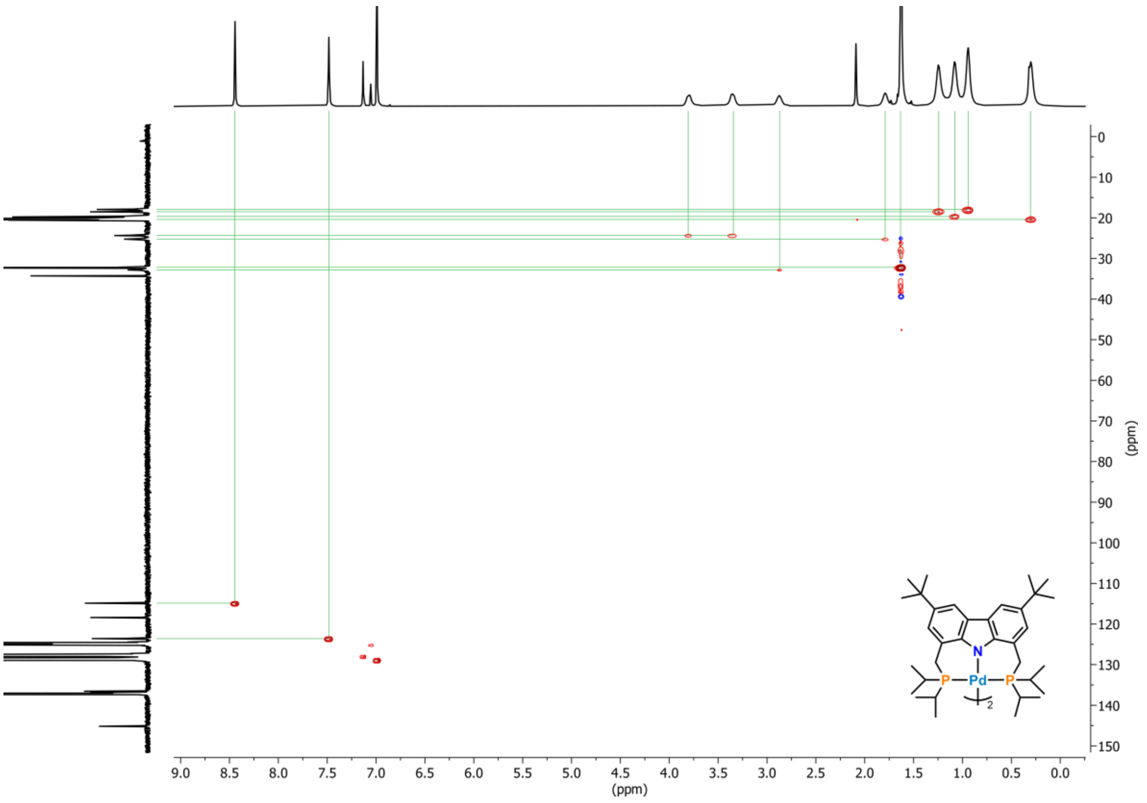

Figure S87. $^{1}H/^{13}C$ HSQC NMR spectrum (tol-d_8, 600/151 MHz, 243 K) of **[(^{iPr}PNP)Pd]$_2$ (4^{iPr}-Pd)**.

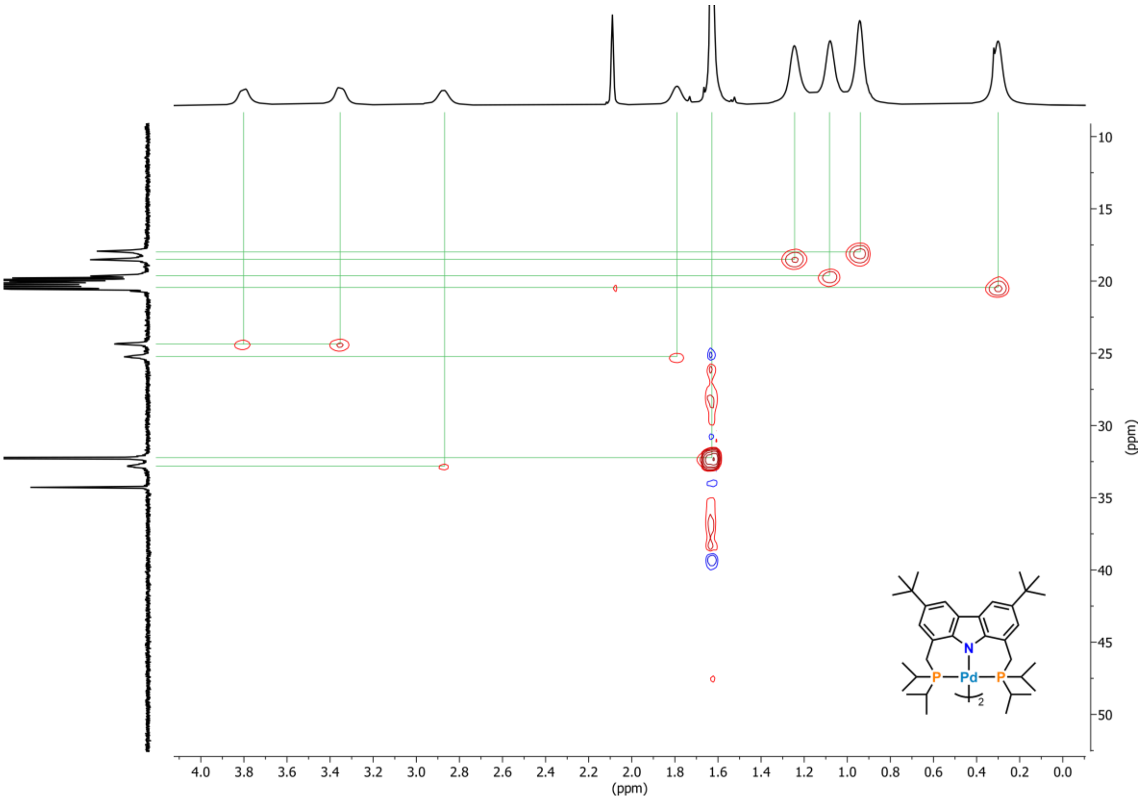

Figure S88. Selected region in the $^{1}H/^{13}C$ HSQC NMR spectrum (tol-d_8, 600/151 MHz, 243 K) of **[(^{iPr}PNP)Pd]$_2$ (4^{iPr}-Pd)** for clarity.

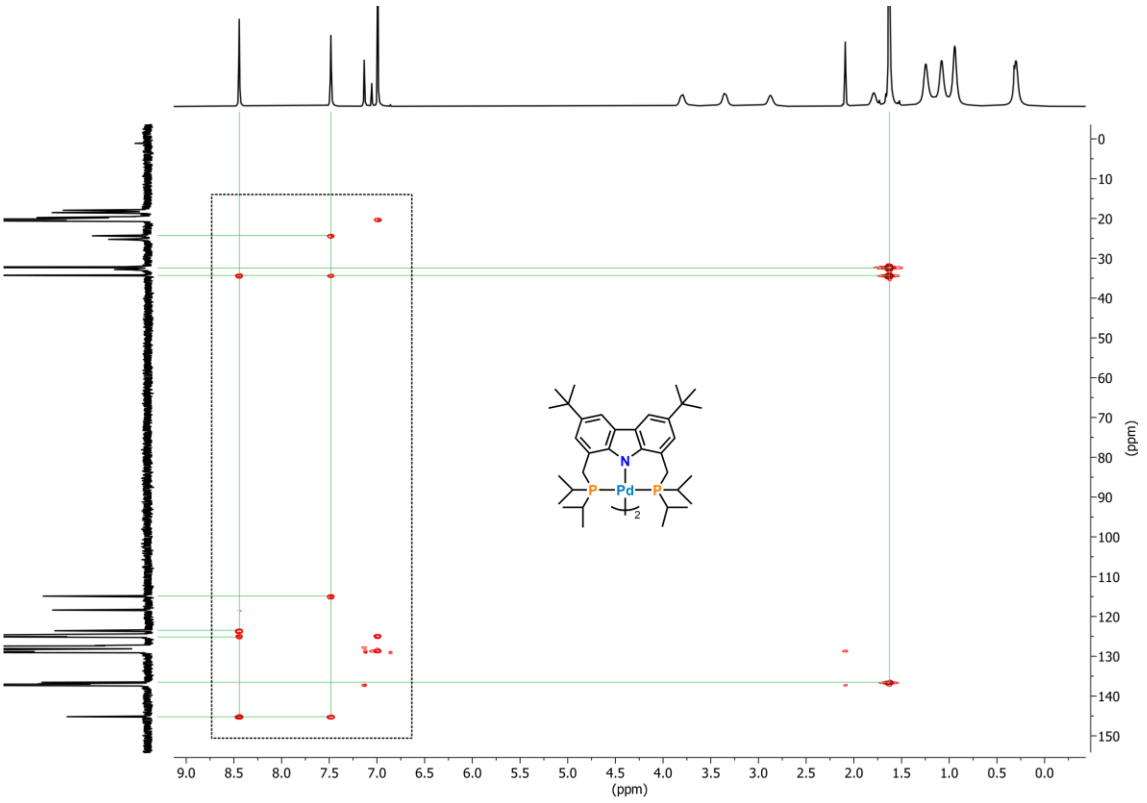

Figure S89. $^{1}H/^{13}C$ HMBC NMR spectrum (tol-d$_8$, 600/151 MHz, 243 K) of **[(^{iPr}PNP)Pd]$_2$ (4^{iPr}-Pd)**. Due to a large dynamic range, the weaker signals were amplified in the inset

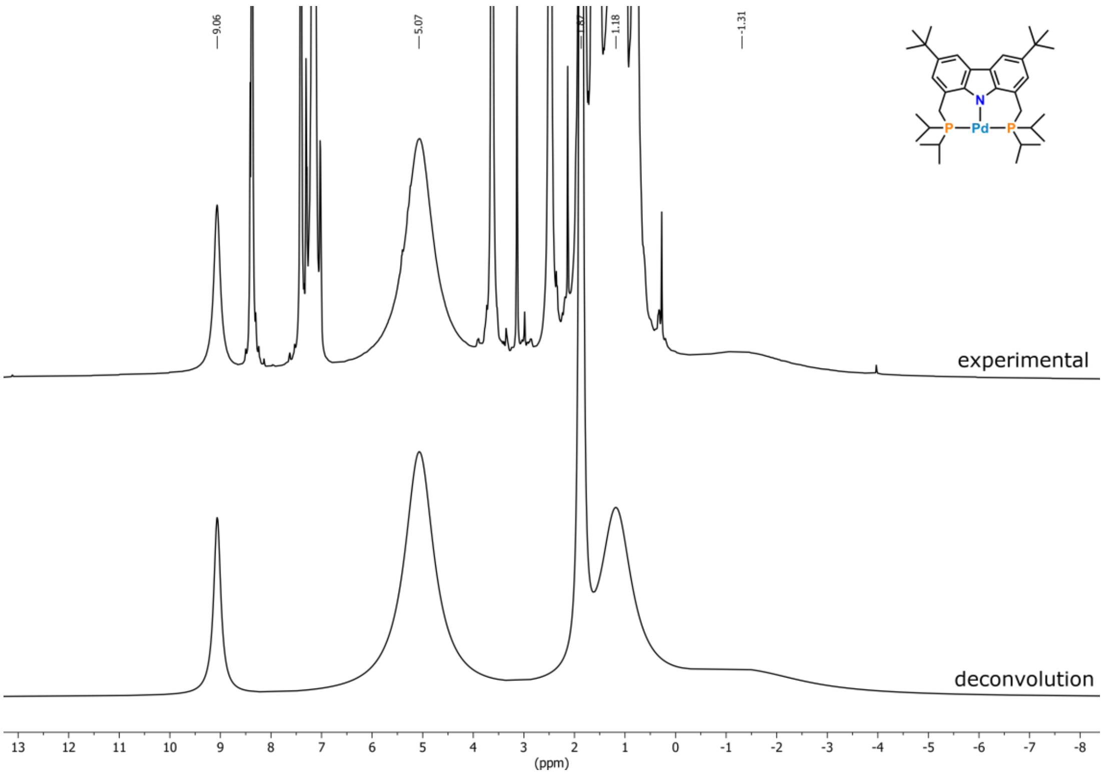

Figure S90. Amplified experimental ^{1}H NMR spectrum (C_6D_6, 600 MHz, 353 K) of **[(^{iPr}PNP)Pd]$_2$ (4^{iPr}-Pd)** including the spectrum of the deconvoluted resonances assigned to **3^{iPr}-Pd**.

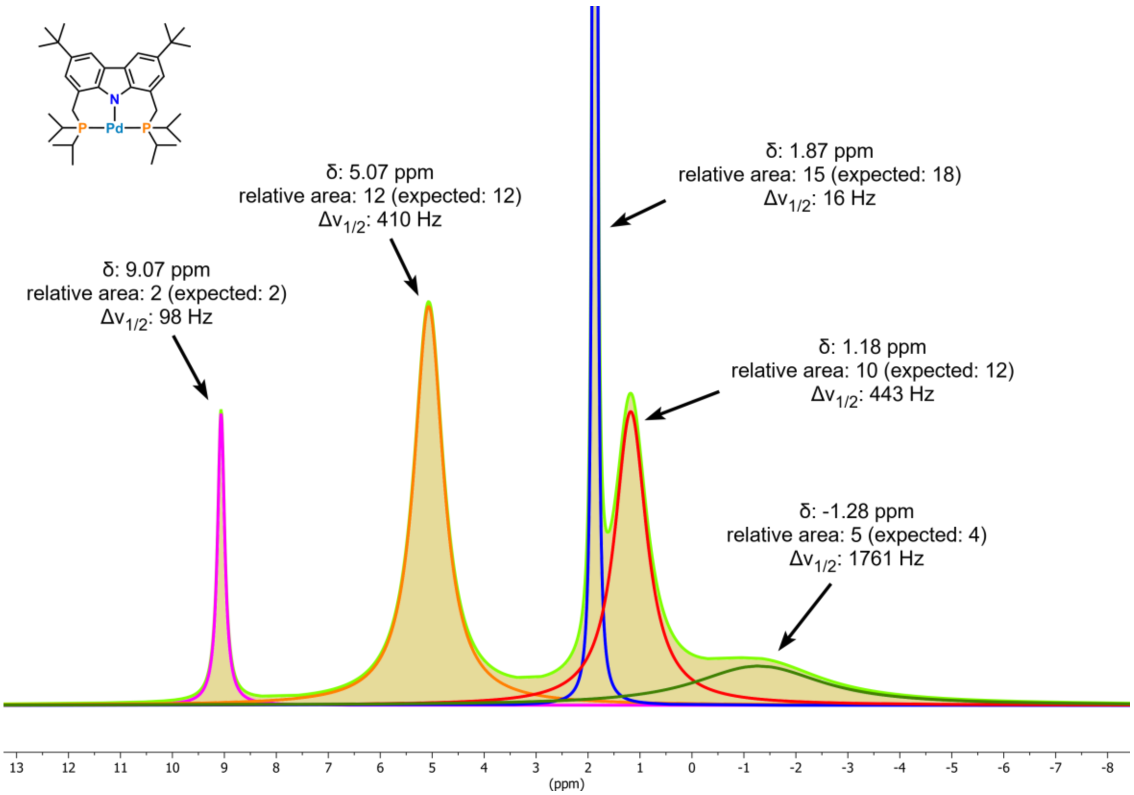

Figure S91. Deconvoluted resonances of the ^{1}H NMR spectrum of **[(^{iPr}PNP)Pd] (3^{iPr}-Pd)** (the light green line resembles the spectrum of the combined deconvoluted signals).

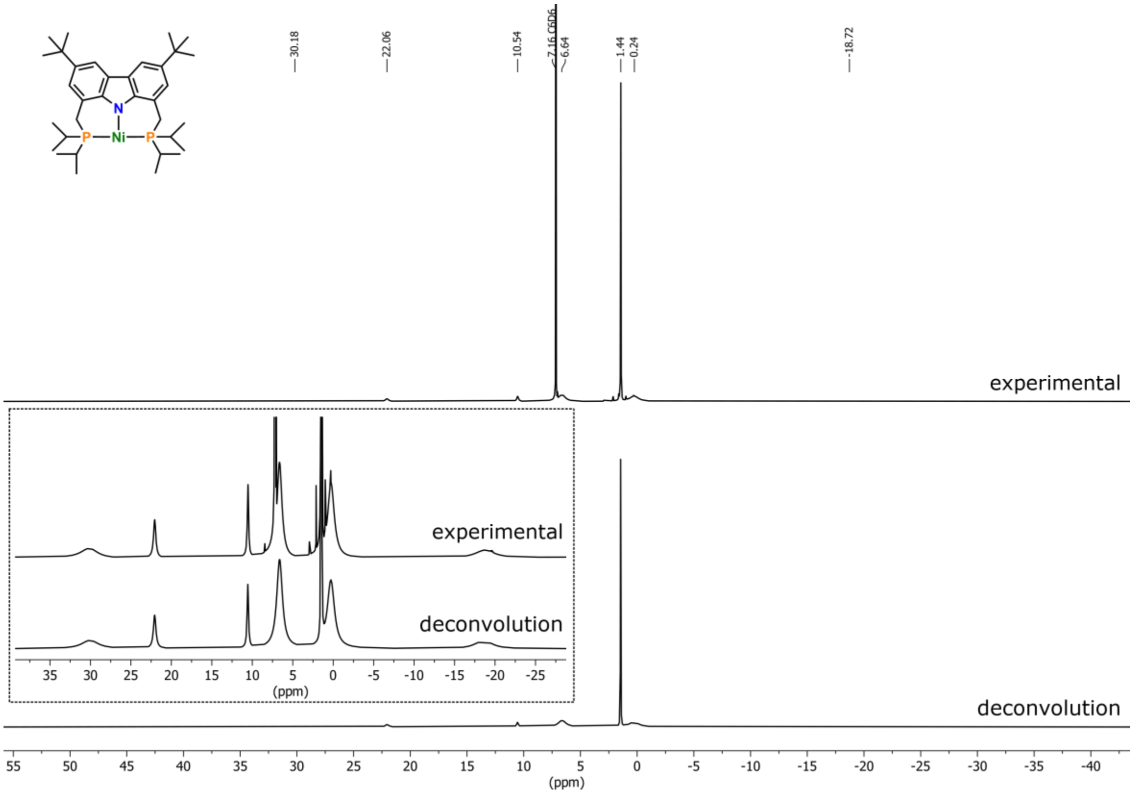

Figure S92. Experimental ^{1}H NMR spectrum (C_6D_6, 600 MHz, 295 K) of **[(^{iPr}PNP)Ni^{I}] (3^{iPr}-Ni)** including the spectrum of the deconvoluted resonances. The inset shows the strongly amplified experimental and deconvoluted spectra.

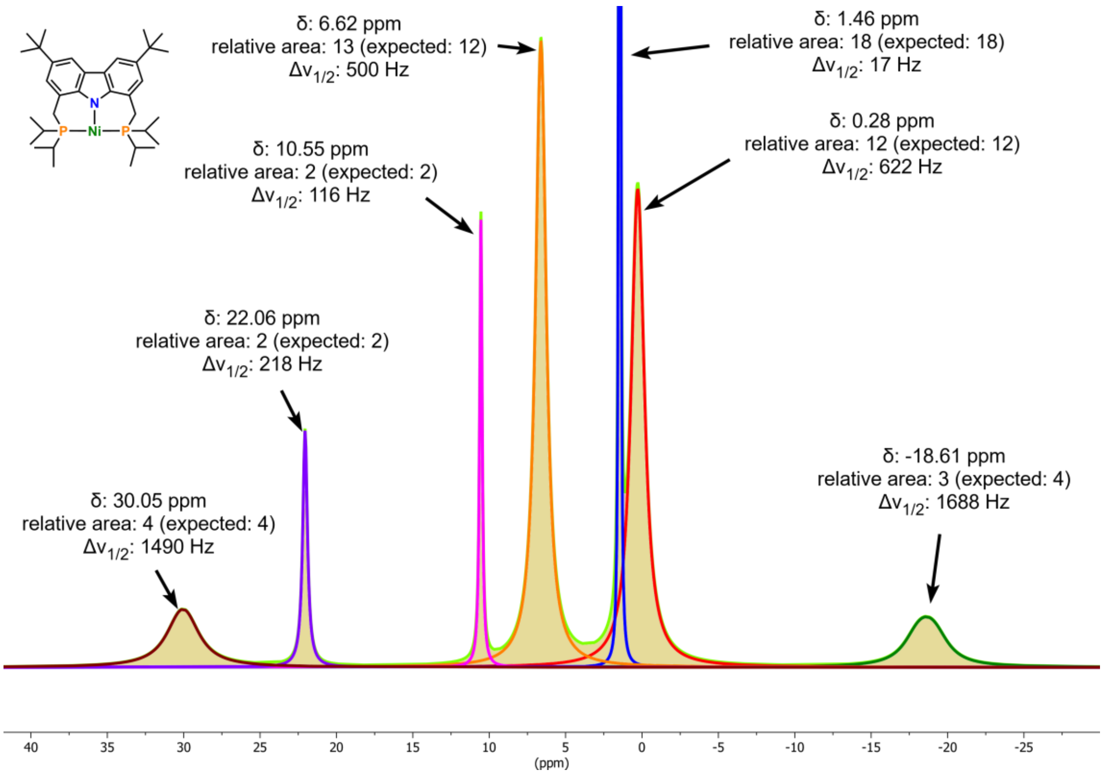

Figure S93. Deconvoluted resonances of the ^{1}H NMR spectrum of **[(^{iPr}PNP)Ni^{I}] (3^{iPr}-Ni)** (the light green line resembles the spectrum of the combined deconvoluted signals).

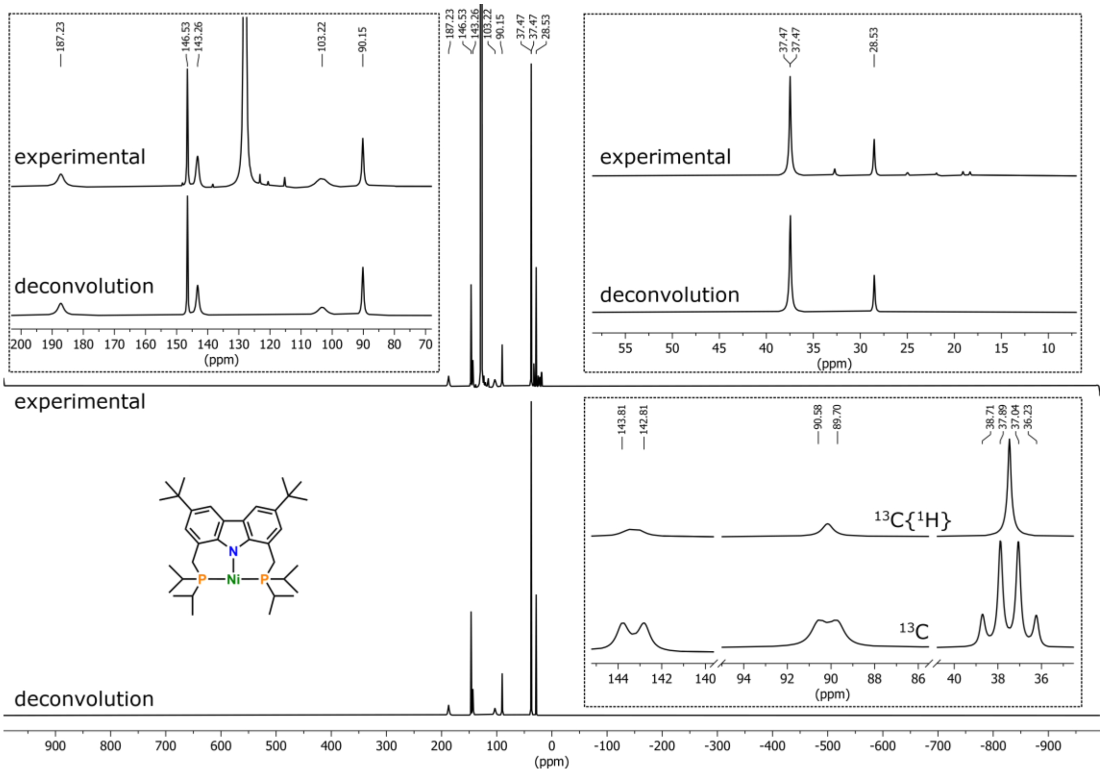

Figure S94. Experimental ^{13}C NMR spectrum (C_6D_6, 151 MHz, 295 K) of **[(^{iPr}PNP)Ni^{I}] (3^{iPr}-Ni)** including the spectrum of the deconvoluted resonances. The insets on top show strongly amplified sections of the experimental and deconvoluted spectra. The inset on bottom shows three carbon resonances exhibiting resolved ^{1}H-coupling in the experimental spectrum.

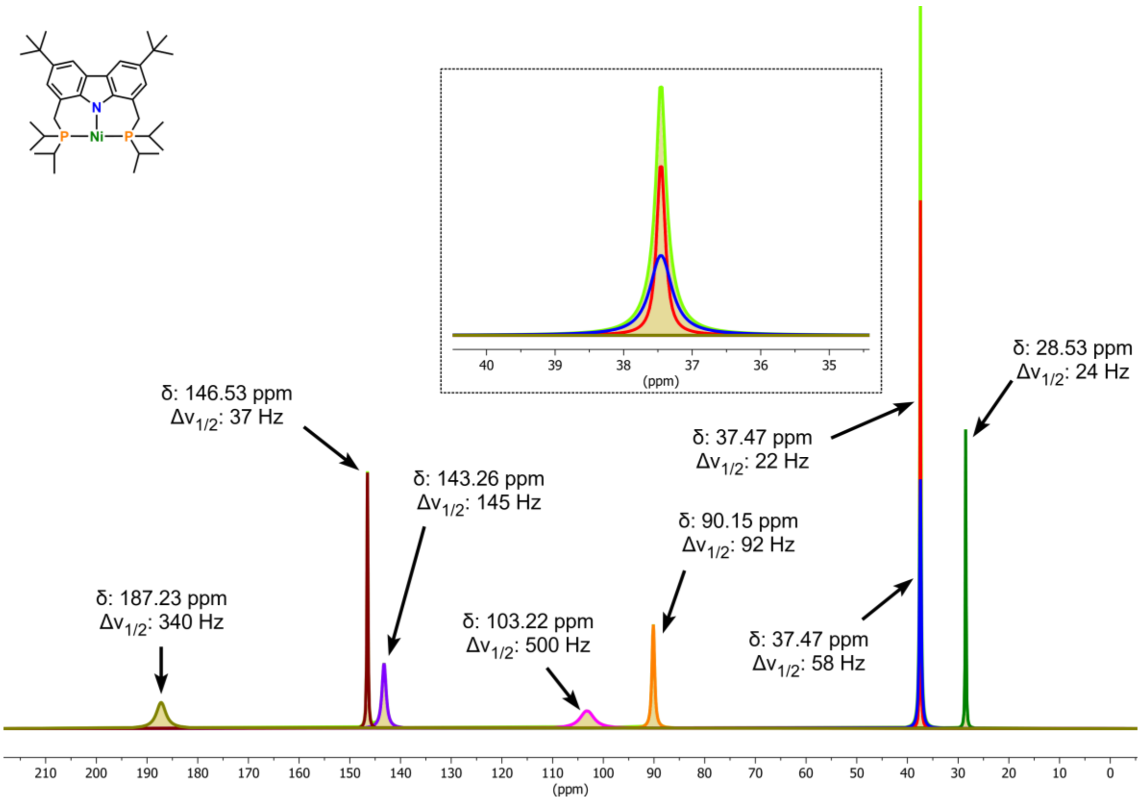

Figure S95. Deconvoluted resonances of the ^{13}C NMR spectrum of **[(^{iPr}PNP)Ni^{I}] (3^{iPr}-Ni)**. The inset shows two superimposed deconvoluted resonances at 37.47 ppm (the light green line resembles the spectrum of the combined deconvoluted signals).

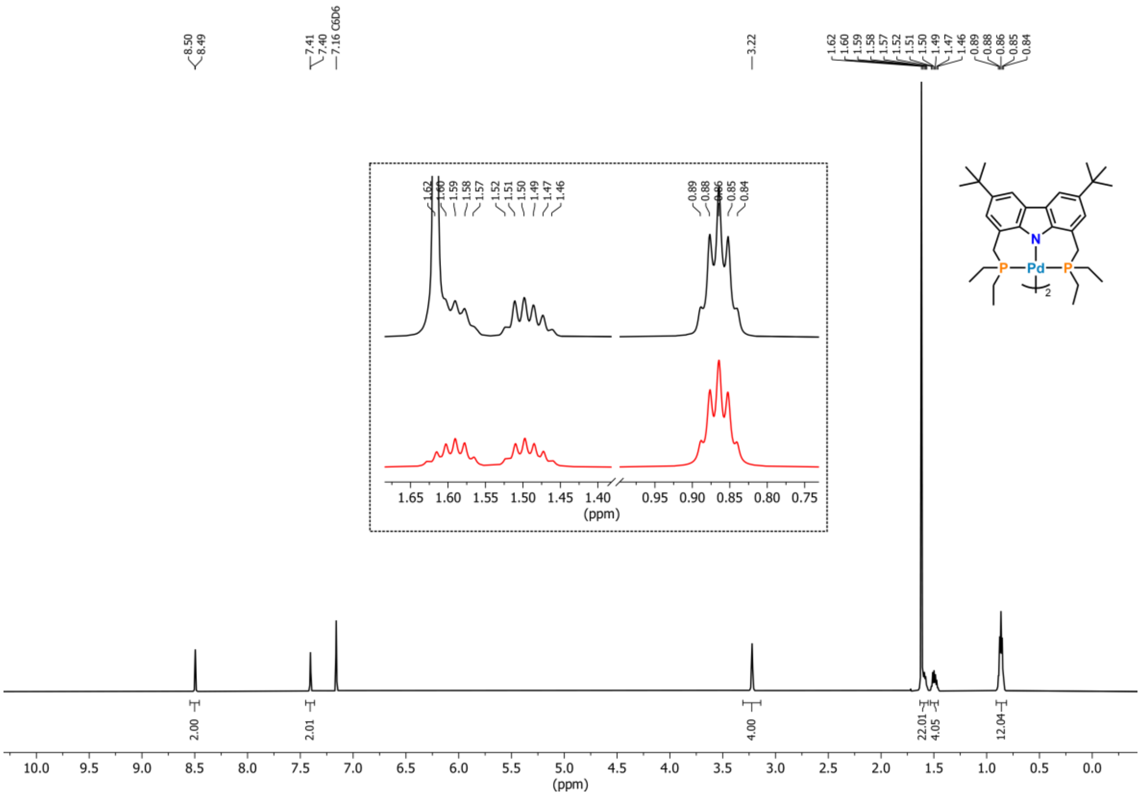

Figure S96. ^{1}H NMR spectrum (C_6D_6, 600 MHz, 295 K) of **[(^{Et}PNP)Pd]$_2$ (4^{Et}-Pd)**. Simulated multiplets are depicted in red.

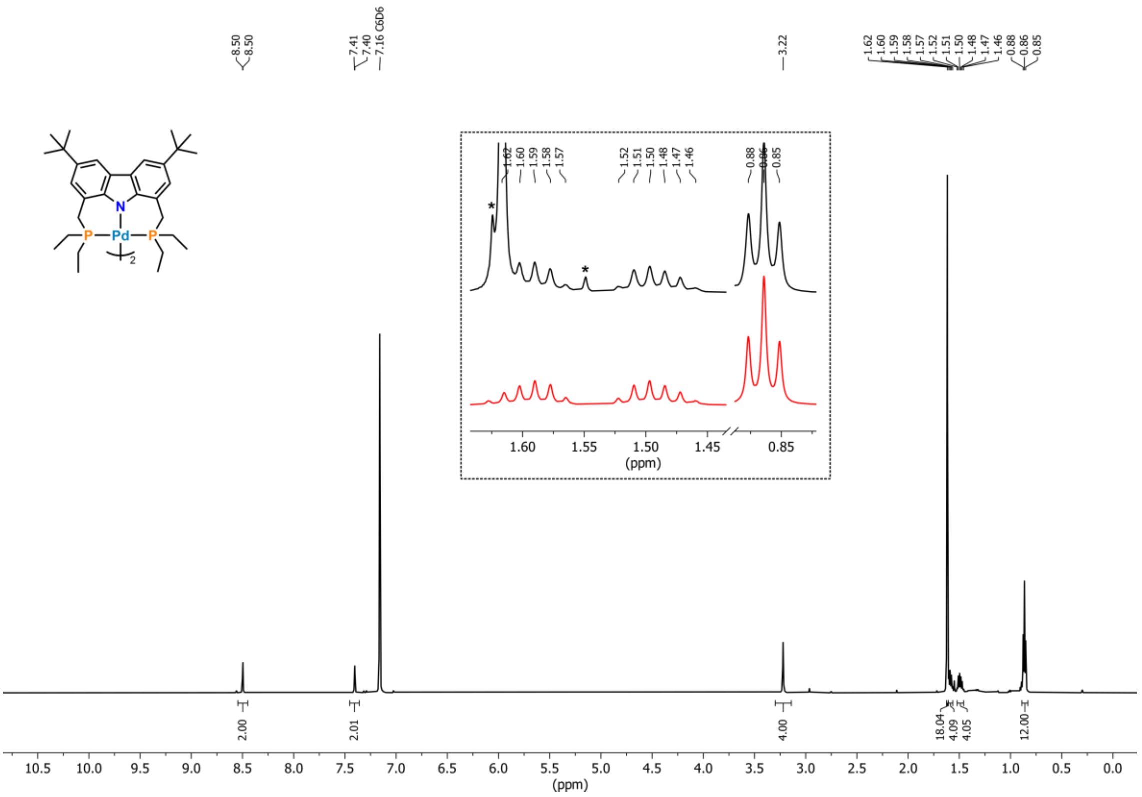

Figure S97. $^{1}H\{^{31}P\}$ NMR spectrum (C_6D_6, 600 MHz, 295 K) of **[(^{Et}PNP)Pd]$_2$ (4^{Et}-Pd)** (* = unidentified impurity). Simulated multiplets are depicted in red.

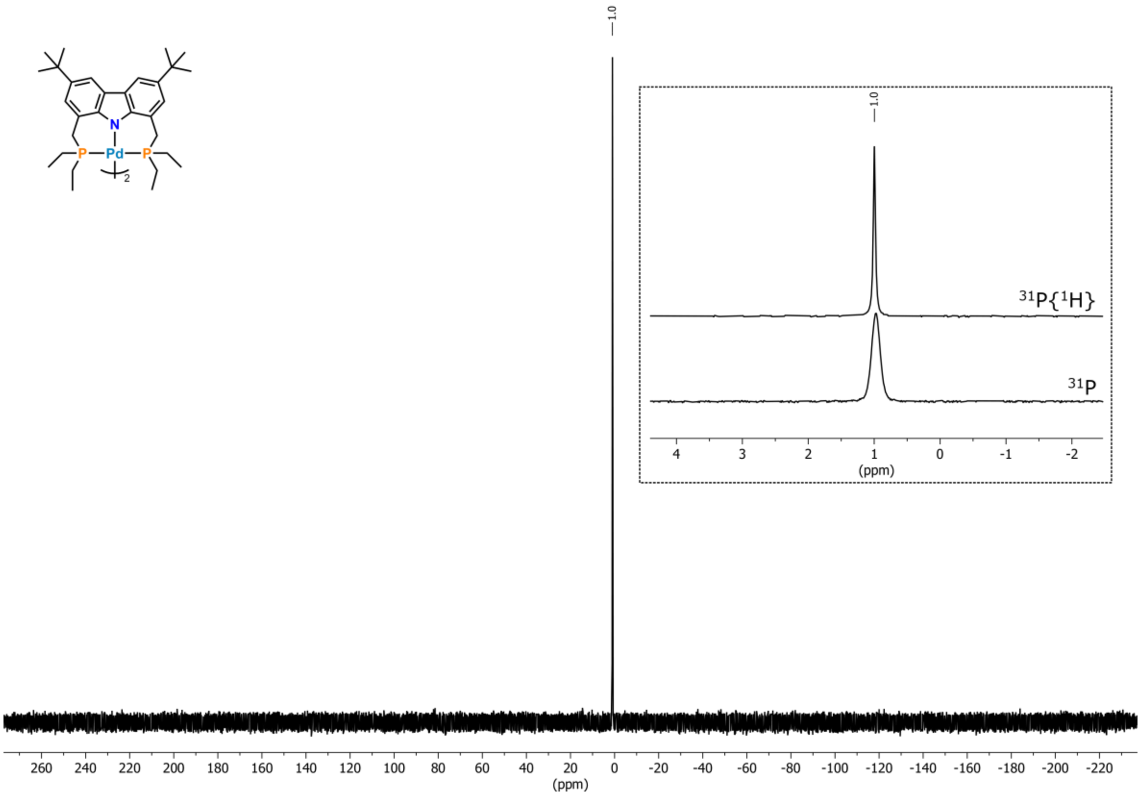

Figure S98. ^{31}P and $^{31}P\{^{1}H\}$ NMR spectrum (C_6D_6, 243 MHz, 295K) of **[(^{Et}PNP)Pd]$_2$ (4^{Et}-Pd)**.

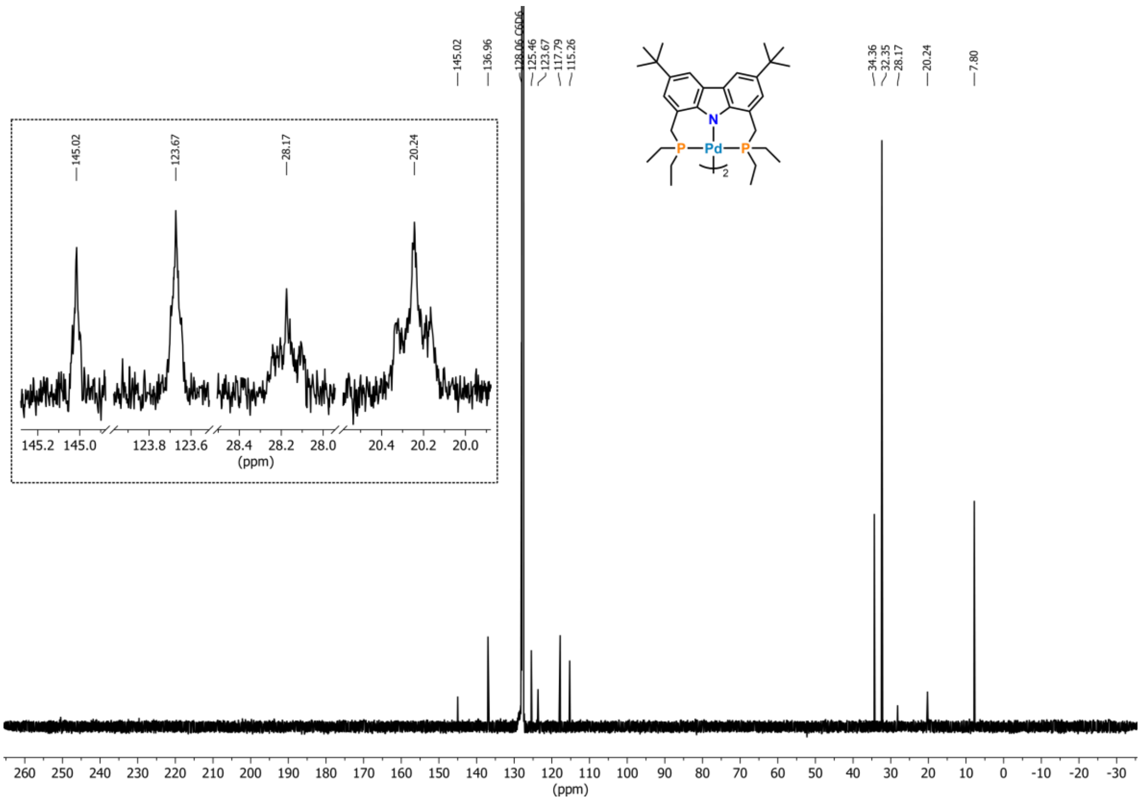

Figure S99. $^{13}C\{^1H\}$ NMR spectrum (C_6D_6, 151 MHz, 295 K) of **[(^{Et}PNP)Pd]$_2$ (4^{Et}-Pd)**.

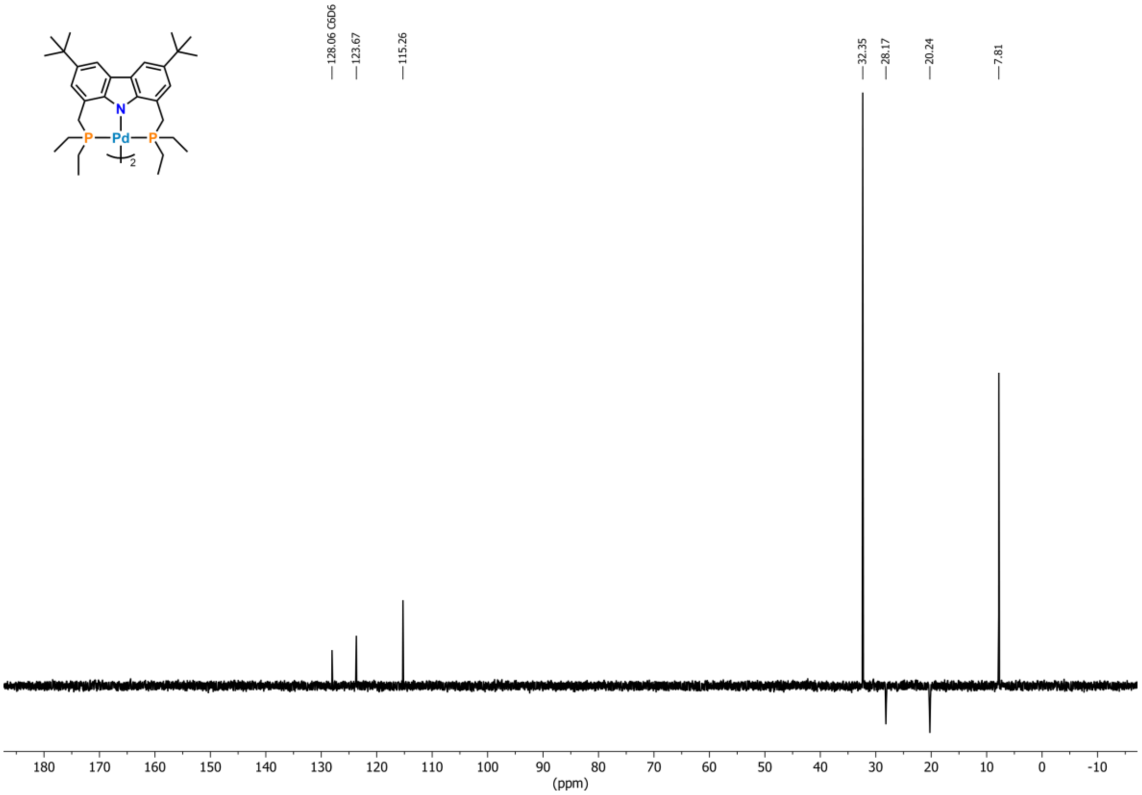

Figure S100. $^{13}C\{^1H\}$ DEPT-135 NMR spectrum (C_6D_6, 151 MHz, 295 K) of **[(^{Et}PNP)Pd]$_2$ (4^{Et}-Pd)**.

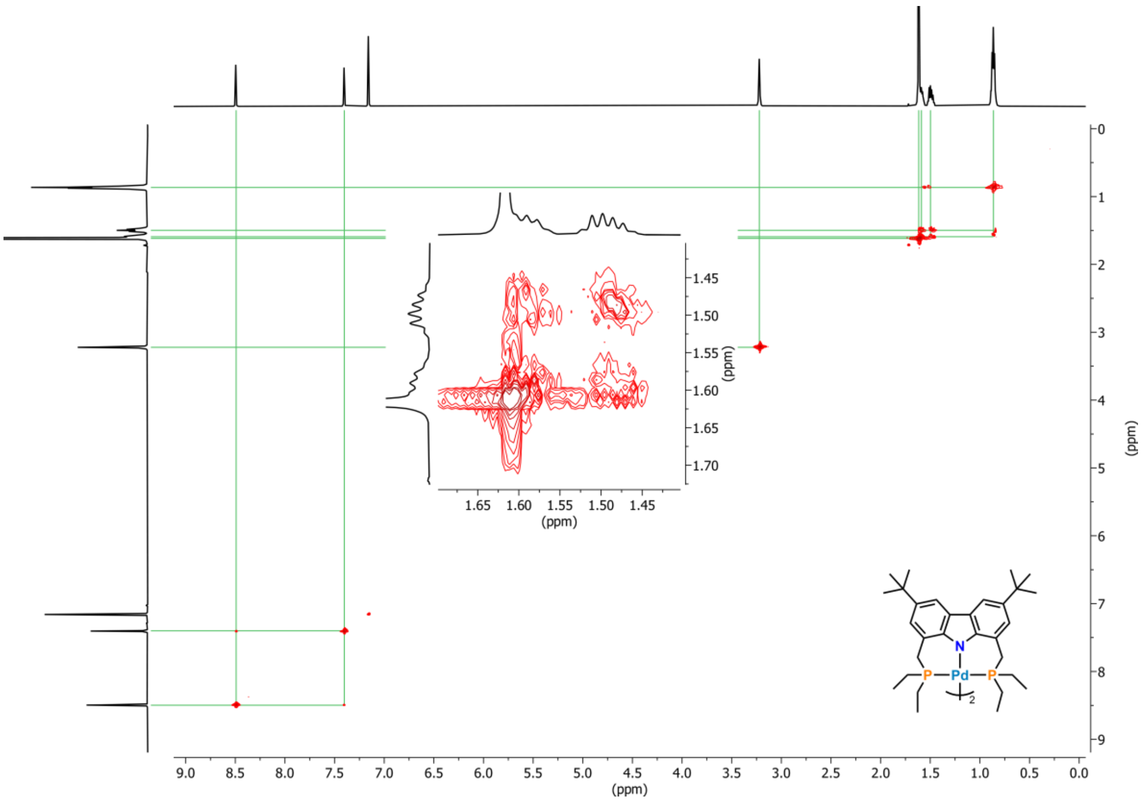

Figure S101. $^1H/^1H$ COSY NMR spectrum (C_6D_6, 600/600 MHz, 295 K) of **[(^{Et}PNP)Pd]$_2$ (4^{Et}-Pd)**.

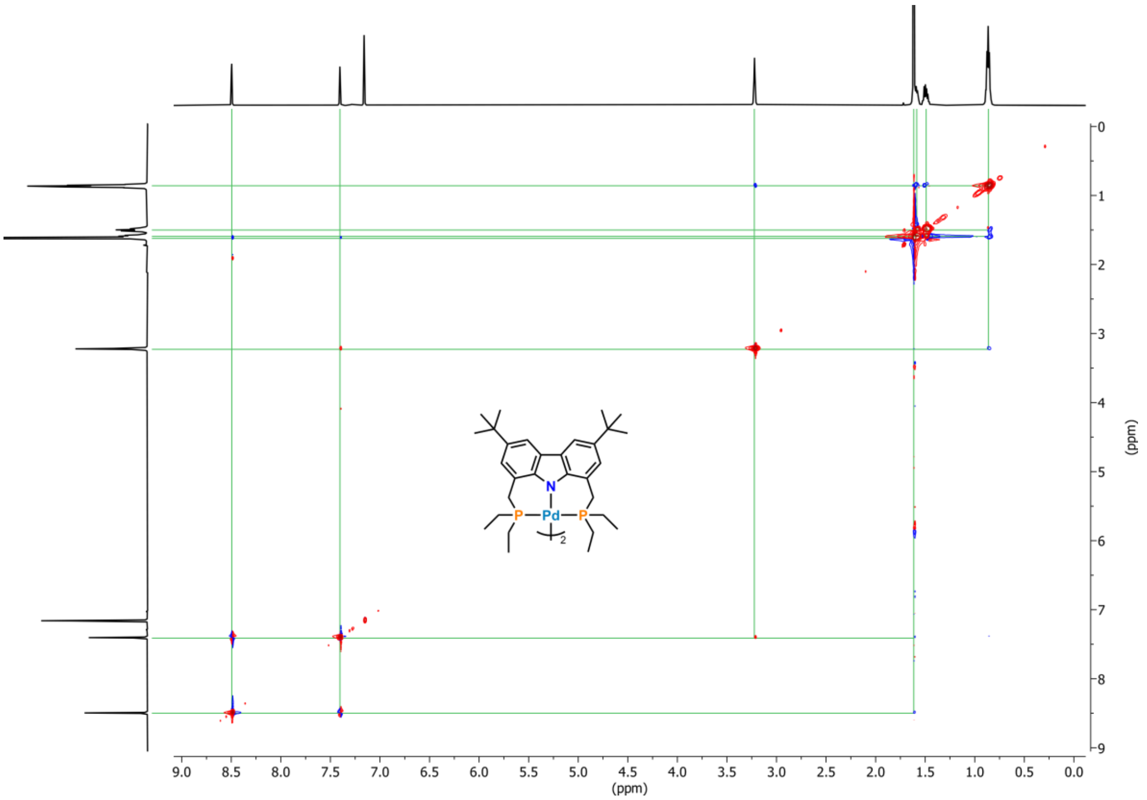

Figure S102. $^1H/^1H$ NOESY NMR spectrum (C_6D_6, 600/600 MHz, 295 K) of **[(^{Et}PNP)Pd]$_2$ (4^{Et}-Pd)**.

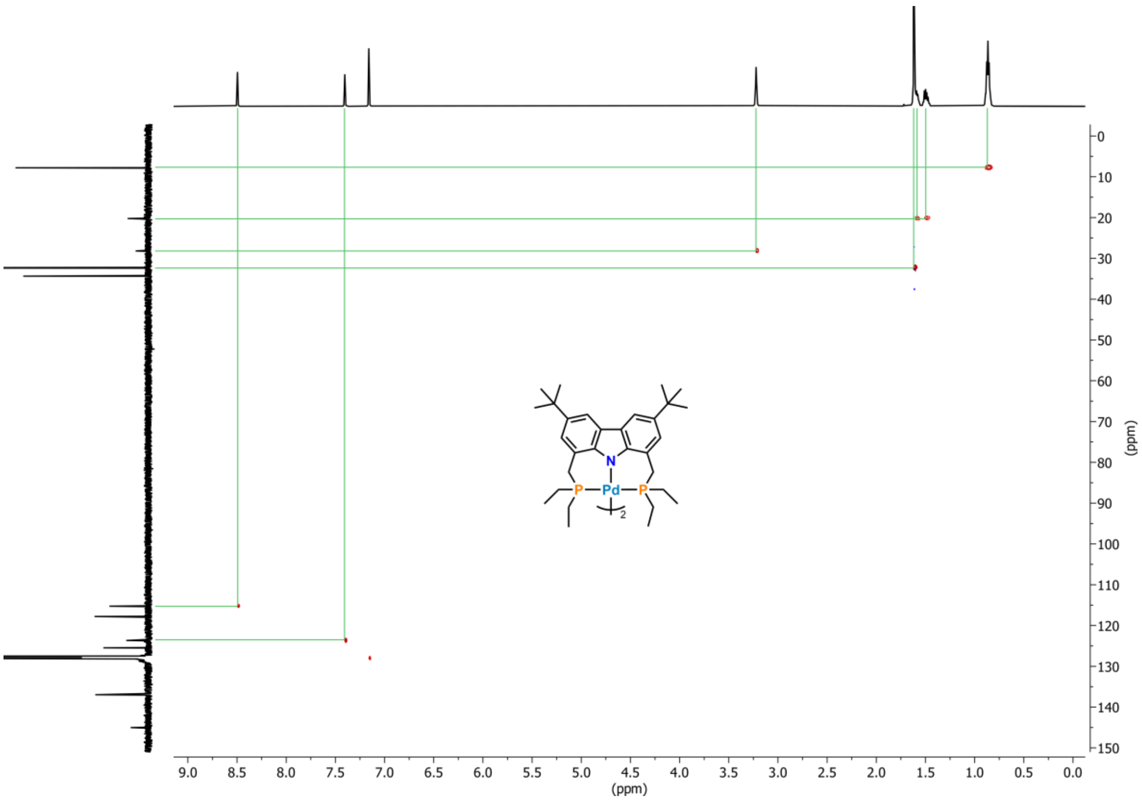

Figure S103. $^{1}H/^{13}C$ HSQC NMR spectrum (C_6D_6, 600/151 MHz, 295 K) of **[(^{Et}PNP)Pd]$_2$ (4^{Et}-Pd)**.

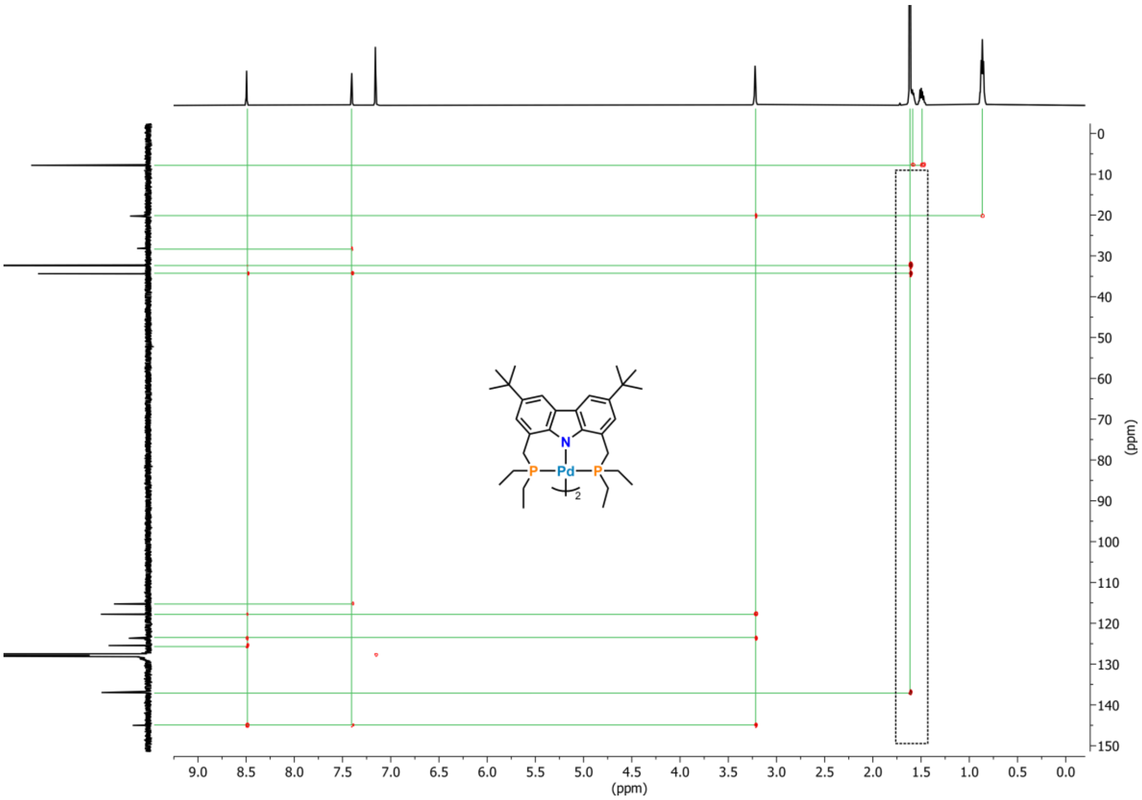

Figure S104. $^{1}H/^{13}C$ HMBC NMR spectrum (C_6D_6, 600/151 MHz, 295 K) of **[(^{Et}PNP)Pd]$_2$ (4^{Et}-Pd)**. Due to a large dynamic range, the signal intensities were decreased in the inset.

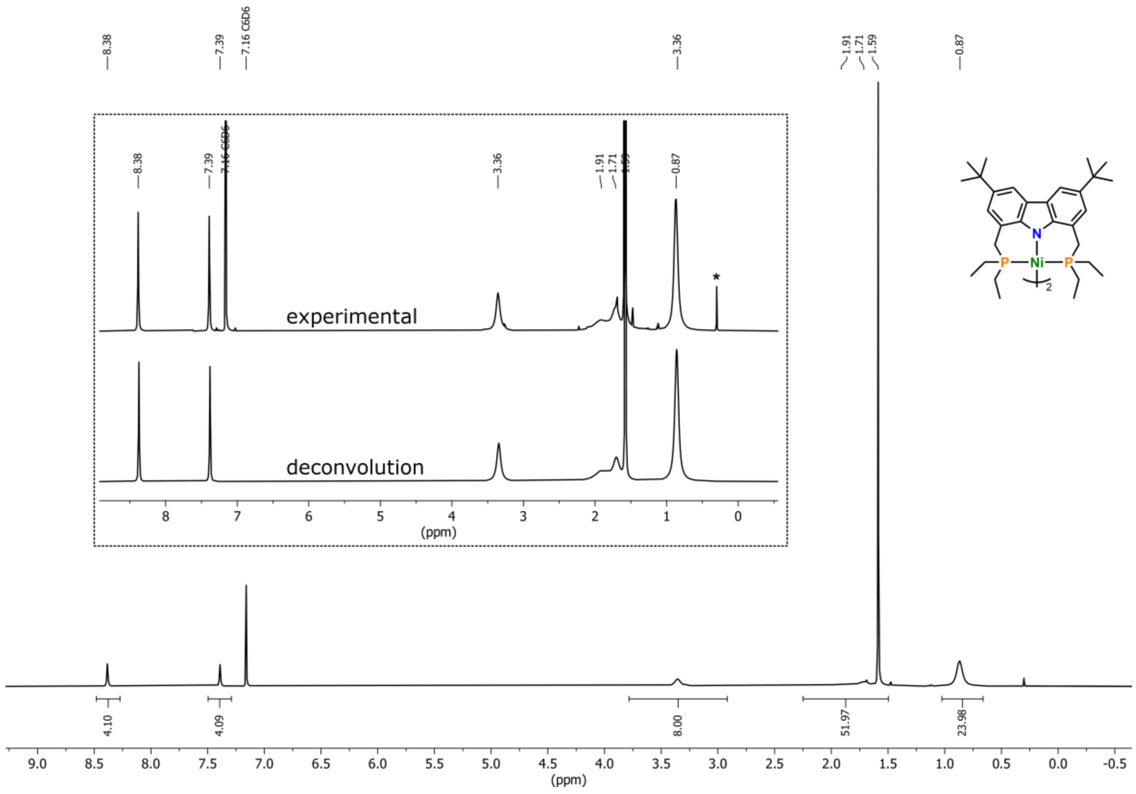

Figure S105. Experimental ^{1}H NMR spectrum (C_6D_6, 600 MHz, 295 K) of **[(^{Et}PNP)Ni]$_2$ (4^{Et}-Ni)**. The inset includes an amplified version of the experimental spectrum and the spectrum of the deconvoluted resonances (* = grease).

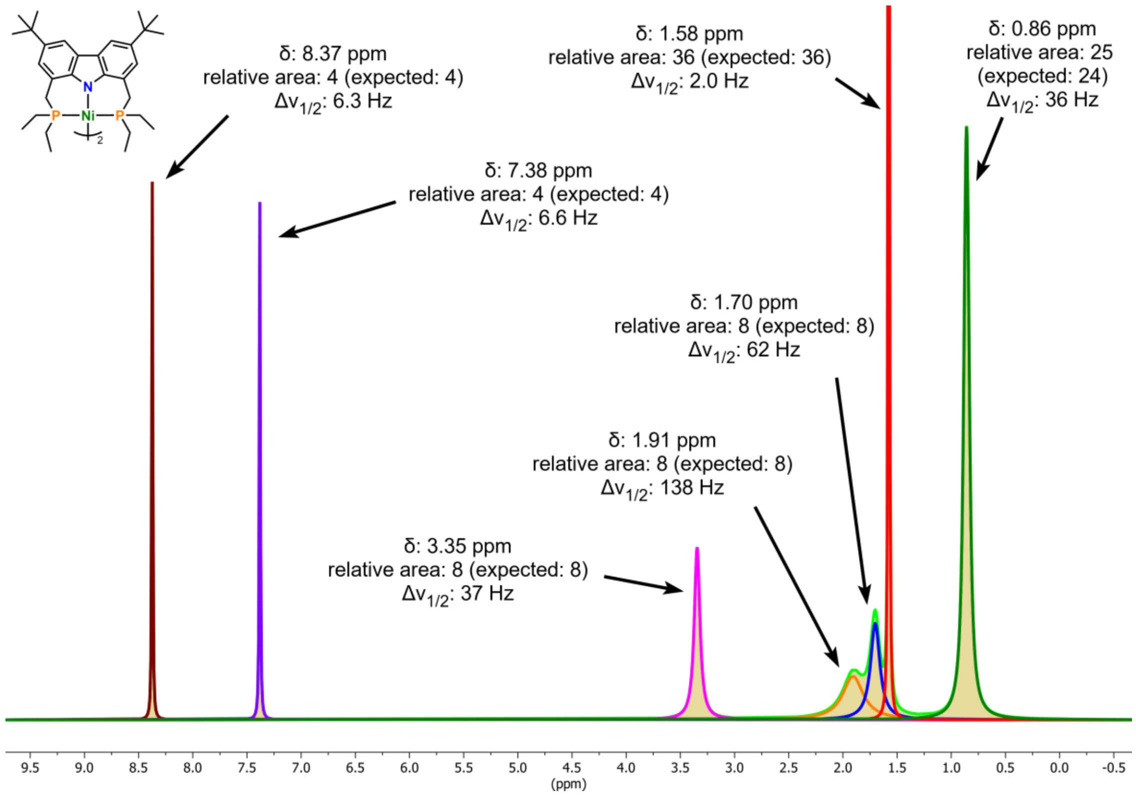

Figure S106. Deconvoluted resonances of the ^{1}H NMR spectrum of **[(^{Et}PNP)Ni]$_2$ (4^{Et}-Ni)** (the light green line resembles the spectrum of the combined deconvoluted signals).

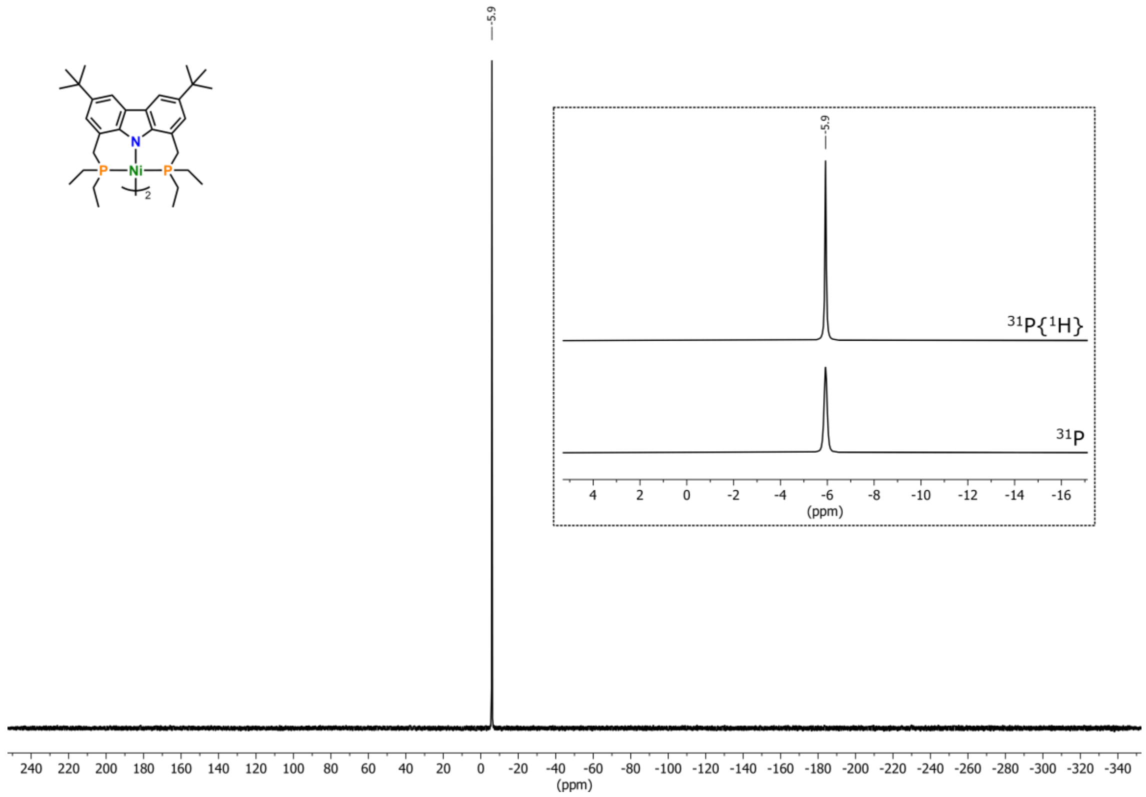

Figure S107. ^{31}P and $^{31}P\{^1H\}$ NMR spectrum (C_6D_6, 243 MHz, 295K) of **$[(^{Et}PNP)Ni]_2$ (4^{iPr}-Ni)**.

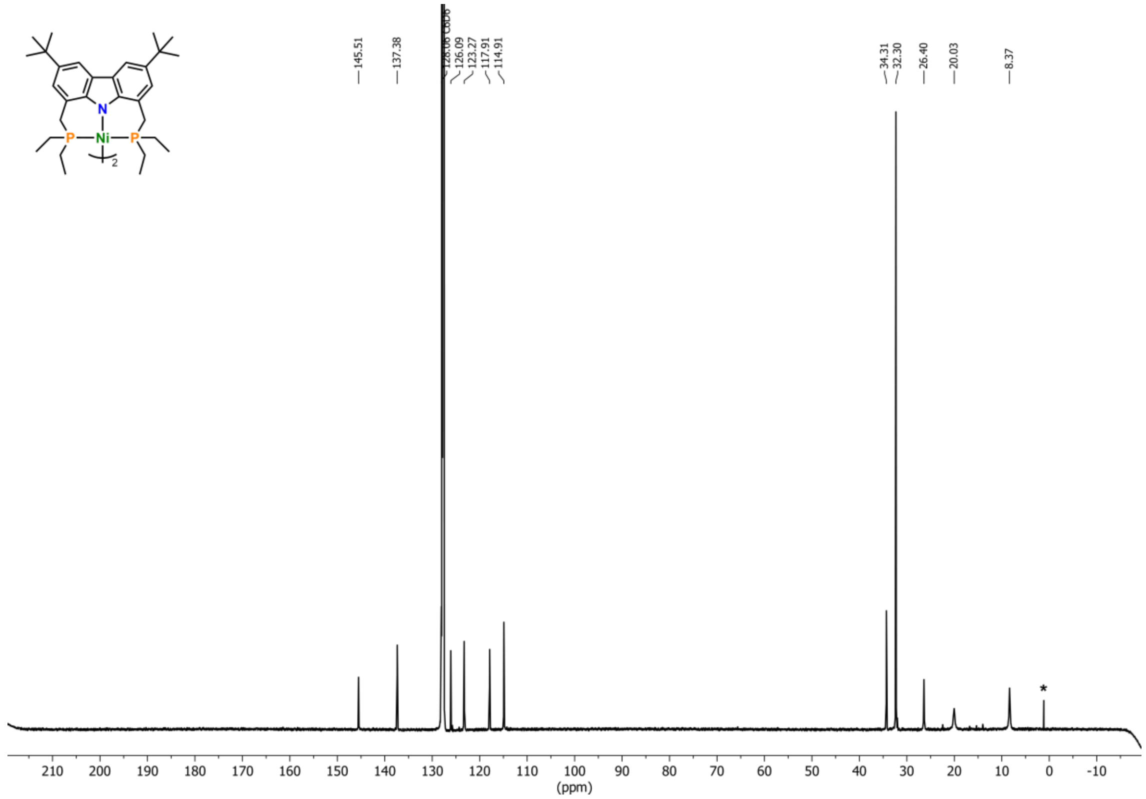

Figure S108. $^{13}C\{^1H\}$ NMR spectrum (C_6D_6, 151 MHz, 295 K) of **$[(^{Et}PNP)Ni]2$ (4^{Et}-Ni)** (* = grease).

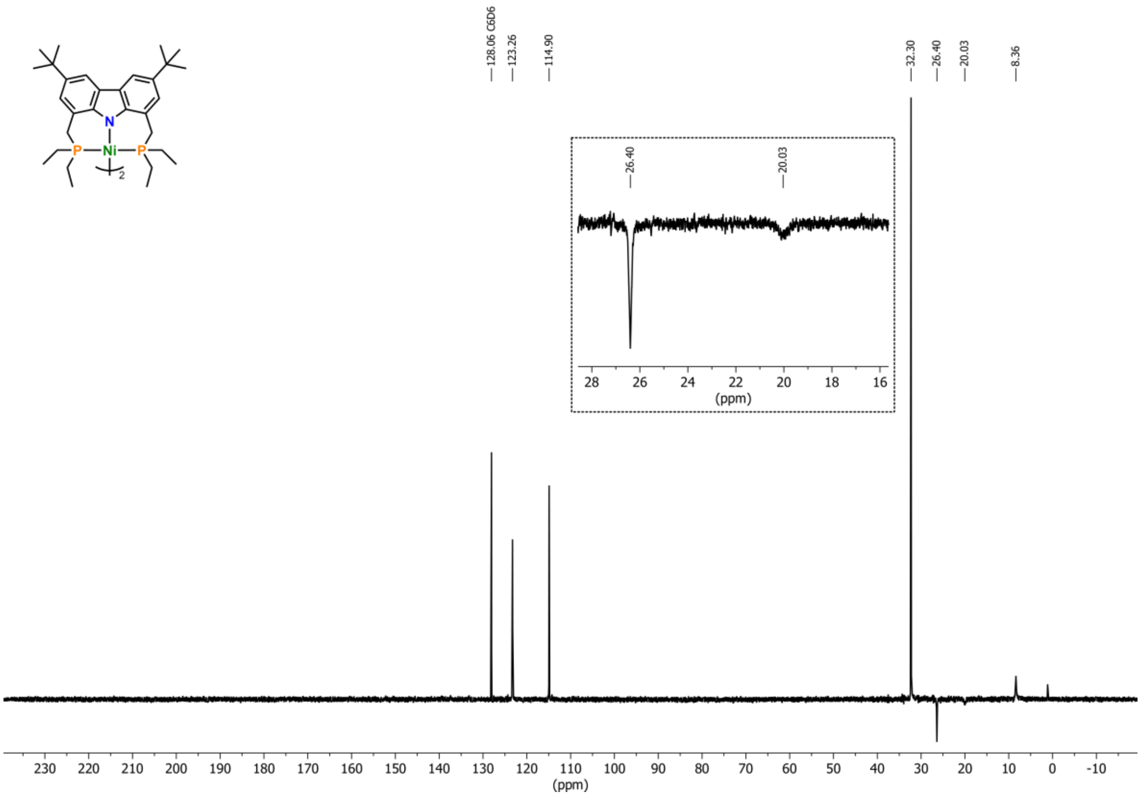

Figure S109. $^{13}C\{^1H\}$ DEPT-135 NMR spectrum (C_6D_6, 151 MHz, 295 K) of **[(^{Et}PNP)Ni]2 (4^{Et}-Ni)**.

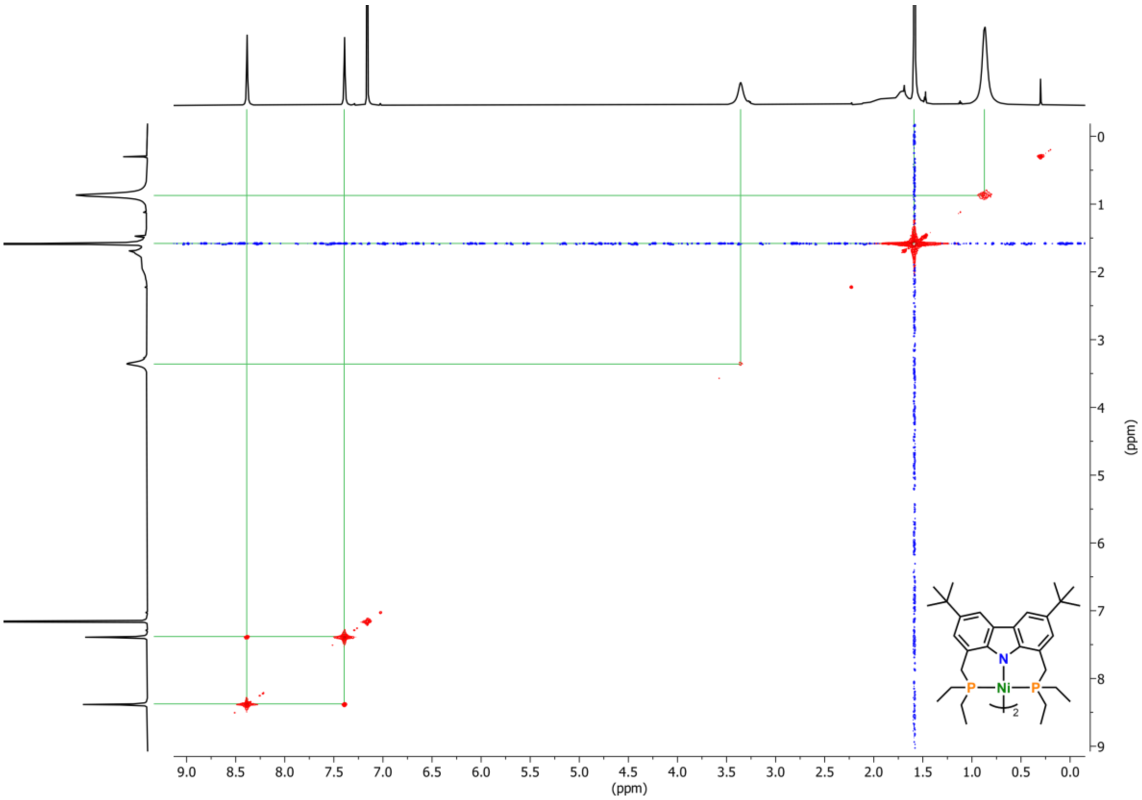

Figure S110. $^1H/^1H$ COSY NMR spectrum (C_6D_6, 600/600 MHz, 295 K) of **[(^{Et}PNP)Ni]$_2$ (4^{Et}-Ni)**.

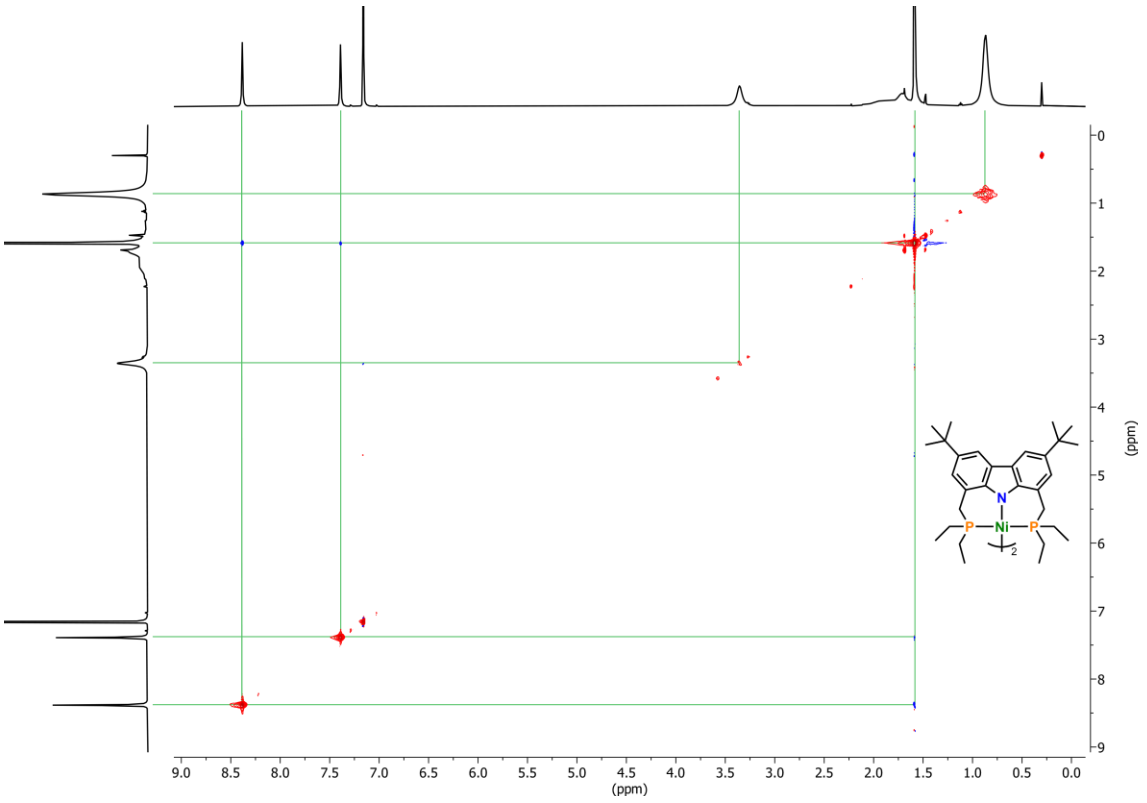

Figure S111. $^1H/^1H$ NOESY NMR spectrum (C_6D_6, 600/600 MHz, 295 K) of **[(^{Et}PNP)Ni]$_2$ (4^{Et}-Ni)**.

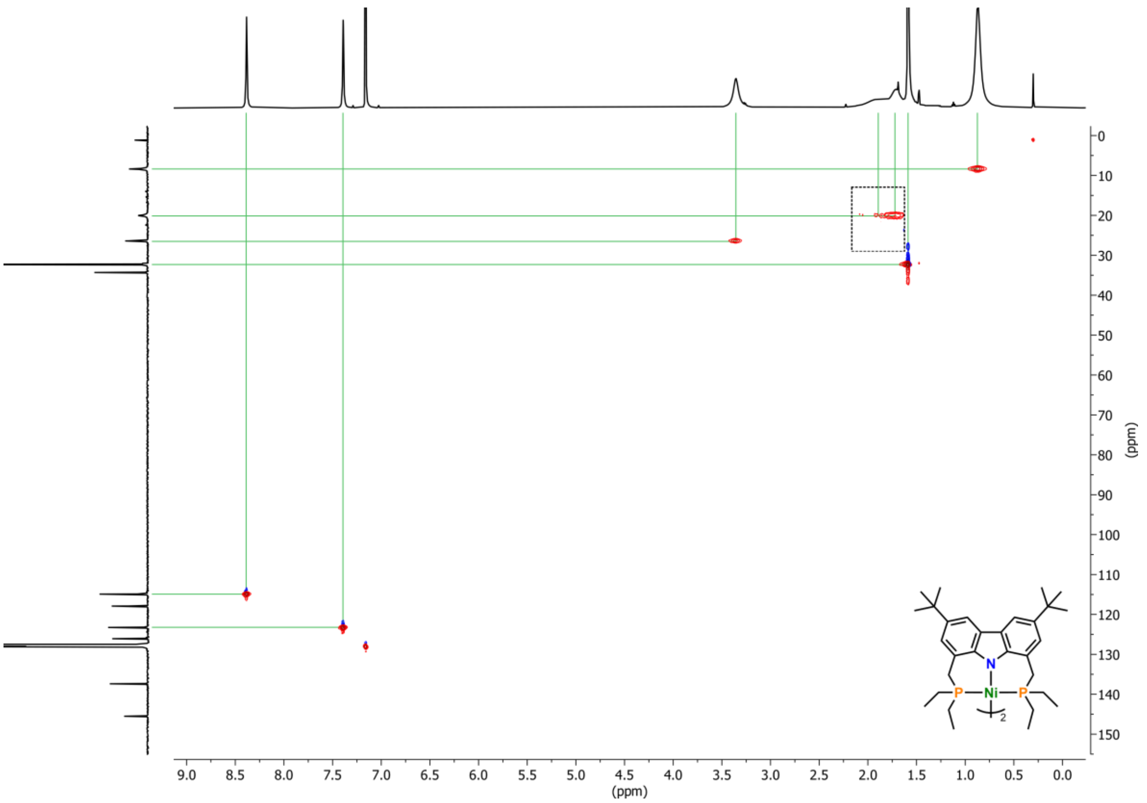

Figure S112. $^1H/^{13}C$ HSQC NMR spectrum (C_6D_6, 600/151 MHz, 295 K) of **[(^{Et}PNP)Ni]2 (4^{Et}-Ni)**. Due to a large dynamic range, the weaker signals were amplified in the inset.

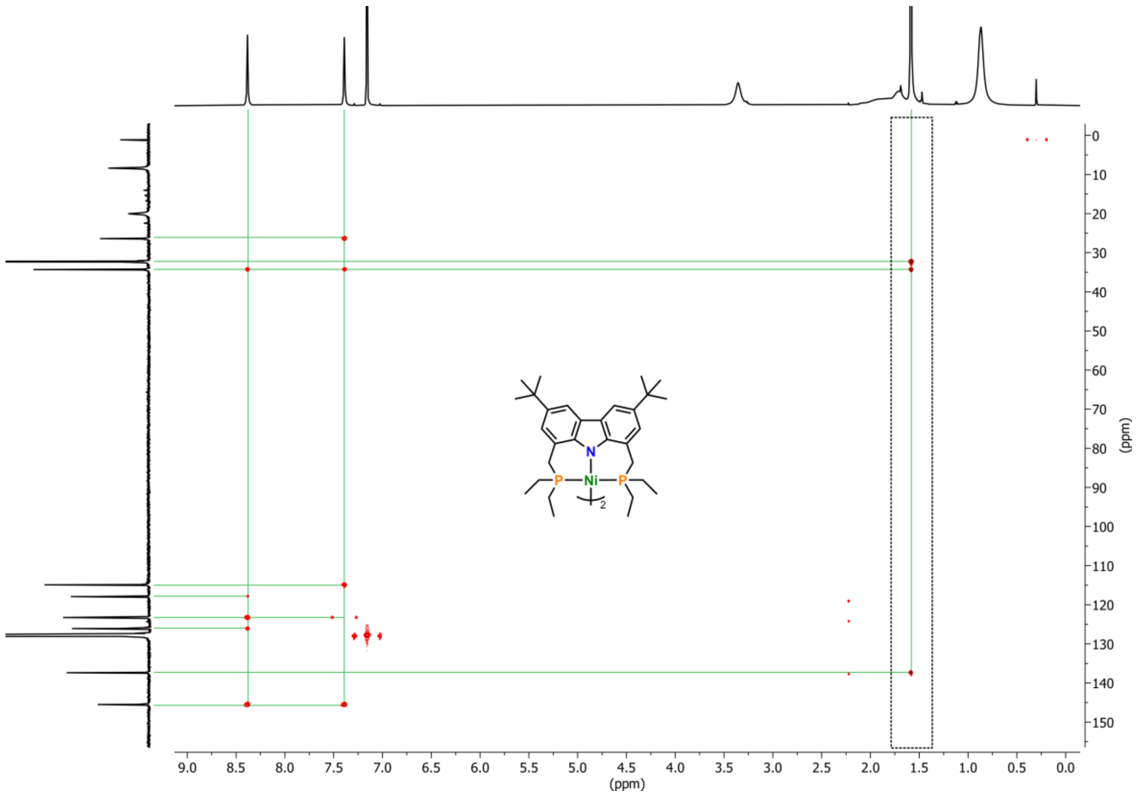

Figure S113. $^{1}H/^{13}C$ HMBC NMR spectrum (C_6D_6, 600/151 MHz, 295 K) of **[(^{Et}PNP)Ni]2 (4^{Et}-Ni)**. Due to a large dynamic range, the signal intensities were decreased in the inset.

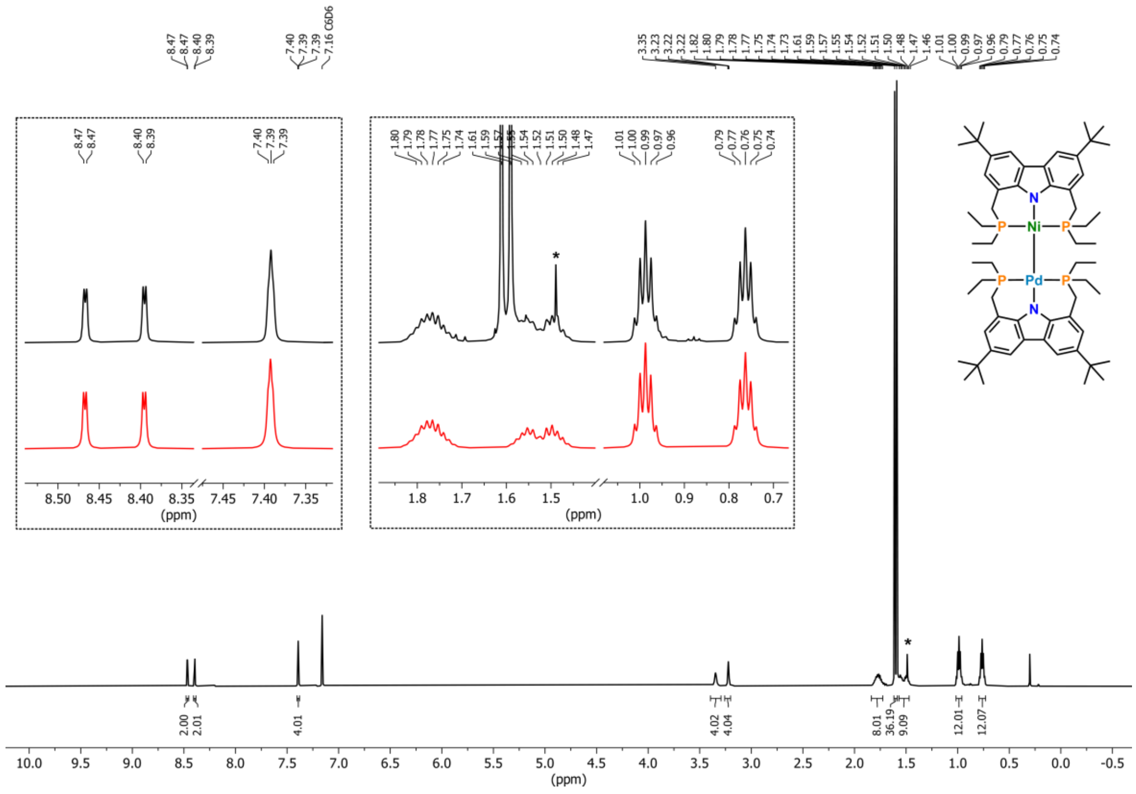

Figure S114. ^{1}H NMR spectrum (C_6D_6, 600 MHz, 295 K) of **[(^{Et}PNP)Ni-Pd(^{Et}PNP)] (7^{Et}-NiPd)** (* unidentified impurity). Simulated multiplets are depicted in red.

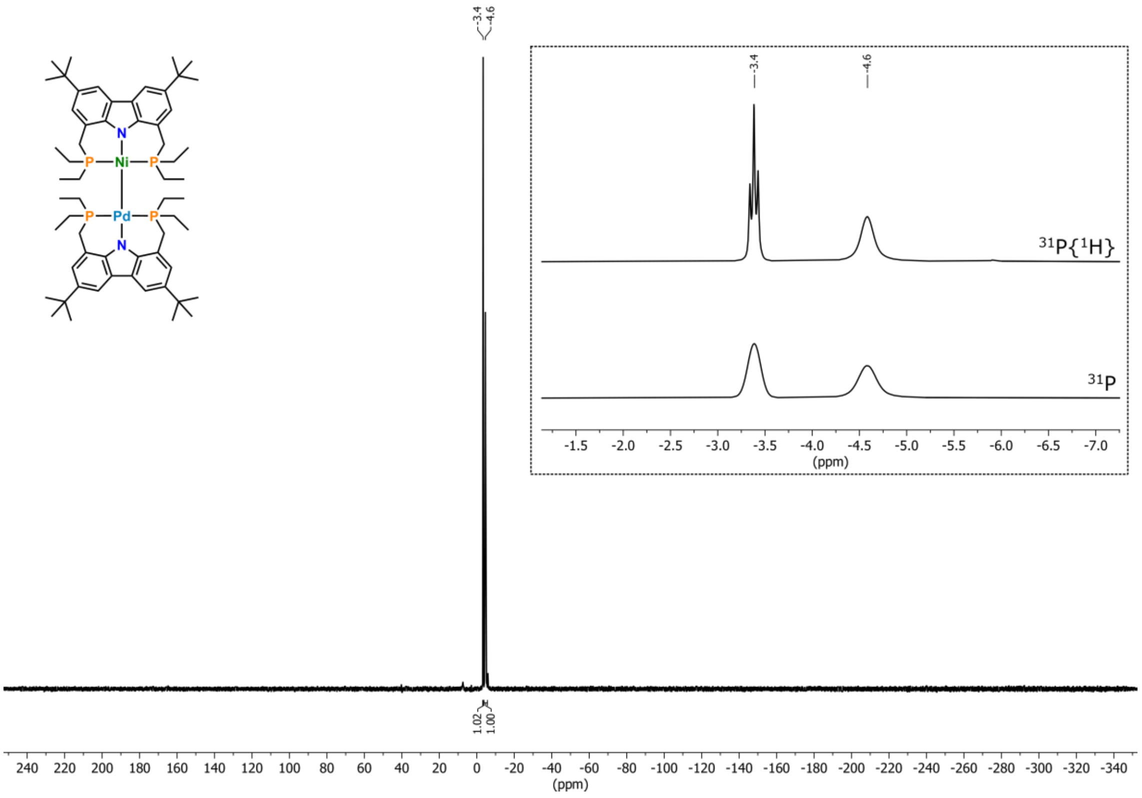

Figure S115. ^{31}P and $^{31}P\{^1H\}$ NMR spectrum (C_6D_6, 243 MHz, 295K) of **[(^{Et}PNP)Ni-Pd(^{Et}PNP)] (7^{Et}-NiPd)**.

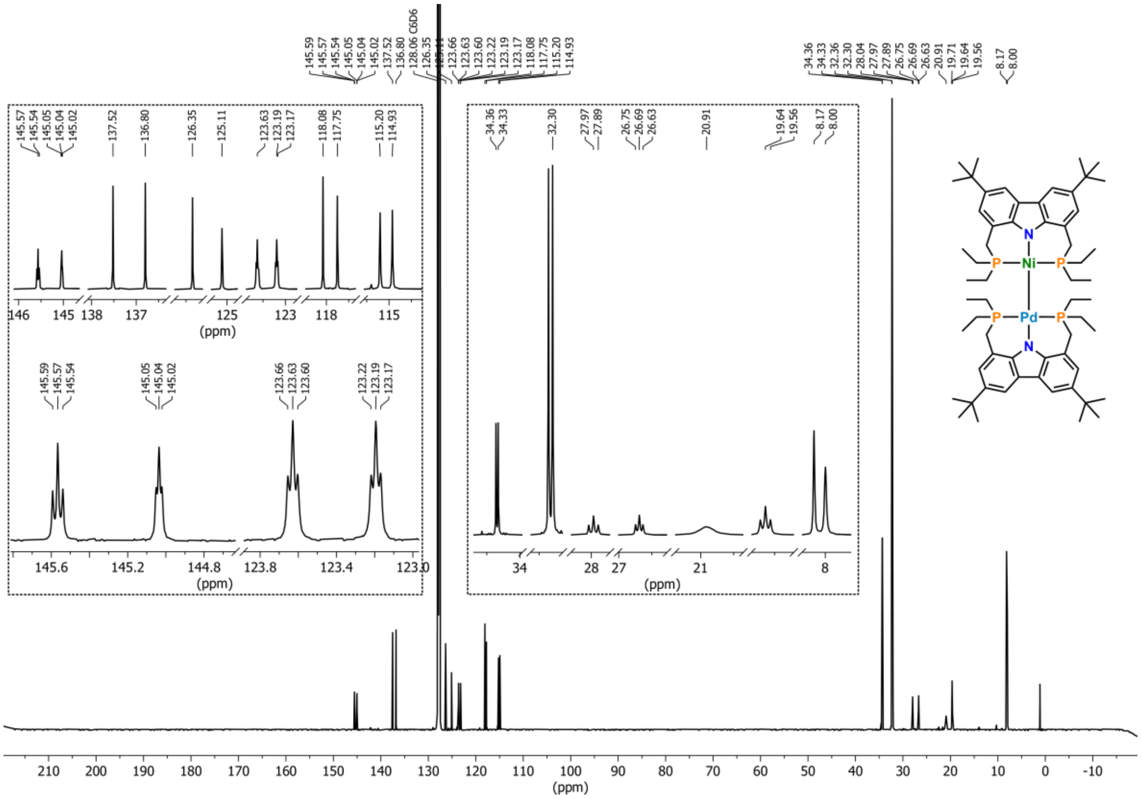

Figure S116. $^{13}C\{^1H\}$ NMR spectrum (C_6D_6, 151 MHz, 295 K) of **[(^{Et}PNP)Ni-Pd(^{Et}PNP)] (7^{Et}-NiPd)**.

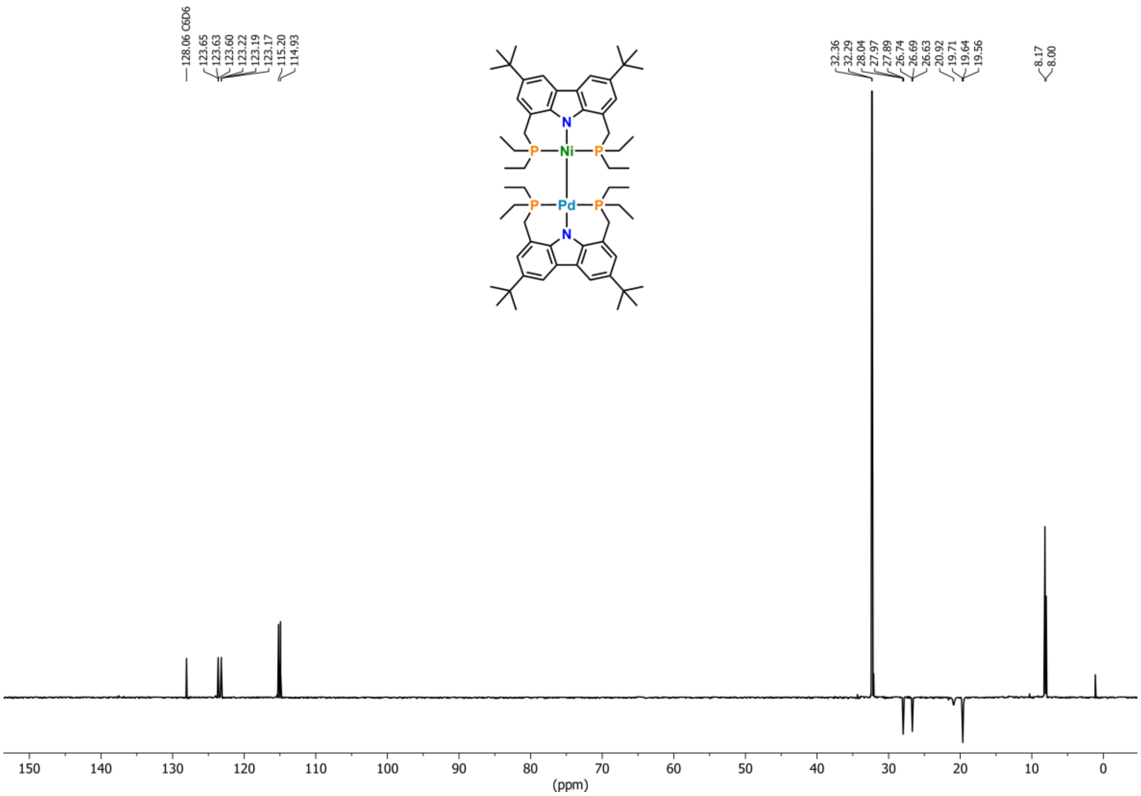

Figure S117. $^{13}C\{^{1}H\}$ DEPT-135 NMR spectrum (C_6D_6, 151 MHz, 295 K) of **[(^{Et}PNP)Ni-Pd(^{Et}PNP)] (7^{Et}-NiPd)**.

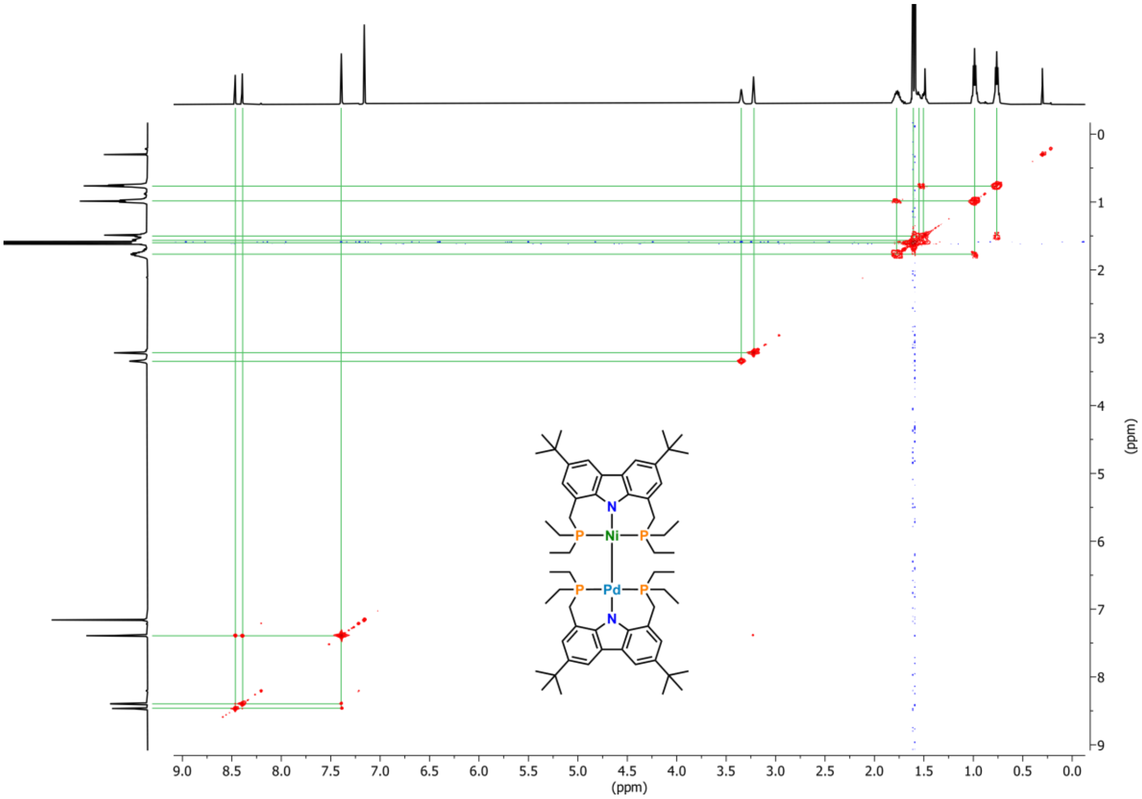

Figure S118. $^{1}H/^{1}H$ COSY NMR spectrum (C_6D_6, 600/600 MHz, 295 K) of **[(^{Et}PNP)Ni-Pd(^{Et}PNP)] (7^{Et}-NiPd)**.

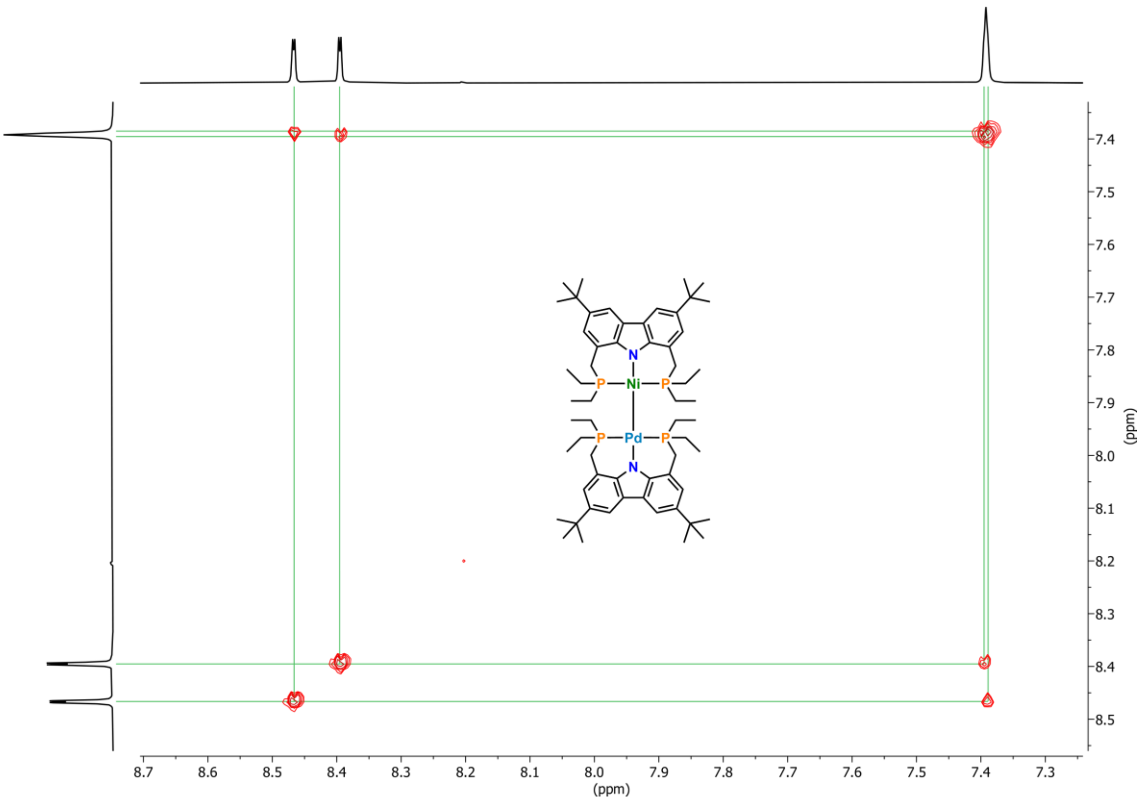

Figure S119. Selected region in the $^{1}H/^{1}H$ COSY NMR spectrum (C_6D_6, 600/600 MHz, 295 K) of **[(^{Et}PNP)Ni-Pd(^{Et}PNP)] (7^{Et}-NiPd)** for clarity.

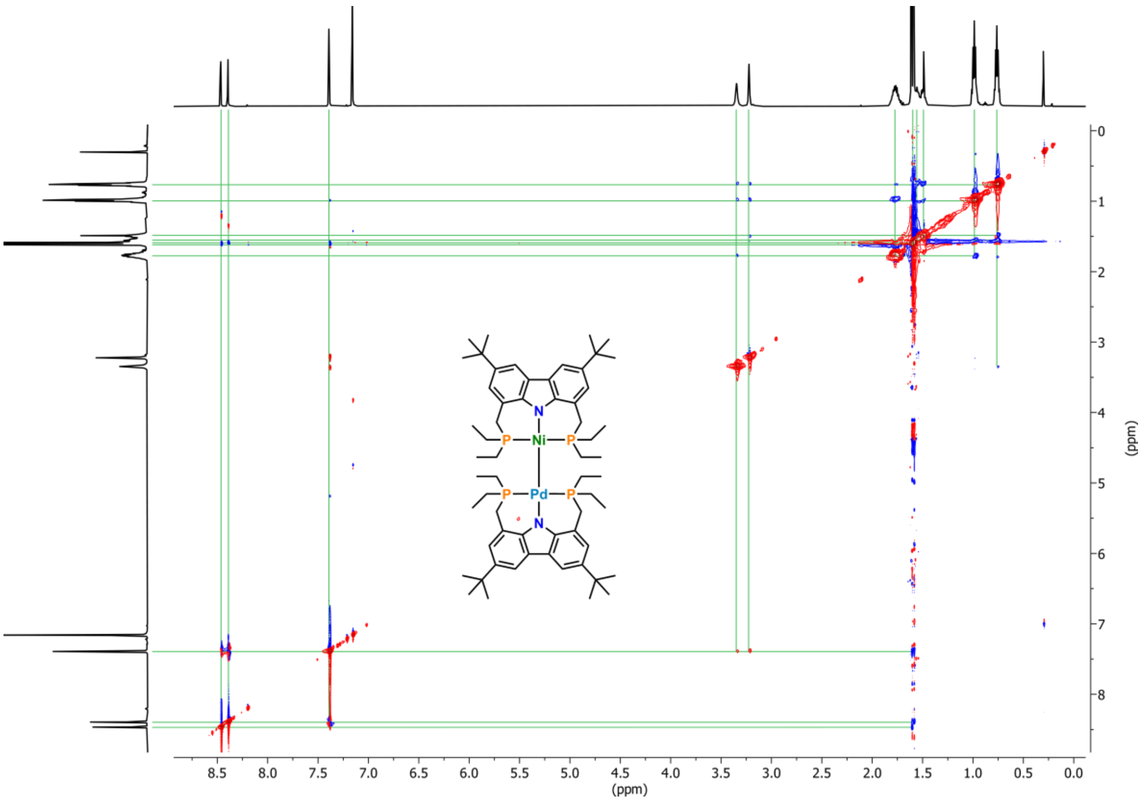

Figure S120. $^{1}H/^{1}H$ NOESY NMR spectrum (C_6D_6, 600/600 MHz, 295 K) of **[(^{Et}PNP)Ni-Pd(^{Et}PNP)] (7^{Et}-NiPd)**.

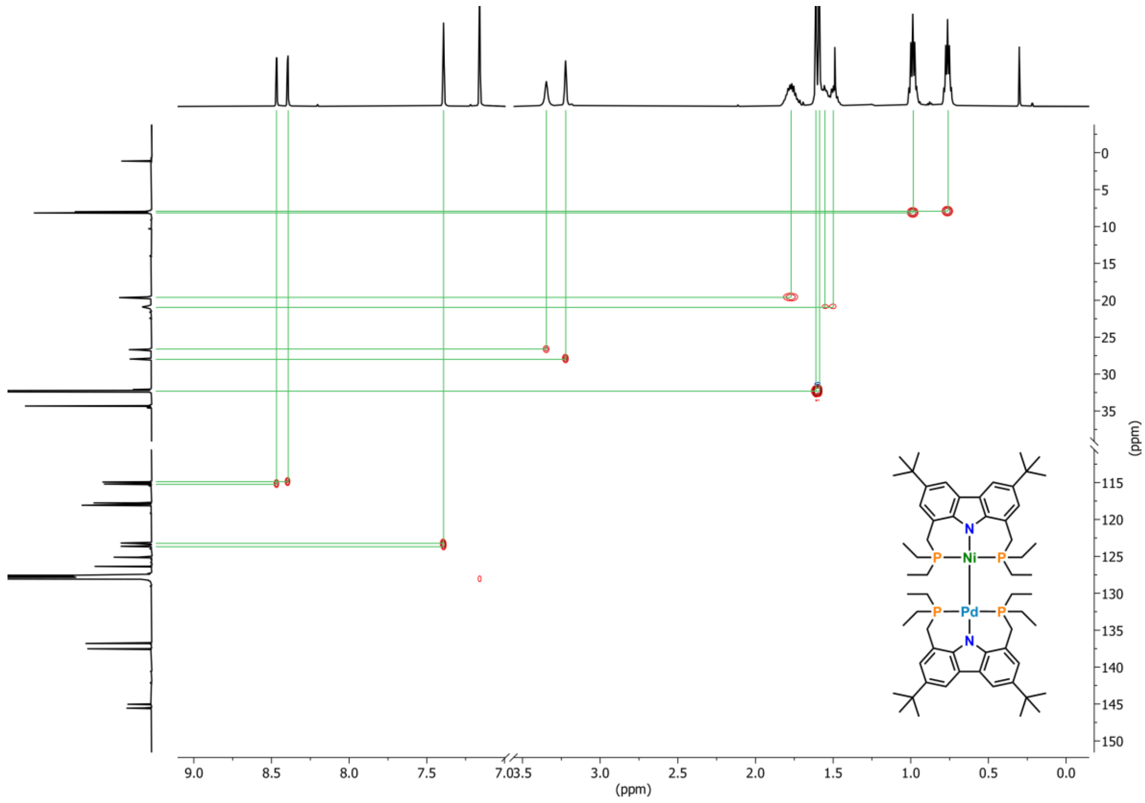

Figure S121. $^{1}H/^{13}C$ HSQC NMR spectrum (C_6D_6, 600/151 MHz, 295 K) of **[(^{Et}PNP)Ni-Pd(^{Et}PNP)] (7^{Et}-NiPd)**.

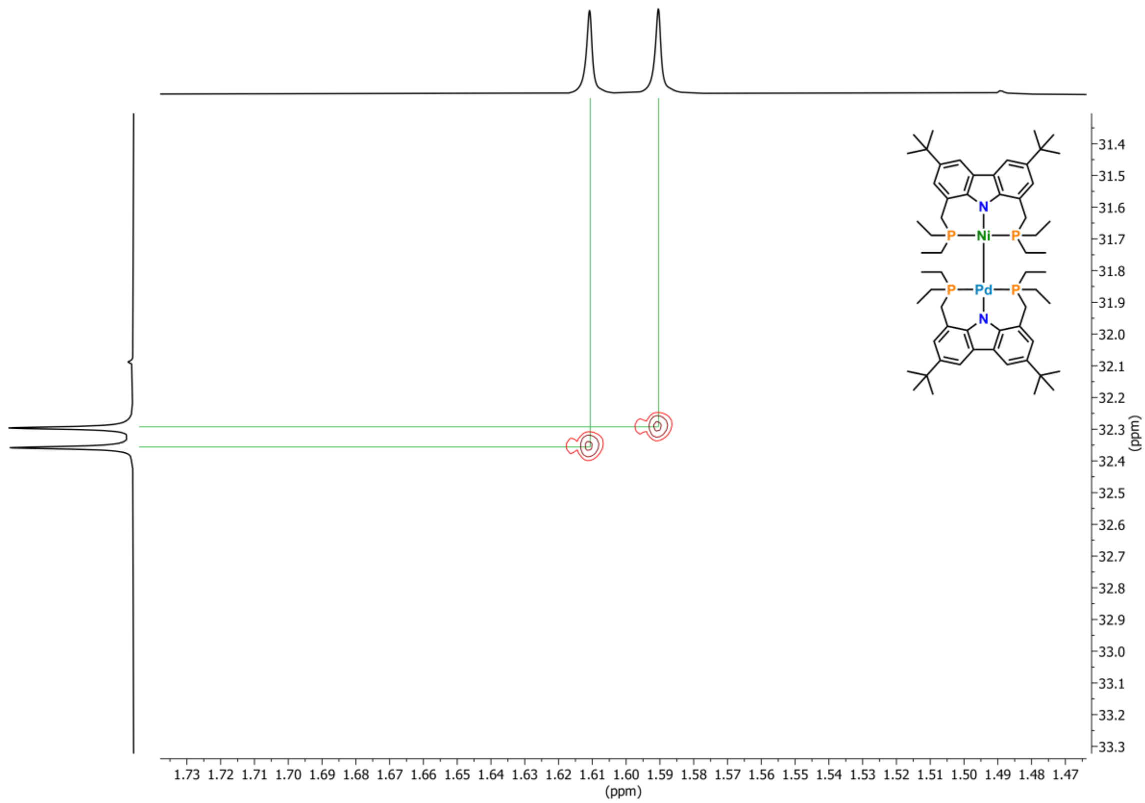

Figure S122. Selected region in the $^{1}H/^{13}C$ HSQC NMR spectrum (C_6D_6, 600/151 MHz, 295 K) of **[(^{Et}PNP)Ni-Pd(^{Et}PNP)] (7^{Et}-NiPd)** for clarity.

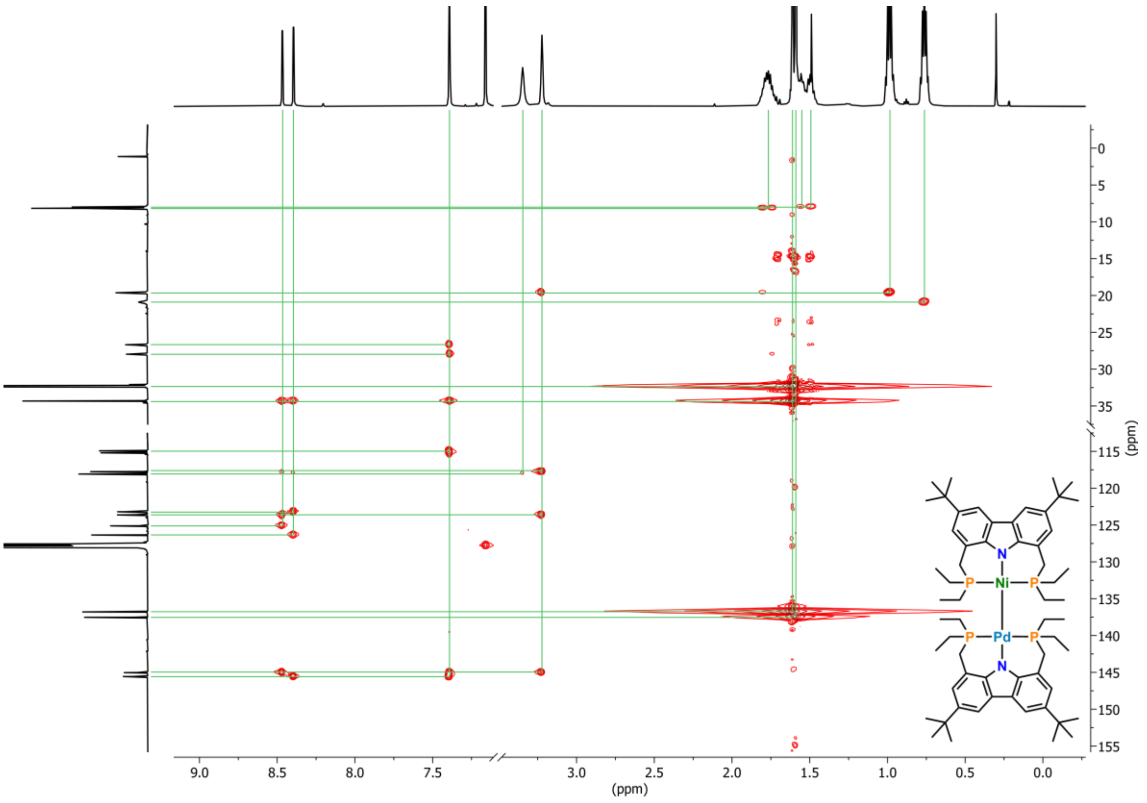

Figure S123. $^1H/^{13}C$ HMBC NMR spectrum (C_6D_6, 600/151 MHz, 295 K) of **[(^{Et}PNP)Ni-Pd(^{Et}PNP)] (7^{Et}-NiPd)**.

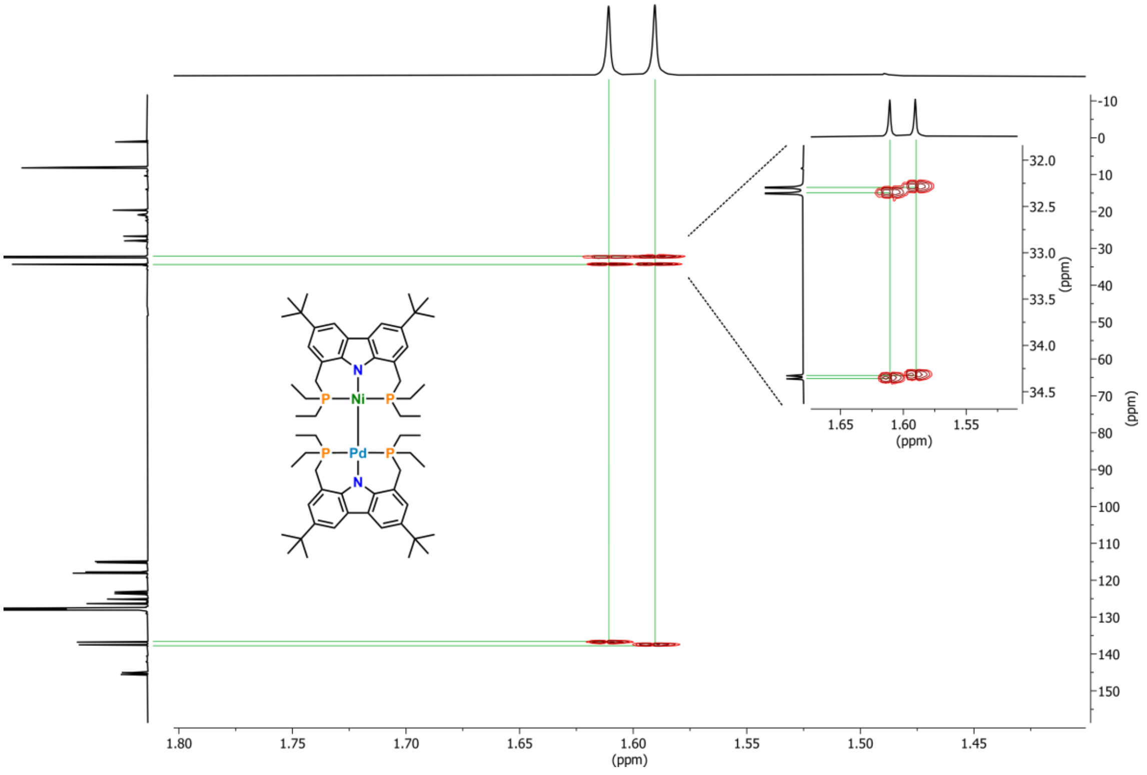

Figure S124. Selected region in the $^1H/^{13}C$ HMBC close NMR spectrum (C_6D_6, 600/151 MHz, 295 K) of **[(^{Et}PNP)Ni-Pd(^{Et}PNP)] (7^{Et}-NiPd)** for clarity.

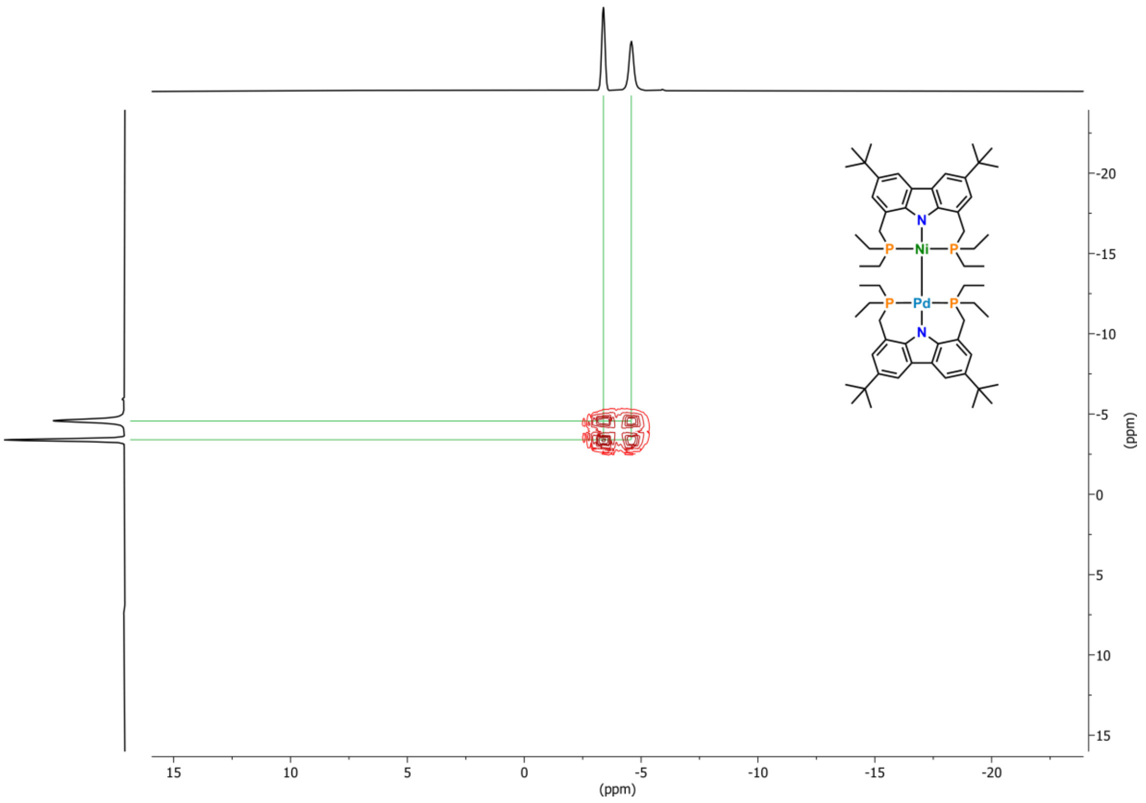

Figure S125. $^{31}P/^{31}P$ COSY NMR spectrum (C_6D_6, 243/243 MHz, 295 K) of **[(^{Et}PNP)Ni-Pd(^{Et}PNP)] (7^{Et}-NiPd)**.

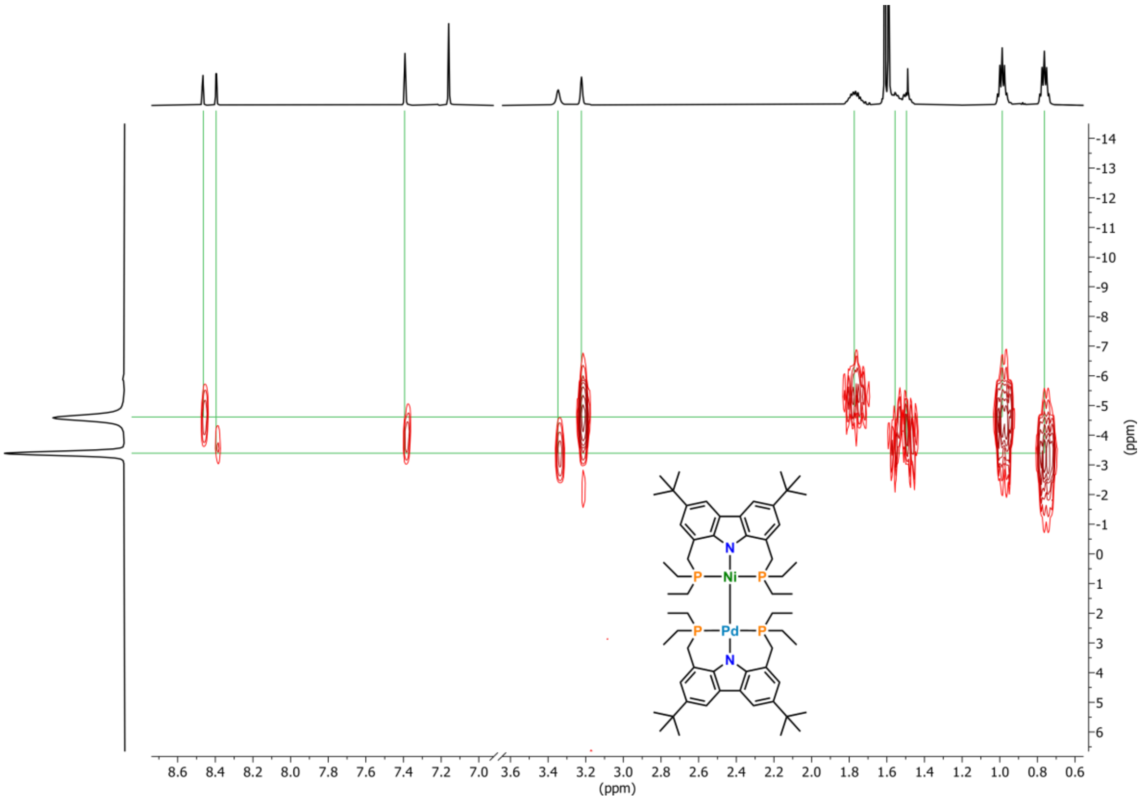

Figure S126. $^{1}H/^{31}P$ HMBC NMR spectrum (C_6D_6, 600/243 MHz, 295 K) of **[(^{Et}PNP)Ni-Pd(^{Et}PNP)] (7^{Et}-NiPd)**.

3) EPR spectroscopy

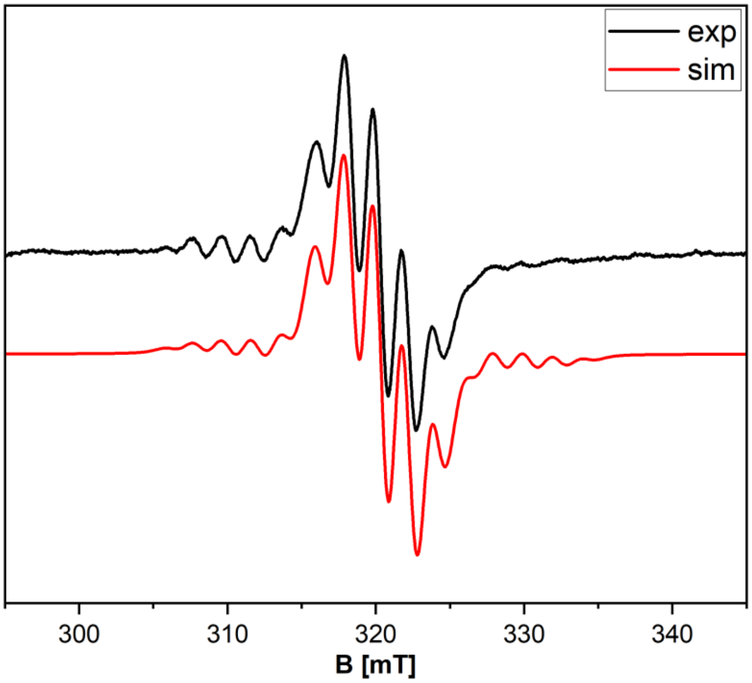

Figure S127. X-band EPR spectrum of **3^{iPr}-Pd** in C_6D_6 at 295 K (black line, frequency = 9.434012 GHz, modulation amplitude = 2.0 G) together with the simulated spectrum (red line). Fitting parameter: g = 2.10407; LWPP = [1.05864 0.763707]; HFC: Pd(22.33% ^{105}Pd, 77.67% ^{106}Pd): 119.771 MHz, ^{31}P: 54.888 MHz, ^{14}N: 54.920 MHz.

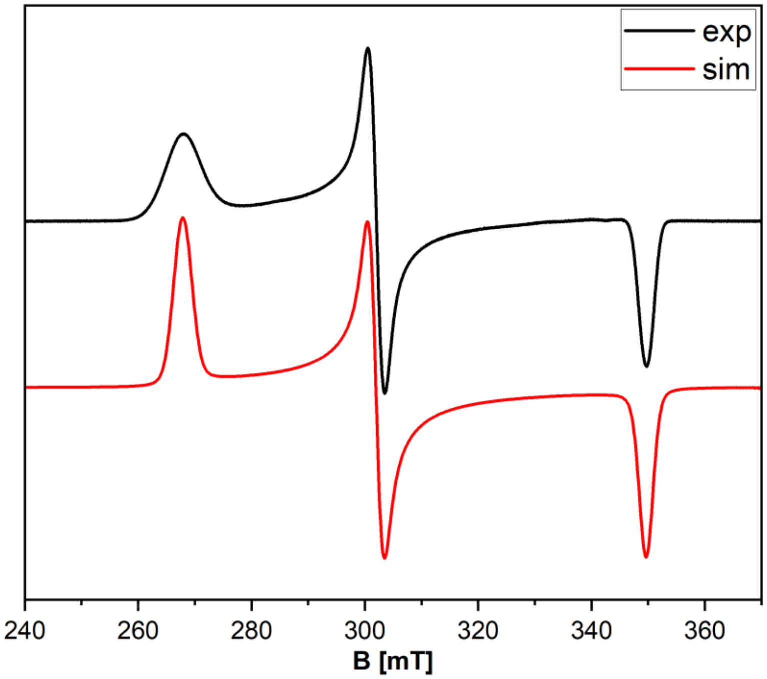

Figure S128. X-band EPR spectrum of **3^{iPr}-Ni** in 2-methyltetrahydrofuran at 6.5 K (black line, frequency = 9.633869 GHz, modulation amplitude = 1.0 G, modulation frequency = 100.0 kHz, microwave power = 0.1994 mW) together with the simulated spectrum (red line). Fitting parameter: g = [2.56927 2.27896 1.96807]; LWPP = [1.87751 0.417409].

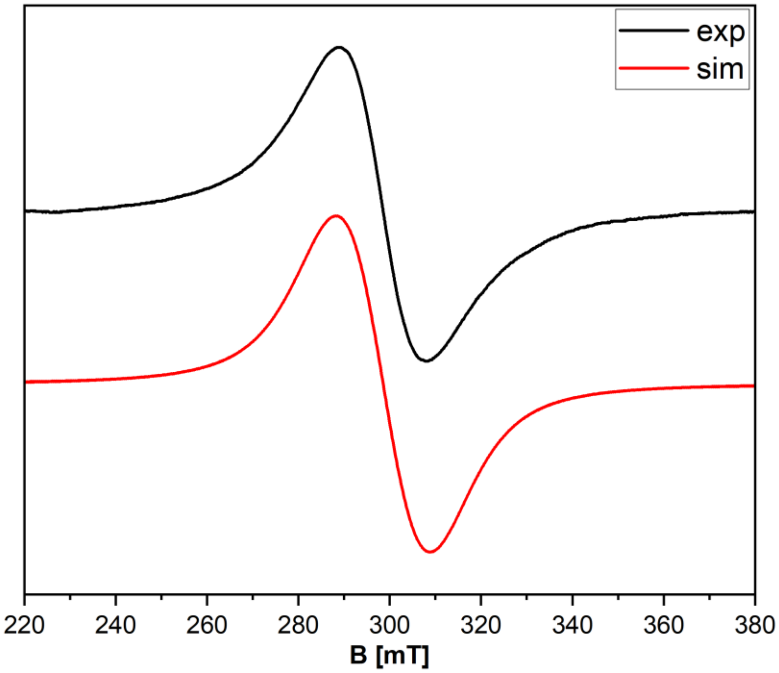

Figure S129.. X-band EPR spectrum of **3^{iPr}-Ni** in C_6D_6 at 295 K (black line, frequency = 9.443080 GHz, modulation amplitude = 2.0 G) together with the simulated spectrum (red line). Fitting parameter: g = 2.25968; LWPP = [13.367 11.8369].

4) UV-VIS spectroscopy

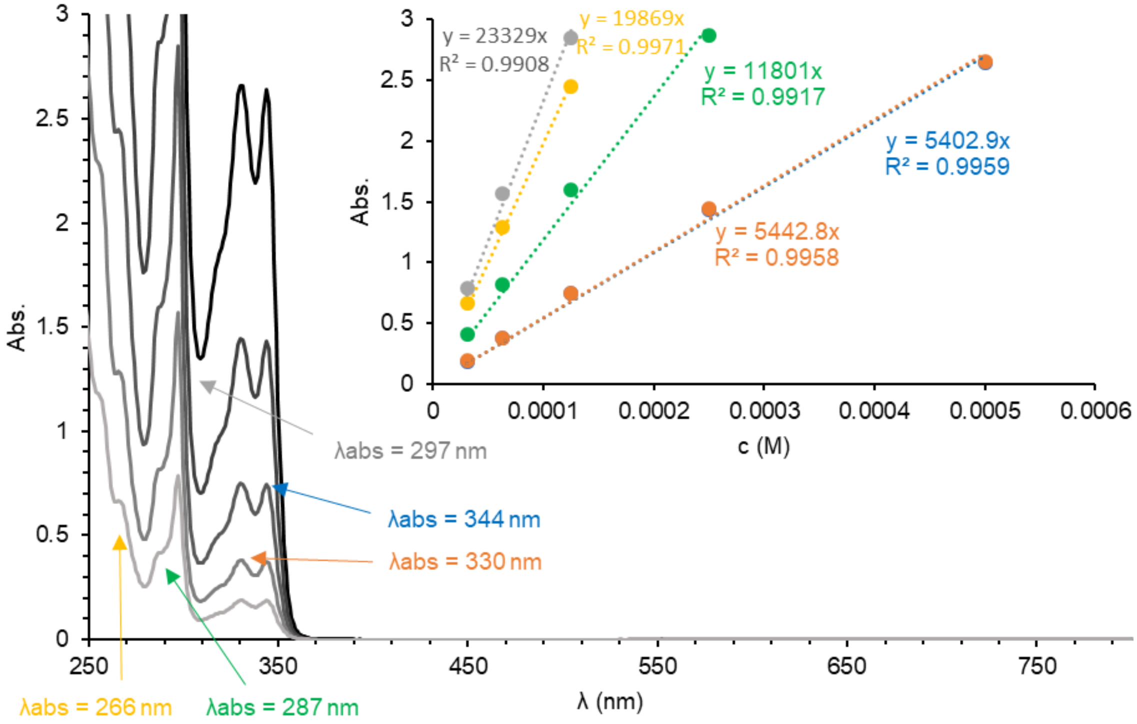

Figure S130. Concentration dependent UV-Vis spectra (*n*-pentane, 295 K) of **(^{Et}PNP)H (1^{Et})**.

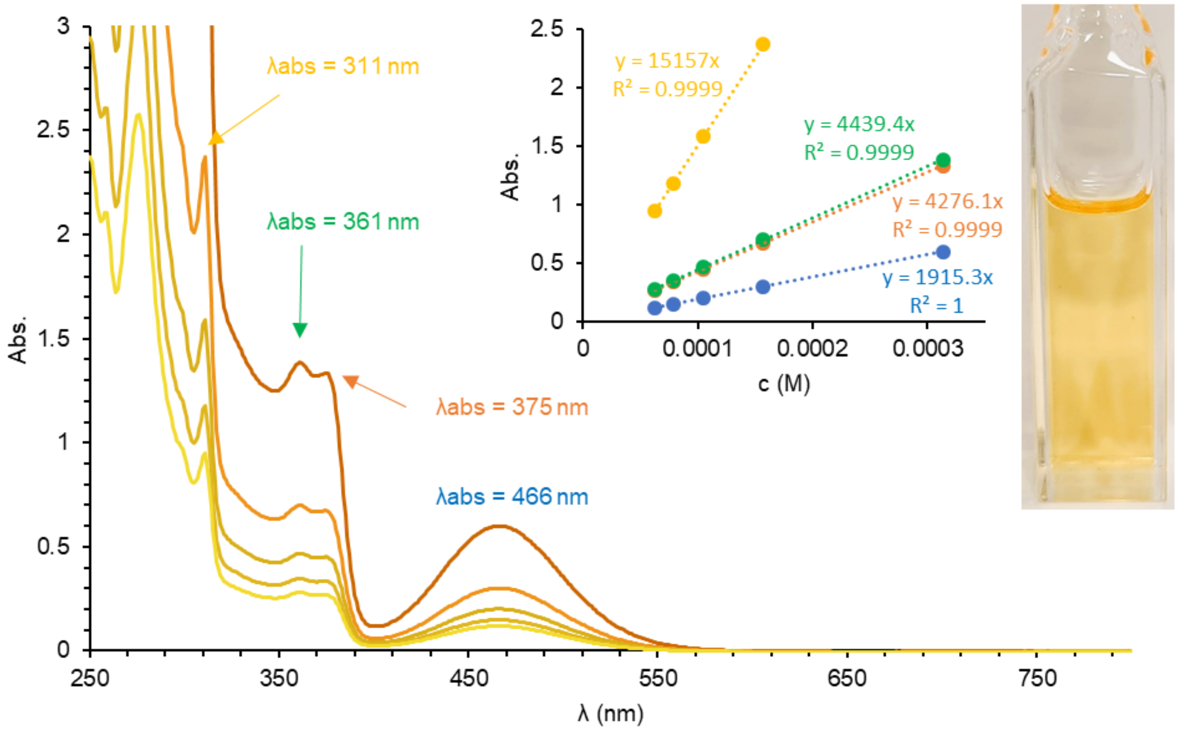

Figure S131. Concentration dependent UV-Vis spectra (THF, 295 K) of **2^{iPr}-Pd**.

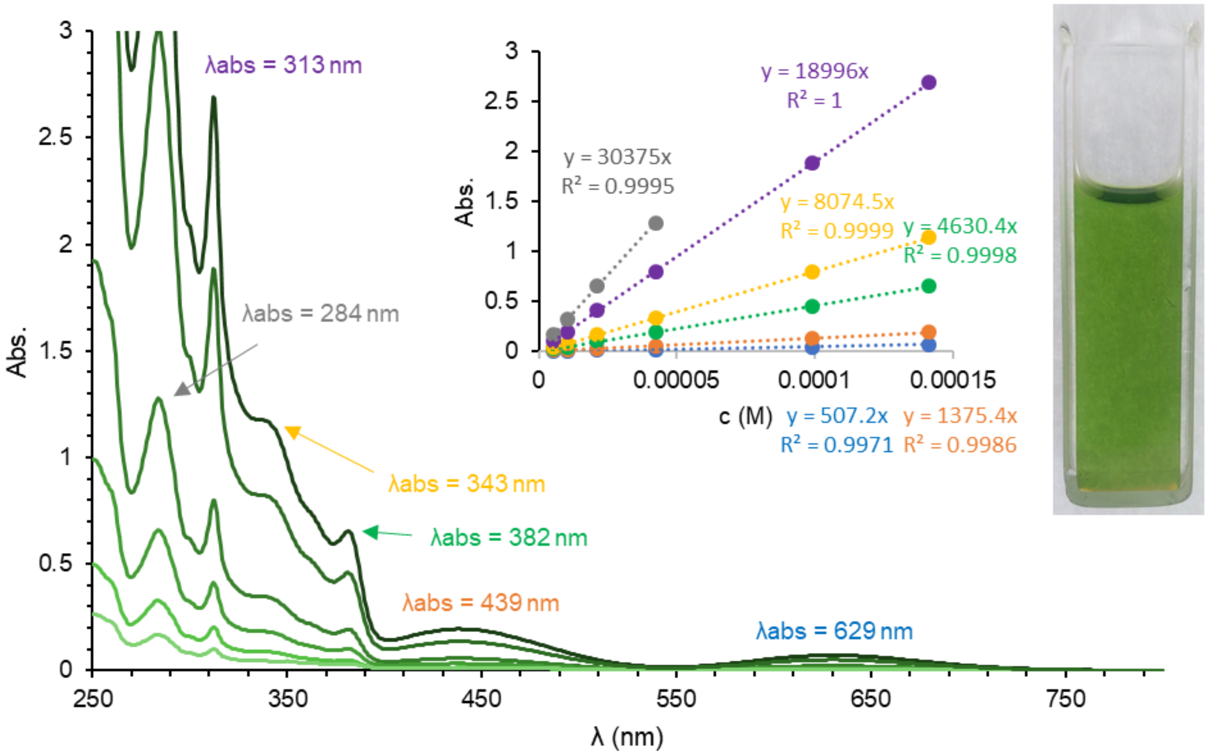

Figure S132. Concentration dependent UV-Vis spectra (*n*-pentane, 295 K) of **2^{iPr}-Ni**.

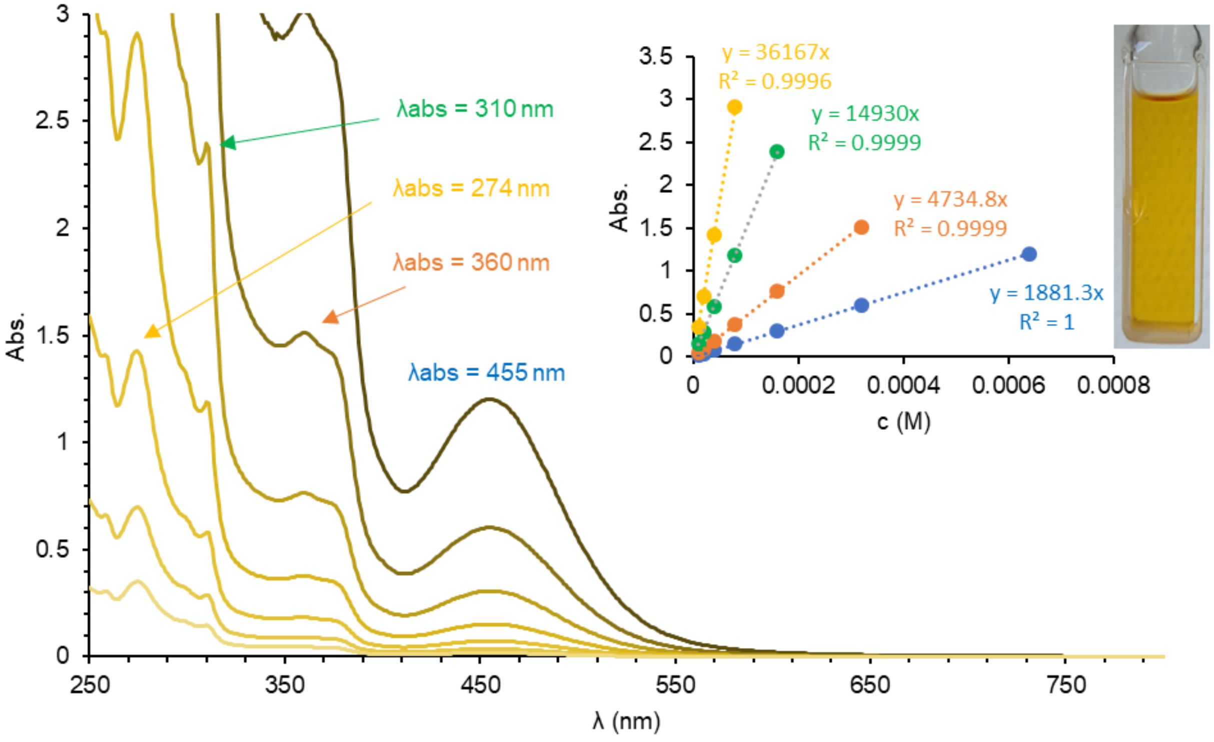

Figure S133. Concentration dependent UV-Vis spectra (THF, 295 K) of **2^{Et}-Pd**.

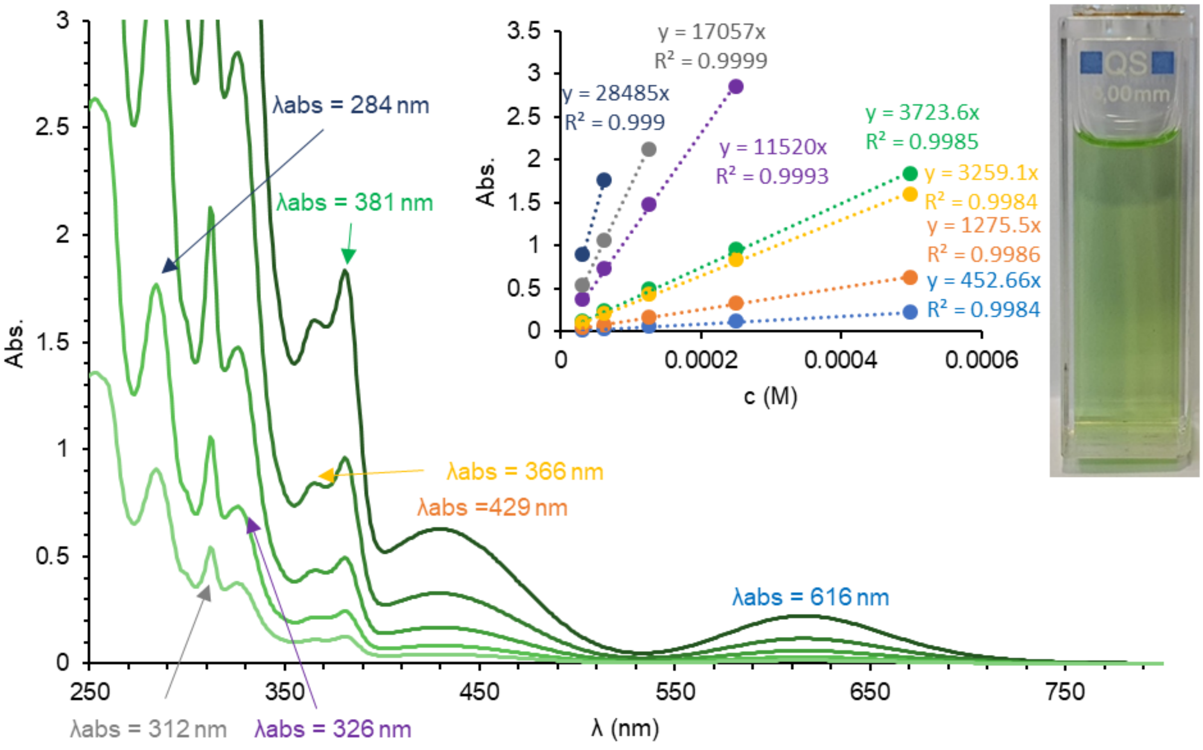

Figure S134. Concentration dependent UV-Vis spectra (*n*-pentane, 295 K) of **2^{Et}-Ni**.

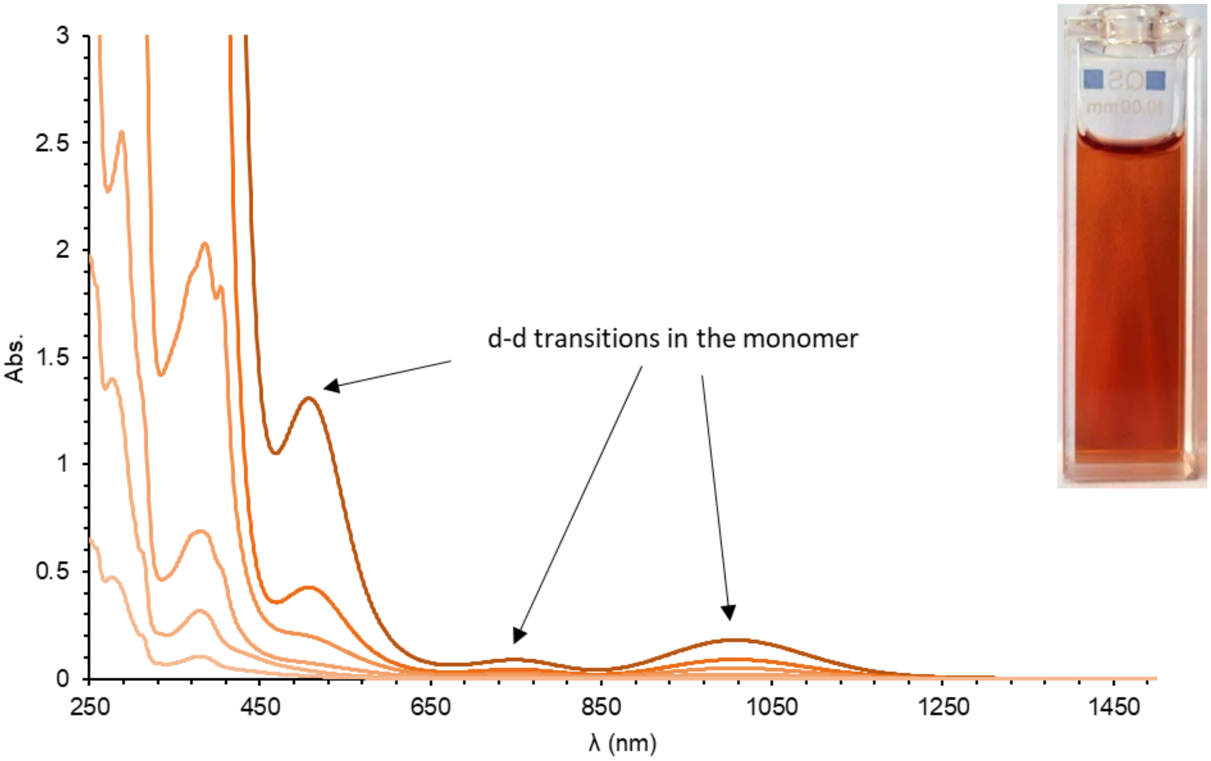

Figure S135. Concentration dependent UV-Vis spectra (THF, 295 K) of **4^{iPr}-Pd**. Concentrations: 700 μM, 233 μM, 140 μM, 47 μM, 23 μM, 8 μM. Due to the dissociation of one dimer into two monomers no extinction coefficients were determinable.

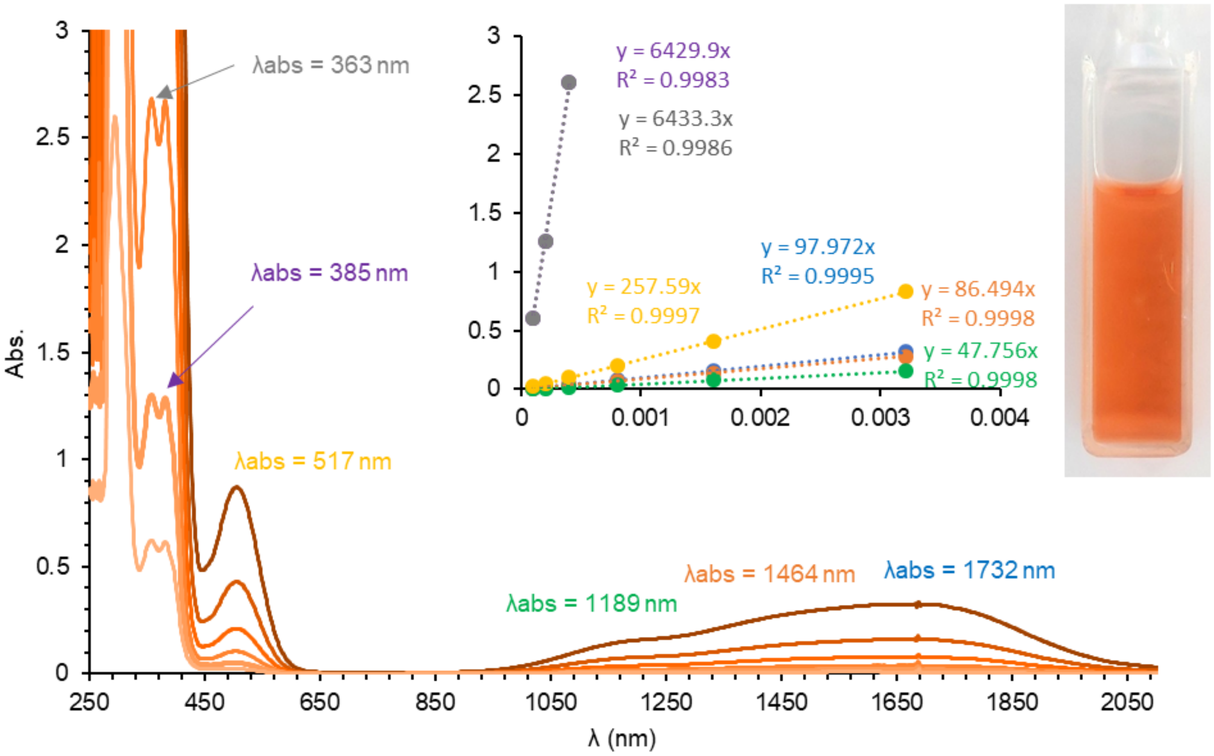

Figure S136. Concentration dependent UV-Vis spectra (*n*-pentane, 295 K) of **3^{iPr}-Ni**.

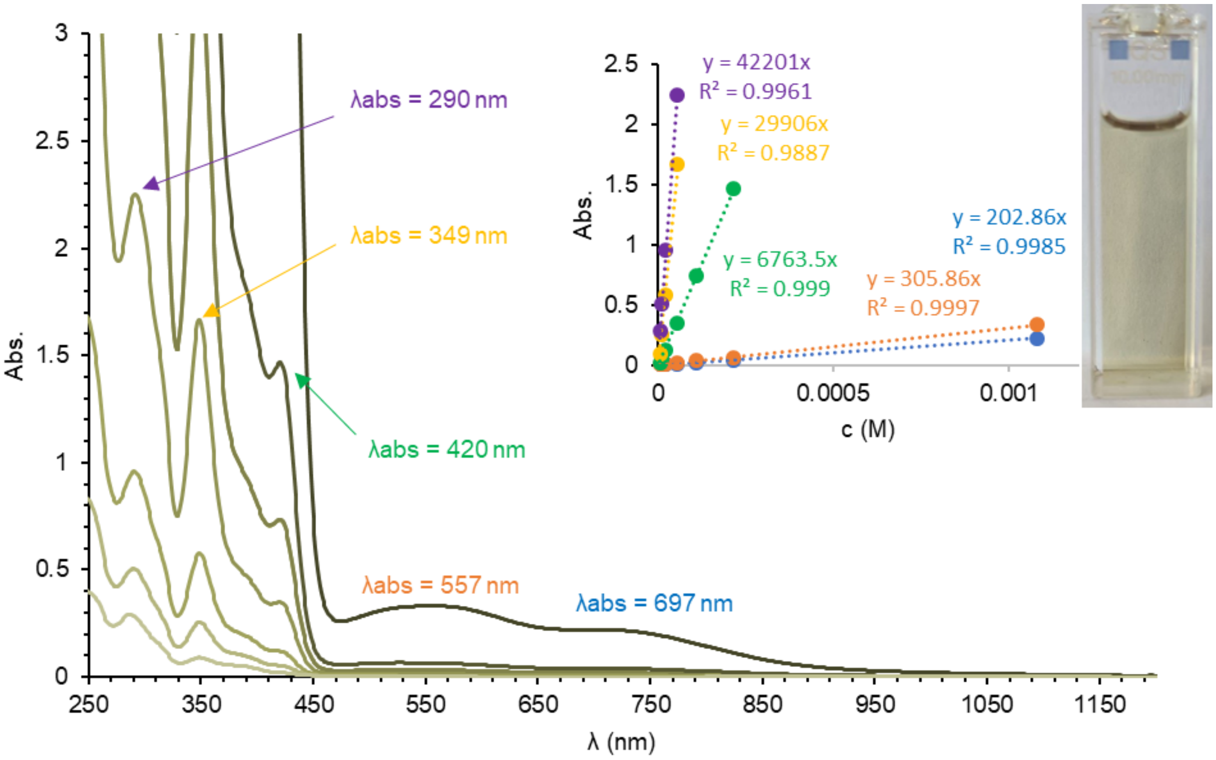

Figure S137. Concentration dependent UV-Vis spectra (THF, 295 K) of **4^{Et}-Ni**.

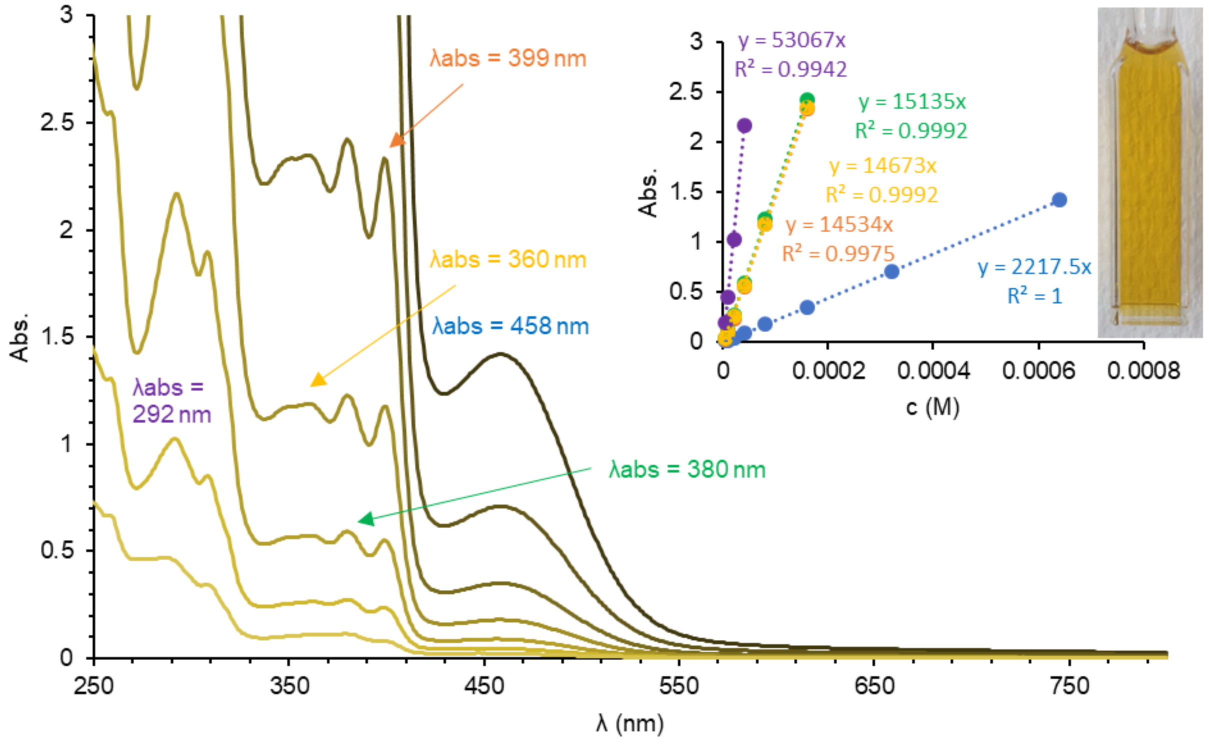

Figure S138. Concentration dependent UV-Vis spectra (THF, 295 K) of **4^{Et}-Pd**.

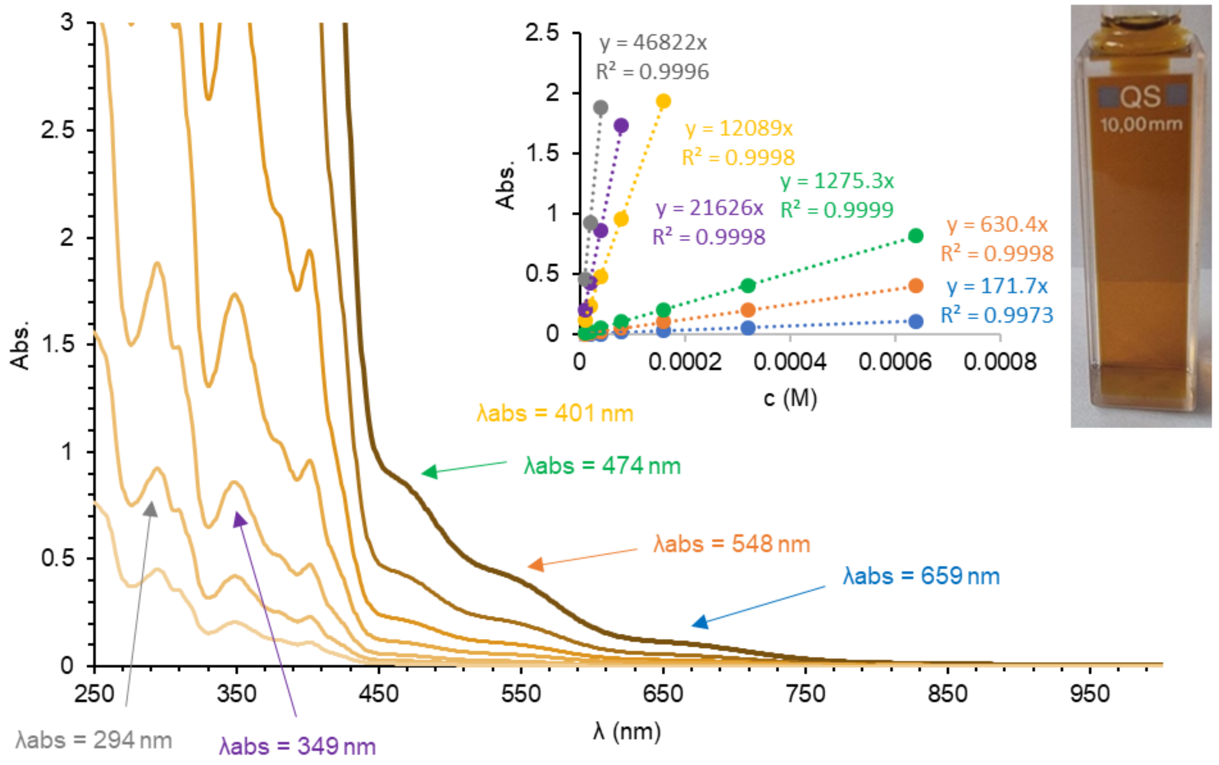

Figure S139. Concentration dependent UV-Vis spectra (THF, 295 K) of **7^{Et}-NiPd**.

5) X-ray characterization data

Crystal data and details of the structure determinations are compiled in Table S1-S13. Full shells of intensity data were collected at 120(1) K with an Agilent Technologies Supernova-E CCD diffractometer (Mo-K_α or Cu-K_α radiation, microfocus X-ray tube, multilayer mirror optics). Detector frames (typically ω-, occasionally φ-scans, scan width 0.5° for Mo and 1.0° for Cu) were integrated by profile fitting.[6] Data were corrected for air and detector absorption, Lorentz and polarization effects[7-8] and scaled essentially by application of appropriate spherical harmonic functions.[9-11] Absorption by the crystal was treated numerically (Gaussian grid).[11-12] An illumination correction was performed as part of the numerical absorption correction.[11]

Using OLEX2,[13] the structures were solved with SHELXT[14-16] (intrinsic phasing) and refined with SHELXL[16-18] by full-matrix least squares methods based on F^2 against all unique reflections. All non-hydrogen atoms were given anisotropic displacement parameters. Hydrogen atoms were generally input at calculated positions and refined with a riding model.[19-22] Split atom models were used to refine disordered groups and/or solvent molecules. When found necessary, suitable geometry and adp restraints were applied.[19-23]

Crystals of **ga_tbr33** and **ga_vk1** were refined as twins (twin laws: –1 0 0, 0 –1 0, 0 0 –1 for **ga_tbr33** and –1 0 0, 0 –1 0, 0 0 1 for **ga_vk1**) with a batch scale factor (BASF) of 0.024(11) and 0.4887(10), respectively. Crystals of **ga_tbr9** were twinned in $P\bar{1}$ (180° around 0 1 0, two components with BASF = 0.4340(8). The structure was solved in hklf4 format (SHELXT) and refined in hklf5 format (SHELXL). Due to severe disorder, electron density attributed to co-crystallized solvent molecules were removed from the structures of **ga_tbr9** (1.5 Et_2O molecules per moiety formula in two voids), **ga_tbr10** (2.0 toluene molecules per moiety formula), **ga_tbr75** (2.0 THF molecules per moiety formula), **ga_vk5** (1.0 toluene molecules per moiety formula), **ga_vk7** (0.5 benzene molecules per moiety formula) and **ga_vk10** (1.0 benzene molecules per moiety formula) using OLEX2 solvent masks.[24] Positional disorder of Ni and Pd in **ga_vk10** was modeled with one free variable (occupancy 0.473(2) PdB, 0.527(2) PdA).

CCDC 2470075 - 2470087 contains the supplementary crystallographic data for this paper. These data can be obtained free of charge from the Cambridge Crystallographic Data Centre's and FIZ Karlsruhe's joint Access Service *via* https://www.ccdc.cam.ac.uk.

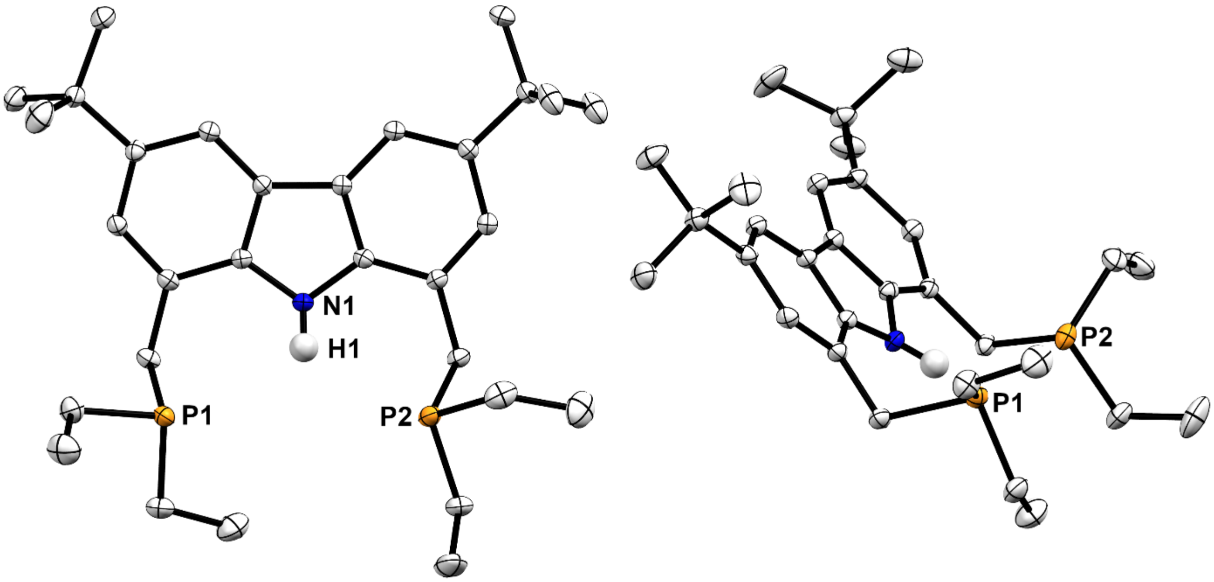

Figure S140. Molecular crystal structure of **1**^{Et} (left: top view, right: view from the side). Ellipsoids shown with 50% probability. Hydrogen atoms (except H1) are omitted for clarity.

Table S1. Crystal data and structure refinement for **1**^{Et}.

Empirical formula	$C_{30}H_{47}NP_2$
Formula weight	483.62
Temperature [K]	120(1)
Crystal system	triclinic
Space group (number)	$P\bar{1}$ (2)
a [Å]	10.1598(2)
b [Å]	12.5986(2)
c [Å]	12.9739(2)
α [°]	107.9000(10)
β [°]	109.360(2)
γ [°]	94.5250(10)
Volume [Å^3]	1460.37(5)
Z	2
ρ_{calc} [gcm^{-3}]	1.100
μ [mm^{-1}]	0.166
transmission factors (max, min)	1.000, 0.663
F(000)	528
Radiation	Mo K_α (λ=0.71073 Å)
2θ range [°]	4.98 to 68.39 (0.63 Å)
Index ranges	−15 ≤ h ≤ 15, −19 ≤ k ≤ 19, −20 ≤ l ≤ 20
Reflections collected	47670
Independent reflections	11510 (R_{int} = 0.0535)
observed [I≥2σ(I)]	8687
Completeness to θ = 25.242°	99.9 %
Data / Restraints / Parameters	11510 / 54 / 343
Goodness-of-fit on F^2	1.040
Final *R* indexes [I≥2σ(I)]	R_1 = 0.0463 wR_2 = 0.1056
Final *R* indexes [all data]	R_1 = 0.0686 wR_2 = 0.1173
Largest peak/hole [eÅ^{-3}]	0.43/−0.34
CCDC number	2470075

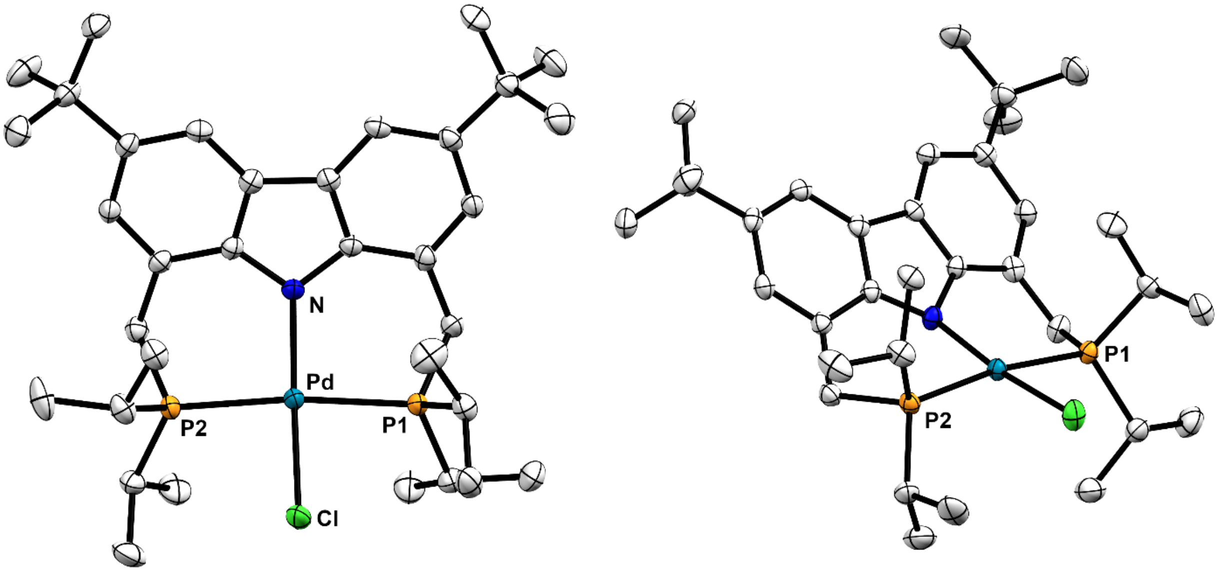

Figure S141. Molecular crystal structure of **2^{iPr}-Pd** (left: top view, right: view from the side). Ellipsoids shown with 50% probability. Hydrogen atoms are omitted for clarity. Selected bond parameters in [Å] and [°]: Pd–N 2.080(3); Pd–P1 2.3183(15); Pd–P2 2.3059(14); Pd–Cl 2.3148(12); N1–Pd–Cl 173.59(9); P2–Pd–P1 171.62(4).

Table S2. Crystal data and structure refinement for **2^{iPr}-Pd**.

Empirical formula	$C_{34}H_{54}ClNP_2Pd$
Formula weight	680.57
Temperature [K]	120(1)
Crystal system	orthorhombic
Space group (number)	$Pna2_1$ (33)
a [Å]	11.49970(10)
b [Å]	23.5440(3)
c [Å]	12.9846(2)
α [°]	90
β [°]	90
γ [°]	90
Volume [Å^3]	3515.57(8)
Z	4
ρ_{calc} [gcm^{-3}]	1.286
μ [mm^{-1}]	5.969
transmission factors (max, min)	0.976, 0.549
F(000)	1432
Radiation	Cu K_α (λ=1.54184 Å)
2θ range [°]	7.78 to 142.03 (0.82 Å)
Index ranges	−13 ≤ h ≤ 14, −28 ≤ k ≤ 28, −13 ≤ l ≤ 15
Reflections collected	89003
Independent reflections	6505 (R_{int} = 0.0958)
observed [$I \geq 2\sigma(I)$]	6251
Completeness to θ = 67.684°	99.9 %
Data / Restraints / Parameters	6505 / 1 / 367
Goodness-of-fit on F^2	1.044
Final *R* indexes [$I \geq 2\sigma(I)$]	R_1 = 0.0327 wR_2 = 0.0844
Final *R* indexes [all data]	R_1 = 0.0342 wR_2 = 0.0858
Largest peak/hole [eÅ^{-3}]	0.53/−0.72
CCDC number	2470076

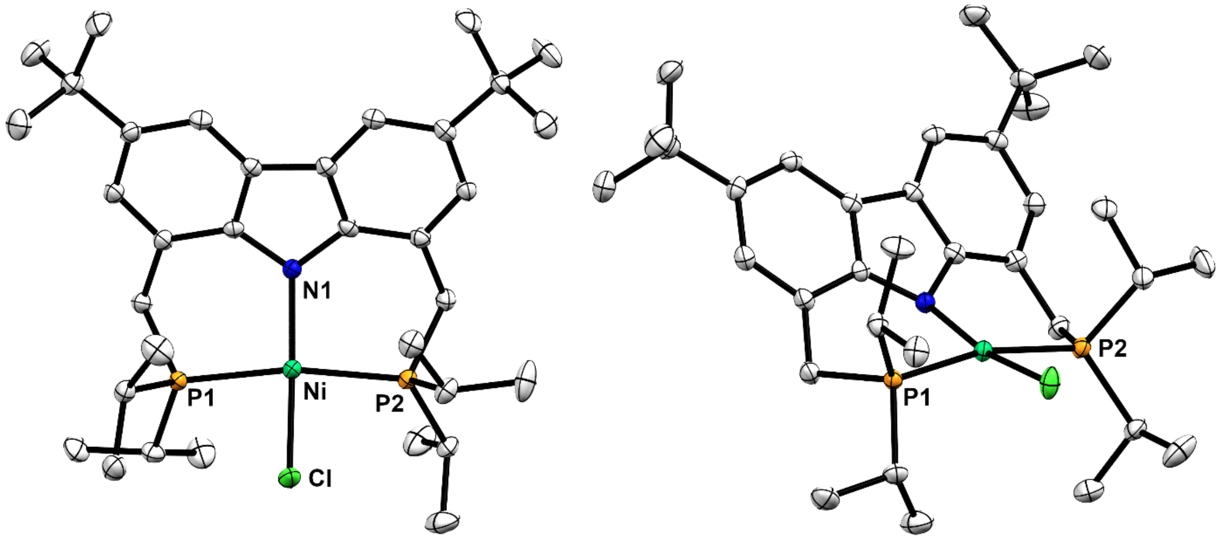

Figure S142. Molecular crystal structure of **2[iPr]-Ni** (left: top view, right: view from the side). Ellipsoids shown with 50% probability. Hydrogen atoms are omitted for clarity. Selected bond parameters in [Å] and [°]: Ni–N1 1.9315(16); Ni–P1 2.2116(6); Ni–P2 2.2238(6); Ni–Cl 2.1914(6); N1–Ni–Cl 167.54(5); P1–Ni–P2 166.75(2).

Table S3. Crystal data and structure refinement for **2[iPr]-Ni**.

Empirical formula	$C_{34}H_{54}ClNNiP_2$
Formula weight	632.88
Temperature [K]	120(1)
Crystal system	orthorhombic
Space group (number)	$Pna2_1$ (33)
a [Å]	11.35819(6)
b [Å]	23.67846(12)
c [Å]	12.91699(6)
α [°]	90
β [°]	90
γ [°]	90
Volume [Å^3]	3473.95(3)
Z	4
ρ_{calc} [gcm^{-3}]	1.210
μ [mm^{-1}]	2.536
transmission factors (max, min)	1.000, 0.850
F(000)	1360
Radiation	Cu K_α (λ=1.54184 Å)
2θ range [°]	7.80 to 142.30 (0.81 Å)
Index ranges	−13 ≤ h ≤ 13, −28 ≤ k ≤ 28, −15 ≤ l ≤ 15
Reflections collected	121855
Independent reflections	6686 (R_{int} = 0.0321)
observed [I≥2σ(I)]	6594
Completeness to θ = 67.684°	100.0 %
Data / Restraints / Parameters	6686 / 1 / 366
Goodness-of-fit on F^2	1.034
Final *R* indexes [I≥2σ(I)]	R_1 = 0.0198 wR_2 = 0.0528
Final *R* indexes [all data]	R_1 = 0.0201 wR_2 = 0.0530
Largest peak/hole [eÅ^{-3}]	0.20/−0.27
CCDC number	2470077

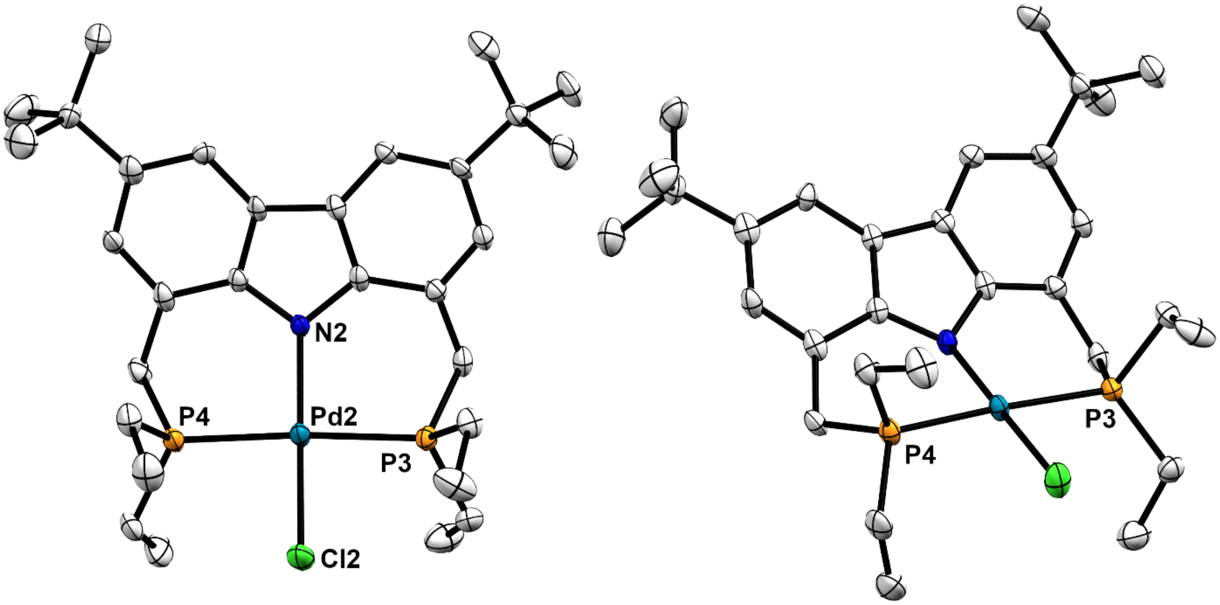

Figure S143. Molecular crystal structure of $\mathbf{2^{Et}}$**-Pd** (left: top view, right: side view), showing one of two crystallographically independent molecules (conformers) in the cell. Ellipsoids shown with 50% probability. Hydrogen atoms and a second conformer are omitted for clarity. Selected bond parameters in [Å] and [°]: Pd2–N2 2.056(6); Pd2–P3 2.2987(17); Pd2–P4 2.3013(19); Pd2–Cl2 2.307(2); N2–Pd2–Cl2 179.23(17); P4–Pd2–P3 176.17(7).

Table S4. Crystal data and structure refinement for $\mathbf{2^{Et}}$**-Pd**.

Empirical formula	$C_{30}H_{46}ClNP_2Pd$
Formula weight	624.47
Temperature [K]	120(1)
Crystal system	monoclinic
Space group (number)	$P2_1$ (4)
a [Å]	12.0399(5)
b [Å]	11.8818(3)
c [Å]	21.3386(6)
α [°]	90
β [°]	90.059(3)
γ [°]	90
Volume [Å^3]	3052.61(17)
Z	4
ρ_{calc} [gcm^{-3}]	1.359
μ [mm^{-1}]	0.819
transmission factors (max, min)	1.000, 0.760
F(000)	1304
Radiation	Mo K_α (λ=0.71073 Å)
2θ range [°]	5.13 to 68.46 (0.63 Å)
Index ranges	−18 ≤ h ≤ 19, −18 ≤ k ≤ 18, −33 ≤ l ≤ 32
Reflections collected	90481
Independent reflections	24132 (R_{int} = 0.0736)
observed [I≥2σ(I)]	20204
Completeness to θ = 25.242°	99.8 %
Data / Restraints / Parameters	24132 / 1 / 652
Goodness-of-fit on F^2	1.045
Final *R* indexes [I≥2σ(I)]	R_1 = 0.0498 wR_2 = 0.0886
Final *R* indexes [all data]	R_1 = 0.0687 wR_2 = 0.0973
Largest peak/hole [eÅ^{-3}]	0.94/−1.35
CCDC number	2470078

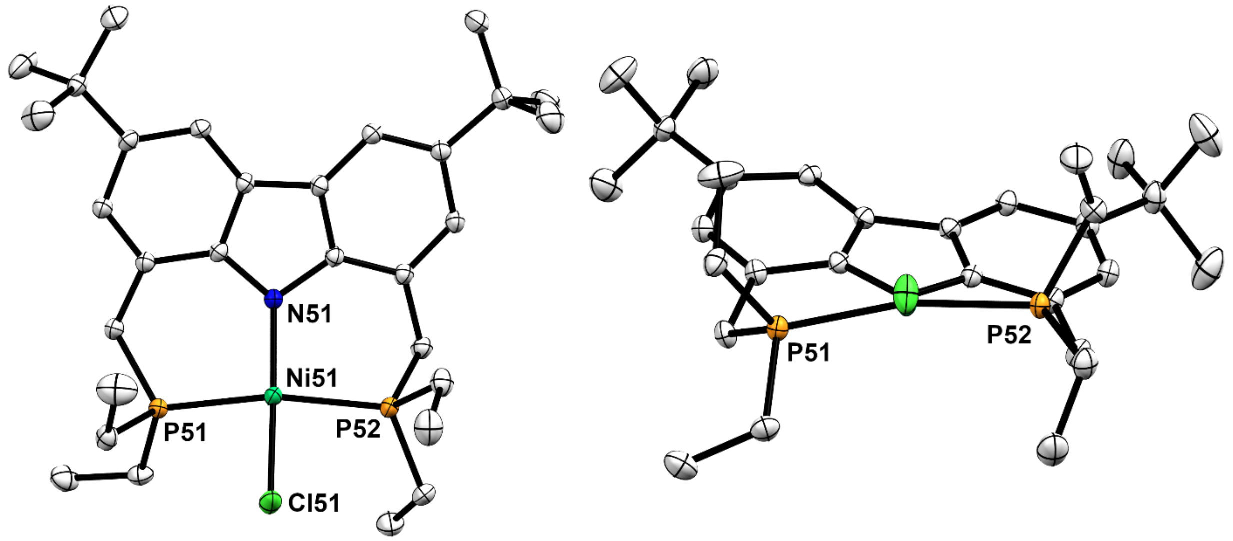

Figure S144. Molecular crystal structure of **2^{Et}-Ni** (left: top view, right: view along the Cl–Ni–N axis), showing one of two crystallographically independent molecules (conformers) in the cell. Ellipsoids shown with 50% probability. Hydrogen atoms and a second conformer are omitted for clarity. Selected bond parameters in [Å] and [°]: Ni51–N51 1.9213(17); Ni51–P51 2.1965(6); Ni51–P52 2.2128(6); Ni51–Cl51 2.1906(6); N51–Ni51–Cl51 173.08(5); P51–Ni51–P52 165.59(2).

Table S5. Crystal data and structure refinement for **2^{Et}-Ni**.

Empirical formula	$C_{30}H_{46}ClNNiP_2$
Formula weight	576.78
Temperature [K]	120(1)
Crystal system	orthorhombic
Space group (number)	$Pna2_1$ (33)
a [Å]	21.1501(2)
b [Å]	23.9942(3)
c [Å]	11.83213(13)
α [°]	90
β [°]	90
γ [°]	90
Volume [Å^3]	6004.58(12)
Z	8
ρ_{calc} [gcm^{-3}]	1.276
μ [mm^{-1}]	0.861
transmission factors (max, min)	1.000, 0.627
F(000)	2464
Radiation	Mo K_α (λ=0.71073 Å)
2θ range [°]	5.13 to 68.42 (0.63 Å)
Index ranges	−33 ≤ h ≤ 33, −36 ≤ k ≤ 37, −18 ≤ l ≤ 18
Reflections collected	198005
Independent reflections	24075 (R_{int} = 0.0677)
observed [I≥2σ(I)]	19960
Completeness to θ = 25.242°	99.9 %
Data / Restraints / Parameters	24075 / 1 / 651
Goodness-of-fit on F^2	1.034
Final *R* indexes [I≥2σ(I)]	R_1 = 0.0351 wR_2 = 0.0730
Final *R* indexes [all data]	R_1 = 0.0503 wR_2 = 0.0791
Largest peak/hole [eÅ^{-3}]	0.33/−0.42
Flack parameter	-0.006(3)
CCDC number	2470079

Table S6. Crystal data and structure refinement for **3^{iPr}-Ni**.

Empirical formula	$C_{34}H_{54}NNiP_2$
Formula weight	597.43
Temperature [K]	120(1)
Crystal system	orthorhombic
Space group (number)	$Pbca$ (61)
a [Å]	12.41166(13)
b [Å]	22.9026(2)
c [Å]	23.1536(2)
α [°]	90
β [°]	90
γ [°]	90
Volume [$Å^3$]	6581.61(12)
Z	8
ρ_{calc} [gcm^{-3}]	1.206
μ [mm^{-1}]	0.709
transmission factors (max, min)	1.000, 0.701
F(000)	2584
Radiation	Mo K_α (λ=0.71073 Å)
2θ range [°]	4.81 to 68.53 (0.63 Å)
Index ranges	$-19 \le h \le 19$, $-36 \le k \le 35$, $-36 \le l \le 36$
Reflections collected	198230
Independent reflections	13429 (R_{int} = 0.0656)
observed [$I \geq 2\sigma(I)$]	10717
Completeness to θ = 25.242°	99.9 %
Data / Restraints / Parameters	13429 / 0 / 357
Goodness-of-fit on F^2	1.045
Final *R* indexes [$I \geq 2\sigma(I)$]	R_1 = 0.0348 wR_2 = 0.0794
Final *R* indexes [all data]	R_1 = 0.0516 wR_2 = 0.0856
Largest peak/hole [$eÅ^{-3}$]	0.43/−0.28
CCDC number	2470080

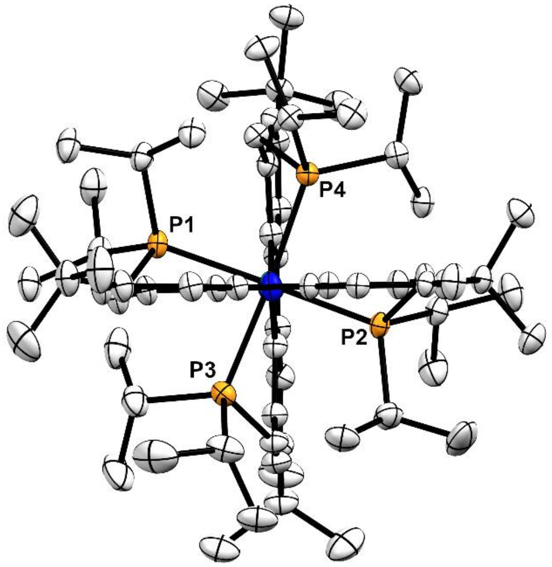

Figure S145. Molecular crystal structure of **4[iPr]-Pd** (view along the N1–Pd1–Pd2–N2 axis; for side view see main article Figure 1b). Ellipsoids shown with 50% probability. Hydrogen atoms, two co-crystallized disordered THF molecules and one disordered ^{t}Bu-group are omitted for clarity.

Table S7. Crystal data and structure refinement for **4[iPr]-Pd**.

Empirical formula	$C_{84}H_{140}N_2O_4P_4Pd_2$
Formula weight	1578.65
Temperature [K]	120(1)
Crystal system	monoclinic
Space group (number)	$P2_1/c$ (14)
a [Å]	16.65648(7)
b [Å]	21.91916(7)
c [Å]	22.20540(7)
α [°]	90
β [°]	91.1451(3)
γ [°]	90
Volume [Å^3]	8105.49(5)
Z	4
ρ_{calc} [gcm^{-3}]	1.294
μ [mm^{-1}]	4.694
transmission factors (max, min)	0.893, 0.640
F(000)	3368
Radiation	Cu K_α (λ=1.54184 Å)
2θ range [°]	5.67 to 141.69 (0.82 Å)
Index ranges	−19 ≤ h ≤ 20, −26 ≤ k ≤ 26, −27 ≤ l ≤ 27
Reflections collected	249939
Independent reflections	15566 (R_{int} = 0.0729)
observed [I≥2σ(I)]	14510
Completeness to θ = 67.684°	100.0 %
Data / Restraints / Parameters	15566 / 348 / 899
Goodness-of-fit on F^2	1.039
Final *R* indexes [I≥2σ(I)]	R_1 = 0.0326 wR_2 = 0.0846
Final *R* indexes [all data]	R_1 = 0.0349 wR_2 = 0.0861
Largest peak/hole [eÅ^{-3}]	0.69/−0.78
CCDC number	2470081

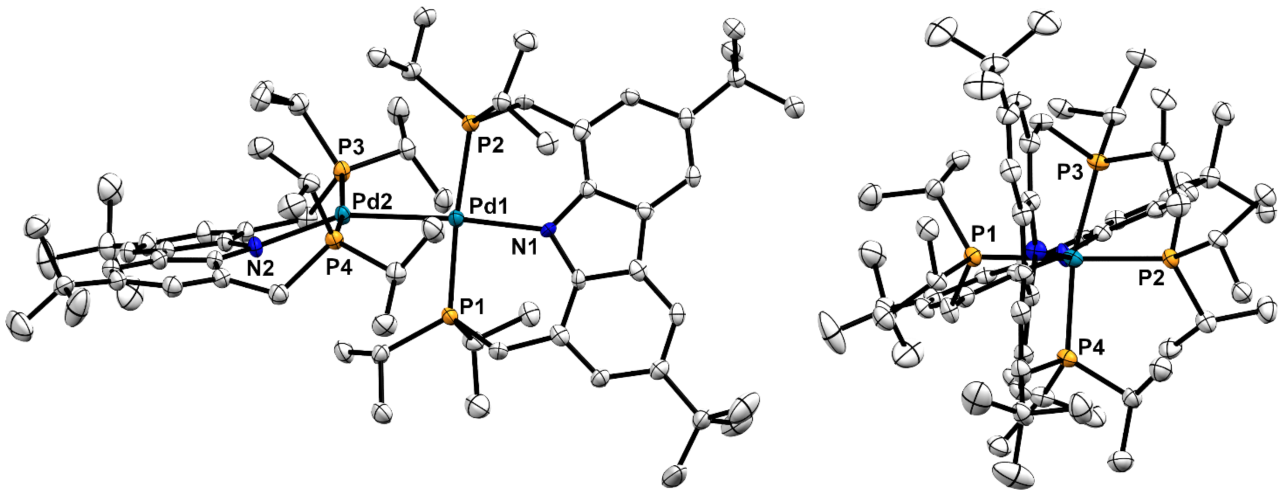

Figure S146. Molecular crystal structure of **4^{iPr}-Pd** exhibiting a different conformation (left: side view, right: view along the Pd2–Pd1 axis). Ellipsoids shown with 50% probability. Hydrogen atoms are omitted for clarity. Selected bond parameters in [Å] and [°] (mean values): Pd1–Pd2 2.6752(4); Pd1–N1 2.179(4); Pd1–P1 2.3453(13); Pd1–P2 2.3038(12); Pd2–N2 2.226(4); Pd2–P3 2.3202(12); Pd2–P4 2.3303(13); N1–Pd1–Pd2 173.84(10); P2–Pd1–P1 175.26(4); N2–Pd2–Pd1 156.63(11); P3–Pd2–P4 167.41(5).

Table S8. Crystal data and structure refinement for the second conformer of **4^{iPr}-Pd**.

Empirical formula	$C_{68}H_{108}N_2P_4Pd_2$
Formula weight	1290.24
Temperature [K]	120(1)
Crystal system	triclinic
Space group (number)	$P\overline{1}$ (2)
a [Å]	14.5017(4)
b [Å]	15.8648(4)
c [Å]	17.0531(4)
α [°]	111.918(2)
β [°]	99.264(2)
γ [°]	108.222(2)
Volume [Å^3]	3281.06(16)
Z	2
ρ_{calc} [gcm^{-3}]	1.306
μ [mm^{-1}]	5.635
transmission factors (max, min)	1.000, 0.830
F(000)	1364
Radiation	Cu K_α (λ=1.54184 Å)
2θ range [°]	5.89 to 134.15 (0.84 Å)
Index ranges	−17 ≤ h ≤ 17, −18 ≤ k ≤ 18, −20 ≤ l ≤ 20
Reflections collected	58683
Independent reflections	11713 (R_{int} = 0.1108)
observed [I≥2σ(I)]	8740
Completeness to θ = 67.684°	99.9 %
Data / Restraints / Parameters	11713 / 0 / 713
Goodness-of-fit on F^2	1.000
Final *R* indexes [I≥2σ(I)]	R_1 = 0.0488 wR_2 = 0.1076
Final *R* indexes [all data]	R_1 = 0.0753 wR_2 = 0.1209
Largest peak/hole [eÅ^{-3}]	0.96/−0.95
CCDC number	2470082

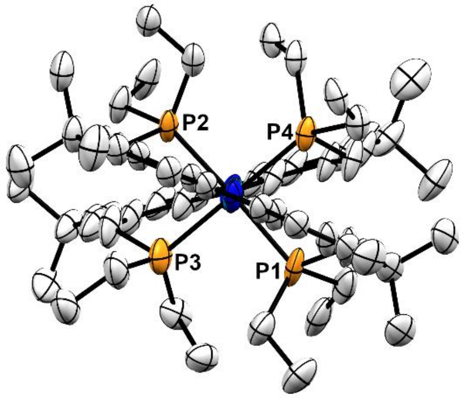

Figure S147. Molecular crystal structure of **4^{Et}-Pd** (view along the Pd1–Pd2 axis; for side view see main article Figure 4b). Ellipsoids shown with 50% probability. Hydrogen atoms and disorder in one ^{t}Bu- and three Et-groups are omitted for clarity.

Table S9. Crystal data and structure refinement for **4^{Et}-Pd**.

Empirical formula	$C_{67}H_{100}N_2P_4Pd_2$
Formula weight	1270.16
Temperature [K]	120(1)
Crystal system	triclinic
Space group (number)	$P\bar{1}$ (2)
a [Å]	12.1035(4)
b [Å]	15.3702(7)
c [Å]	18.8872(7)
α [°]	76.688(4)
β [°]	73.438(3)
γ [°]	73.904(3)
Volume [Å^3]	3191.9(2)
Z	2
ρ_{calc} [gcm^{-3}]	1.322
μ [mm^{-1}]	5.787
transmission factors (max, min)	1.000, 0.607
F(000)	1336
Radiation	Cu K_α (λ=1.54184 Å)
2θ range [°]	6.07 to 134.15 (0.84 Å)
Index ranges	$-14 \le h \le 14$, $-18 \le k \le 18$, $-22 \le l \le 22$
Reflections collected	68087
Independent reflections	11209 (R_{int} = 0.1110)
observed [$I \ge 2\sigma(I)$]	8188
Completeness to θ = 67.077°	98.2 %
Data / Restraints / Parameters	11209 / 535 / 733
Goodness-of-fit on F^2	1.055
Final *R* indexes [$I \ge 2\sigma(I)$]	R_1 = 0.0635 wR_2 = 0.1591
Final *R* indexes [all data]	R_1 = 0.0866 wR_2 = 0.1787
Largest peak/hole [eÅ^{-3}]	1.63/−1.77
CCDC number	2470083

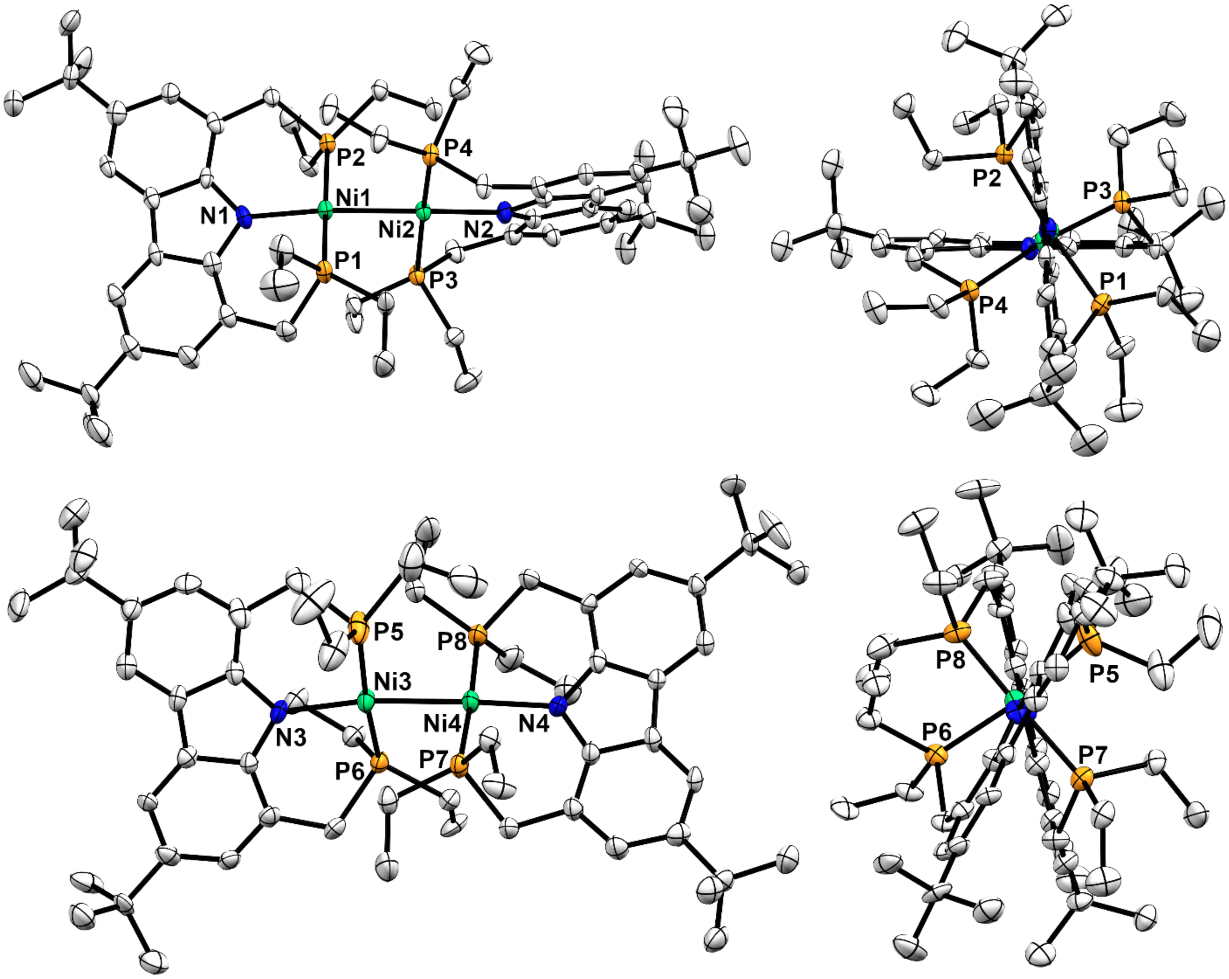

Figure S148. Molecular crystal structures of **4^Et-Ni** (top left: first conformer side view; top right: first conformer view along the Ni1–N2 axis; bottom left: second conformer side view; bottom right: second conformer view along the Ni3–Ni4 axis), showing the two crystallographically independent molecules in the cell (conformers). Ellipsoids shown with 50% probability. Hydrogen atoms and co-crystallized Et_2O molecules as well as disorder of two ^{t}Bu groups in the second conformer are omitted for clarity. Selected bond parameters in [Å] and [°]: Ni1–Ni2 2.3827(14); Ni3–Ni4 2.3703(14); Ni1–N1 2.000(5); Ni2–N2 1.997(5); Ni3–N3 1.987(5); Ni4–N4 1.983(5); Ni1–P1 2.180(2); Ni1–P2 2.194(2); Ni2–P3 2.1866(18); Ni2–P4 2.1882(18); Ni3–P5 2.194(2); Ni3–P6 2.170(2); Ni4–P7 2.181(2); Ni4–P8 2.178(2); N1–Ni1–Ni2 172.90(16); N2–Ni2–Ni1 174.45(16); N3–Ni3–Ni4 171.62(17); N4–Ni4–Ni3 176.78(16); P1–Ni1–P2 175.57(8); P3–Ni2–P4 172.93(9); P5–Ni3–P6 171.20(9); P7–Ni4–P8 176.92(9).

Table S10. Crystal data and structure refinement for **4^Et-Ni**.

Empirical formula	$C_{130}H_{209}N_4Ni_4O_{2.50}P_8$
Formula weight	2350.60
Temperature [K]	120(1)
Crystal system	triclinic
Space group (number)	$P\bar{1}$ (2)
a [Å]	16.1407(6)
b [Å]	17.7970(7)
c [Å]	24.8898(9)
α [°]	73.664(4)
β [°]	86.054(3)
γ [°]	70.418(4)
Volume [Å³]	6461.7(5)
Z	2
ρ_{calc} [gcm⁻³]	1.208
μ [mm⁻¹]	1.961
transmission factors (max, min)	1.000, 0.630
F(000)	2538

Radiation	Cu $K_α$ (λ=1.54184 Å)
2θ range [°]	5.48 to 134.65 (0.84 Å)
Index ranges	−19 ≤ h ≤ 19, −21 ≤ k ≤ 21, −29 ≤ l ≤ 29
Reflections collected	41419
Independent reflections	41419 (R_{int} = 0.124)<br>footnote: R_{int} from HKLF5 statistics (CrysAlisPro)
observed [I≥2σ(I)]	23038
Completeness to θ = 67.326°	98.9 %
Data / Restraints / Parameters	41419 / 47 / 1312
Goodness-of-fit on F^2	0.901
Final R indexes [I≥2σ(I)]	R_1 = 0.0787<br>wR_2 = 0.1935
Final R indexes [all data]	R_1 = 0.1258<br>wR_2 = 0.2118
Largest peak/hole [eÅ^{-3}]	1.08/−0.93
CCDC number	2470084

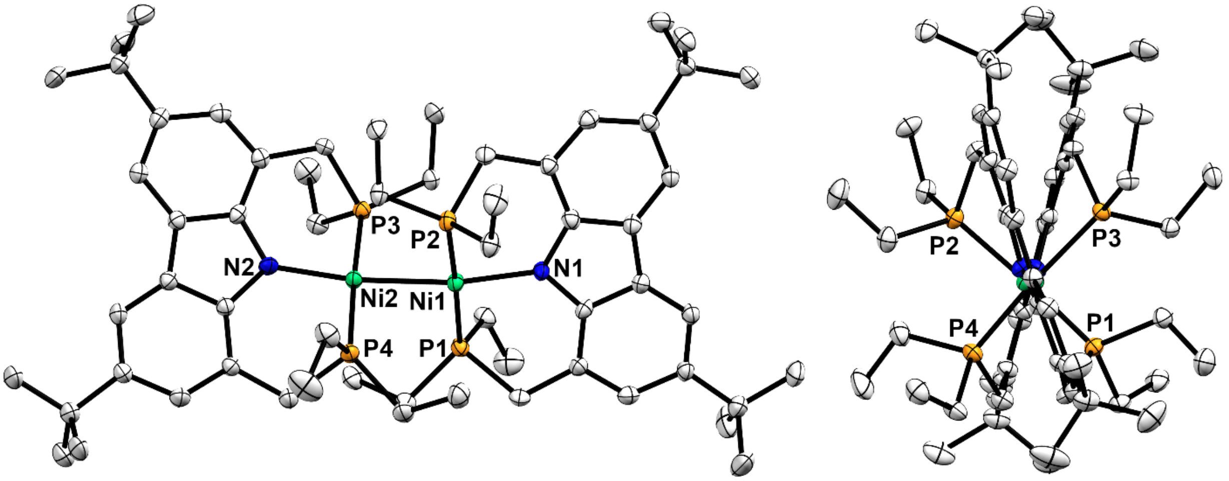

Figure S149. Molecular crystal structure of a third conformer for **4^{Et}-Ni** (left: side view; right: view along the Ni1–N2 axis). Ellipsoids shown with 50% probability. Hydrogen atoms and co-crystallized toluene are omitted for clarity. Selected bond parameters in [Å] and [°]: Ni1–Ni2 2.3228(4); Ni1–N1 1.973(2); Ni2–N2 1.974(2); Ni1–P1 2.1821(8); Ni1–P2 2.1865(8); Ni2–P3 2.1955(8); Ni2–P4 2.1831(8); N1–Ni1–Ni2 170.65(6); N2–Ni2–Ni1 171.47(6); P1–Ni1–P2 176.39(3); P3–Ni2–P4 175.23(3).

Table S11. Crystal data and structure refinement for the third conformer for **4^{Et}-Ni**.

Empirical formula	$C_{81}H_{116}N_2Ni_2P_4$
Formula weight	1359.05
Temperature [K]	120(1)
Crystal system	monoclinic
Space group (number)	$P2_1/c$ (14)
a [Å]	15.5728(4)
b [Å]	25.7704(5)
c [Å]	18.9835(4)
α [°]	90
β [°]	99.490(2)
γ [°]	90
Volume [Å^3]	7514.1(3)
Z	4
ρ_{calc} [gcm^{-3}]	1.201
μ [mm^{-1}]	0.629
transmission factors (max, min)	1.000, 0.869
F(000)	2928
Radiation	Mo K_α (λ=0.71073 Å)
2θ range [°]	4.97 to 52.74 (0.80 Å)
Index ranges	−19 ≤ h ≤ 19, −32 ≤ k ≤ 32, −23 ≤ l ≤ 23
Reflections collected	142414
Independent reflections	15353 (R_{int} = 0.1432)
observed [I≥2σ(I)]	10233
Completeness to θ = 25.242°	99.9 %
Data / Restraints / Parameters	15353 / 0 / 697
Goodness-of-fit on F^2	1.001
Final *R* indexes [I≥2σ(I)]	R_1 = 0.0488 wR_2 = 0.0992
Final *R* indexes [all data]	R_1 = 0.0813 wR_2 = 0.1100
Largest peak/hole [eÅ^{-3}]	0.45/−0.34
CCDC number	2470085

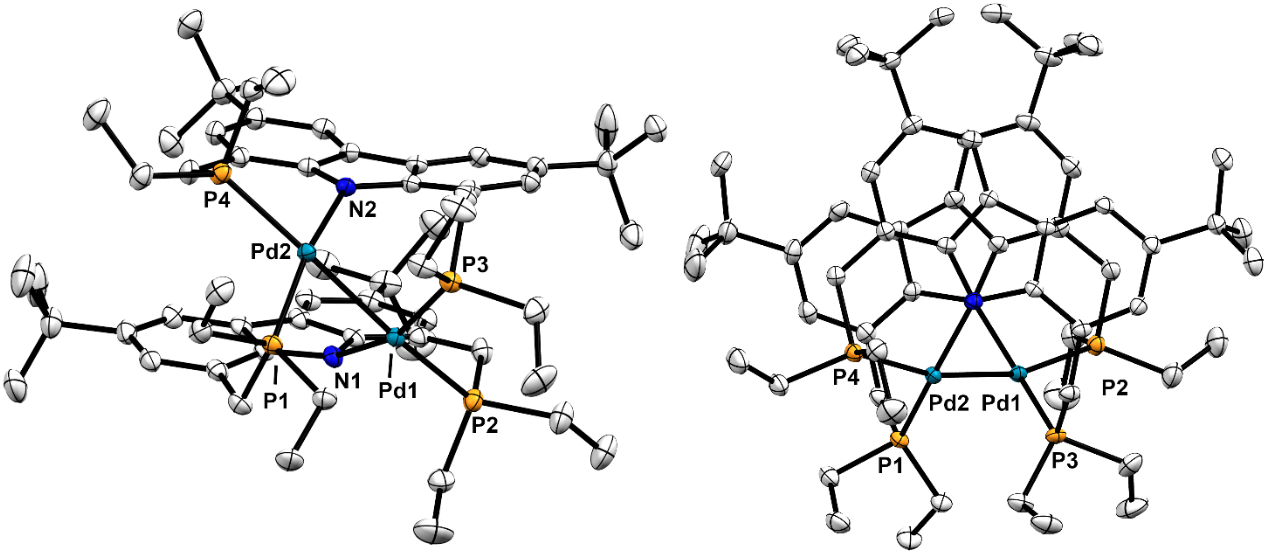

Figure S150. Molecular crystal structure of a bridged constitution isomer of **4^{Et}-Pd** (left: side view; right: view along the N2–N1 axis). Ellipsoids shown with 50% probability. Hydrogen atoms and one disordered ^{t}Bu-group are omitted for clarity.

Table S12. Crystal data and structure refinement for the bridged constitution isomer of **4^{Et}-Pd**.

Empirical formula	$C_{63}H_{95}N_2P_4Pd_2$
Formula weight	1217.08
Temperature [K]	120(1)
Crystal system	monoclinic
Space group (number)	$P2_1/c$ (14)
a [Å]	12.16235(4)
b [Å]	25.81107(7)
c [Å]	19.59073(7)
α [°]	90
β [°]	96.9393(3)
γ [°]	90
Volume [Å^3]	6104.94(3)
Z	4
ρ_{calc} [gcm^{-3}]	1.324
μ [mm^{-1}]	6.027
transmission factors (max, min)	1.000, 0.644
F(000)	2556
Radiation	Cu K_α (λ=1.54184 Å)
2θ range [°]	5.69 to 141.79 (0.82 Å)
Index ranges	−14 ≤ h ≤ 14, −31 ≤ k ≤ 31, −22 ≤ l ≤ 23
Reflections collected	180212
Independent reflections	11709 (R_{int} = 0.0457)
observed [I≥2σ(I)]	11515
Completeness to θ = 67.684°	99.9 %
Data / Restraints / Parameters	11709 / 39 / 664
Goodness-of-fit on F^2	1.036
Final *R* indexes [I≥2σ(I)]	R_1 = 0.0244 wR_2 = 0.0658
Final *R* indexes [all data]	R_1 = 0.0248 wR_2 = 0.0661
Largest peak/hole [eÅ^{-3}]	0.42/−0.97
CCDC number	2470086

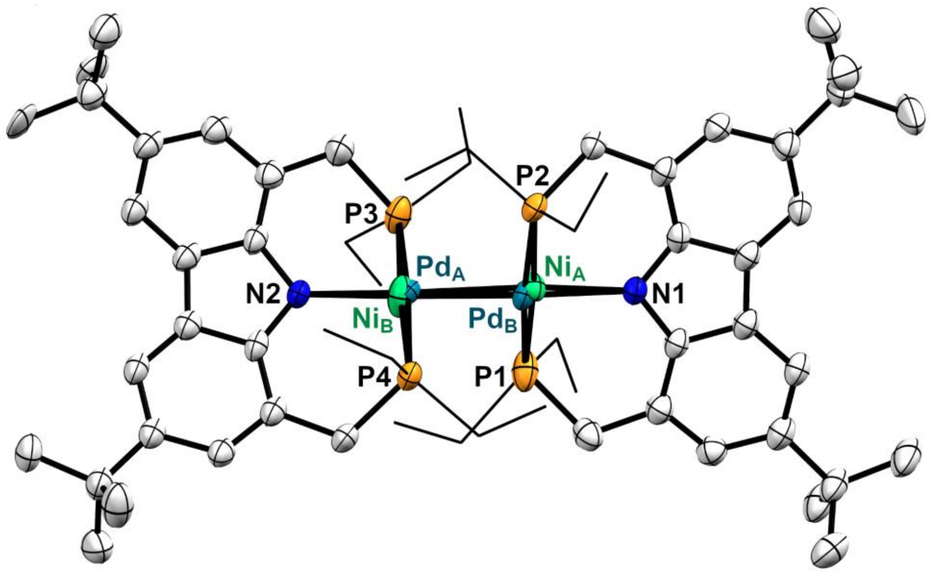

Figure S151. Molecular crystal structure of **7^{Et}-NiPd** showing disordered Ni/Pd metal centres. Ellipsoids shown with 50% probability. Hydrogen atoms, one disordered ^{t}Bu-group and three disordered Et-groups are omitted for clarity. Selected bond parameters in [Å]: NiA–PdA 2.486(8); NiB–PdB 2.442(8).

Table S13. Crystal data and structure refinement for **7^{Et}-NiPd**.

Empirical formula	$C_{66}H_{98}N_2NiP_4Pd$
Formula weight	1208.45
Temperature [K]	120(1)
Crystal system	triclinic
Space group (number)	$P\bar{1}$ (2)
a [Å]	12.1970(5)
b [Å]	15.2108(4)
c [Å]	18.7052(5)
α [°]	78.192(2)
β [°]	74.371(3)
γ [°]	74.429(3)
Volume [Å^3]	3186.42(19)
Z	2
ρ_{calc} [gcm^{-3}]	1.260
μ [mm^{-1}]	3.880
transmission factors (max, min)	1.000, 0.603
F(000)	1284
Radiation	Cu K_α (λ=1.54184 Å)
2θ range [°]	6.09 to 142.68 (0.81 Å)
Index ranges	−14 ≤ h ≤ 14, −18 ≤ k ≤ 18, −22 ≤ l ≤ 22
Reflections collected	87595
Independent reflections	12105 (R_{int} = 0.1002)
observed [I≥2σ(I)]	8860
Completeness to θ = 67.684°	99.8 %
Data / Restraints / Parameters	12105 / 80 / 740
Goodness-of-fit on F^2	1.028
Final *R* indexes [I≥2σ(I)]	R_1 = 0.0560 wR_2 = 0.1286
Final *R* indexes [all data]	R_1 = 0.0808 wR_2 = 0.1434
Largest peak/hole [eÅ^{-3}]	0.79/−0.95
CCDC number	2470087

6) IR spectroscopy

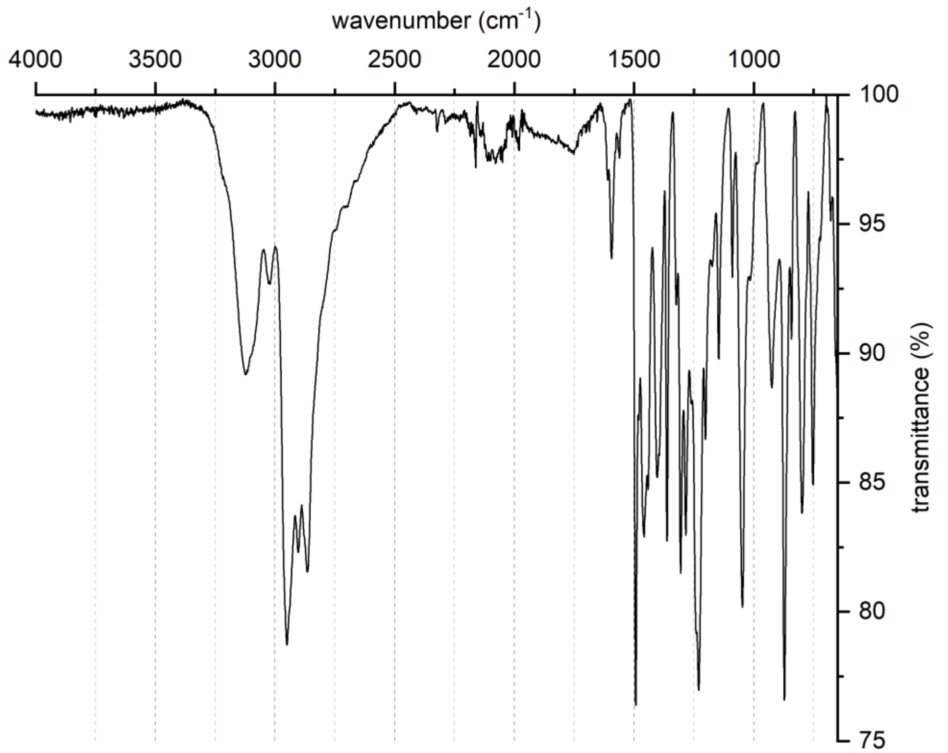

Figure S152. IR (ATR) spectrum of **[(^{Et}PNP)H_3]Br_2** at 298 K.

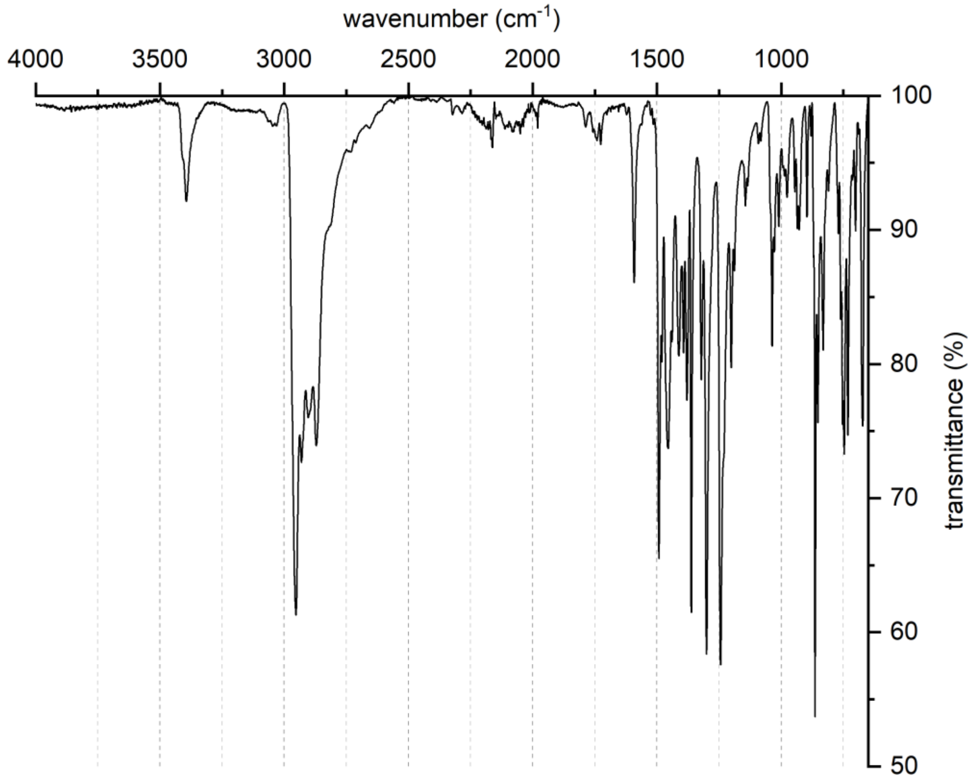

Figure S153. IR (ATR) spectrum of **(^{Et}PNP)H (1^{Et})** at 298 K.

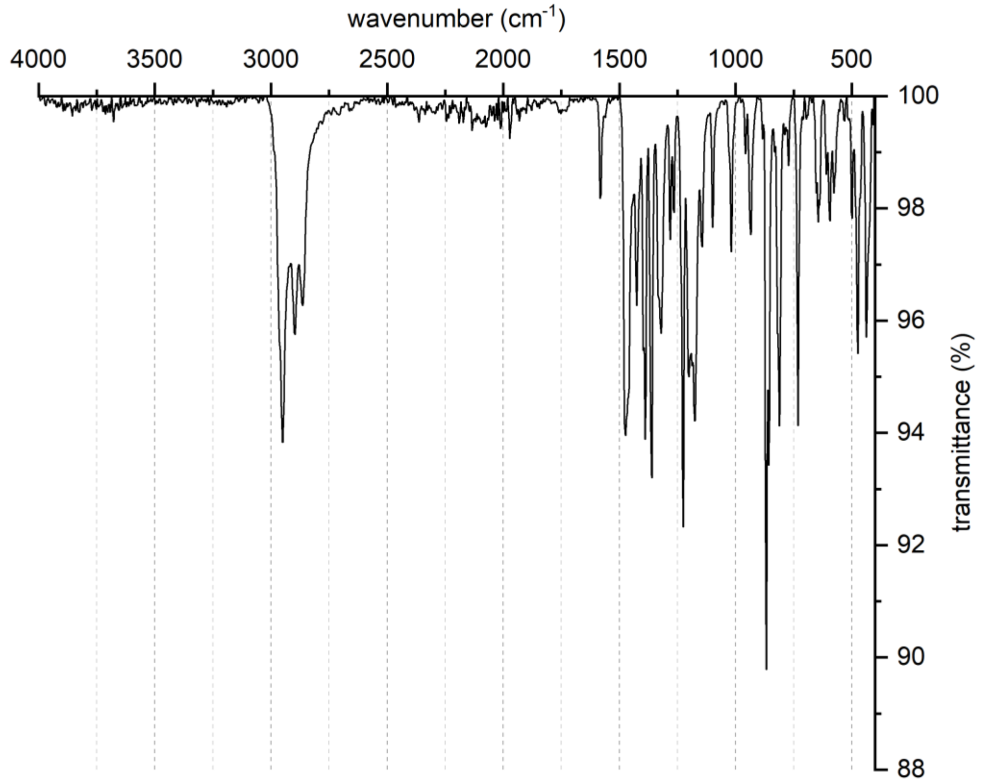

Figure S154. IR (ATR) spectrum of **2^{iPr}-Pd** at 298 K.

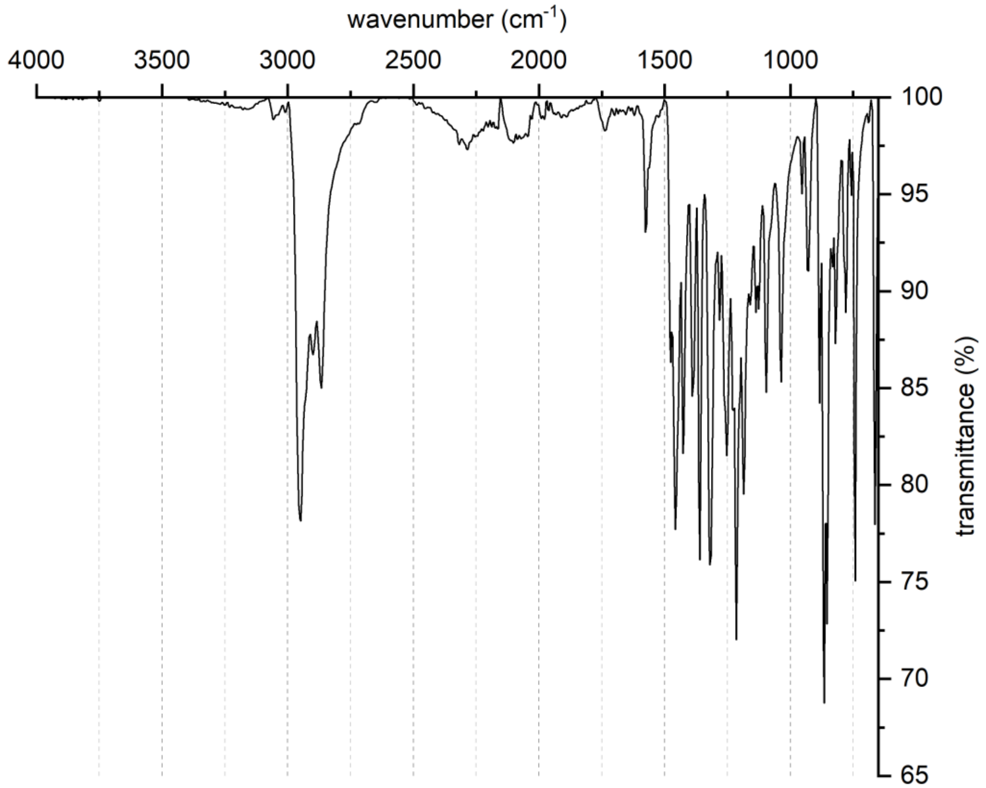

Figure S155. IR (ATR) spectrum of **2^{iPr}-Ni** at 298 K.

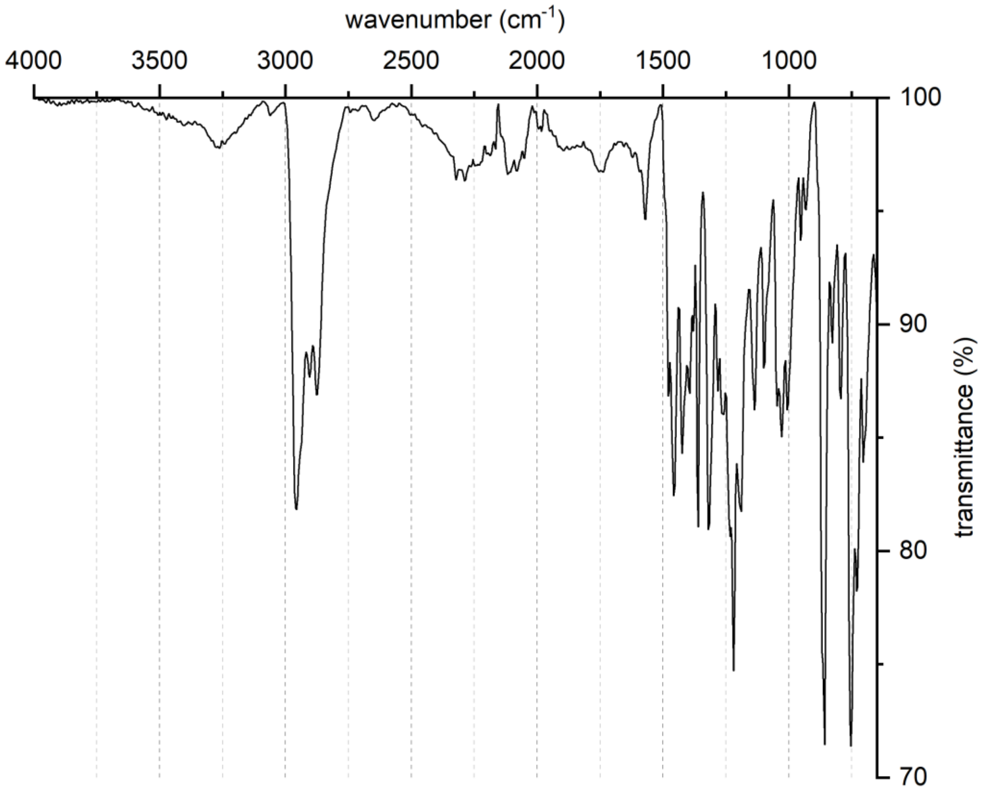

Figure S156. IR (ATR) spectrum of **2^{Et}-Pd** at 298 K.

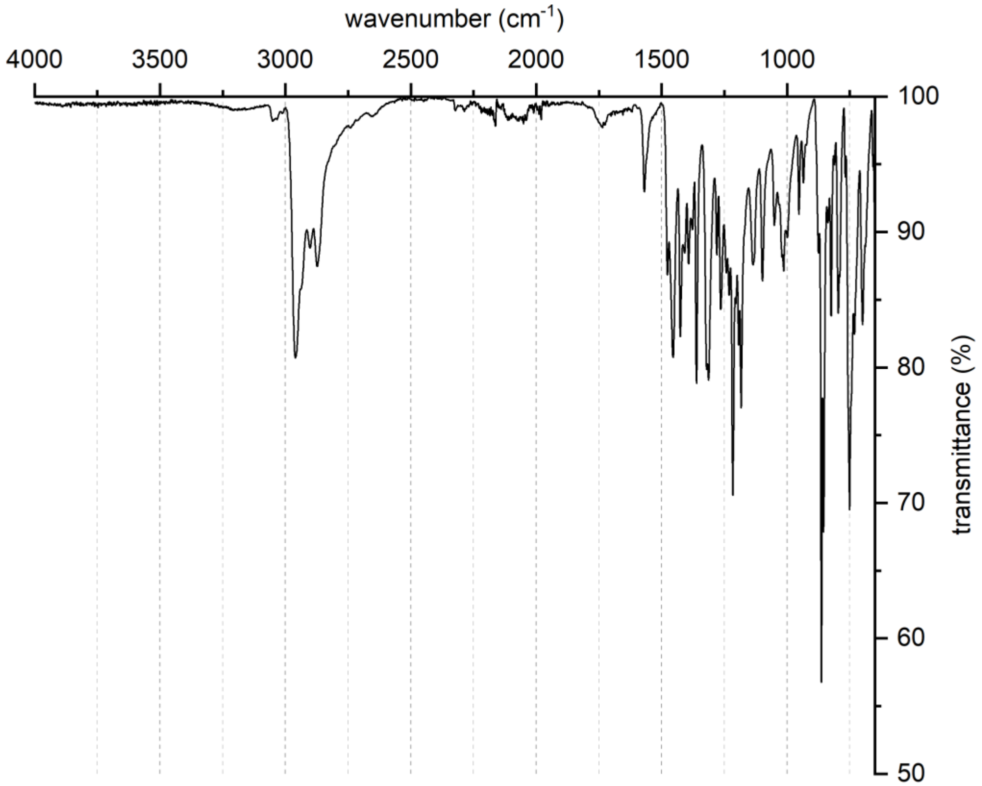

Figure S157. IR (ATR) spectrum of **2^{Et}-Ni** at 298 K.

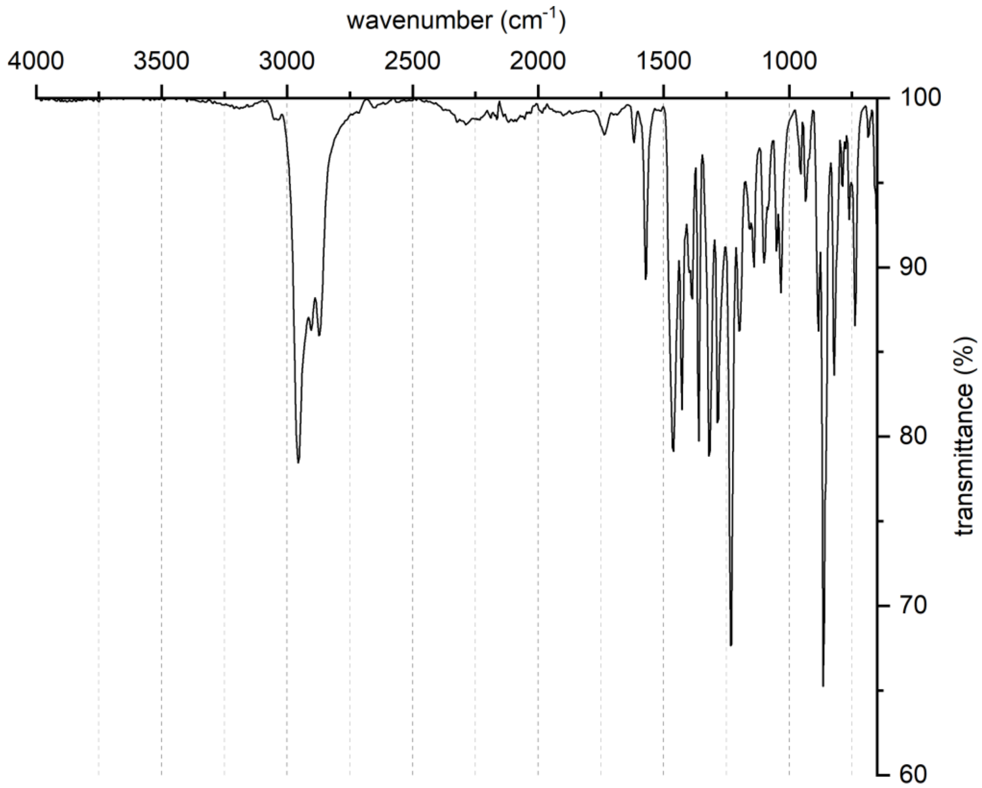

Figure S158. IR (ATR) spectrum of **4^{iPr}-Pd** at 298 K.

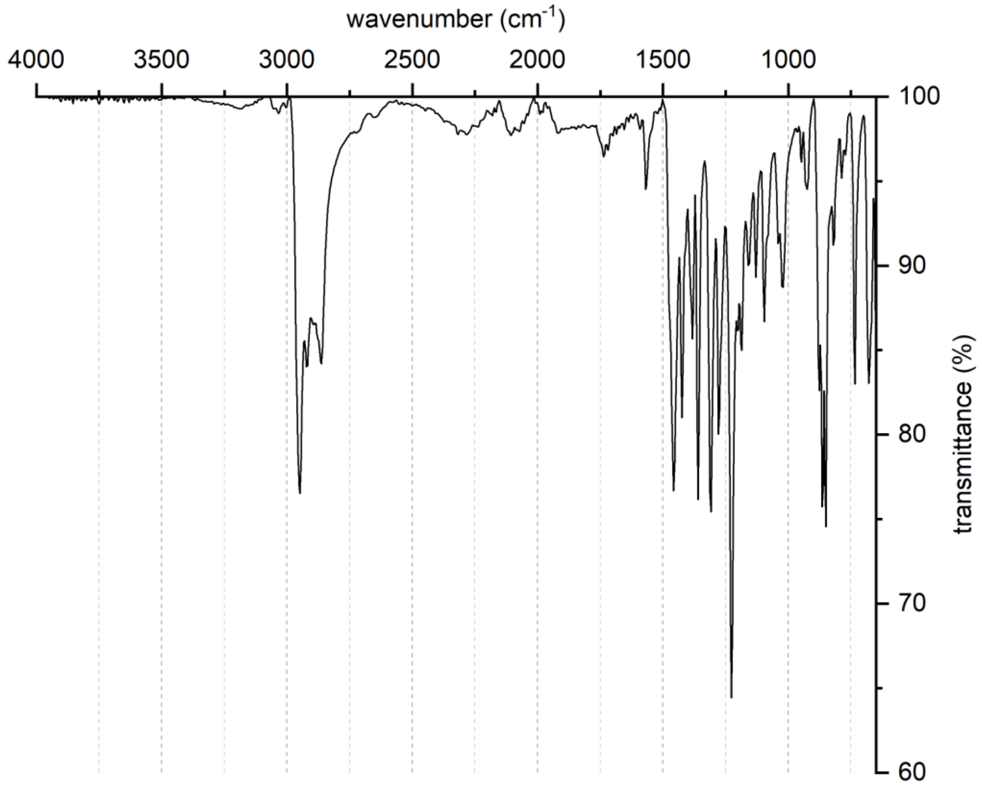

Figure S159. IR (ATR) spectrum of **3^{iPr}-Ni** at 298 K.

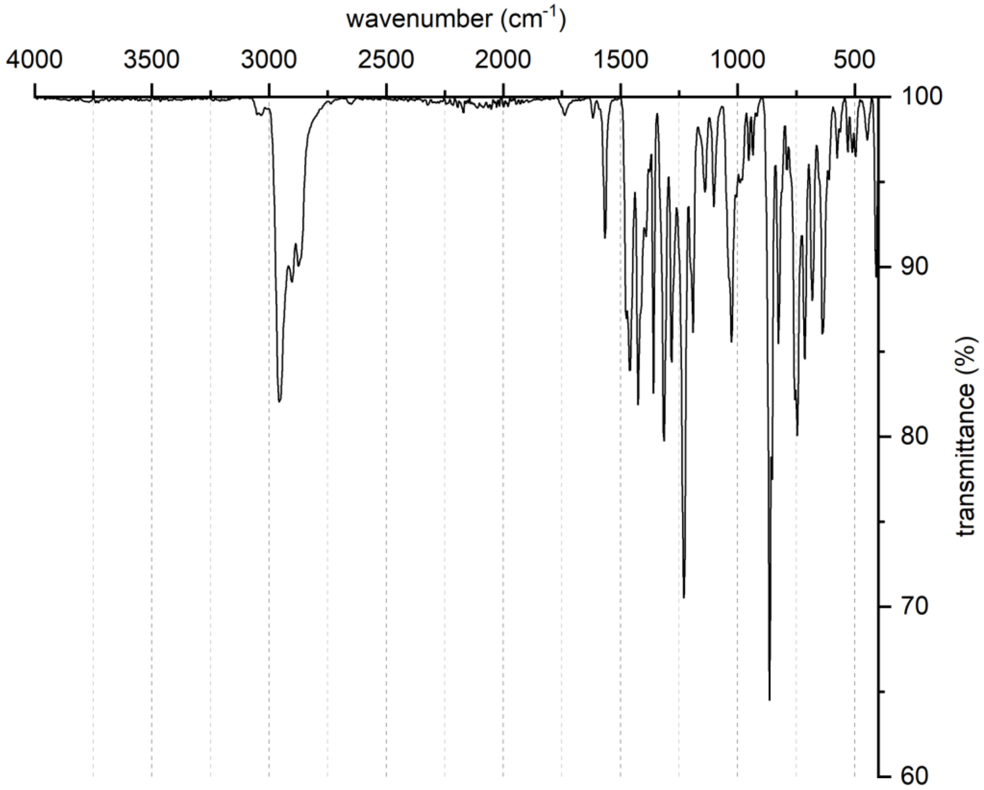

Figure S160. IR (ATR) spectrum of **4^{Et}-Pd** at 298 K.

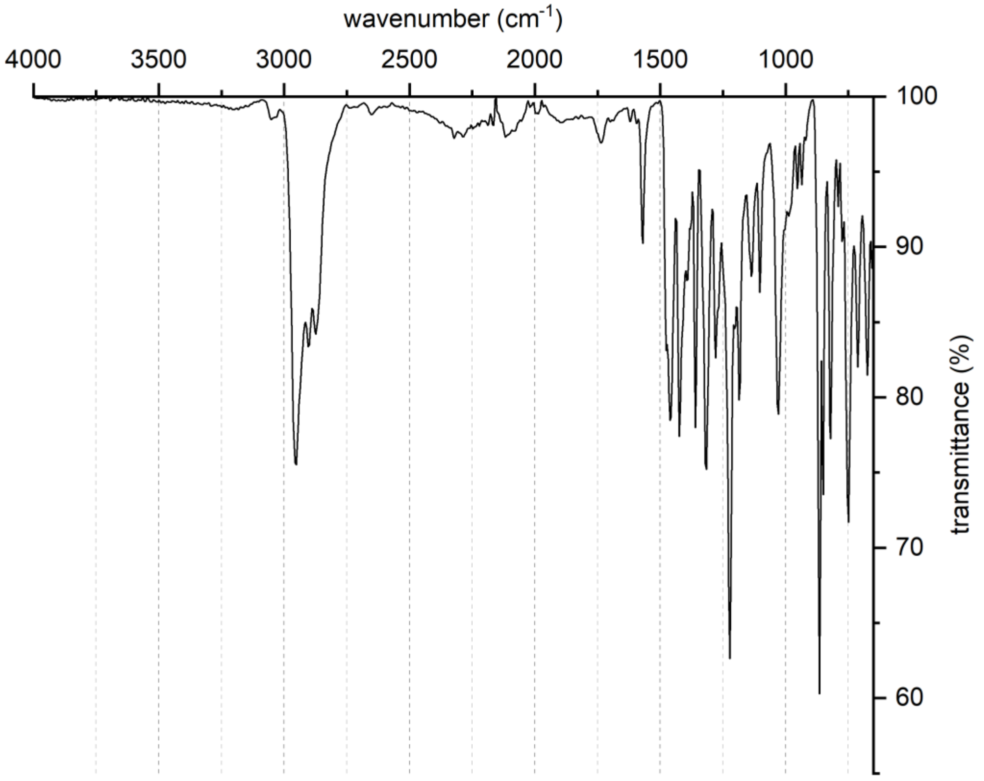

Figure S161. IR (ATR) spectrum of **4^{Et}-Ni** at 298 K.

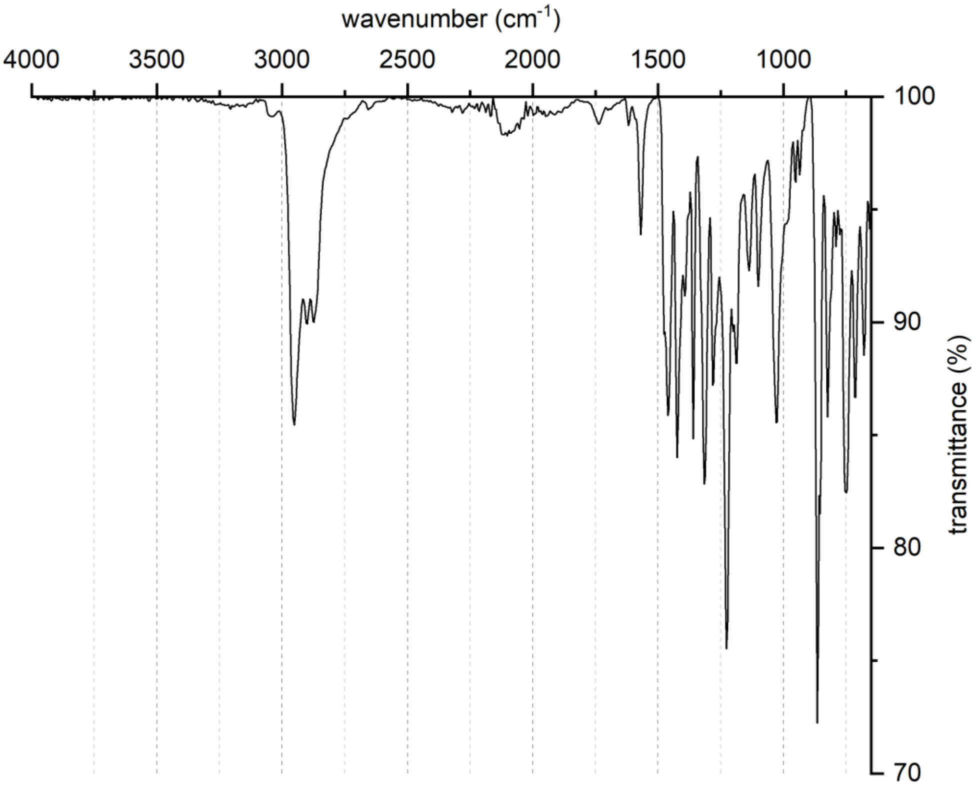

Figure S162. IR (ATR) spectrum of **7^{Et}-NiPd** at 298 K.

7) Kinetic and Thermodynamic Measurements

8.1) First Isomerization Barrier of 4^{iPr}-Pd

Variable temperature ^{1}H NMR measurements (Figure S163) were performed to quantify the energy barrier associated with the isomerization arising from conformational flexibility of the methylene bridge (-CH_2-) linking the carbazole core with a phosphine group in complex **4^{iPr}-Pd**. Merging of the diastereotopic (at low temperatures) -CH_2- proton resonances occurred at the coalescence temperature T_c = 276.15 K (Figure S163 d). Deconvolution of these coalescing signals (Figure S164) afforded a precise chemical shift difference of Δv = 120.702 Hz. The exchange rate constant (k_c) at the coalescence temperature was calculated according to:

$$k_c = \frac{\pi \Delta v}{\sqrt{2}} = 268.133\ Hz$$

The Gibbs free energy of activation ($\Delta G^{\ddagger}$) at T_c was determined using the Eyring-Polanyi equation, assuming a transmission coefficient κ = 1:

$$k_c = \frac{\kappa k_B T_c}{h} e^{-\Delta G^{\ddagger}/RT_c}$$

Solving for $\Delta G^{\ddagger}$ gives:

$$\Delta G^{\ddagger}_{276.15\ \mathrm{K}} = -RT_c \ln\left(\frac{k_c h}{\kappa k_B T_c}\right) = 13.1\ \mathrm{kcal\ mol^{-1}}$$

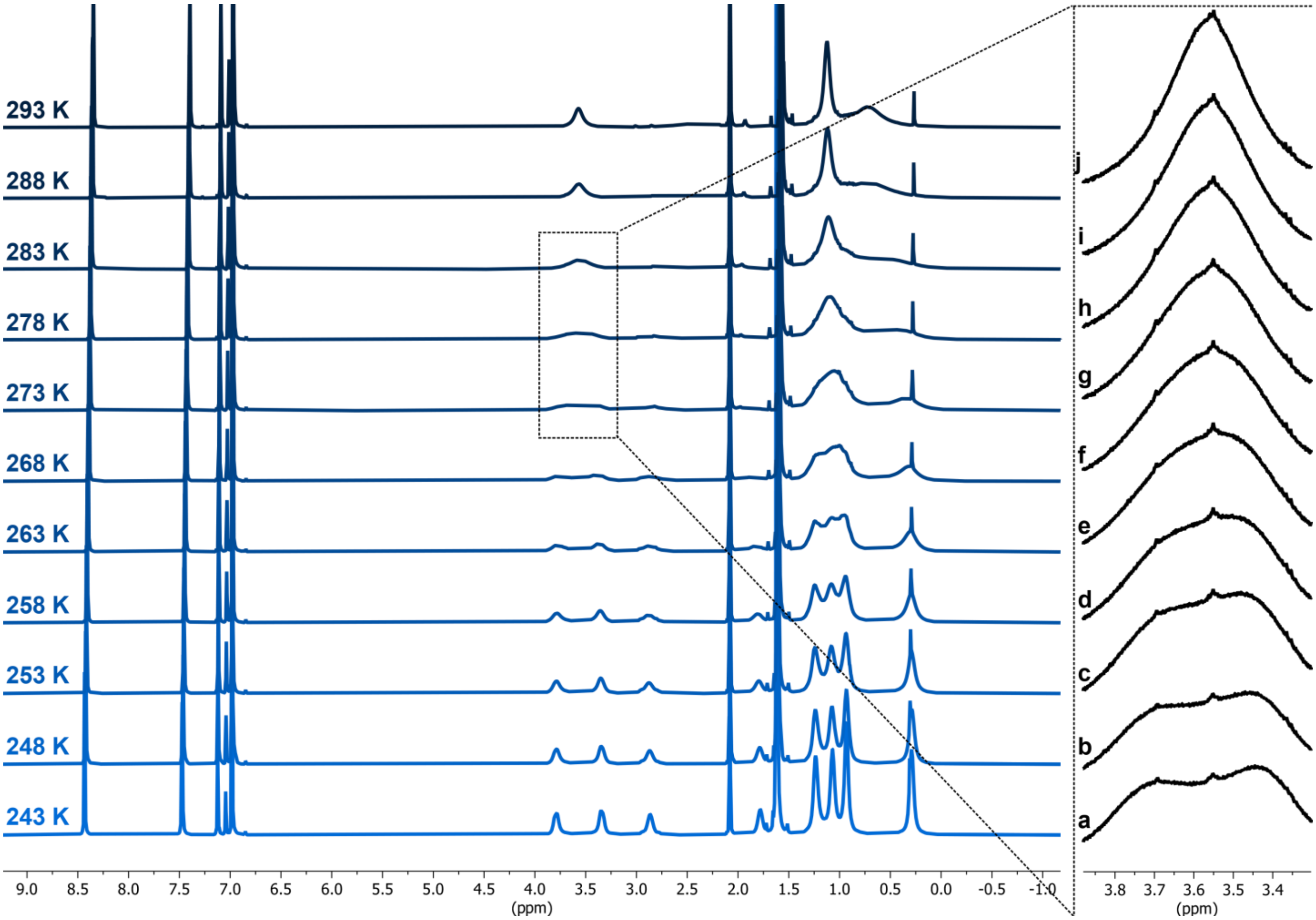

Figure S163. ^{1}H NMR spectra (tol-d_8, 600 MHz) of **[(^{iPr}PNP)Pd]$_2$ (4^{iPr}-Pd)** at low temperatures with an interval of 5 K. For a more precise determination of the coalescence temperature, ten spectra in 1 K intervals (a-j) were recorded between 273.15 K (a) and 282.15 K (j).

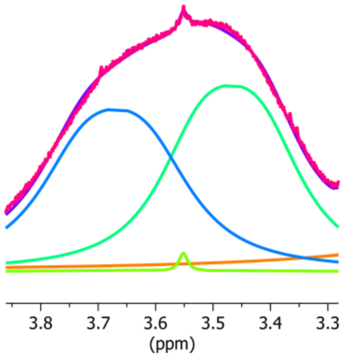

Figure S164. Deconvoluted resonances of the -CH_2- protons at 276.15 K of **[(^{iPr}PNP)Pd]$_2$ (4^{iPr}-Pd)**.

8.2) Thermodynamics of a Low-Energy Conformer of 4^{iPr}-Pd

At temperatures significantly below T_c, an additional resonance emerged in the ^{31}P NMR spectrum (Figure S82). As conformational isomerization is very slow at these low temperatures ("frozen ligand"), this signal was attributed to a second conformation that is slightly higher in energy than the major conformation. Density functional theory (DFT) computations corroborated the existence of such a low-energy conformer (see Computational Section). Integration of the ^{31}P signals at 243 K allowed the calculation of an equilibrium constant (K_{eq}) for the conformational interconversion:

$$K_{eq} = \frac{[major\ conformer]}{[minor\ conformer]} = \frac{0.889}{0.111} = 8.039$$

The relative Gibbs free energy difference between the two conformers at this temperature is therefore:

$$\Delta G_{conf}(243\ \text{K}) = -RT\ln(K_{eq}) = -4.206\ \text{kJ mol}^{-1} = -1.005\ \text{kcal mol}^{-1}$$

Integration ratios determined *via* spectral deconvolution at 243.15, 248.15 and 253.15 K (Table S14) enabled a van 't Hoff analysis:

$$\ln(K_{eq}) = -\frac{\Delta H°}{R}\left(\frac{1}{T}\right) + \frac{\Delta S°}{R}$$

Linear regression of $\ln(K_{eq})$ against 1/*T* provided the relative enthalpy ($\Delta H°$) and entropy ($\Delta S°$) (Figure S165). The relative Gibbs free energy ($\Delta G°_{298\ \text{K}}$) was then calculated as:

$$\Delta G°_{298\ \text{K}} = \Delta H° - T\Delta S° = (-1.39 \pm 0.14)\ \text{kcal mol}^{-1}$$

Table S14. Temperature-dependent change of ^{31}P signal integration ratios and therefore the equilibrium between two low-energy conformers of **4^{iPr}-Pd**.

T (K)	1/T (1/K)	Integral 1	Integral 2	K_{eq}	$\ln(K_{eq})$
243.15	0.00411269	0.8894	0.1106	8.039	2.084
248.15	0.00402982	0.8928	0.1072	8.325	2.119
253.15	0.00395024	0.8948	0.1052	8.506	2.141

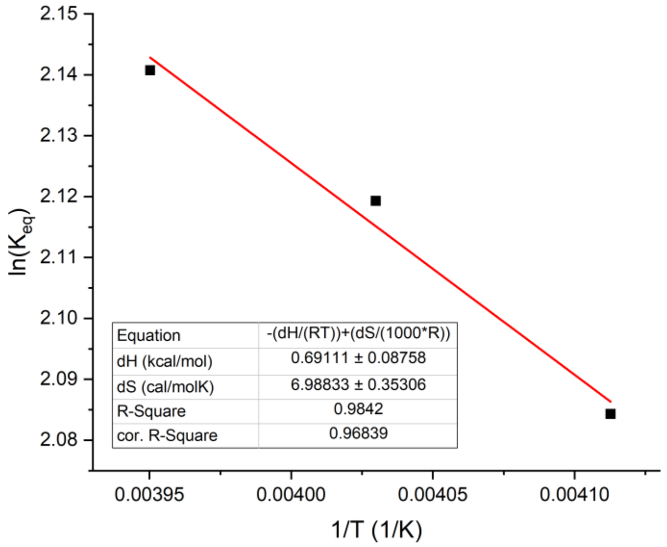

Figure S165. Van ’t Hoff Plot for the equilibrium between low-energy conformers of **4^{iPr}-Pd**.

8.3) Thermodynamics of the Reversible Pd–Pd Bond Dissociation in [(^{iPr}PNP)Pd]$_2$

The reversible Pd–Pd bond dissociation in **4^{iPr}-Pd** forming the monomer (**3^{iPr}-Pd**) was investigated using ^{1}H NMR spectroscopy (Figure S166/S167). The concentration ratios at different temperatures (Table S15) were determined by integrating exchange coupled proton signals at δ = 9.3 ppm (monomer, **3^{iPr}-Pd**) and δ = 7.5 ppm (dimer, **4^{iPr}-Pd**), referenced to a solution concentration of [**4^{iPr}-Pd**]$_0$ = 0.0183 mol L^{-1} (concentration before dissociation). The equilibrium constant is defined as

$$K_{eq} = \frac{[monomer]^2}{[dimer]}$$

A van ‘t Hoff analysis (Figure S168) of the temperature-dependent equilibrium constants (Table S16) provided the bond dissociation enthalpy (BDE, $\Delta H°$), entropy ($\Delta S°$), and bond dissociation free energy (BDFE, $\Delta G°$):

$$\Delta H° = (24.064 \pm 0.424)\ \mathrm{kcal\ mol^{-1}}$$

$$\Delta S° = (62.203 \pm 1.321)\ \mathrm{cal\ mol^{-1} K^{-1}}$$

$$\Delta G°_{298\ \mathrm{K}} = \Delta H° - T\Delta S° = (5.527 \pm 0.579)\ \mathrm{kcal\ mol^{-1}}$$

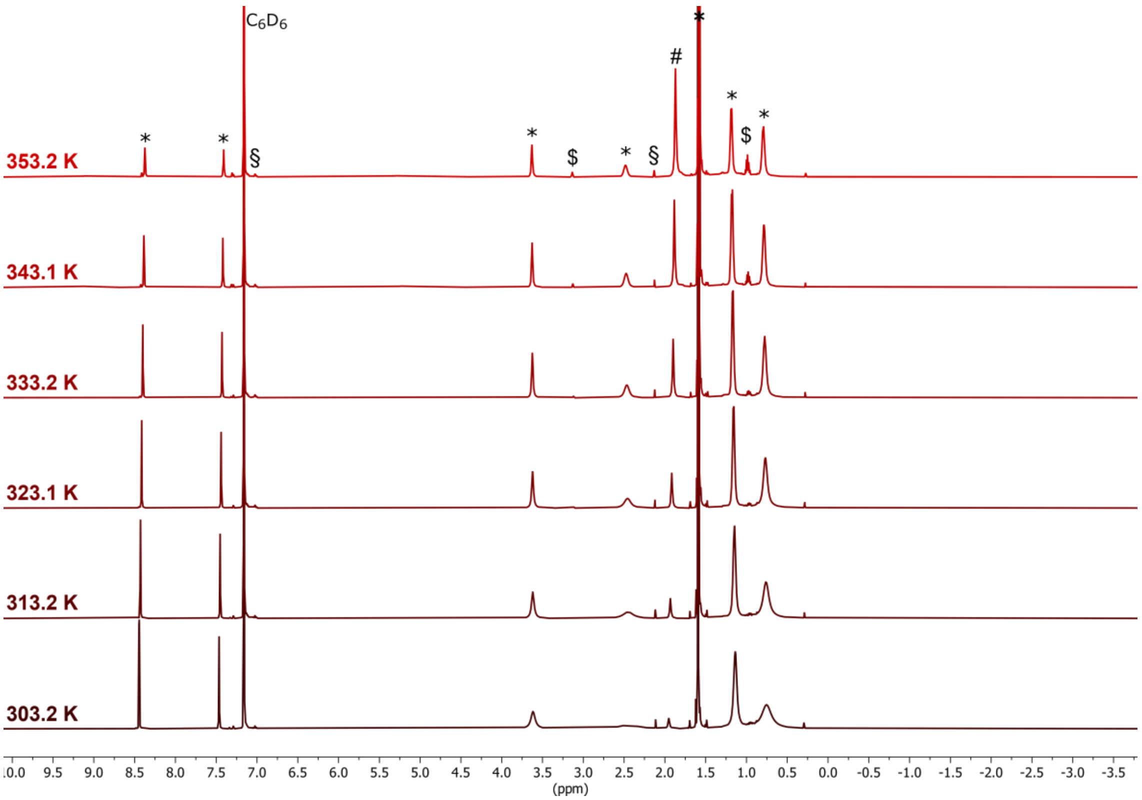

Figure S166. ^{1}H NMR spectra (C_6D_6, 600 MHz) of **[(^{iPr}PNP)Pd]$_2$ (4^{iPr}-Pd)** at variable temperatures with an interval of 10 K. Signal assignments were made in the spectrum at 353.15 K: * = **4^{iPr}-Pd**, $ = **[(^{iPr}PNP)PdH]**, § = toluene, # = ^{t}Bu-group of the monomer **3^{iPr}-Pd**.

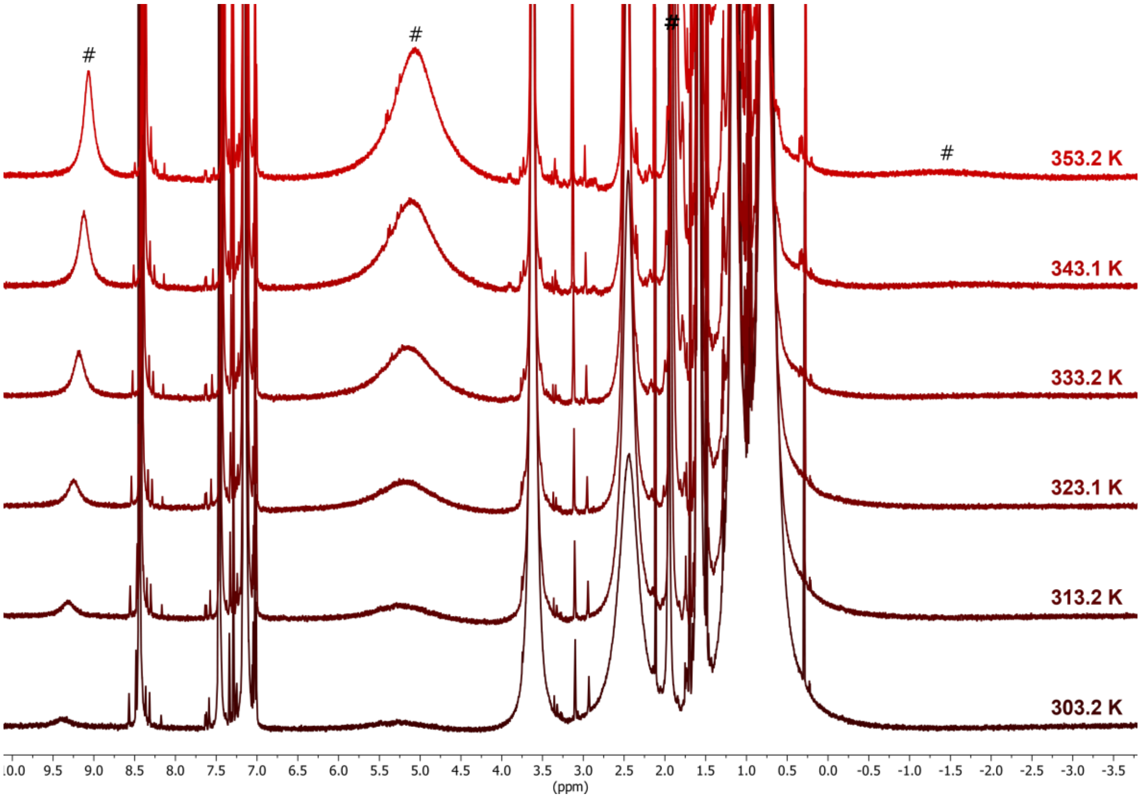

Figure S167. Intensified ^{1}H NMR spectra (C_6D_6, 600 MHz) of **[(^{iPr}PNP)Pd]$_2$ (4^{iPr}-Pd)** at variable temperatures with an interval of 10 K. Observable paramagnetic proton resonances of the monomer **3^{iPr}-Pd** are labeled with #.

Table S15. Temperature-dependent change of ^{1}H signal integration ratios and therefore concentrations between **4^{iPr}-Pd** and **3^{iPr}-Pd** ([**4^{iPr}-Pd**]$_0$ = 0.0183 mol L^{-1}).

T (K)	NMR integration of Dimer	NMR integration of Monomer	Dimer [%]	Monomer [%]	[Dimer] (mmol/L)	[monomer] (mmol/L)
293.2	1	0.0119	97.68	2.32	17.828	0.849
303.2	1	0.0249	95.26	4.74	17.386	1.732
313.2	1	0.0529	90.43	9.57	16.506	3.493
323.1	1	0.0967	83.79	16.21	15.294	5.916
333.2	1	0.1769	73.87	26.13	13.482	9.540
343.1	1	0.3082	61.87	38.13	11.292	13.920
353.2	1	0.5942	45.70	54.30	8.340	19.823

Table S16. Temperature-dependent values of the equilibrium between **4^{iPr}-Pd** and **3^{iPr}-Pd** used in the van 't Hoff plot, including the corresponding relative Gibbs free energies.

1/T (1/K)	K_{eq}	$\ln(K_{eq})$	ΔG (kcal/mol)
0.0034	4.039e-5	-10.117	5.89
0.0033	1.725e-4	-8.665	5.22
0.0032	7.390e-4	-7.210	4.49
0.0031	2.288e-3	-6.080	3.90
0.0030	6.750e-2	-4.998	3.31
0.0029	1.716e-2	-4.065	2.77
0.0028	4.712e-2	-3.055	2.14

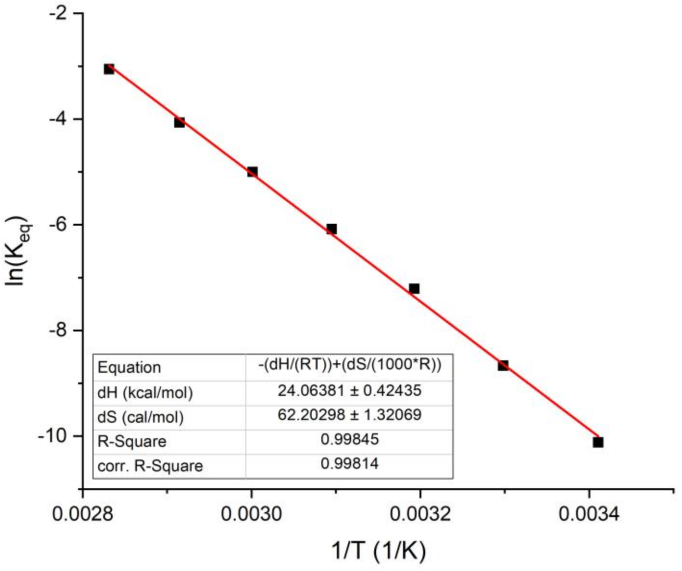

Figure S168. Van 't Hoff plot for the reversible Pd–Pd bond dissociation in **4^{iPr}-Pd**.

8.4) Exchange Kinetics from Line Broadening Analysis for the Reversible Bond Dissociation in [(^{iPr}PNP)Pd]$_2$

While EXSY experiments proved exchange processes between **4^{iPr}-Pd** and **3^{iPr}-Pd** at higher temperatures, quantification of activation parameters *via* cross-peak integration was precluded by overlapping signals and intensity limitations. Alternatively, the exchange kinetics were investigated through temperature-dependent line broadening observed for the singlet resonance of the *tert*-butyl groups in **4^{iPr}-Pd**. This broadening arises from exchange with the paramagnetic monomeric species **3^{iPr}-Pd**. The rate constant (*k*) relates directly to the additional contribution (Δv_{exc}) to the signal's full width at half-maximum (FWHM) caused by exchange:[25]

$$k = \pi \Delta v_{exc}$$

where $\Delta v_{exc} = \Delta v_{tot} - \Delta v_{min}$. Here Δv_{tot} is the observed FWHM at a given temperature T and Δv_{min} represents the intrinsic (minimum) linewidth under non-exchange conditions. The minimum linewidth (Δv_{min}) could not be directly measured due to exchange broadening arising from conformational interchange close to room temperature (c.f. 8.1). Therefore, Δv_{min} was determined by a model incorporating the Eyring-Polanyi equation:

$$\Delta v_{tot}(T) = \Delta v_{min} + \frac{1}{\pi}\left(\frac{\kappa k_B T}{h} e^{-\Delta G^{\ddagger}/RT}\right)$$

Assuming again κ = 1 and considering only $\Delta v_{tot}(T)$ values (Table S17) at least 40 K away from the coalescence temperature (T_c = 276.15 K), non-linear fitting of the experimental Δv_{tot} against T resulted in (Figure S169 left):

$$\Delta v_{min} = (1.382 \pm 0.009)\ \mathrm{Hz}$$

$$\Delta G^{\ddagger} = (19.44 \pm 0.01)\ \mathrm{kcal\ mol^{-1}}$$

Utilizing the fitted Δv_{min}, a linear Eyring-Polanyi plot of $\ln(k/T)$ against T^{-1} (Table S17, Figure S169) established values for $\Delta H^{\ddagger}$, $\Delta S^{\ddagger}$ and $\Delta G^{\ddagger}$:

$$\ln\left(\frac{k}{T}\right) = -\frac{\Delta H^{\ddagger}}{T}\left(\frac{1}{T}\right) + \ln\left(\frac{\kappa k_B}{h}\right) + \frac{\Delta S^{\ddagger}}{R}$$

$$\Delta H^{\ddagger} = (19.66 \pm 0.57)\ \mathrm{kcal\ mol^{-1}}$$

$$\Delta S^{\ddagger} = (0.64 \pm 1.67)\ \mathrm{cal\ mol^{-1}\ K^{-1}}$$

$$\Delta G^{\ddagger}_{298.15\ \mathrm{K}} = \Delta H^{\ddagger} - T\Delta S^{\ddagger} = (19.47 \pm 0.76)\ \mathrm{kcal\ mol^{-1}}$$

Table S17. Temperature-dependent linewidth contributions of the *tert*-butyl proton resonance in **4^{iPr}-Pd** and derived rate constants for the dissociation into **3^{iPr}-Pd** used in the Eyring-Polanyi fitting. Δv_{tot} and their standard deviations (**σ**) were determined by multiple independent measurements at the given temperature.

T (K)	Δv_{tot} (Hz)	$\sigma(\Delta v_{tot})$ (Hz)	Δv_{exc} (Hz)	$\sigma(\Delta v_{exc})$ (Hz)	1/T (K^{-1})	k (Hz)	$\ln(k/T)$ (ln(s^{-1} K^{-1}))	$\sigma(\ln(k/T))$ (ln(s^{-1} K^{-1}))
320.0	1.505	0.035	0.123	0.036	0.00313	0.387	-6.719	0.295
322.5	1.530	0.076	0.148	0.076	0.00310	0.465	-6.541	0.514
325.0	1.550	0.076	0.168	0.076	0.00308	0.528	-6.422	0.453
327.5	1.605	0.105	0.223	0.105	0.00305	0.701	-6.147	0.473
330.0	1.650	0.078	0.268	0.079	0.00303	0.842	-5.971	0.293
332.5	1.727	0.091	0.345	0.091	0.00301	1.083	-5.727	0.265
335.0	1.823	0.042	0.441	0.043	0.00299	1.387	-5.487	0.097
337.5	1.977	0.059	0.595	0.059	0.00296	1.868	-5.196	0.100
340.0	2.100	0.010	0.718	0.014	0.00294	2.256	-5.015	0.019

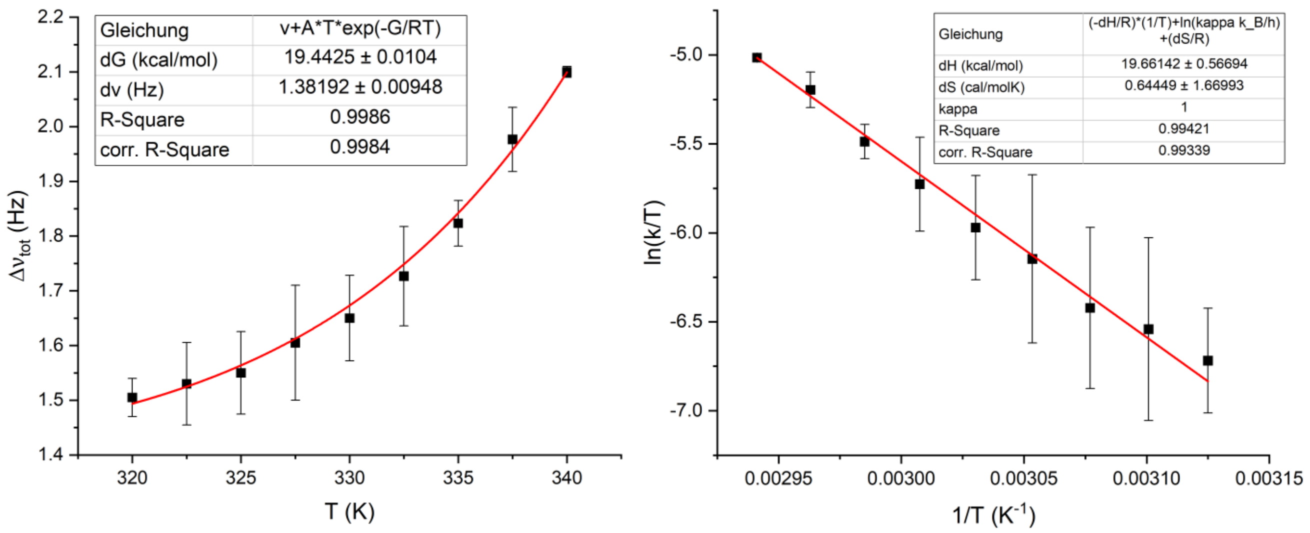

Figure S169. Exponential fit of $\Delta G^{\ddagger}$ and Δv_{min} to experimentally observed total linewidths of the tert-butyl groups resonance in **4^{iPr}-Pd** at different temperatures (left) and Eyring-Polanyi plot for the reversible Pd–Pd bond dissociation in **4^{iPr}-Pd** (right).

8.5) Thermodynamics of the Equilibrium between 7^{Et}-NiPd and 4^{Et}-Pd & 4^{Et}-Ni

Integration ratios between the ^{31}P resonances of **4^{Et}-Pd**, **4^{Et}-Ni** and **7^{Et}-NiPd** were determined at 295 K after thermal equilibrium between the hetero- and homodinuclear complexes had been reached. The equilibrium constant was determined as:

$$K_{eq} = \frac{[7^{\mathrm{Et}}\mathrm{NiPd}]^2}{[4^{\mathrm{Et}}\mathrm{Pd}][4^{\mathrm{Et}}\mathrm{Ni}]} = \frac{0.8921^2}{0.059 * 0.049} = 275.85$$

The relative Gibbs free energy at 295 K between two heterodinuclear complexes and the homodinuclear congeners was calculated as:

$$\Delta G_{295\,\mathrm{K}} = RT\ln(K_{eq}) = -3.29\ \mathrm{kcal\ mol^{-1}}$$

Therefore, **7^{Et}-NiPd** is energetically slightly more favored ($0.5 * \Delta G_{298\,\mathrm{K}} = 1.65\ \mathrm{kcal\ mol^{-1}}$) than the homodinuclear complexes.

9) Variable Temperature NMR experiments of 4^{Et}-Ni and 4^{Et}-Pd

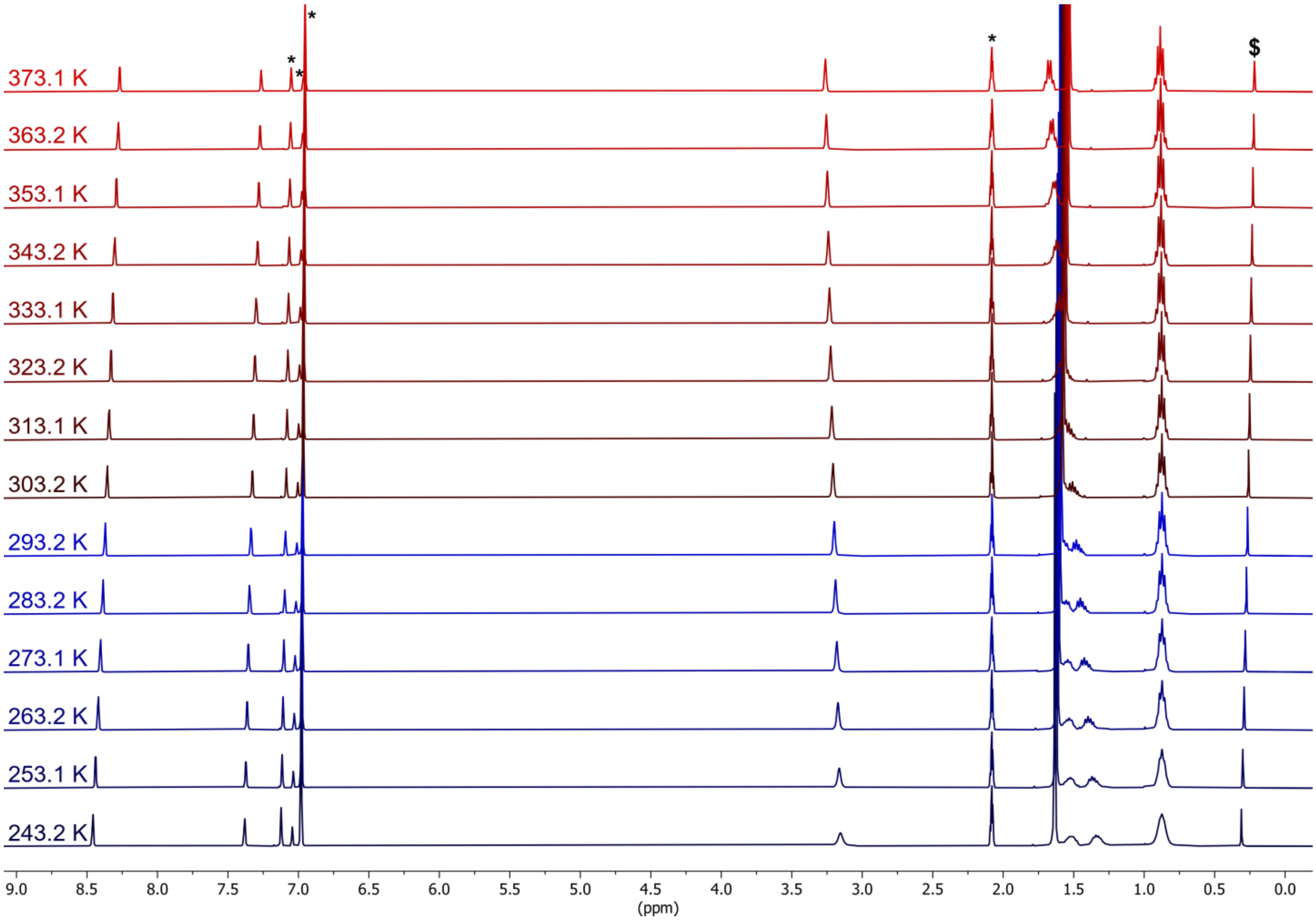

Figure S170. ^{1}H NMR spectra (tol-d_8, 400 MHz) of **$[(^{Et}PNP)Pd]_2$ (4^{Et}-Pd)** at temperatures between 243 and 373 K with an interval of 10 K (* = tol-d8, $ = grease).

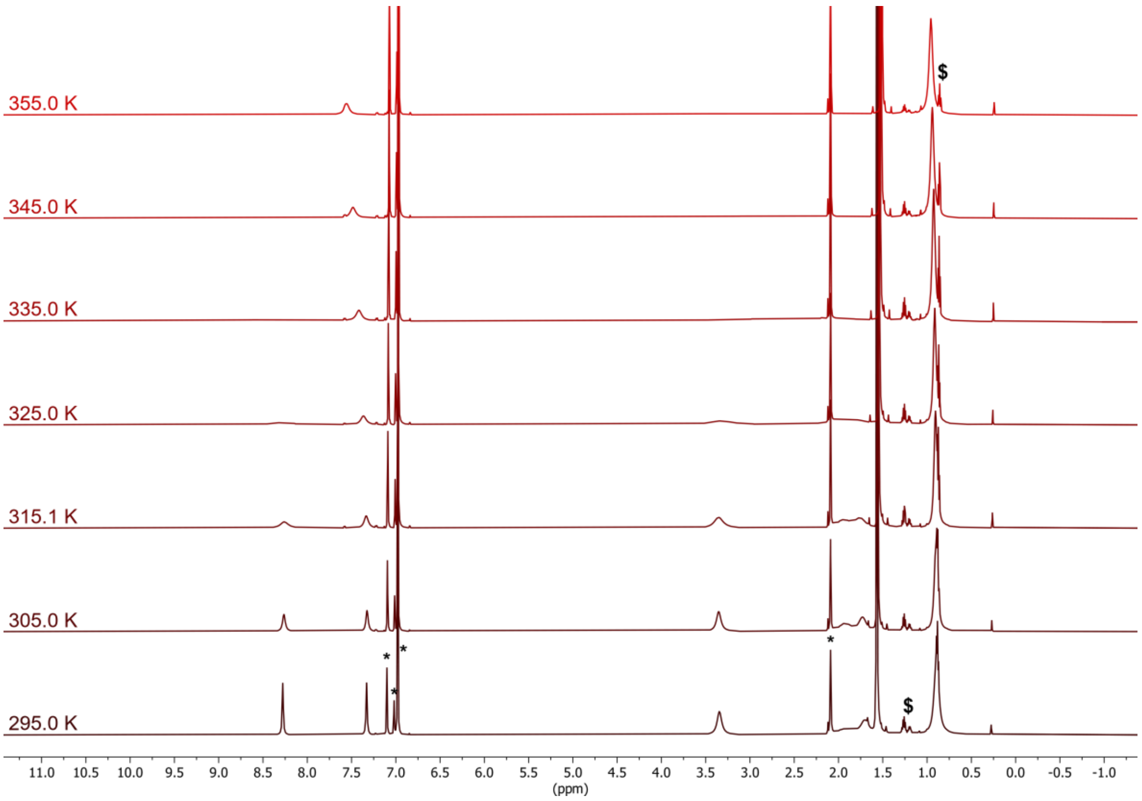

Figure S171. ^{1}H NMR spectra (tol-d_8, 600 MHz) of **[(^{Et}PNP)Ni]$_2$ (4^{Et}-Ni)** at temperatures between 295 and 355 K with an interval of 10 K (* = tol-d8, $ = *n*-pentane).

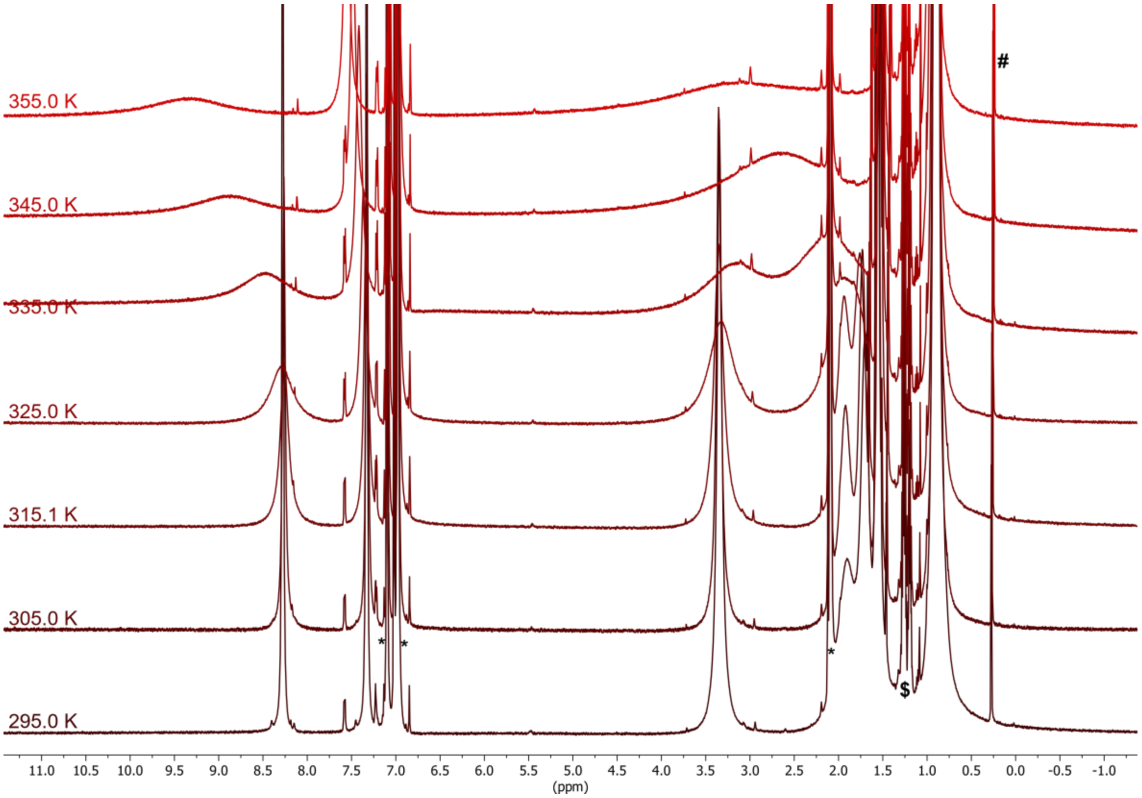

Figure S172. Intensified ^{1}H NMR spectra (tol-d_8, 600 MHz) of **[(^{Et}PNP)Ni]$_2$ (4^{Et}-Ni)** at temperatures between 295 and 355 K with an interval of 10 K (* = tol-d_8, $ = *n*-pentane, # = grease).

10) Computational Details

All density functional theory (DFT) and *ab initio* computations were performed using the ORCA program package (version 6.0.0 for XTB2 calculations and version 6.0.1 for all other computations).[26-29] Geometry optimizations and analytical frequency calculations utilized the unrestricted Kohn-Sham (UKS) r2SCAN-3c[30-32] composite method in conjunction with the modified Def2-mTZVP[30, 33-34] basis set, the SPLIT resolution of identity approximation (SPLIT-RI)[35], and the def2/J[34] auxiliary basis set. Potential energy surface (PES) scans employed the r2SCAN-3c/Def2-mSVP[36-37] level of theory. Analytical frequency calculations identified ground states and transition states based on the absence any or presence of exactly one negative (imaginary) vibrational frequency, respectively. Conformational searches were performed with ORCA's Global Optimizer Algorithm (GOAT)[38] with the semiempirical GFN2-xTB[39-40] method. Solvation effects were incorporated *via* the conductor-like polarizable continuum model (CPCM)[41-42]. CPCM for benzene was applied during optimizations and frequency calculations of structures used for NMR shielding tensor computations ([(^{iPr}PNP)MCl], [(^{iPr}PNP)MH], [(^{iPr}PNP)M^{I}] (M = Ni, Pd), and TMS), while toluene solvation parameters were used in all other geometry optimizations and frequency analyses. Very tight convergence criteria were applied throughout all computations.

Nuclear magnetic shielding tensors were calculated using the TPSSh[43-44] functional combined with Grimme's dispersion correction D3 and Becke-Johnson damping (hereinafter referred to as GD3(BJ)[45-48]). The palladium or nickel centers were described with Ahlrich's triple-ζ basis set Def2-TZVP[33] including effective core potentials (ECP), while all other elements used the Pople basis set 6-311G(d,p)[49-50]. CPCM for benzene was included.

EPR parameters (g-tensors and hyperfine coupling tensors) for complex **3^{iPr}-Pd** were computed relativistically according to a previously established level of theory for the [(^{tBu}PNP)Pd^{I}] metalloradical,[51] utilizing the UKS B3LYP[52-53]-GD3(BJ) functional with ZORA-Def2-TZVP(-f)[33] for light atoms and SARC-ZORA-TZVP[54] for palladium, incorporating the zero-order regular approximation (ZORA)[55-57]. The resolution of identity with the chain of spheres approximation (RIJCOSX)[35] was employed with the SARC/J[34] auxiliary basis set, and spin orbit coupling was considered *via* SOMF(1X)[58-59]. For complex **3^{iPr}-Ni**, EPR parameters were computed with UKS B3PW91[52, 60]-GD3(BJ)/ZORA-Def2-TZVP(-f) using ZORA, the SARC/J auxiliary basis set and SOMF(1X). Mulliken spin densities for **3^{iPr}-Ni** and corresponding spin density plots were generated at the same level of theory.

Single-point computations on **3^{iPr}-Ni**, aimed at generating initial unrestricted natural orbitals (UNOs) for subsequent complete active space self-consistent field (CASSCF)[61-62] calculations, employed the TPSSh-GD3(BJ) functional with Def2-TZVP(Ni) and 6-311G(d,p). CASSCF calculations used the RIJCOSX approximation and were corrected by second-order perturbation theory (SC-NEVPT2)[63-65]. Spin-orbit coupling was included using operators equivalent to SOMF(1X) (SOCType 3, SOCFlags 1,2,3,0, SOCMaxCenter 4), with PictureChange set to true. The complete active space was initially constructed non-relativistically employing Def2-TZVP(Ni), 6-311G(d,p) and either Def2-TZVP/C[66] or AUTOAUX[67] to generate an auxiliary correlation basis set. For ZORA relativistic calculations, the basis sets were projected onto ZORA-Def2-TZVP(-f), using SARC/J combined with AUTOAUX for generating the auxiliary correlation basis set. Computations of UV/Vis spectra, *ab initio* ligand field theory (AILFT)[68] analysis, and EPR parameters included all five doublet states within a CAS(9,5) active space. The depicted CASSCF-derived UV/Vis spectrum in the main manuscript was depicted with a peak half-width at half-height value of 0.12 eV.

Time-dependent DFT (TD-DFT) spectra were generally visualized with a peak half-width at half-height of 0.2 eV. For **3^{iPr}-Ni**, TD-DFT calculations utilized UKS B3PW91-GD3(BJ)/ZORA-Def2-TZVP(-f), incorporating ZORA, the SARC/J auxiliary basis set, SOMF(1X) and CPCM for *n*-pentane and considered 50 roots. For complexes **4^{iPr}-Pd**, **4^{Et}-Pd**, and **4^{Et}-Ni**, TD-DFT employed UKS B3LYP-GD3(BJ)/Def2-TZVP(Ni or Pd),6-311G(d,p), the def2/J auxiliary basis set, CPCM for THF and considered 50 roots. The geometries for **4^{iPr}-Pd**, **4^{Et}-Pd**, and **4^{Et}-Ni** were optimized as described in chapter 10.6.

Multiwfn 3.8[69-71] was used to analyze electronic excitations from TD-DFT calculations and to perform quantum theory of atoms in molecules (QTAIM)[72-74] analysis. Molecular geometries, molecular orbital visualization, spin density plots, and natural orbital pictures were created using Chemcraft (version 1.8)[75].

10.1) NMR Simulations for T-shaped Pd(I) and Ni(I) complexes

The paramagnetic ^{1}H and ^{13}C NMR spectra of complexes **3^{iPr}-Pd** and **3^{iPr}-Ni** were simulated by calculating isotropic chemical shifts (δ_{calc}) using the expression:[76-78]

$$\delta_{calc} = \delta_{dia} + \delta_{HF} = \delta_{dia} + \frac{1}{3}\,Tr(\boldsymbol{\chi A})$$

Here, δ_{dia} is the diamagnetic contribution, while δ_{HF}, the hyperfine shift, arises from coupling between nuclear spins and the unpaired electron density. **χ** denotes the magnetic susceptibility tensor, and **A** is the hyperfine coupling tensor for each nucleus.

The molecular structures of both complexes possess a mirror plane coinciding with the xz-plane in the chosen reference coordinate system (Figure S173), with its origin at the metal center. This symmetry simplifies the susceptibility tensor with:

$$\chi_{xy} = \chi_{yx} = \chi_{yz} = \chi_{zy} = 0$$

Therefore, only diagonal elements ($\chi_{xx}, \chi_{yy}, \chi_{zz}$) and off-diagonal elements (χ_{xz}, χ_{zx}) require consideration.

The diamagnetic term δ_{dia} was computed combining density functional theory (DFT) with empirical corrections to enhance accuracy. First the chlorido complexes [(^{iPr}PNP)MCl], the hydrido congeners [(^{iPr}PNP)MH] (M = Pd, Ni)[79], and tetramethylsilane (TMS) were optimized at the CPCM(benzene)/r^{2}SCAN-3c/Def2-mTZVP level of theory starting with initial geometries derived from X-ray structures. The diamagnetic shielding tensors were then computed employing CPCM(benzene)/TPSSh-GD3(BJ)/Def2-TZVP(Pd, Ni),6-311G(d,p) and referenced against TMS (δ_{dia}^{DFT}). Comparison with the experimental diamagnetic shifts of these compounds revealed systematic offsets of similar magnitude in both chlorido and hydrido complexes (see Table S19/S27). This implies transferable errors in DFT predicted shieldings for the target T-shaped M(I) systems. Corrected diamagnetic shifts for **3^{iPr}-Pd** and **3^{iPr}-Ni** were then obtained by applying a mean offset ($\Delta\delta$) as:

$$\delta_{dia} = \delta_{dia}^{DFT} + \Delta\delta$$

Due to the absence of a crystal structure for **3^{iPr}-Pd**, conformational sampling was performed using GOAT. The ten lowest-energy conformers were reoptimized at CPCM(benzene)/r^{2}SCAN-3c/Def2-mTZVP, for which the geometry with the lowest Gibbs-free-energy ($\Delta G = 0\ kcal/mol$) was selected for NMR and EPR (vide infra) calculations (Table S18). For **3^{iPr}-Ni,** the geometry was again first derived from its X-ray structure and optimized using the same level of theory. However, by using the same approach as for **3^{iPr}-Pd**, conformational sampling led to a slightly more stable conformer that was chosen for all further computations (Table S26).

Hyperfine coupling tensors (**A**) and diamagnetic shifts δ_{dia}^{DFT} were computed for the optimized T-shaped structures and the corrected δ_{dia} values calculated for each nucleus. The susceptibility tensor **χ** was iteratively refined to minimize the root-mean-square deviation (RMSD) between calculated (δ_{calc}) and experimental shifts using only well-resolved experimental resonances.

The paramagnetic shift δ_{HF} was further decomposed into Fermi contact (δ_{FC}) and pseudocontact (δ_{PC}) contributions:[76-77]

$$\delta_{FC} = \chi_{iso} A_{iso}$$

$$\delta_{PC} = \frac{1}{3}\,Tr\left(\Delta\boldsymbol{\chi}\boldsymbol{A_{dip}}\right)$$

with $\boldsymbol{\Delta\chi} = \boldsymbol{\chi} - \chi_{iso}\boldsymbol{I}$ and $\boldsymbol{A_{dip}} = \boldsymbol{A} - A_{iso}\boldsymbol{I}$.

Simulated spectra were generated by assigning a Lorentzian line shape to each resonance with full width at half-maximum (FWHM) values derived from transverse relaxation rates (R_2^{total}):[77, 80-82]

$$FWHM = \frac{R_2^{total}}{\pi} * \frac{2\pi}{\gamma_{nuc} B_0} * 10^6$$

with γ_{nuc} as the nuclear gyromagnetic ratio (rad T^{-1} s^{-1}), B_0 the magnetic field strength (T), and the relaxation rate[77, 80-82]

$$R_2^{total} = R_2^{SRSK} + R_2^{HF} + R_2^{Curie+CSA}$$

where the relaxation components comprise: scalar relaxation of the second kind (R_2^{SRSK}), dipolar hyperfine coupling (R_2^{HF}) and Curie and chemical shift anisotropy relaxation ($R_2^{Curie+CSA}$). These terms were computed from DFT-derived tensors (**A**, **σ**), estimated electron relaxation times (T_{1e}, T_{2e}) and reorientation/rotational correlation time:

$$\tau_R = \frac{4\pi\eta r^3}{3k_B T}$$

Where η is the solvent viscosity (benzene), r the hydrodynamic radius (estimated as half the maximum interatomic distance in Å), k_B the Boltzmann constant and T the temperature (353 K for **3^{iPr}-Pd** and 295 K for **3^{iPr}-Ni**). Exchange broadening was ignored since slow exchange correlation was assumed ($\tau_M \gg T_{1e}, T_{2e}, \tau_R$).

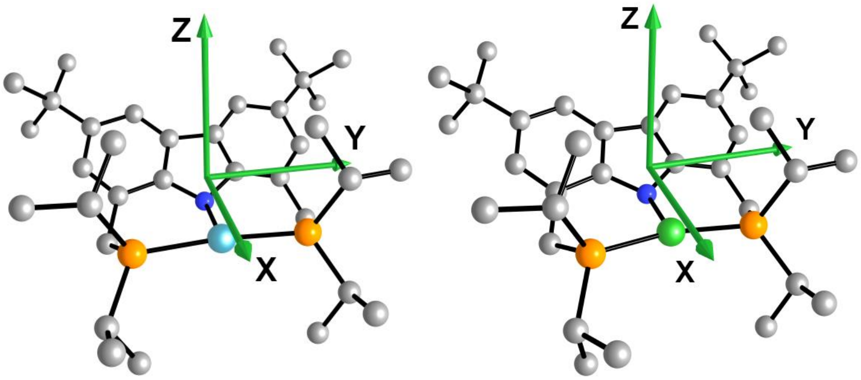

Figure S173. Molecular orientations of **3^{iPr}-Pd** and **3^{iPr}-Ni** chosen for all NMR related computations.

Table S18. Relative Gibbs free energies of the ten most stable conformers for **3^{iPr}-Pd** found using GOAT, reoptimized at the CPCM(benzene)/r^{2}SCAN-3c/Def2-mTZVP level of theory.

Conformer Entry	Relative Gibbs free Energy ΔG (kcal/mol)
1	0.00
2	0.18
3	0.62
4	0.61
5	2.48
6	1.94
7	1.87
8	2.33
9	1.33
10	3.17

Table S19. DFT calculated diamagnetic chemical shifts (δ_{dia}^{calc}) of **[LPdCl]** and **[LPdH]** (L = (^{iPr}PNP)) at the CPCM(benzene)/TPSSh-GD3(BJ)/Def2-TZVP(Pd),6-311G(d,p) level of theory in comparison to the experimentally observed values (δ_{exp}). $\Delta\delta_{PdX}$ is the difference between the experimentally found and the DFT computed shifts (X = Cl, H); the mean value of the shift differences is denoted as $\Delta\delta$; the computed diamagnetic shifts for **3^{iPr}-Pd** corrected with $\Delta\delta$ are denoted as δ_{dia}.

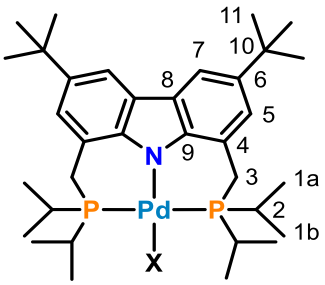

Position	δ_{exp} LPdCl (ppm)	δ_{exp} LPdH[a] (ppm)	δ_{dia}^{DFT} LPdCl (ppm)	δ_{dia}^{DFT} LPdH (ppm)	$\Delta\delta_{PdCl}$ (ppm)	$\Delta\delta_{PdH}$ (ppm)	$\Delta\delta$ (ppm)	δ_{dia}^{DFT} LPd(I) (ppm)	δ_{dia} LPd(I) (ppm)
H_{1a}	1.32	0.93	1.24	0.70	0.08	0.24	0.16	0.76	0.92
H_{1b}	0.90	0.95	1.14	1.31	-0.24	-0.36	-0.30	1.47	1.17
H_2	2.35	1.72	2.44	2.08	-0.09	-0.36	-0.23	2.09	1.87
H_3	2.87	3.09	3.13	3.33	-0.27	-0.24	-0.25	3.31	3.06
H_5	7.24	7.34	7.37	7.43	-0.13	-0.09	-0.11	7.31	7.20
H_7	8.36	8.49	8.08	8.14	0.28	0.35	0.32	7.98	8.29
H_{11}	1.56	1.63	1.42	1.44	0.14	0.19	0.17	1.39	1.56
C_{1a}	19.4	19.3	21.3	20.9	-1.9	-1.6	-1.8	21.6	19.9
C_{1b}	17.7	18.6	21.1	20.2	-3.4	-1.6	-2.5	20.4	17.9
C_2	24.4	24.7	33.8	32.8	-9.4	-8.1	-8.7	32.9	24.2
C_3	19.9	23.6	28.5	29.1	-8.6	-5.5	-7.0	30.3	23.3
C_4	119.7	120.1	121.0	120.1	-1.3	0.0	-0.7	122.3	121.6
C_5	123.7	123.5	124.7	124.6	-1.0	-1.1	-1.0	124.7	123.7
C_6	139.6	137.9	140.4	137.8	-0.8	0.1	-0.3	138.8	138.5
C_7	115.3	115.7	115.5	115.3	-0.2	0.4	0.1	115.5	115.6
C_8	126.6	126.1	126.5	125.0	0.1	1.1	0.6	128.4	129.0
C_9	148.4	148.9	150.2	149.9	-1.8	-1.0	-1.4	150.7	149.3
C_{10}	34.3	34.7	39.9	39.9	-5.6	-5.2	-5.4	39.9	34.5
C_{11}	32.1	32.7	33.2	33.4	-1.1	-0.7	-0.9	33.3	32.4

[a] [(^{iPr}PNP)PdH] was prepared according to the literature.[79] The centres of the ^{1}H and ^{13}C resonances were determined within this work.

Table S20. Components of ^{1}H hyperfine coupling tensors for **3^{iPr}-Pd** computed with B3LYP-GD3(BJ)/SARC-ZORA-TZVP(Pd), ZORA-Def2-TZVP(-f) in units of ppm Å^{-3}.

Atom No.[a]	A_{xx}	A_{xy}	A_{xz}	A_{yx}	A_{yy}	A_{yz}	A_{zx}	A_{zy}	A_{zz}
38	422.6	578.2	-234.1	615.6	692.8	-161.1	-223.6	-164.9	-98.9
39	1604.3	242.7	-483.7	246.2	1334.3	-227.2	-502.7	-228.7	1089.8
40	1612.1	-227.4	-490.8	-230.9	1331.4	236.7	-510.1	238.1	1117.1
41	418.6	-568.9	-254.1	-606.2	680.2	186.3	-245.5	190.9	-88.1
42	-939.8	-697.3	50.4	-772.5	154.6	-415.2	69.6	-435.5	-1455.6
43	579.8	-1080.5	550.2	-1205.5	1458.8	-1677.7	653.1	-1767.8	-50.8
44	531.7	1074.3	584.0	1200.1	1367.3	1744.4	696.1	1840.3	-21.1

45	-929.0	692.0	70.0	767.2	151.3	456.2	91.3	476.8	-1429.2
46	66.0	144.8	-256.8	152.5	-136.7	-121.2	-265.1	-121.6	10.0
47	50.9	140.9	-135.4	147.8	-65.4	-92.8	-140.2	-94.2	-76.6
48	218.0	151.2	-189.4	159.1	-139.8	-65.3	-196.3	-68.5	-135.7
49	294.4	204.0	-56.3	211.9	319.0	-56.9	-58.4	-60.1	123.4
50	-48.6	246.8	-34.7	260.2	134.7	33.3	-34.4	28.5	-250.2
51	146.2	287.4	45.5	296.5	-28.1	-5.3	42.5	-12.5	-217.7
52	229.3	157.4	-102.4	166.4	342.2	-171.8	-106.5	-172.8	185.5
53	-115.5	176.2	-110.9	188.3	-9.2	-301.0	-121.3	-301.1	24.8
54	-113.4	152.3	-113.9	168.2	184.6	-210.6	-119.6	-211.7	-245.2
55	307.1	-202.8	-62.8	-210.6	328.2	64.2	-65.3	67.6	141.1
56	153.5	-285.1	34.3	-294.3	-34.6	15.6	30.7	22.7	-219.5
57	-47.6	-248.9	-41.3	-262.3	133.2	-24.9	-41.7	-19.6	-249.3
58	52.0	-136.1	-140.7	-142.8	-65.9	94.9	-145.9	96.3	-63.0
59	59.0	-137.4	-262.2	-145.0	-144.5	120.0	-271.1	120.2	23.8
60	214.8	-144.7	-197.3	-152.6	-140.3	66.5	-204.6	69.6	-123.6
61	-115.6	-150.2	-118.1	-166.0	175.9	217.5	-124.2	219.1	-233.3
62	-117.3	-170.7	-113.2	-182.6	-20.1	303.6	-124.1	303.9	34.1
63	212.0	-153.6	-106.2	-162.4	318.5	175.8	-110.7	177.0	179.1
64	-390.5	172.8	-70.9	175.0	1360.5	198.1	-78.1	194.5	-267.4
65	546.6	44.3	115.9	38.7	1535.4	584.7	113.5	588.5	894.3
66	-389.0	-310.7	-79.2	-340.5	685.7	462.0	-89.7	457.7	-677.0
67	-1562.9	-372.6	-763.3	-378.2	-1121.4	2171.4	-844.6	2280.3	2382.0
68	-398.6	-465.9	-540.0	-516.3	98.3	1241.2	-631.7	1274.7	272.4
69	-866.2	-42.3	-70.5	-47.8	-269.4	924.0	-85.7	954.9	389.2
70	-912.9	-129.2	292.3	-142.2	-33.1	-1475.7	316.8	-1529.7	841.2
71	-2383.7	501.9	-1277.6	561.4	-884.0	-2507.7	-1453.9	-2593.5	2297.7
72	-1019.7	322.5	-492.4	336.6	-320.0	-926.7	-522.1	-953.5	174.9
73	-179.1	827.0	-576.2	871.0	-50.1	-647.6	-611.9	-647.3	-885.2
74	1137.7	2560.6	-1037.9	2696.6	591.7	-1153.8	-1104.8	-1158.8	-2540.3
75	-9.4	1246.5	119.6	1304.2	876.3	102.7	114.3	111.9	-924.4
76	-836.9	66.1	-107.3	73.9	-259.6	-914.0	-126.6	-944.3	376.5
77	-363.8	484.7	-545.9	536.4	113.3	-1213.9	-638.5	-1245.9	226.0
78	-1437.2	403.4	-841.6	415.7	-1115.6	-2121.6	-944.6	-2231.3	2288.4
79	473.9	-23.7	94.5	-16.3	1462.3	-580.5	91.1	-583.8	826.4
80	-394.2	-161.0	-77.3	-162.1	1341.9	-212.2	-84.7	-207.3	-284.0
81	-390.0	320.1	-74.1	351.5	688.3	-444.2	-84.5	-438.6	-684.2
82	-942.3	94.5	246.9	104.7	-32.2	1485.9	265.5	1538.3	848.2
83	-993.0	-357.2	-540.7	-372.9	-308.7	922.2	-572.0	947.9	132.8
84	-2352.7	-590.5	-1449.1	-655.8	-833.3	2545.5	-1633.8	2626.7	2171.8
85	-21.0	-1241.3	161.9	-1299.0	880.4	-163.4	158.1	-172.9	-910.9
86	1202.5	-2611.3	-877.4	-2749.8	601.6	1032.8	-936.0	1036.3	-2646.8
87	-137.9	-854.4	-544.4	-899.5	-44.7	612.6	-578.8	611.9	-942.6
88	195.3	891.5	963.8	949.3	2109.3	1866.9	1014.2	1895.0	622.7
89	2585.3	121.0	-432.8	134.0	4964.3	-995.1	-434.2	-999.1	2776.6
90	2681.2	-149.1	-451.8	-163.1	5062.2	984.5	-453.8	989.3	2797.5

91	128.6	-815.8	912.1	-867.7	2097.8	-1886.2	959.3	-1914.1	730.8

[a] Atom No. Refers to the atom counts position in the cartesian coordinates of **3^{iPr}-Pd**.

Table S21. Components of ^{13}C hyperfine coupling tensors for **3^{iPr}-Pd** computed with B3LYP-GD3(BJ)/SARC-ZORA-TZVP(Pd),ZORA-Def2-TZVP(-f) in units of ppm Å^{-3}.

Atom No.[a]	A_{xx}	A_{xy}	A_{xz}	A_{yx}	A_{yy}	A_{yz}	A_{zx}	A_{zy}	A_{zz}
4	-6686.0	3847.4	-6848.4	3027.8	1219.0	-5089.6	-7524.8	-4876.7	-3006.6
5	5542.6	2563.2	6732.4	3361.2	2431.6	1170.6	6567.9	1497.9	9886.3
6	-6844.0	979.7	-3142.3	1015.3	-4860.3	-56.4	-3154.7	-141.7	-9208.0
7	9962.7	-349.8	8498.4	-552.7	1802.9	-1357.8	8454.4	-1576.7	10965.6
8	-5077.6	685.1	-3494.1	613.9	-2173.8	-743.6	-3588.5	-649.5	-5409.4
9	16689.5	547.9	2674.5	924.4	16020.4	337.8	2681.7	509.8	33938.9
10	16239.3	-670.7	2703.7	-1024.1	15605.4	-943.2	2698.5	-1110.1	33320.2
11	-5137.2	-557.5	-3514.1	-483.4	-2272.7	852.4	-3606.5	759.2	-5404.6
12	10021.5	57.6	8504.0	256.1	1702.0	1030.1	8466.4	1247.4	10938.8
13	-6877.3	-891.2	-3193.1	-928.4	-4843.5	188.9	-3205.1	273.3	-9142.3
14	5544.2	-2748.5	6668.0	-3537.3	2398.3	-1334.2	6478.7	-1662.0	9514.9
15	-7076.1	-3577.3	-6848.4	-2742.9	568.7	5213.3	-7499.5	5018.0	-2989.4
16	-17854.5	729.9	-1014.1	529.1	-14184.7	-277.7	-1036.5	-515.0	-17708.0
17	-17925.7	-690.7	-1032.5	-484.2	-14241.5	309.3	-1039.7	543.1	-17828.4
18	-2378.7	316.5	-62.8	329.4	-2454.0	-195.7	-67.6	-198.5	-2599.6
19	246.9	204.1	-228.1	220.5	34.0	-99.6	-233.3	-96.0	69.2
20	3199.9	479.0	86.8	493.0	3540.5	198.9	86.8	186.1	2965.8
21	3717.4	-24.4	20.4	-9.2	4223.2	-497.8	23.6	-489.0	3743.8
22	-2375.5	-315.3	-72.0	-328.2	-2461.6	200.1	-78.0	203.7	-2583.6
23	3323.2	-495.8	82.8	-509.8	3652.1	-186.9	82.0	-173.7	3068.3
24	269.7	-198.1	-234.5	-214.1	54.8	104.4	-240.9	100.4	98.8
25	3588.9	26.0	15.6	10.8	4073.9	498.6	17.6	490.2	3624.9
26	15246.0	-1052.1	-784.0	-839.6	20145.9	3697.4	-880.0	3782.6	18234.6
27	7258.2	-807.2	-190.9	-781.6	11619.1	978.1	-238.1	967.7	7240.2
28	1740.0	-290.5	-387.4	-308.5	3003.4	1803.7	-458.2	1902.5	3413.2
29	33846.0	3412.8	-4032.3	3552.9	36512.9	-4936.3	-4099.1	-5034.8	35843.0
30	11381.4	-137.7	-405.0	-120.9	14150.3	-2206.2	-394.6	-2253.1	13600.4
31	13826.1	1163.4	-520.2	1246.6	17515.9	-1089.7	-595.5	-1010.1	12309.4
32	13816.9	1188.2	-896.4	983.7	18517.2	-3472.8	-999.7	-3546.9	16390.2
33	1220.6	309.3	-418.6	335.0	2339.5	-1757.6	-494.2	-1848.9	2788.9
34	6934.1	834.0	-180.1	809.6	11154.5	-892.0	-226.9	-876.8	6865.6
35	35358.3	-3713.4	-4218.8	-3856.6	37765.5	4917.5	-4284.4	5022.8	36743.4
36	12188.6	94.4	-479.8	77.2	15102.3	2243.1	-473.0	2285.5	14358.8
37	14029.8	-1200.2	-456.6	-1289.0	17690.8	1009.3	-530.7	932.4	12419.1

[a] Atom No. Refers to the atom counts position in the cartesian coordinates of **3^{iPr}-Pd**.

Table S22. Fitted susceptibility tensor (χ) for **3[iPr]-Pd** in cm^3 K mol^{-1}.

0.04812 ± 0.00306	0	-0.09525 ± 0.00204
0	0.08056 ± 0.00278	0
-0.09525 ± 0.00204	0	0.28187 ± 0.00356

Table S23. Fitted mean values (for chemically equivalent atoms) of the diamagnetic (δ_{dia}), fermi contact (δ_{FC}) and pseudo contact (δ_{PC}) components of the ^{1}H and ^{13}C chemical shifts together with the calculated total shift (δ_{calc}), standard error (σ) and linewidth (FWHM) for **3[iPr]-Pd**.

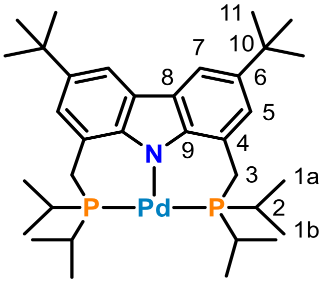

Group	δ_{dia} (ppm)	δ_{FC} (ppm)	δ_{PC} (ppm)	δ_{calc} (ppm)	σ(δ_{calc}) (ppm)	FWHM (ppm)
H_{1a}	0.92	-1.37	1.64	1.19	0.09	0.74
H_{1b}	1.17	0.40	3.50	5.07	0.06	0.63
H_2	1.87	18.03	-2.42	17.49	0.33	4.94[a]
H_3	3.06	-0.39	-3.95	-1.28	0.07	0.77
H_5	7.20	2.73	-0.87	9.07	0.06	0.16
H_7	8.29	10.91	0.73	19.93	0.19	1.26[a]
H_{11}	1.56	0.23	0.03	1.82	0.02	0.02
C_{1a}	19.86	68.96	-0.81	88.02	1.17	18.61[a]
C_{1b}	17.94	88.89	0.33	107.17	1.53	21.45[a]
C_2	24.20	214.66	11.80	250.66	3.66	133.79[a]
C_3	23.25	-134.48	0.14	-111.09	2.39	46.18[a]
C_4	121.64	47.62	-9.47	159.79	1.57	8.98[a]
C_5	123.67	-56.33	3.07	70.42	1.24	8.81[a]
C_6	138.50	61.20	-19.95	179.75	1.95	14.19[a]
C_7	115.59	-34.35	9.27	90.51	0.92	3.79[a]
C_8	129.04	177.72	39.68	346.44	3.48	85.44[a]
C_9	149.30	-24.23	29.42	154.49	1.27	6.37[a]
C_{10}	34.50	-20.03	-0.26	14.21	0.35	1.03[a]
C_{11}	32.39	19.55	-0.43	51.50	0.33	1.43[a]

[a] Severe line broadening render these signals invisible in experiment.

Table S24. Relaxation Parameters used for the simulation of ^{1}H and ^{13}C signal linewidths for **3^{iPr}-Pd.**

T_{1e}	5.041*10^{-9} s
T_{2e}	9.523*10^{-9} s
τ_R	9.481*10^{-8} s
r	7.030 Å
η (benzene, 353 K)	0.318 mPa*s

Table S25. Experimental chemical shifts and their corresponding weights used for the fitting procedure of the calculated **3^{iPr}-Pd** NMR signals.

Group	δ_{exp} (ppm)	weight
H_{1a}	1.182	1
H_{1b}	5.071	1
H_3	-1.277	1
H_5	9.066	1
H_{11}	1.871	1

Table S26. Relative Gibbs free energies of the ten most stable conformers for **3^{iPr}-Ni** found using GOAT, reoptimized at the CPCM(benzene)/r^{2}SCAN-3c/Def2-mTZVP level of theory. The energy of the geometry derived from the molecular solid-state X-ray structure is appended as 11th structure.

Conformer Entry	Relative Gibbs free Energy ΔG (kcal/mol)
1	0.22
2	0.00
3	0.86
4	1.98
5	0.55
6	0.81
7	2.40
8	2.61
9	1.88
10	1.31
11 (X-ray)	1.95

Table S27. DFT calculated diamagnetic chemical shifts (δ_{dia}^{calc}) of **[LNiCl]** and **[LNiH]** (L = (^{iPr}PNP)) at the CPCM(benzene)/TPSSh-GD3(BJ)/Def2-TZVP(Ni),6-311G(d,p) level of theory in comparison to the experimentally observed values (δ_{exp}). $\Delta\delta_{PdX}$ is the difference between the experimentally found and the DFT computed shifts (X = Cl, H); the mean value of the shift differences is denoted as $\Delta\delta$; the computed diamagnetic shifts for **3^{iPr}-Ni** corrected with $\Delta\delta$ are denoted as δ_{dia}..

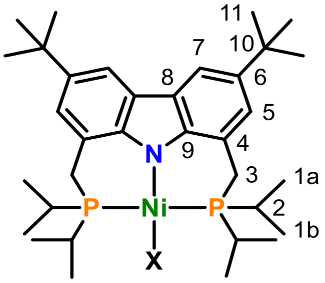

Position	δ_{exp} LNiCl (ppm)	δ_{exp} LNiH[a] (ppm)	δ_{dia}^{DFT} LNiCl (ppm)	δ_{dia}^{DFT} LNiH (ppm)	$\Delta\delta_{NiCl}$ (ppm)	$\Delta\delta_{NiH}$ (ppm)	$\Delta\delta$ (ppm)	δ_{dia}^{DFT} LNi(I) (ppm)	δ_{dia} LNi(I) (ppm)
H_{1a}	1.47	0.98	1.31	0.74	0.15	0.24	0.19	0.73	0.92
H_{1b}	1.07	0.99	1.27	1.31	-0.20	-0.32	-0.26	1.69	1.43
H_2	2.68	1.70	2.37	2.04	0.30	-0.34	-0.02	2.43	2.41
H_3	2.19	2.94	3.05	3.27	-0.86	-0.33	-0.60	3.92	3.32
H_5	7.17	7.33	7.30	7.38	-0.14	-0.05	-0.10	7.44	7.35
H_7	8.28	8.44	8.05	8.09	0.24	0.35	0.30	7.84	8.13
H_{11}	1.54	1.61	1.41	1.43	0.13	0.19	0.16	1.39	1.55
C_{1a}	19.9	18.1	21.7	20.7	-1.8	-2.6	-2.2	22.7	20.6
C_{1b}	17.7	18.8	21.5	20.2	-3.8	-1.3	-2.5	20.8	18.2
C_2	24.2	24.7	33.5	32.3	-9.3	-7.6	-8.4	32.1	23.7
C_3	19.0	21.6	28.4	27.8	-9.4	-6.2	-7.8	30.3	22.5
C_4	119.6	120.3	120.8	120.9	-1.2	-0.7	-0.9	121.1	120.2
C_5	122.9	122.9	123.9	124.0	-1.1	-1.0	-1.1	123.7	122.7
C_6	139.8	138.1	140.1	138.1	-0.3	0.0	-0.2	137.0	136.8
C_7	114.9	115.0	115.3	115.1	-0.4	-0.1	-0.3	115.1	114.8
C_8	127.4	126.0	127.2	124.8	0.2	1.2	0.7	126.9	127.6
C_9	148.6	147.8	150.2	149.2	-1.6	-1.4	-1.5	149.5	148.0
C_{10}	34.3	34.4	39.8	39.8	-5.6	-5.5	-5.5	39.8	34.3
C_{11}	32.1	32.4	33.2	33.3	-1.1	-1.0	-1.0	33.4	32.3

[a] [(^{iPr}PNP)NiH] was prepared according to the literature.[79] The centres of the ^{1}H and ^{13}C resonances were determined within this work.

Table S28. Components of ^{1}H hyperfine coupling tensors for **3^{iPr}-Ni** computed with B3PW91-GD3(BJ)/ZORA-Def2-TZVP(-f) in units of ppm Å^{-3}.

Atom No.[a]	A_{xx}	A_{xy}	A_{xz}	A_{yx}	A_{yy}	A_{yz}	A_{zx}	A_{zy}	A_{zz}
38	87.3	631.8	-81.6	631.8	522.7	-111.8	-81.7	-111.8	-360.3
39	941.9	230.8	-358.0	230.8	265.2	-111.1	-358.1	-111.1	295.8
40	948.9	-231.4	-357.4	-231.4	271.2	110.1	-357.5	110.1	301.9
41	95.2	-634.3	-83.8	-634.4	524.5	112.0	-83.8	112.0	-353.6
42	-2961.7	-834.8	81.7	-834.8	-985.7	-437.4	81.6	-437.3	-3259.9
43	-631.6	-1625.1	1018.9	-1625.2	1335.3	-2177.5	1018.9	-2177.4	-613.1
44	-643.8	1657.8	1045.6	1658.0	1332.4	2226.1	1045.6	2226.0	-618.1

45	-2983.0	838.3	91.7	838.4	-1019.3	452.2	91.6	452.1	-3259.7
46	91.9	124.9	-96.8	124.9	-27.0	-61.3	-96.8	-61.3	-60.2
47	202.8	145.5	-117.0	145.5	-83.8	-48.6	-117.1	-48.6	-109.3
48	128.8	146.9	-191.2	146.9	-92.9	-96.2	-191.2	-96.2	-43.9
49	59.3	260.6	-17.7	260.7	111.6	-11.0	-17.7	-11.0	-182.3
50	162.5	225.5	-6.7	225.5	-10.5	-8.3	-6.7	-8.3	-162.5
51	103.3	164.0	-38.3	164.0	70.0	-34.7	-38.3	-34.7	-70.6
52	-16.3	215.6	-180.9	215.7	29.4	-218.1	-180.9	-218.1	-26.9
53	-43.4	246.4	-112.8	246.4	174.4	-174.8	-112.9	-174.8	-152.5
54	29.4	155.0	-91.8	155.0	75.7	-110.2	-91.8	-110.2	-45.6
55	103.0	-163.9	-38.3	-163.9	69.6	34.6	-38.3	34.6	-70.9
56	161.6	-225.4	-6.1	-225.4	-9.7	7.6	-6.2	7.6	-162.1
57	59.5	-260.9	-18.4	-261.0	112.4	11.9	-18.5	11.9	-182.3
58	204.0	-145.0	-115.2	-145.0	-84.1	47.5	-115.2	47.5	-110.3
59	92.2	-124.6	-96.1	-124.6	-28.5	60.3	-96.1	60.3	-61.9
60	130.6	-145.7	-189.5	-145.7	-93.4	94.2	-189.5	94.2	-45.8
61	-13.6	-215.4	-182.3	-215.4	25.6	216.4	-182.4	216.5	-25.5
62	31.6	-154.9	-92.5	-155.0	75.0	110.0	-92.5	110.0	-44.2
63	-42.2	-246.8	-114.6	-246.9	171.7	176.2	-114.7	176.3	-150.9
64	-464.1	92.9	63.1	92.9	547.1	566.2	63.0	566.3	-161.8
65	-537.3	-259.6	-78.7	-259.6	874.2	454.1	-78.7	454.3	-560.9
66	-489.9	253.1	1.7	253.1	1142.7	288.4	1.6	288.6	-442.2
67	-624.4	-745.9	-762.4	-746.0	398.7	1494.2	-762.6	1494.2	330.0
68	-766.0	-47.8	-88.6	-47.8	-45.2	1066.6	-88.7	1066.6	655.7
69	-1886.4	-366.3	-871.4	-366.3	-1212.2	2824.2	-871.6	2824.3	3676.2
70	-1125.7	177.5	-106.8	177.8	213.6	-1790.0	-107.2	-1789.9	1112.7
71	-1410.9	1134.6	-2389.3	1135.5	-1517.1	-2301.2	-2391.0	-2300.8	2341.7
72	-685.9	515.3	-651.7	515.6	-232.7	-907.7	-652.0	-907.5	183.3
73	251.4	1385.8	192.1	1386.0	705.3	170.9	192.5	171.4	-869.0
74	6.7	939.5	-481.7	939.9	-222.9	-453.8	-481.5	-453.4	-994.5
75	2037.1	2693.1	-761.1	2694.3	-533.8	-593.5	-760.1	-592.6	-2528.2
76	-697.9	809.5	-918.1	809.6	306.9	-1735.4	-918.4	-1735.3	641.4
77	-2118.8	61.7	-287.6	61.7	-1428.2	-2701.9	-287.8	-2702.0	3795.7
78	-871.7	32.2	-60.3	32.3	-150.9	-1066.2	-60.4	-1066.3	552.8
79	-517.0	314.4	-113.0	314.4	845.2	-507.6	-113.0	-507.6	-549.2
80	-486.4	-61.2	45.7	-61.2	514.7	-560.8	45.7	-560.9	-200.4
81	-502.4	-128.8	-20.3	-128.8	1146.1	-262.7	-20.3	-262.8	-448.0
82	-179.0	-500.7	-536.0	-501.0	301.7	868.9	-536.2	868.5	619.4
83	-1451.7	-654.6	-1150.0	-655.2	-733.1	2312.2	-1151.0	2311.6	2415.8
84	-821.2	-101.2	0.4	-101.4	505.2	1347.3	0.2	1347.1	444.2
85	632.7	-1012.3	-631.7	-1012.7	108.9	465.8	-631.5	465.4	-587.9
86	797.2	-2023.5	297.2	-2023.7	128.9	-232.3	297.9	-232.9	-1492.7
87	2958.0	-2824.4	-2357.6	-2825.8	-753.6	1077.5	-2356.7	1076.2	-2189.9
88	-430.3	1295.2	1017.8	1295.0	1674.9	2017.8	1017.8	2018.3	401.4
89	277.9	508.5	-343.2	508.8	2746.3	-871.9	-343.3	-871.6	347.1
90	230.5	-799.2	-396.8	-799.3	2675.7	573.5	-396.8	573.2	138.3

91	-706.7	-1257.6	874.5	-1257.5	1460.4	-1808.1	874.5	-1808.4	-46.7

[a] Atom No. Refers to the atom counts position in the cartesian coordinates of **3^{iPr}-Ni**.

Table S29. Components of ^{13}C hyperfine coupling tensors for **3^{iPr}-Ni** computed with B3PW91-GD3(BJ)/ZORA-Def2-TZVP(-f) in units of ppm Å^{-3}.

Atom No.[a]	A_{xx}	A_{xy}	A_{xz}	A_{yx}	A_{yy}	A_{yz}	A_{zx}	A_{zy}	A_{zz}
4	6131.7	2844.9	-2601.6	2845.3	4873.1	-3133.1	-2602.0	-3133.1	4778.7
5	2690.5	2729.3	437.0	2729.7	3494.1	559.1	436.6	559.1	1539.5
6	-900.4	929.6	-629.1	930.0	-602.3	-239.3	-629.1	-239.3	-2332.7
7	1334.2	367.8	730.3	368.2	94.8	-471.0	730.3	-471.0	757.6
8	1365.0	651.9	-994.1	651.9	665.5	-217.3	-994.5	-217.3	-304.1
9	10003.5	476.6	-1786.4	476.6	7383.5	200.5	-1786.4	200.5	11356.6
10	9996.3	-472.2	-1778.4	-472.6	7362.3	-198.5	-1778.4	-198.5	11367.8
11	1395.5	-659.1	-1005.7	-659.1	686.3	203.3	-1006.1	203.3	-326.6
12	1346.6	-361.4	723.1	-361.8	98.0	472.6	722.7	472.6	734.7
13	-881.6	-930.0	-641.9	-930.0	-597.9	240.5	-641.9	240.5	-2346.7
14	2669.3	-2706.1	426.6	-2706.1	3444.0	-568.3	426.6	-568.3	1557.9
15	6292.6	-2855.4	-2608.8	-2855.8	5120.0	3175.1	-2609.2	3175.1	5024.4
16	-13053.8	-954.1	-347.8	-954.1	-9843.1	-395.8	-347.8	-395.8	-14231.5
17	-13656.1	866.0	-402.6	866.0	-10257.3	406.2	-402.6	406.2	-14646.1
18	-160.5	286.1	-133.7	286.5	-290.5	-119.3	-134.1	-119.3	-457.8
19	180.9	168.9	-147.7	169.3	-16.4	-75.2	-147.7	-75.2	-54.0
20	497.8	260.5	-26.4	260.5	458.6	-13.2	-26.4	-13.2	230.1
21	433.8	204.9	-120.5	204.9	534.7	-192.5	-120.5	-192.5	329.4
22	-153.3	-286.1	-133.7	-286.1	-287.7	118.5	-133.7	118.5	-453.8
23	483.4	-259.7	-27.2	-259.7	444.6	14.0	-27.2	14.0	215.7
24	175.7	-167.7	-146.9	-167.7	-23.6	73.2	-147.3	73.2	-61.2
25	443.4	-204.5	-121.7	-204.5	541.5	192.9	-121.7	192.9	338.6
26	214.9	-345.8	-705.1	-345.8	4492.1	2595.2	-705.5	2595.2	1581.1
27	2264.3	-126.1	-36.4	-126.1	4801.5	651.1	-36.4	651.5	2401.5
28	-5521.0	-264.1	-367.0	-264.1	-4200.8	2097.0	-367.0	2097.0	-3370.4
29	23962.5	3560.9	-3490.9	3561.7	25885.1	-3813.8	-3490.9	-3813.0	24420.0
30	-1231.8	687.9	-972.1	688.7	117.7	-2078.6	-972.9	-2078.2	610.7
31	4834.7	1688.4	-481.8	1689.2	6159.7	-564.7	-481.4	-563.9	2861.8
32	-937.6	226.5	-599.1	226.5	3096.3	-2340.3	-599.1	-2340.3	399.8
33	-5198.5	189.7	-328.6	190.1	-3849.0	-2250.3	-328.6	-2250.3	-2813.7
34	1780.4	309.7	-86.0	309.7	4097.9	-700.3	-86.0	-700.3	1859.7
35	23062.5	-3729.8	-3492.9	-3730.2	25027.4	3923.1	-3493.3	3922.3	22944.5
36	-3642.1	-596.3	-670.3	-596.7	-2431.6	1654.8	-670.7	1654.4	-1969.3
37	17060.1	-2750.9	-842.8	-2751.3	17985.3	557.5	-842.4	556.7	14297.6

[a] Atom No. Refers to the atom counts position in the cartesian coordinates of **3^{iPr}-Ni**.

Table S30. Fitted susceptibility tensor (χ) for **3^iPr^-Ni** in cm³ K mol⁻¹.

0.21074 ± 0.08611	0	-0.09115 ± 0.06825
0	0.55171 ± 0.05256	0
-0.09115 ± 0.06825	0	0.29450 ± 0.09414

Table S31. Fitted mean values (for chemically equivalent atoms) of the diamagnetic (δ_{dia}), fermi contact (δ_{FC}) and pseudo contact (δ_{PC}) components of the ^{1}H and ^{13}C chemical shifts together with the calculated total shift (δ_{calc}), standard error (σ) and linewidth (FWHM) for **3^{iPr}-Ni**.

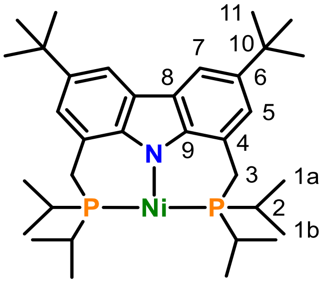

Group	δ_{dia} (ppm)	δ_{FC} (ppm)	δ_{PC} (ppm)	δ_{calc} (ppm)	σ(δ_{calc}) (ppm)	FWHM (ppm)
H_{1a}	0.92	-1.38	1.32	0.86	3.50	1.94
H_{1b}	1.43	-0.21	5.05	6.27	3.52	0.89
H_2	2.41	18.21	9.06	29.68	2.87	2.05
H_3	3.32	-29.71	7.04	-19.35	3.77	4.89
H_5	7.35	2.14	3.00	12.49	1.42	0.16
H_7	8.13	12.56	-0.76	19.93	2.45	0.38
H_{11}	1.55	0.10	0.40	2.05	0.71	0.02
C_{1a}	20.56	79.43	8.02	108.01	5.49	24.73
C_{1b}	18.22	17.98	9.34	45.54	3.98	2.06
C_2	23.67	320.13	22.21	366.00	21.49	89.13[a]
C_3	22.54	-314.37	18.63	-273.20	21.20	47.48[a]
C_4	120.19	63.94	3.40	187.54	5.42	2.64
C_5	122.66	-31.82	6.07	96.91	6.23	0.67
C_6	136.79	18.13	-8.16	146.77	5.54	0.28
C_7	114.80	14.46	3.30	132.56	7.14	0.30
C_8	127.58	238.70	-6.55	359.74	16.60	27.72[a]
C_9	147.96	133.83	7.27	289.06	16.71	10.50[a]
C_{10}	34.29	-7.49	0.36	27.16	1.23	0.04
C_{11}	32.32	7.13	0.48	39.94	0.79	0.04

[a] Severe line broadening render these signals invisible in experiment.

Table S32. Relaxation parameters used for the simulation of ^{1}H and ^{13}C signal linewidth for **3^{iPr}-Ni**.

T_{1e}	$8.961*10^{-9}$ s
T_{2e}	$1.792*10^{-8}$ s
τ_R	$2.205*10^{-7}$ s
r	6.980 Å
η (benzene, 295 K)	0.630 mPa*s

Table S33. Experimental chemical shifts and their corresponding weights used for the fitting procedure of the calculated **3^{iPr}-Ni** NMR signals.

Group	δ_{exp} (ppm)	weight[a]
H_{1a}	0.280	1
H_{1b}	6.620	1
H_2	30.050	1
H_3	-18.605	1
H_5	10.547	1
H_7	22.057	1
H_{11}	1.459	1
C_{1a}	103.22	0.2
C_{1b}	37.47	0.2
C_4	187.23	0.2
C_5	90.15	0.2
C_6	146.53	0.2
C_7	143.26	0.2
C_{10}	28.53	0.2
C_{11}	37.47	0.2

[a] A weight of 0.2 was set for ^{13}C resonances to reduce their influence on the fitting procedure, due to larger chemical shift deviations.

10.2) EPR results

Table S34. Isotropic and anisotropic g-values for complexes **3^{iPr}-Pd** and **3^{iPr}-Ni** computed on B3LYP-GD3(BJ)/SARC-ZORA-TZVP(Pd only)/ZORA-Def2-TZPV(-f) (for **3^{iPr}-Pd**) and B3PW91-GD3(BJ)/ZORA-Def2-TZVP(-f) (for **3^{iPr}-Ni**) level of theory.

compound	g_x	g_y	g_z	g_{iso}
**3^{iPr}-Pd**	2.026630	2.1221191	2.1614308	2.1033933
**3^{iPr}-Ni**	2.0046027	2.2089648	2.3456905	2.1864193

Table S35. Isotropic and anisotropic hyperfine coupling constants (HFC) in MHz for complexes **3^{iPr}-Pd** and **3^{iPr}-Ni** computed on B3LYP-GD3(BJ)/SARC-ZORA-TZVP(Pd only)/ZORA-Def2-TZPV(-f) (for **3^{iPr}-Pd**) and B3PW91-GD3(BJ)/ZORA-Def2-TZVP(-f) (for **3^{iPr}-Ni**) level of theory.

compound	Atom	A_x	A_y	A_z	A_{iso}
**3^{iPr}-Pd**	Pd	-74.6719	-125.1548	-147.4909	-115.7725
	P1	31.7428	36.7404	73.6419	47.3750
	P2	32.0173	37.2651	74.2033	47.8286
	N	44.2019	46.4830	67.7174	52.8008
**3^{iPr}-Ni**	P1	-7.92	-22.0136	-30.3798	-20.1045
	P2	-4.5279	-18.6605	-26.8074	-16.6653
	N	17.5427	17.5704	23.5884	19.5671

10.3) CASSCF results (3^{iPr}-Ni)

Table S36. Configurations for the complete active space used for **3^{iPr}-Ni** (CAS(9,5)).

CASSCF states for 3iPr-Ni (MULT=2 NROOTS=5)	
ROOT 0: E= -3585.0188378184 Eh<br>**0.74211 [3]: 22212**<br>0.25119 [0]: 12222<br>0.00669 [1]: 21222	==<br>NEVPT2 Results<br>==<br>Total Correction: dE = -7.34692696401044<br>---<br>Reference: E0 = -3585.01883781844026<br>---<br>Total E (E0+dE): E = -3592.36576478245070<br>---
ROOT 1: E= -3585.0103389844 Eh<br>**0.98859 [1]: 21222**<br>0.01012 [0]: 12222	==<br>NEVPT2 Results<br>==<br>Total Correction: dE = -7.34689957945966<br>---<br>Reference: E0 = -3585.01033898443075<br>---<br>Total E (E0+dE): E = -3592.35723856389041<br>---
ROOT 2: E= -3584.9983272988 Eh<br>**0.73635 [0]: 12222**<br>0.25566 [3]: 22212<br>0.00469 [1]: 21222<br>0.00317 [4]: 22221	==<br>NEVPT2 Results<br>==<br>Total Correction: dE = -7.34362837150320<br>---<br>Reference: E0 = -3584.99832729877744<br>---<br>Total E (E0+dE): E = -3592.34195567028064<br>---
ROOT 3: E= -3584.9970486760 Eh<br>**0.98225 [4]: 22221**<br>0.01447 [2]: 22122	==<br>NEVPT2 Results<br>==<br>Total Correction: dE = -7.34108257638445<br>---<br>Reference: E0 = -3584.99704867600303<br>---<br>Total E (E0+dE): E = -3592.33813125238748<br>---
ROOT 4: E= -3584.9890517113 Eh<br>**0.98540 [2]: 22122**<br>0.01458 [4]: 22221	==<br>NEVPT2 Results<br>==<br>Total Correction: dE = -7.33935275764679<br>---<br>Reference: E0 = -3584.98905171130855<br>---<br>Total E (E0+dE): E = -3592.32840446895534<br>---

Table S37. SC-NEVPT2 corrected AILFT (one electron eigenfunctions) results for **3^{iPr}-Ni** (CAS(9,5)). SOC constant ζ_D = 570.10 cm^{-1}.

Orbital	Energy (eV)	Energy (cm^{-1})	dz^2	dxz	dyz	dx^2-y^2	dxy
1	0.000	0.0	-0.003488	0.002263	-0.992674	-0.002235	0.120731
2	0.265	2134.8	-0.048281	0.004700	-0.120289	-0.030484	-0.991084
3	0.369	2974.1	-0.858106	0.068514	0.011161	-0.505632	0.056325
4	0.785	6328.4	0.100601	0.994277	0.002074	-0.035883	0.000666
5	1.017	8199.6	0.501189	-0.081801	0.000195	-0.861461	0.001670

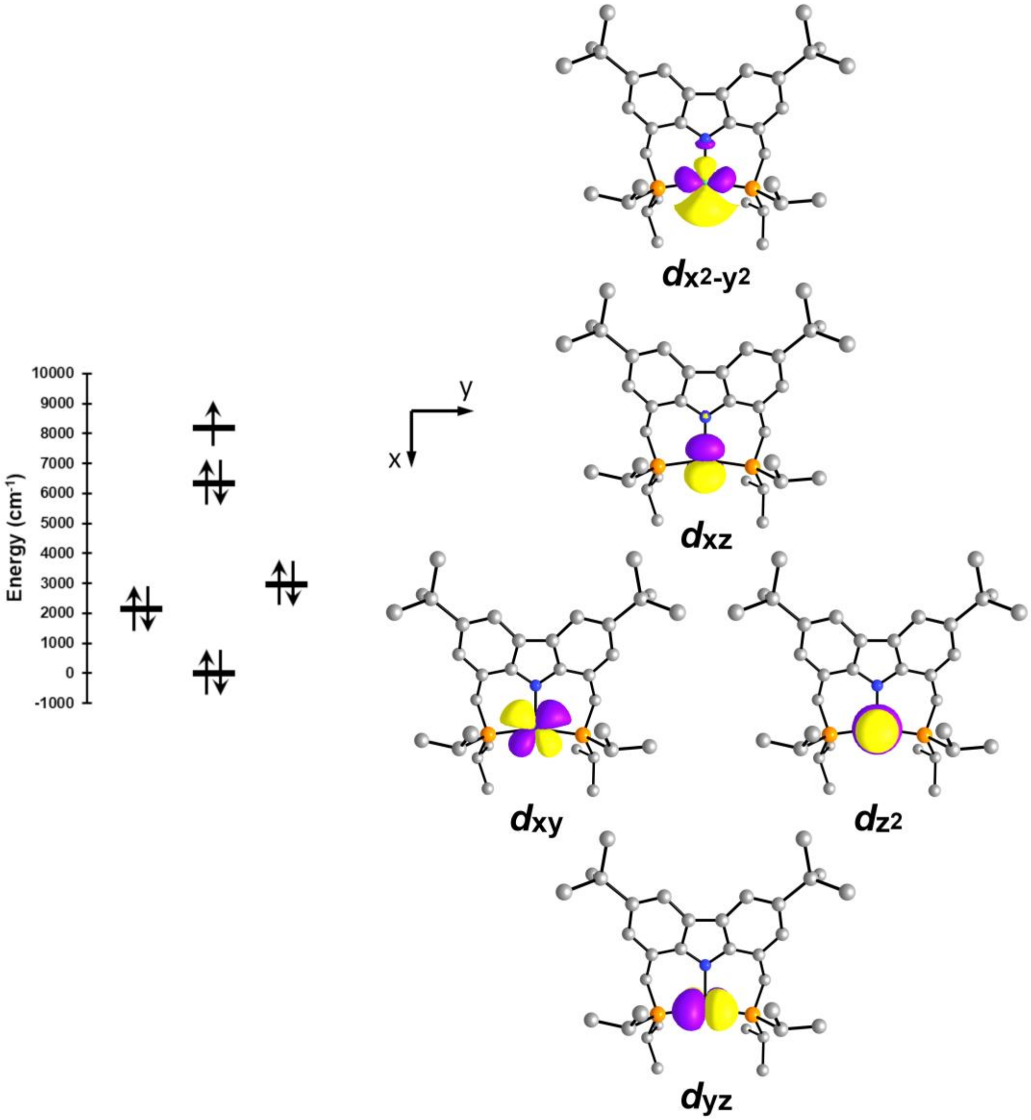

Figure S174. *Ab initio* ligand field theory (AILFT) computed splitting of the d-orbital energies for **3^{iPr}-Ni** and corresponding natural orbitals within the active space (derived from a SC-NEVPT2 corrected CAS(9,5), isovalue for printing = 0.03).

Table S38. Electronic g-values for **3^{iPr}-Ni** from effective Hamiltonian.

g_x	g_y	g_z	g_{iso}
1.820	2.152	3.474	2.482

Table S39. Magnetic susceptibility tensor (cm^3 K mol^{-1}) for **3^{iPr}-Ni** at 295 K (SC-NEVPT2 corrected CAS(9,5)).

0.3242	0.0007	-0.0120
0.0007	1.2922	-0.0006
-0.0120	-0.0006	0.5156

Table S40. SC-NEVPT2/SOC corrected CASSCF absorption spectrum of **3^{iPr}-Ni**.

States	Energy (cm^{-1})	Wavelength (nm)	Oscillator Strength
0 1	0.0	0.0	0.000000000
1 2	2076.0	4817.0	0.000016291
1 3	2076.0	4817.0	0.000009226
0 2	2076.0	4817.0	0.000009226
0 3	2076.0	4817.0	0.000016291
1 4	5265.9	1899.0	0.000097122
1 5	5265.9	1899.0	0.000013947
0 4	5265.9	1899.0	0.000013947
0 5	5265.9	1899.0	0.000097122
1 6	6280.2	1592.3	0.000082679
1 7	6280.2	1592.3	0.000087005
0 6	6280.2	1592.3	0.000087005
0 7	6280.2	1592.3	0.000082679
1 8	8518.5	1173.9	0.000005227
1 9	8518.5	1173.9	0.000005800
0 8	8518.5	1173.9	0.000005800
0 9	8518.5	1173.9	0.000005227

10.4) UV/Vis

The first six TD-DFT-computed excited states in **3^{iPr}-Ni** (Table S41) were analyzed using Multiwfn. Table S42 lists the spatial distributions for hole (excited from) and electron (excited to) densities, the Sr fuctions (hole-electron overlap) and transition densities. The first four transitions are primarily d→d excitations (see table S1). Their Sr indices (~0.63 a.u., theoretical maximum = 1.0) indicate substantial spatial overlap between hole and electron densities (Table S43). Excitations 5 and 6 originate from the ligand, exhibiting ligand-to-metal charge transfer (LMCT, state 5, Sr ≈ 0.38) and ligand-to-ligand excitation (LL, state 6).

The computed excitation spectra of **4^{iPr}-Pd**, **4^{Et}-Pd** and **4^{Et}-Ni** were illustrated against their respective experimental spectra (Figure S175, S176, S178, S179) together with their absorption bands with >80% M–M-σ* (Figure S176, S178, S181) contribution to the excited-electron distribution (electron accepting orbitals; Table S44-S46).

Table S41. Listing of the first 30 excited states of **3^{iPr}-Ni** (CPCM(pentane)/B3PW91-GD3(BJ)/ZORA-Def2-TZVP(-f) level of theory).

Excited State	Wavelength (nm)	Energy (cm^{-1})	Oscillator Strength
1	1896.8	5272	0.00014
2	1519.4	6582	0.00098
3	1066.7	9375	0.00064
4	1031.0	9699	0.00041
5	447.2	22361	0.00350
6	429.4	23288	0.00001
7	377.6	26483	0.00009
8	376.3	26575	0.00673
9	367.0	27248	0.00022
10	361.6	27655	0.00379
11	355.9	28098	0.00990
12	340.5	29369	0.13492
13	338.8	29516	0.00050
14	319.5	31299	0.00894
15	317.2	31526	0.01670
16	311.6	32092	0.03728
17	302.6	33047	0.02956
18	300.9	33234	0.00077
19	298.0	33557	0.00006
20	295.1	33887	0.00543
21	294.8	33921	0.00070
22	293.2	34106	0.00076
23	290.7	34400	0.02708
24	285.2	35063	0.00128
25	284.9	35100	0.04032
26	283.7	35249	0.00635
27	282.3	35423	0.00127
28	279.2	35817	0.02635
29	278.0	35971	0.00089
30	274.9	36377	0.03046

Table S42. Listing of hole and electron distributions, Sr functions and transition densities of the first six excited states of **3[iPr]-Ni** (isovalue for printing: 0.001).

State	hole distribution	electron distribution	Sr function	transition density
1	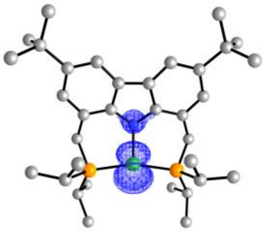 d_{xz}	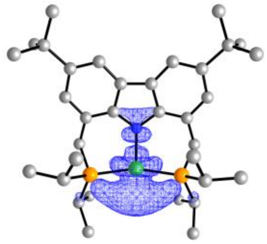 $d_{x^2-y^2}$	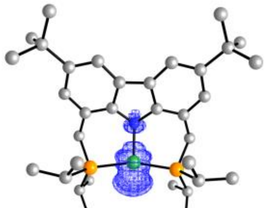	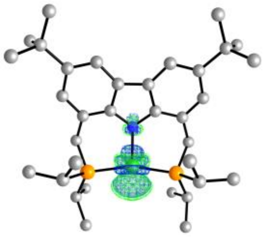
2	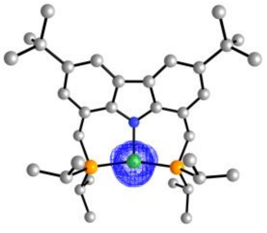 d_{z^2}	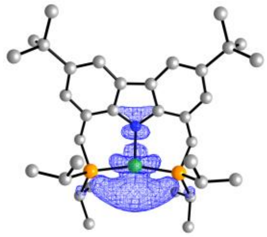 $d_{x^2-y^2}$	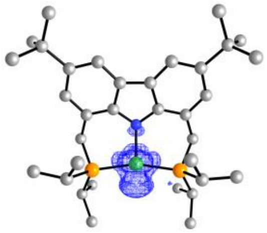	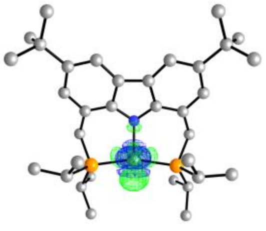
3	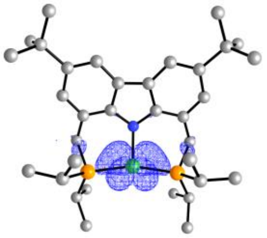 d_{xy}/d_{yz}	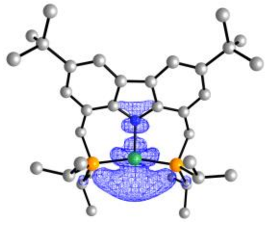 $d_{x^2-y^2}$	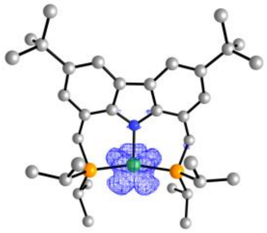	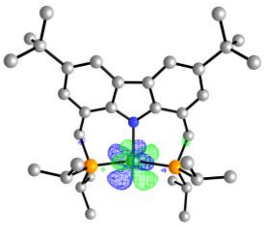
4	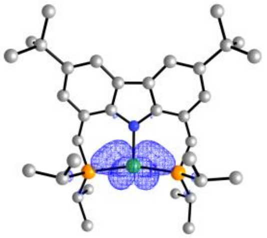 d_{xy}/d_{yz}	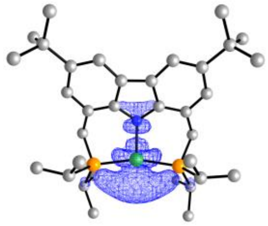 $d_{x^2-y^2}$	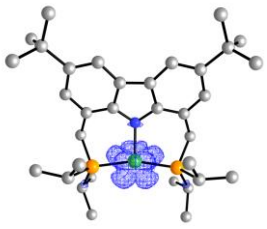	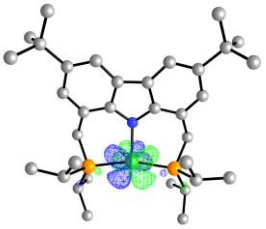
5	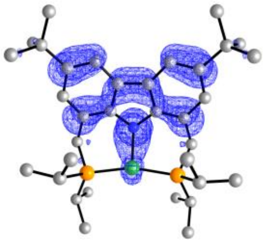 π	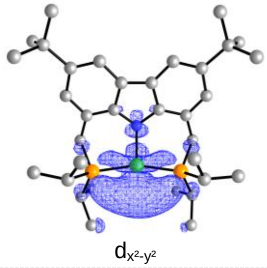 $d_{x^2-y^2}$	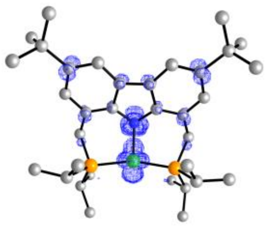	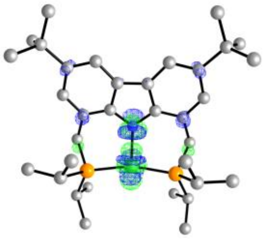
6	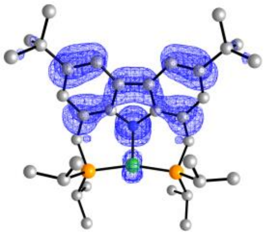 π	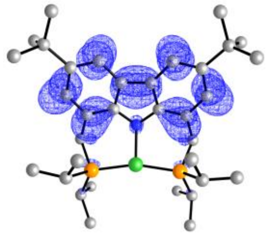 π	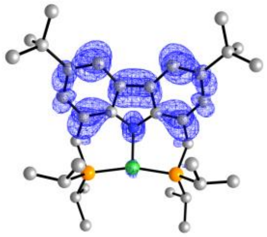	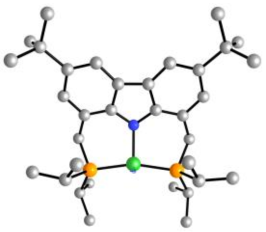

Table S43. Integrals of the hole, electron and transition density distributions, Sr indices and coulomb attractive energies (exciton binding energies) of the first six excited states of **3^{iPr}-Ni**.

State	Integral of hole	Integral of electron	Integral of transition density	Sr index (a.u.)	Coulomb attractive energy (eV)
1	1.013	0.988	-0.009	0.628	10.31
2	0.993	0.988	-0.002	0.629	10.08
3	0.997	0.988	-0.010	0.621	10.28
4	1.018	0.988	0.003	0.634	10.24
5	1.001	0.991	0.003	0.381	3.97
6	1.000	1.000	<0.001	0.707	4.78

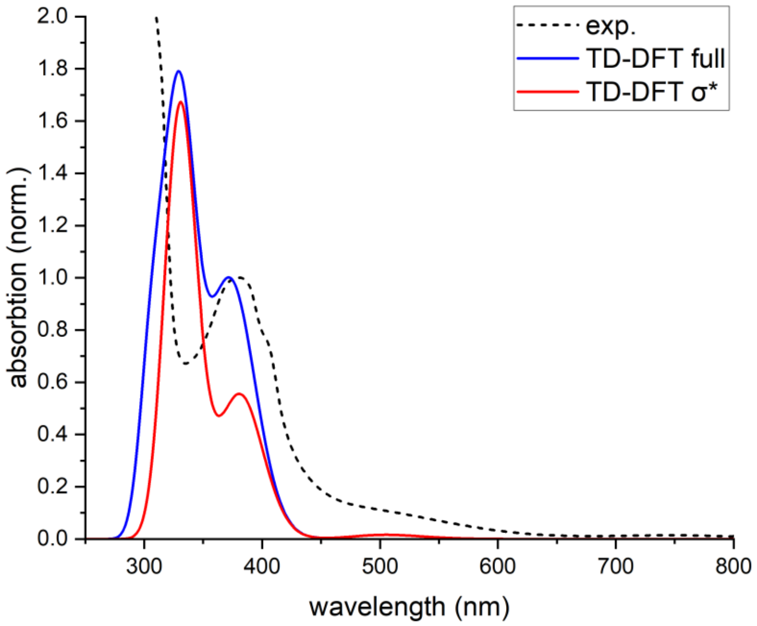

Figure S175. Experimental UV/Vis absorption spectrum of **4^{iPr}-Pd** in THF at room temperature illustrated in relation to the TD-DFT computed absorption spectrum (blue line). The absorption maxima at 380 nm (exp.) and 373 nm (TD-DFT) were normalized, since no experimental extinction coefficients were determinable. The spectrum considering only M/L→M–M-σ* excitations is depicted in red with absorption intensities referenced against the full TD-DFT computed spectrum. Theoretical spectra were depicted with a peak half-width at half height value of 0.2 eV.

Table S44. Listing of excited states of **4^{iPr}-Pd** (CPCM(THF)/B3LYP-GD3(BJ)/Def2-TZVP level of theory). Excitations with an oscillator strength of zero were omitted.

Excited State	Energy (cm-1)	Wavelength (nm)	Oscillator Strength	M–M-σ*-composition of excited-electron distribution (%)
4	19735.9	506.7	0.00406	99
5	19957.1	501.1	0.00150	99
12	25425.3	393.3	0.02266	98
13	26037.3	384.1	0.13481	95
16	26895	371.8	0.08910	4
17	26975.7	370.7	0.01549	99
18	27218.3	367.4	0.02834	95

20	27504.4	363.6	0.08922	6
21	27640.6	361.8	0.00787	99
24	28159.5	355.1	0.01064	99
27	28967.5	345.2	0.00002	0
30	29568.3	338.2	0.00074	0
32	30058.4	332.7	0.48571	88
33	31250.1	320.0	0.13319	94
38	32252.1	310.1	0.00927	0
39	32420.0	308.5	0.05744	2
40	32584.3	306.9	0.07631	14
41	32670.4	306.1	0.08423	36
42	32674.5	306.0	0.00025	46
44	32731.6	305.5	0.01880	0
45	32901.4	303.9	0.00078	0

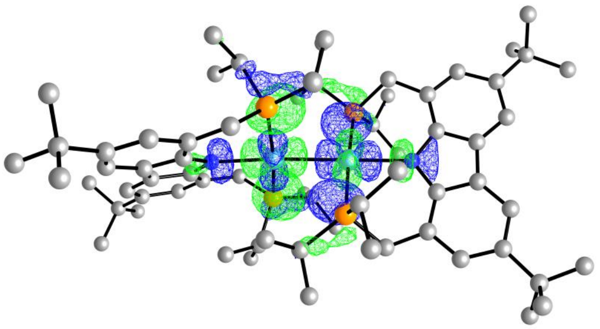

Figure S176. Representation of the M–M-σ* molecular orbital in **4^{iPr}-Pd** (isovalue 0.03).

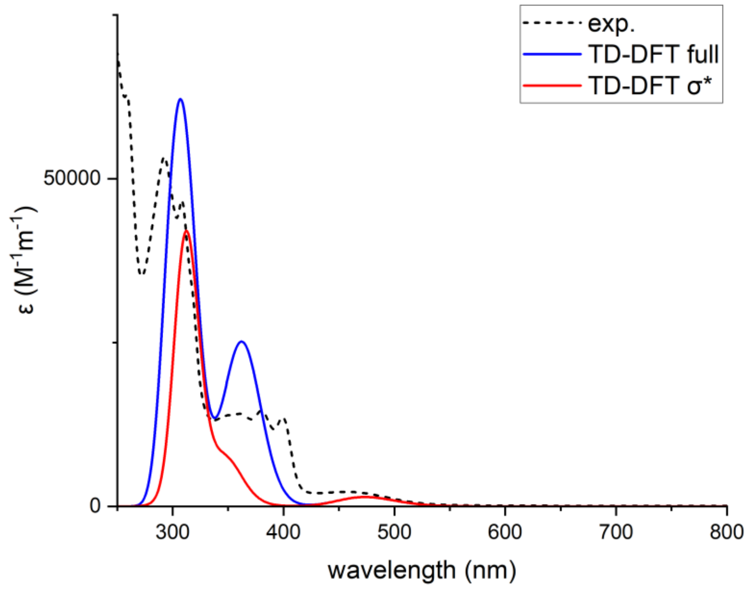

Figure S177. Experimental UV/Vis absorption spectrum of **4^{Et}-Pd** in THF at room temperature illustrated in relation to the TD-DFT computed absorption spectrum (blue line). The spectrum considering only M/L→M–M-σ* excitations is depicted in red. Theoretical spectra were depicted with a peak half-width at half height value of 0.2 eV and their absorption intensities referenced against the experimental extinction coefficients.

Table S45. Listing of excited states of **4^{Et}-Pd** (CPCM(THF)/B3LYP-GD3(BJ)/Def2-TZVP level of theory). Excitations with an oscillator strength of zero were omitted.

Excited State	Energy (cm-1)	Wavelength (nm)	Oscillator Strength	M–M-σ*-composition of excited-electron distribution (%)
3	21044.7	475.2	0.01461	99
5	21461.4	466.0	0.00320	99
11	26561.4	376.5	0.00179	98
15	27359.0	365.5	0.24513	3
16	27459.4	364.2	0.01297	0
17	28274.9	353.7	0.00129	98
18	28499.0	350.9	0.04992	96
19	28828.9	346.9	0.01864	98
23	29218.6	342.2	0.00099	99
25	29317.1	341.1	0.02159	97
27	29362.3	340.6	0.00290	0
29	29484.4	339.2	0.00076	0
32	32021.8	312.3	0.51712	83
35	32767.4	305.2	0.02842	59
36	32785.2	305.0	0.00712	38
37	32825.4	304.6	0.00747	0
39	33487.0	298.6	0.35309	4
41	33565.4	297.9	0.00412	0
42	33619.2	297.4	0.00100	0
43	33824.6	295.6	0.00818	0
50	34596.8	289.0	0.00581	93

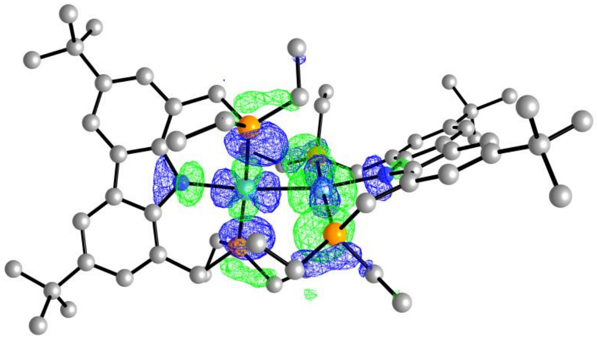

Figure S178. Representation of the M–M-σ* molecular orbital in **4^{Et}-Pd** (isovalue 0.03).

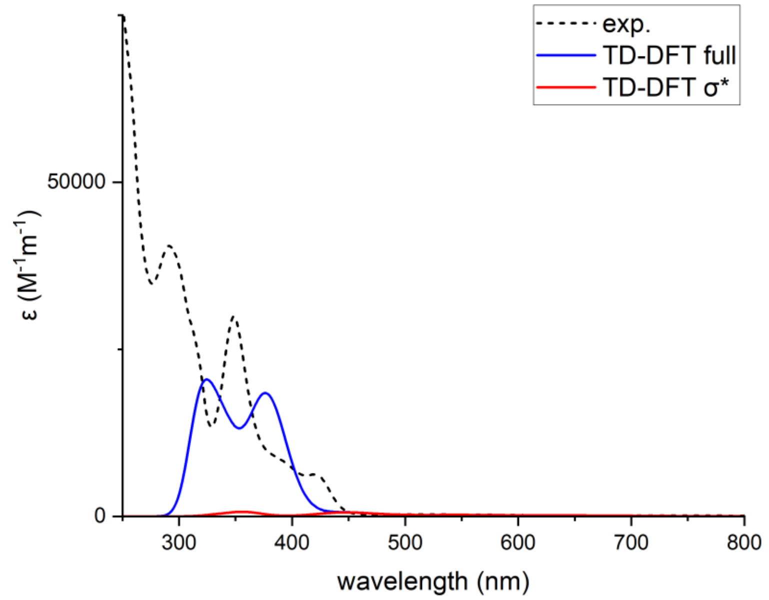

Figure S179. Experimental UV/Vis absorption spectrum of **4^{Et}-Ni** in THF at room temperature illustrated in relation to the TD-DFT computed absorption spectrum (blue line). The spectrum considering only M/L→M–M-σ* excitations is depicted in red. Theoretical spectra were depicted with a peak half-width at half height value of 0.2 eV and their absorption intensities referenced against the experimental extinction coefficients.

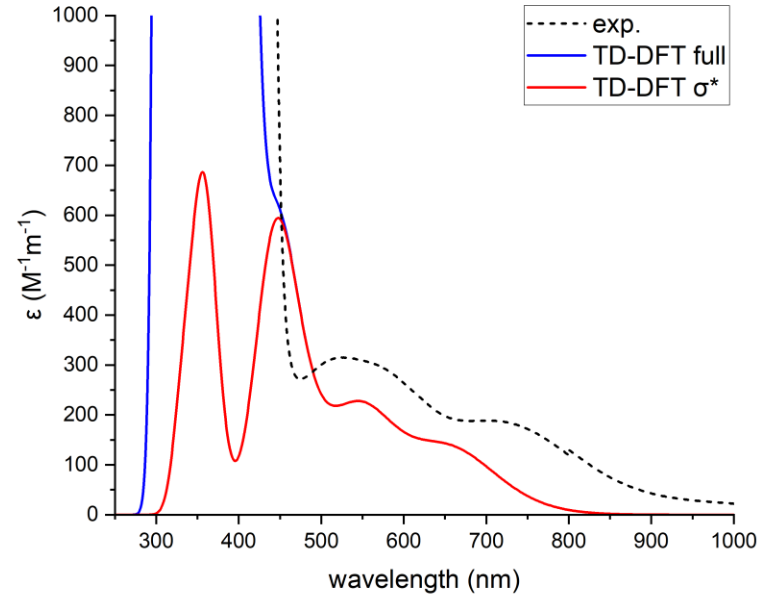

Figure S180. Intensified version of Figure S179.

Table S46. Listing of excited states of **4^{Et}-Ni** (CPCM(THF)/B3LYP-GD3(BJ)/Def2-TZVP level of theory). Excitations with an oscillator strength of zero were omitted.

Excited State	Energy (cm-1)	Wavelength (nm)	Oscillator Strength	M–M-σ*-composition of excited-electron distribution (%)
6	14880.2	672.0	0.00068	97
8	15292.5	653.9	0.00083	97
9	17264.9	579.2	0.00105	97

11	18478.8	541.2	0.00186	97
14	20469.3	488.5	0.00140	96
15	22119.2	452.1	0.00320	96
18	22466.6	445.1	0.00313	96
19	23661.5	422.6	0.00141	96
21	26441.3	378.2	0.20778	5
24	26589.3	376.1	0.00951	0
26	27781.3	360.0	0.00382	91
27	27955.2	357.7	0.00392	93
29	28129.5	355.5	0.00009	3
31	28393.4	352.2	0.00088	4
34	29283.0	341.5	0.12627	57
36	29830.2	335.2	0.00060	100
38	29923.1	334.2	0.00261	99
42	30459.1	328.3	0.00330	0
44	30646.1	326.3	0.00059	0
46	30990.4	322.7	0.00145	0
48	31214.9	320.4	0.16977	4
49	31415.9	318.3	0.03891	0

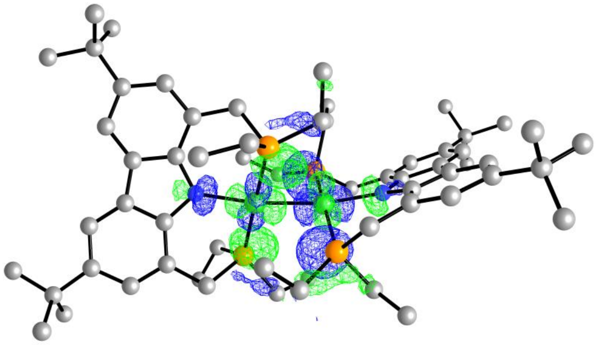

Figure S181. Representation of the M–M-σ* molecular orbital in **4^{Et}-Ni** (isovalue 0.03).

10.5) Analysis of experimentally found conformations of 4^{iPr}-Pd

Table S47. Relative Gibbs free energies for two experimentally observed conformers of **4^{iPr}-Pd** calculated at the CPCM(toluene)/ r^{2}SCAN-3c/Def2-mTZVP level of theory.

	$\Delta G_{298\ \mathrm{K}}$ (kcal/mol)	Conformer Structure
**4^{iPr}-Pd-conformer1**	0.0	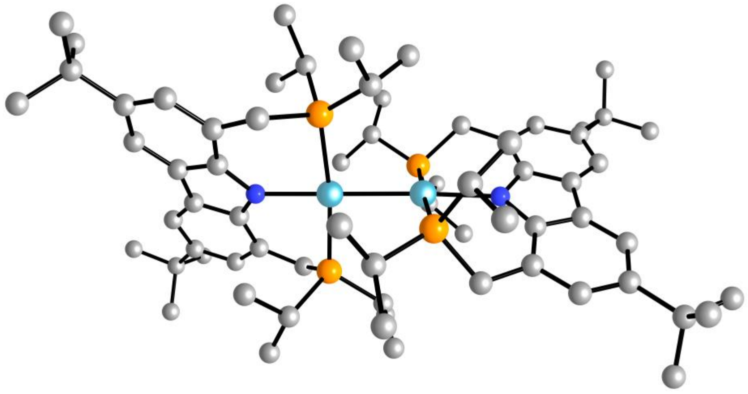
**4^{iPr}-Pd-conformer2**	3.7	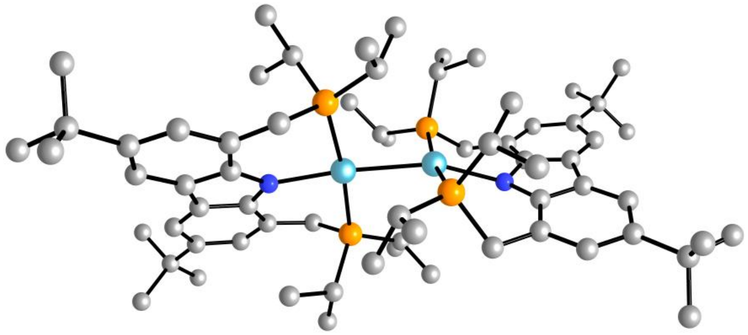

10.6) Modeling Results for the Dissociation of Homodinuclear Dimers

The thermodynamic parameters *bond dissociation enthalpy* (BDE, ΔH), *bond dissociation free energy* (BDFE, ΔG) and the associated *entropy change* (ΔS) were modeled for the dissociation of **4^{iPr}-Pd**, **4^{Et}-Pd**, and **4^{Et}-Ni** into their monomers. For the isopropyl derivative **4^{iPr}-Pd**, relative values were determined using the energetically most favorable ground state monomer structure (earlier identified, *vide supra*) and the dimer with its experimentally optimal conformer geometry (**4^{iPr}-Pd-conformer1**). To identify the lowest-energy geometries for the **4^{Et}-Ni** dimer as well as the monomers **3^{Et}-Pd** and **3^{Et}-Ni**, conformational samplings were performed analogous to the prior method for the **3^{Et}-Pd** monomer using GOAT. The energetically lowest conformers (five for **3^{Et}-Pd**, four for **3^{Et}-Ni**) were subsequently reoptimized with r^{2}SCAN-3c and comparatively evaluated. To reduce computational time, a full re-evaluation was omitted for the **4^{Et}-Ni** dimer. Instead, the lowest-energy XTB2 structure from the GOAT screening served as its representative low-lying conformer and was reoptimized using r^{2}scan-3c. For **4^{Et}-Pd** the same geometry as for **4^{Et}-Ni** was used. Comparison of the monomer conformer energies revealed that variations in ethyl substituent orientations on the ligand have negligible impact on the relative Gibbs energies. Computation of BDE, BDFE, and ΔS values relied exclusively on the dimer structures and their corresponding monomers with minimal structural energy. Note: transferring calculated free energies from the gas phase into the solution usually requires a correction of 1.89 kcal mol^{-1} that needs to be considered when the molecularity changes during a reaction. This correction however was omitted, as no high-level theory was used to recompute the electronic energies of the complexes and thus the error of the used DFT method already lies within that energy range.

Table S48. Relative Gibbs free energies of the five most stable conformers for **3^{Et}-Pd** found using GOAT, reoptimized at the CPCM(toluene)/ r^{2}SCAN-3c/Def2-mTZVP level of theory.

Conformer Entry	Relative Gibbs free Energy ΔG (kcal/mol)
1	0.04
2	0.09
3	0.12
4	0.21
5	0.00

Table S49. Relative Gibbs free energies of the four most stable conformers for **3^{Et}-Ni** found using GOAT, reoptimized at the CPCM(toluene)/ r^{2}SCAN-3c/Def2-mTZVP level of theory.

Conformer Entry	Relative Gibbs free Energy ΔG (kcal/mol)
1	0.00
2	0.10
3	0.18
4	0.05

Table S50. Gibbs free energy, enthalpy and entropy values computed at the CPCM(toluene)/r^{2}SCAN-3c/Def2-mTZVP level of theory for the dimers **4^{iPr}-Pd**, **4^{Et}-Pd** and **4^{Et}-Ni** and two isolated monomers of **3^{iPr}-Pd**, **3^{Et}-Pd** and **3^{Et}-Ni** together with their relative values (Δ).

	G (Eh)	H (Eh)	S (Eh)	ΔG (kcal/mol)	ΔH (kcal/mol)	ΔS (cal/molK)
**4^{iPr}-Pd-conformer1**	-4385.00041	-4384.80580	0.00065306	0.0	0.0	0.0
2x **3^{iPr}-Pd**	-4384.98976	-4384.76193	0.00076453	6.7	27.5	70.0
**4^{Et}-Pd**	-4070.86402	-4070.68575	0.00059822	0.0	0.0	0.0
2x **3^{Et}-Pd**	-4070.81714	-4070.60728	0.00070422	29.4	49.2	66.5
**4^{Et}-Ni**	-6831.57904	-6831.40396	0.00058752	0.0	0.0	0.0
2x **3^{Et}-Ni**	-6831.56323	-6831.35319	0.00070482	9.9	31.9	73.6

10.7) Isomerization Mechanism of 4^{iPr}-Pd

The isomerization mechanism of **4^{iPr}-Pd** was modeled using a truncated structure wherein the *tert*-butyl groups on the carbazole units were replaced with hydrogen atoms (see Table S51). A conformational screening performed with GOAT established the starting geometry, which is nearly identical to the energetically favored X-ray structure conformer **4^{iPr}-Pd-conformer1** exhibiting a *twist* conformation in both ligands. Isomerization into a *seat-twist* conformation (**4''-Pd**) occurs *via* flipping of one -CH_2- sidearm and proceeds over two transition states (**TS1** and **TS2**). **TS1** corresponds directly to the arm flip itself, leading to intermediate **INT1**. However, a second transition state (**TS2**) is needed to obtain the more stable conformer **INT3** and involves reorientation of isopropyl groups, yielding the less strained conformation. This transition state corresponds to the experimentally observed coalescence of the two -CH_2- proton resonances in the ^{1}H NMR spectrum. A backwards IRC scan initiated from **TS2** resulted in an alternative intermediate **INT2** featuring slightly different isopropyl group orientations compared to **INT1**, without significant change in molecular strain. A NEB calculation confirmed that this minor reorientation involves an energy barrier of less than 2 kcal mol^{-1}. For the isomerization into the *seat-seat* conformation (**4''-Pd**), a third transition state (**TS3**) was identified for the flip of the second -CH2- sidearm, starting from **INT3**. With this transition state, a backwards IRC scan towards **INT3** from **TS3** led to isomer 4''-Pd, which is slightly more stable than **INT3** (obtained directly *via* **TS2**) due again to minor isopropyl reorientation with a barrier less than 2 kcal mol^{-1}. A forwards IRC scan led to intermediate **INT4**. A final transition state (**TS4**) for isopropyl group reorientation then releases molecular strain, leading to the third isomer **4''-Pd**.

Note: Comparison of **INT1** vs. **INT2**, or **INT3** vs. **4''-Pd** demonstrate that slight variations in isopropyl orientations result in energy differences of a few kcal mol^{-1}. Consequently, the identified isomerization transition states might not represent the theoretical absolute energetic minima. Potentially lower-energy pathways could exist involving slightly higher-lying intermediates connected by reorientation transition states also lying below the insomerization barriers. Thus, the computed isomerization transition state energies likely represent upper bounds and may be overestimated. However, comprehensive conformational sampling for transition states is computationally expensive given the molecule's size and complexity.

Table S51. Relative Gibbs free energies for ground and transition state structures of truncated conformers and their interconversion of **4^{iPr}-Pd** at the CPCM(toluene)/r^{2}SCAN-3c/Def2-mTZVP level of theory.

	$\Delta G_{298\ \mathrm{K}}$ (kcal mol^{-1})	Structure
**4'-Pd**	0.0	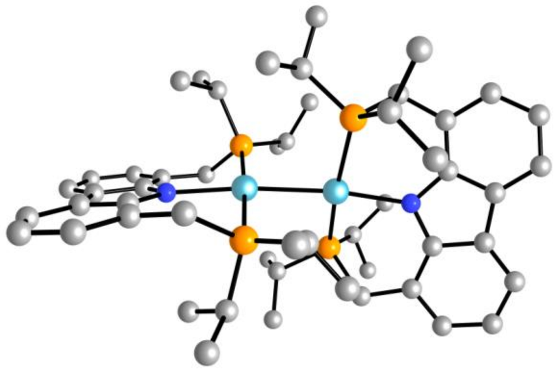
**TS1**	6.4	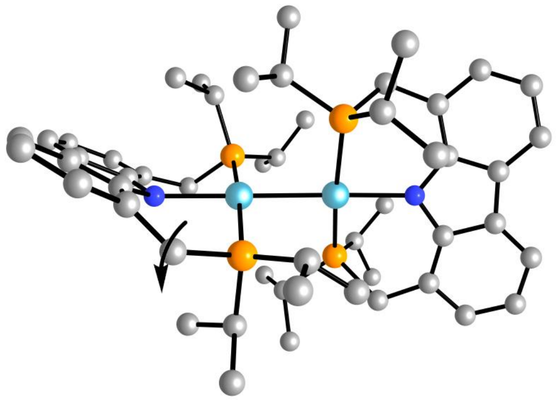

**INT1**	5.8	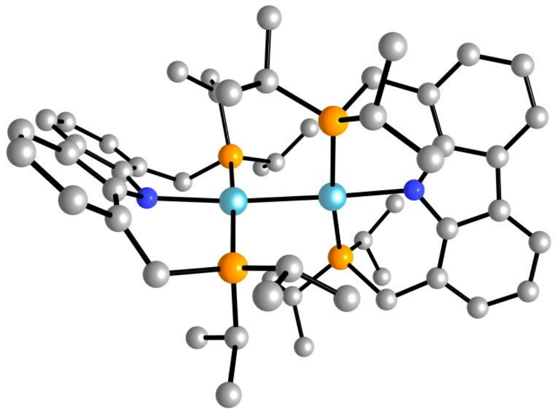
**INT2**	6.9	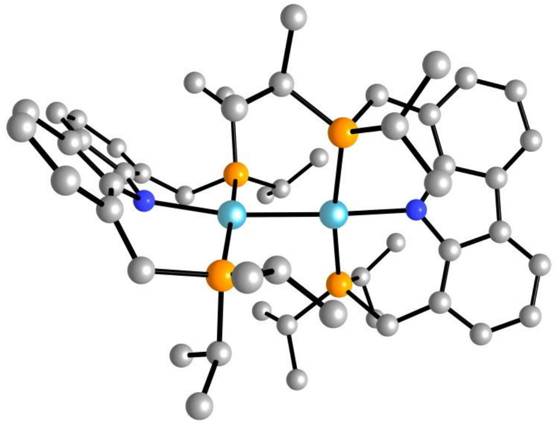
**TS2**	17.0	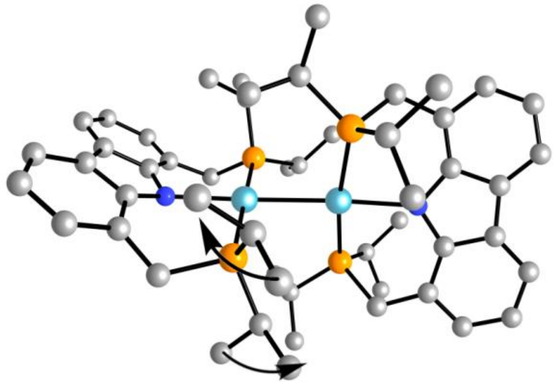
**INT3**	2.2	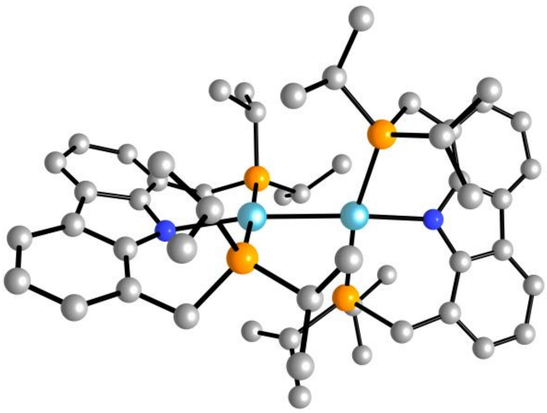

4’’-Pd 0.6

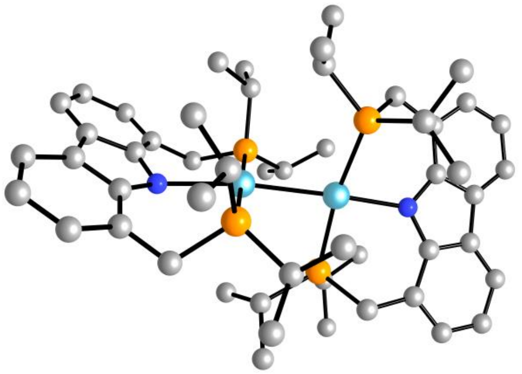

TS3 10.1

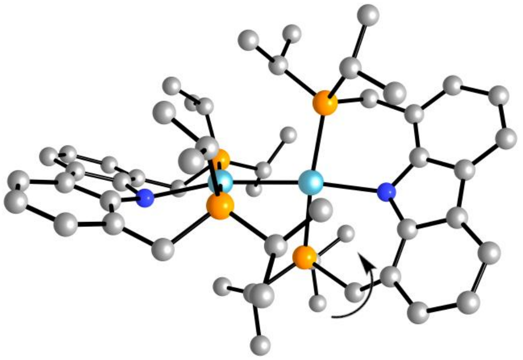

INT4 9.0

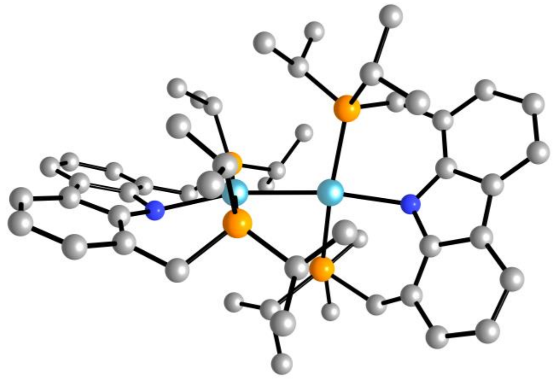

TS4 19.9

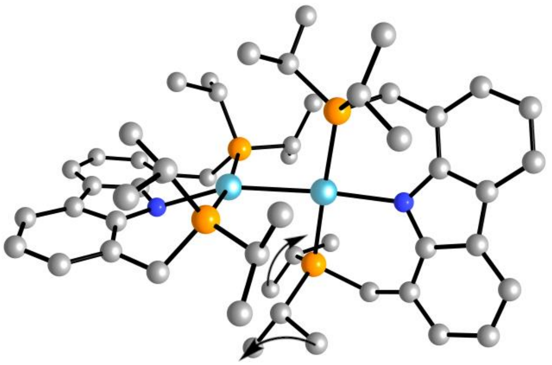

**4'''-Pd**	3.2	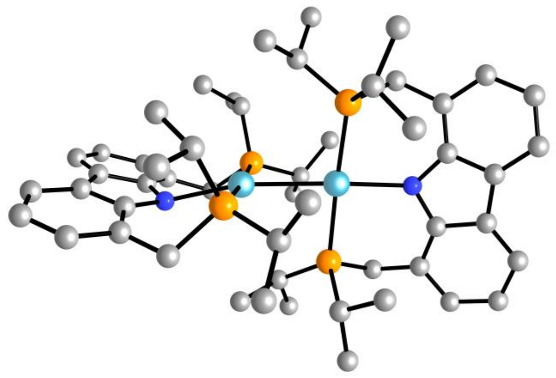

10.8) Monomerizations starting at 4'-Pd, 4''-Pd, 4'''-Pd

The observed dissociation of the **4^{iPr}-Pd** dimer can occur in all three identified low-energy conformers (local minima). However, the binding strength between monomer moieties varies depending on their conformation, resulting in different dissociation propensities. The dissociation process was modeled separately for each conformer (**4'-Pd**, **4''-Pd**, and **4'''-Pd**). Broken-symmetry (1,1) PES scans revealed a continuous increase in electronic energy (SCF) upon increasing the Pd–Pd distance for all conformers, yet without characteristic maxima indicative of transition states. Furthermore, broken symmetry optimizations starting from separated monomers with a Pd–Pd distance of 5 Å consistently resulted in recombination of the monomers into their respective dimers. This suggests that the dissociation itself proceeds without a transition state.

The monomer geometries of **3'-Pd** and **3''-Pd** (Table S52) were optimized directly from the *twist* and *seat* conformations within their respective halves in **4'-Pd** and **4'''-Pd**. Assuming dissociation initiates from the dimeric conformers without significant conformational changes (e.g., isopropyl reorientation), structures **3'-Pd** and **3''-Pd** then represent the initially formed monomer isomers. Relative to the most stable dimer conformer **4'-Pd** (ΔG = 0 kcal mol^{-1}), the direct dissociation requires 21.8 kcal mol^{-1} for **4'-Pd**, 18.9 kcal mol^{-1} for **4''-Pd**, and 16.0 kcal mol^{-1} for **4'''-Pd**. Although **4'''-Pd** enables the energetically most favorable dissociation, isomerization barriers from **4'-Pd** to **4'''-Pd** must first be surmounted.

After dissociation, the monomers isomerize into their most stable conformation. This state was earlier identified for **3^{iPr}-Pd** in chapter 10.1 and adapted for the truncated structure **3-Pd-seat**. Starting from this geometry, a transition state (**3-Pd-TS**) connecting to the *twist* conformer (**3-Pd-twist**) was located. However, the resulting **3-Pd-twist** exhibits differing isopropyl orientations compared to **3''-Pd**. As the isomerization of the monomers occurs far from the dissociation coordinate *via* vibrational relaxation events and no closed system applies experimentally, the reorientation and isomerization (*twist* to *seat*) energy does not contribute to the overall (direct) dissociation energy.

Table S52. Relative Gibbs free energies for ground and transition state structures of truncated conformers and their interconversion of **3^{iPr}-Pd** at the CPCM(toluene)/r^{2}SCAN-3c/Def2-mTZVP level of theory.

	$\Delta G_{298\ K}$ (kcal mol^{-1})	Structure
**3'-Pd**	7.3	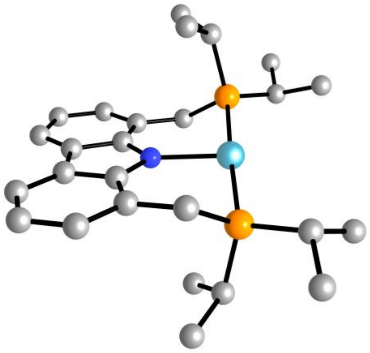
**3''-Pd**	4.3	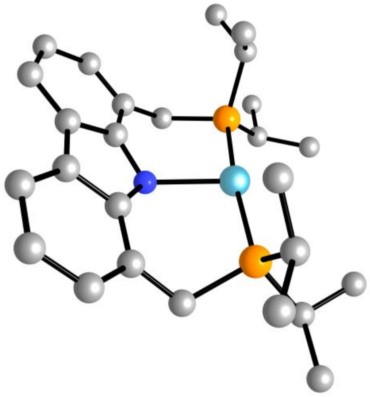

**3-Pd-seat**	0.0	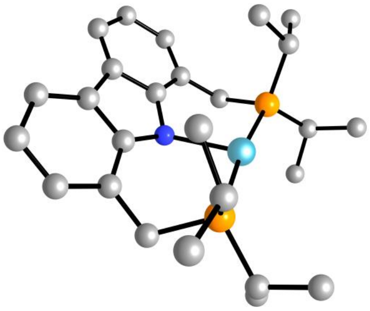
**3-Pd-TS**	6.0	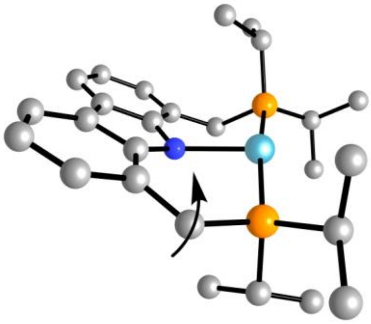
**3-Pd-twist**	4.1	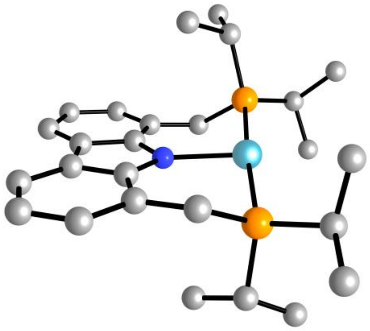

10.9) Triplet Structures

For the **4^{Et}-Pd** and **4^{Et}-Ni** dimers, two triplet-state structures each were investigated (Table S53). Conformational sampling of the Ni compounds using GOAT with constrained N and Ni coordinates led to the energetically preferred conformations. Re-evaluation of global minima with r^{2}SCAN-3c and conformational screening for the Pd derivatives were omitted to reduce computational time. Instead, the lowest-energy XTB2 structures of **4^{Et}-Ni-M3** and **4^{Et}-Ni-M3'** were directly reoptimized using CPCM(toluene)/r^{2}SCAN-3c/Def2-mTZVP. The resulting geometries served as starting points for the Pd congeners, which were reoptimized using the identical method. Relative energies were referenced to the singlet ground-state structures of **4^{Et}-Ni** and **4^{Et}-Pd** (vide supra). (Starting with the geometry of **4^{Et}-Ni-M3**, a singlet state of similar structure but higher relative energy was optimized. Starting with the geometry of **4^{Et}-Ni**, a triplet state of similar structure but higher relative energy was optimized.)

The bonding situation in **4^{Et}-Ni-M3** was further analyzed *via* QTAIM topology analysis, based on a single-point calculation (CPCM(toluene)/TPSSh-GD3(BJ)/Def2-TZVP). A bond critical point (BCP, (3, -1)) between the metal centers revealed an electron density $\rho(r) = 0.048\ a.u.$, a Laplacian of the electron density $\nabla^2\rho(r) = 0.109\ a.u.$ and ellipticity ε = 0.043 at this point.

The low ρ-value indicates a weak non-covalent interaction, corroborated by the positive $\nabla^2\rho$ (electron density depletion at BCP). The negligible ellipticity suggest radially symmetric σ-type bonding. Analysis of the singly occupied quasi-restricted orbitals (Figure S182) confirms two metal-centered unpaired electrons, with one residing in a σ-bonding orbital ($d_{x^2-y^2}$ + $d_{x^2-y^2}$), the other in a σ*-antibonding orbital ($d_{x^2-y^2}$ - $d_{x^2-y^2}$).

Table S53. Relative Gibbs free energies for ground state triplet structures of **4^{Et}-Pd** and **4^{Et}-Ni** at the CPCM(toluene)/r^{2}SCAN-3c/Def2-mTZVP level of theory.

	$\Delta G_{298\ K}$ (kcal mol^{-1})	Structure
**4^{Et}-Ni-M3**	1.5	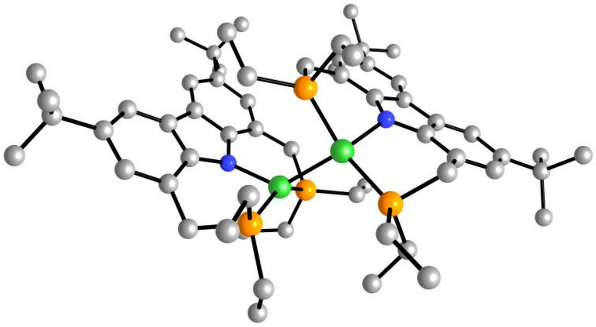
**4^{Et}-Ni-M3'**	19.2	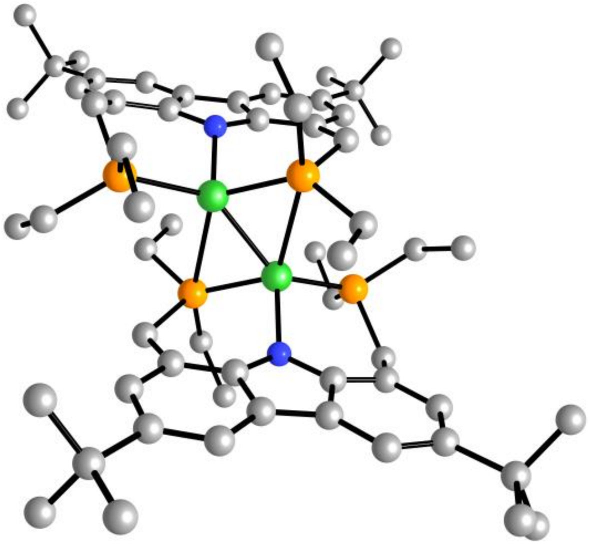

**4^{Et}-Pd-M3**	26.4	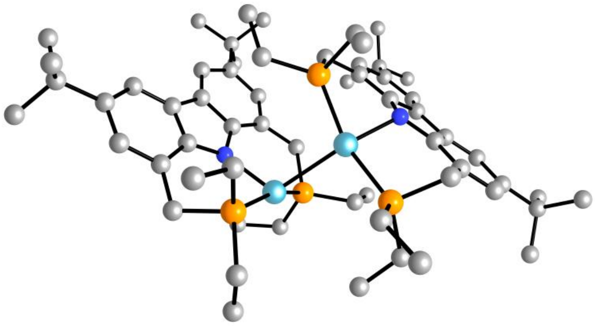
**4^{Et}-Pd-M3'**	45.2	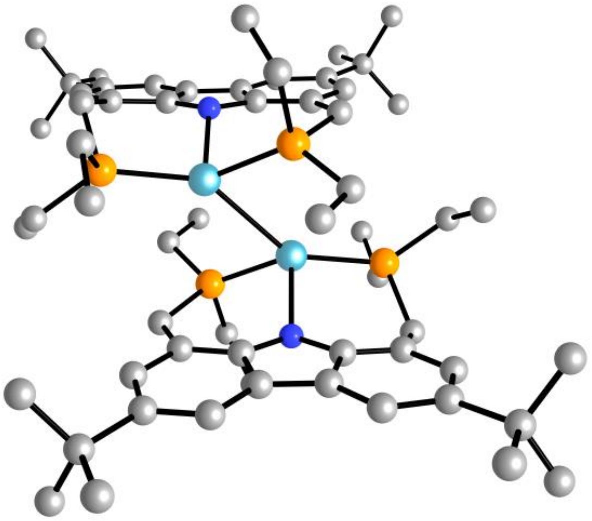

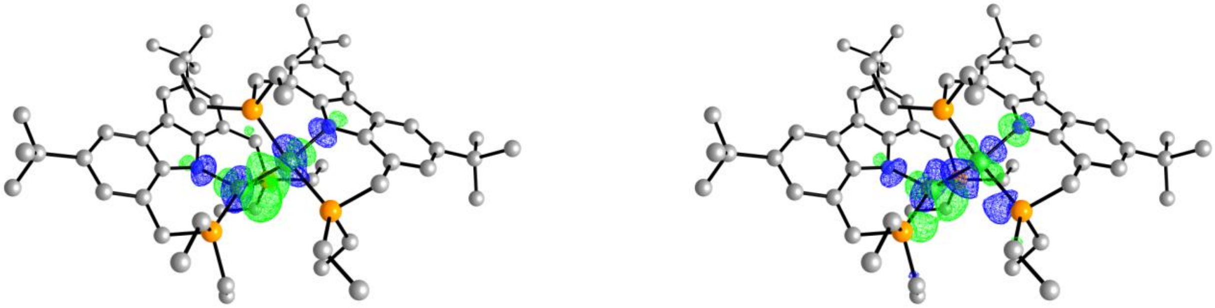

σ σ*

Figure S182. Visualization of the SOMOs (singly occupied QROs) in **4^{Et}-Ni-M3** (isovalue 0.035).

10.10) Energy Decomposition Analysis (EDA)

Energy decomposition analyses were performed using ADF 2024.1[83-84] employing the B3LYP functional with D3 dispersion correction and TZ2P basis set, incorporating scalar relativistic effects via ZORA. The geometries of **4^{iPr}-Pd** (conformer 1, chapter 10.6), **4^{Et}-Pd** (chapter 10.6), **4^{Et}-Ni** (chapter 10.6) and **7^{Et}-NiPd** were utilized and each complex was fragmented into its neutral metalloradical moieties. Additionally, to isolate the purely electronic influence of substituting *iso*-propyl with ethyl groups, a non-fully-relaxed geometry (**4^{Et}-Pd-nonrelax**) was generated based on the structure of **4^{iPr}-Pd** and used in an EDA (again neutral metalloradical fragments). The *iso*-propyl substituents were replaced by ethyl-groups, followed by constrained optimization where the Pd, P, and N atom coordinates remained frozen. This restricted geometric relaxation solely to the ligand framework (primarily the ethyl substituents), maintaining identical overall ligand positioning, orientation and inter-fragment distances. Consequently, any observed differences in the total orbital interaction energies arise exclusively from altered electronic properties.

The total intrinsic interaction energy between the [(PNP)M^{I}] fragments is calculated by

$$\Delta E_{int} = \Delta E_{Pauli} + \Delta E_{elstat} + \Delta E_{orb} + \Delta E_{dispersion}$$

where ΔE_{Pauli} is the Pauli repulsion, ΔE_{elstat} is the electrostatic interaction, ΔE_{orb} is the total orbital interaction, $\Delta E_{dispersion}$ is the dispersion energy. The total steric interaction is defined as:

$$\Delta E_{steric} = \Delta E_{Pauli} + \Delta E_{elstat}$$

Table S54. Summary of the EDA results for the components of the intrinsic interaction energy for **4^{iPr}-Pd**, **4^{Et}-Pd-nonrelax** and **4^{Et}-Pd**. $\Delta\Delta E$ values were calculated with respect to ΔE(**4^{iPr}-Pd**).

Type	ΔE(**4^{iPr}-Pd**) (kcal mol^{-1})	ΔE(**4^{Et}-Pd-nonrelax**) (kcal mol^{-1})	ΔE(**4^{Et}-Pd**) (kcal mol^{-1})	$\Delta\Delta E$(**4^{iPr}-Pd**) (kcal mol^{-1})	$\Delta\Delta E$(**4^{Et}-Pd-nonrelax**) (kcal mol^{-1})	$\Delta\Delta E$(**4^{Et}-Pd**) (kcal mol^{-1})
ΔE_{Pauli}	136.4	120.2	146.8	0.0	-16.2	10.4
ΔE_{elstat}	-103.1	-97.3	-116.6	0.0	5.8	-13.5
ΔE_{steric}	33.3	22.8	30.2	0.0	-10.5	-3.1
ΔE_{orb}	-55.1	-52.8	-58.8	0.0	2.3	-3.7
$\Delta E_{dispersion}$	-45.9	-33.2	-36.6	0.0	12.7	9.3
ΔE_{int}	-67.7	-63.2	-65.2	0.0	4.5	2.5

Table S55. Summary of the EDA results for the components of the intrinsic interaction energy for **4^{Et}-Ni**, **4^{Et}-Pd** and **7^{Et}-NiPd**. $\Delta\Delta E$ values were calculated with respect to ΔE(**7^{Et}-NiPd**). The overall stabilization or destabilization contributions for the reaction of **4^{Et}-Ni** with **4^{Et}-Pd** to **7^{Et}-NiPd** (ΔE(reaction)) was calculated as ΔE(reaction) $= -(\Delta\Delta E$(**4^{Et}-Ni**) $+ \Delta\Delta E$(**4^{Et}-Pd**)).

Type	ΔE(**4^{Et}-Ni**) (kcal mol^{-1})	ΔE(**4^{Et}-Pd**) (kcal mol^{-1})	ΔE(**7^{Et}-NiPd**) (kcal mol^{-1})	$\Delta\Delta E$(**4^{Et}-Pd**) (kcal mol^{-1})	$\Delta\Delta E$(**4^{Et}-Pd**) (kcal mol^{-1})	$\Delta\Delta E$(**7^{Et}-NiPd**) (kcal mol^{-1})	ΔE(reaction) (kcal mol^{-1})
ΔE_{Pauli}	161.9	146.8	147.9	14.0	-1.1	0.0	-12.9
ΔE_{elstat}	-104.3	-116.6	-104.2	-0.1	-12.4	0.0	12.4
ΔE_{steric}	57.6	30.2	43.7	13.9	-13.5	0.0	-0.4
ΔE_{orb}	-55.5	-58.8	-55.6	0.1	-3.3	0.0	3.2
$\Delta E_{dispersion}$	-43.1	-36.6	-42.8	-0.2	6.2	0.0	-6.0
ΔE_{int}	-40.9	-65.2	-54.7	13.8	-10.5	0.0	-3.2

The open-shell fragments of **4^{iPr}-Pd** and **4^{Et}-Pd** were optimized at the CPCM(toluene)/r^{2}SCAN-3c/Def2-mTZVP level of theory. To obtain the relative electronic stabilization energy of each dimer with respect to its relaxed, dissociated (twisted) metalloradical fragments, the respective deformation energies were added to the EDA-derived total bonding energies. The deformation energies ($\Delta E^{iPr}_{deformation}$ for **4^{iPr}-Pd**, $\Delta E^{Et}_{deformation}$ for **4^{Et}-Pd**) were calculated as the sum of electronic energy differences between non-relaxed and fully relaxed geometries for both fragments of each dimer:

$$\Delta E^{iPr}_{deformation} = |\Delta SCF(fragment1)| + |\Delta SCF(fragment2)| = (10.7 + 7.5)\ \text{kcal mol}^{-1} = 18.2\ \text{kcal mol}^{-1}$$

$$\Delta E^{Et}_{deformation} = |\Delta SCF(fragment1)| + |\Delta SCF(fragment2)| = (2.8 + 2.1)\ \text{kcal mol}^{-1} = 4.9\ \text{kcal mol}^{-1}$$

The resulting relative electronic stabilization energies therefore are:

$$\Delta E^{iPr}_{rel} = \Delta E_{int}(\mathbf{4^{iPr}\text{-}Pd}) + \Delta E^{iPr}_{deformation} = -49.5\ \text{kcal mol}^{-1}$$

$$\Delta E^{Et}_{rel} = \Delta E_{int}(\mathbf{4^{Et}\text{-}Pd}) + \Delta E^{Et}_{deformation} = -60.3\ \text{kcal mol}^{-1}$$

This result aligns with experimental observations showing greater stability for **4^{Et}-Pd** relative to **4^{iPr}-Pd**. Additionally, ΔE^{iPr}_{rel} agrees well with the relative electronic energy computed for the dissociation of **4'-Pd** into two **3'-Pd** radicals (ΔSCF(**4'-Pd** → 2x **3'-Pd**) $= 46.4\ \text{kcal mol}^{-1}$; structures from chapters 10.7 and 10.8).

To assess the polarity of the Ni–Pd bond in **7^{Et}-NiPd**, partial charges for both metals and the [(PNP)M] fragments were computed using various charge analysis methods (see Table S56). These included Mulliken, Hirshfeld,[85] Atoms-in-Molecules (AIM), Natural Population Analysis (NPA),[86-87] and charges from electrostatic potentials (CHELGP)[88]. The charges were determined for an electron density computed relativistically (ZORA, SOMF(1x)) utilizing the B3LYP-GD3(BJ) functional, the SARC-ZORA-TZVP basis set for Pd, the ZORA-Def2-TZVP(-f) basis set for all other atoms and the SARC/J auxiliary basis set.

Table S56. Metal and [(PNP)M] fragment charges computed on the relativistic B3LYP-GD3(BJ)/SARC-ZORA-TZVP(Pd), ZORA-Def2-TZVP(-f) level of theory for **7^{Et}-NiPd**.

	Mulliken	Hirshfeld	AIM	NPA	CHELGP
Ni	-0.34	-0.23	0.32	0.37	-0.48
Pd	0.28	0.33	0.03	0.14	0.80
[(PNP)Ni]	0.01	-0.18	0.08	0.05	0.06
[(PNP)Pd]	-0.01	0.18	-0.08	-0.05	-0.06

11) References

[1] G. T. Plundrich, H. Wadepohl, L. H. Gade, "Synthesis and Reactivity of Group 4 Metal Benzyl Complexes Supported by Carbazolide-Based PNP Pincer Ligands" *Inorg. Chem.* **2016**, *55*, 353-365.

[2] G. W. Parshall, "Tetramethylbiphosphine Disulfide" *Org. Synth.* **1965**, *45*, 102.

[3] A. Trenkle, H. Vahrenkamp, "Eine unerwartet einfache Synthese von Dimethylphosphin / An Unexpectedly Simple Synthesis of Dimethyl Phosphine" *Z. Naturforsch. B* **1979**, *34*, 642-643.

[4] G. R. Fulmer, A. J. M. Miller, N. H. Sherden, H. E. Gottlieb, A. Nudelman, B. M. Stoltz, J. E. Bercaw, K. I. Goldberg, "NMR Chemical Shifts of Trace Impurities: Common Laboratory Solvents, Organics, and Gases in Deuterated Solvents Relevant to the Organometallic Chemist" *Organometallics* **2010**, *29*, 2176-2179.

[5] G. M. Tsivgoulis, G. G. Kordopati, D. G. Vachliotis, P. V. Ioannou, "The Reaction of Diethylthiophosphinyl Iodide, $Et_2P(S)I$, with Nucleophiles" *Z. Anorg. Allg. Chem.* **2017**, *643*, 1075-1081.

[6] K. Kabsch, in *International Tables for Crystallography* (Eds. M. G. Rossmann, E. Arnold), *Vol. F,* Ch. 11.3, Kluwer Academic Publishers, Dordrecht, The Netherlands, **2001**.

[7] SAINT, Bruker AXS GmbH, Karlsruhe, Germany **1997-2013**, and SAINT V8.40A, Bruker AXS Inc., Madison, Wisconsin, USA,**2018**.

[8] *CrysAlisPro,* Agilent Technologies UK Ltd., Oxford, UK **2011-2014**, and Rigaku Oxford Diffraction, Rigaku Polska Sp.z o.o., Wrocław, Poland **2015-2020**.

[9] R. H. Blessing, "An empirical correction for absorption anisotropy" *Acta Crystallogr., Sect. A:Found. Adv.* **1995**, *51*, 33-38.

[10] G. M. Sheldrick, *SADABS,* Bruker AXS GmbH, Karlsruhe, Germany **2004-2014**.

[11] *SCALE3 ABSPACK, CrysAlisPro,* Agilent Technologies UK Ltd., Oxford, UK **2011-2014**, and Rigaku Oxford Diffraction, Rigaku Polska Sp.z o.o., Wrocław, Poland **2015-2020**.

[12] W. R. Busing, H. A. Levy, "High-speed computation of the absorption correction for single-crystal diffraction measurements" *Acta Crystallogr.* **1957**, *10*, 180-182.

[13] O. V. Dolomanov, L. J. Bourhis, R. J. Gildea, J. A. K. Howard, H. Puschmann, "*OLEX2*: a complete structure solution, refinement and analysis program" *J. Appl. Crystallogr.* **2009**, *42*, 339-341.

[14] G. M. Sheldrick, SHELXT, University of Göttingen and Bruker AXS GmbH, Karlsruhe, Germany, **2012-2018**.

[15] M. Ruf, B. C. Noll, Application Note SC-XRD 503, Bruker AXS GmbH, Karlsruhe, Germany **2014**.

[16] G. M. Sheldrick, "Crystal structure refinement with *SHELXL*" *Acta Crystallogr., Sect. C:Struct. Chem.* **2015**, *71*, 3-8.

[17] G. M. Sheldrick, *SHELXL-20xx,* University of Göttingen and Bruker AXS GmbH, Karlsruhe, Germany **2012-2018**.

[18] G. M. Sheldrick, "A short history of SHELX" *Acta Crystallogr., Sect. A:Found. Adv.* **2008**, *64*, 112-122.

[19] J. S. Rollett, in *Crystallographic Computing* (Eds.: F. R. Ahmed, S. R. Hall, H. C. P.), Munksgaard, Copenhagen, Denmark, **1970**, p. 167.

[20] D. Watkin, in *Crystallographic Computing 4* (Eds.: N. W. Isaaks, M. R. Taylor), IUCr and Oxford University Press, Oxford, UK, **1988**.

[21] P. Müller, R. Herbst-Irmer, A. L. Spek, T. R. Schneider, M. R. Sawaya, in *Crystal Structure Refinement* (Ed.: P. Müller), Ch. 5, Oxford University Press, Oxford, UK, **2006**.

[22] D. Watkin, "Structure refinement: some background theory and practical strategies" *J. Appl. Crystallogr.* **2008**, *41*, 491-522.

[23] A. Thorn, B. Dittrich, G. M. Sheldrick, "Enhanced rigid-bond restraints" *Acta Crystallogr., Sect. A:Found. Adv.* **2012**, *68*, 448-451.

[24] B. Rees, L. Jenner, M. Yusupov, "Bulk-solvent correction in large macromolecular structures" *Acta Crystallogr., Sect. D:Struct. Biol.* **2005**, *61*, 1299-1301.

[25] G. H. Imler, M. J. Zdilla, B. B. Wayland, "Evaluation of the $Rh^{(II)}$–$Rh^{(II)}$ Bond Dissociation Enthalpy for $[(TMTAA)Rh]_2$ by ^1H NMR T_2 Measurements: Application in Determining the Rh–C(O)– BDE in $[(TMTAA)Rh]_2C{=}O$" *Inorg. Chem.* **2013**, *52*, 11509-11513.

[26] F. Neese, "The ORCA program system" *WIREs Comput. Mol. Sci.* **2012**, *2*, 73-78.

[27] F. Neese, "Software update: the ORCA program system, version 4.0" *WIREs Comput. Mol. Sci.* **2018**, *8*, e1327.

[28] F. Neese, F. Wennmohs, U. Becker, C. Riplinger, "The ORCA quantum chemistry program package" *J. Chem. Phys.* **2020**, *152*, 224108.

[29] F. Neese, "Software update: The ORCA program system—Version 5.0" *WIREs Comput. Mol. Sci.* **2022**, *12*, e1606.

[30] S. Grimme, A. Hansen, S. Ehlert, J.-M. Mewes, "r^2SCAN-3c: A “Swiss army knife” composite electronic-structure method" *J. Chem. Phys.* **2021**, *154*, 064103.

[31] J. W. Furness, A. D. Kaplan, J. Ning, J. P. Perdew, J. Sun, "Accurate and Numerically Efficient r^2SCAN Meta-Generalized Gradient Approximation" *J. Phys. Chem. Lett.* **2020**, *11*, 8208-8215.

[32] S. Ehlert, U. Huniar, J. Ning, J. W. Furness, J. Sun, A. D. Kaplan, J. P. Perdew, J. G. Brandenburg, "r^2SCAN-D4: Dispersion corrected meta-generalized gradient approximation for general chemical applications" *J. Chem. Phys.* **2021**, *154*, 061101.

[33] F. Weigend, R. Ahlrichs, "Balanced basis sets of split valence, triple zeta valence and quadruple zeta valence quality for H to Rn: Design and assessment of accuracy" *Phys. Chem. Chem. Phys.* **2005**, *7*, 3297-3305.

[34] F. Weigend, "Accurate Coulomb-fitting basis sets for H to Rn" *Phys. Chem. Chem. Phys.* **2006**, *8*, 1057-1065.

[35] F. Neese, "An Improvement of the Resolution of the Identity Approximation for the Formation of the Coulomb Matrix" *J. Comput. Chem.* **2003**, *24*, 1740-1747.

[36] A. Schäfer, H. Horn, R. Ahlrichs, "Fully optimized contracted Gaussian basis sets for atoms Li to Kr" *J. Chem. Phys.* **1992**, *97*, 2571-2577.

[37] S. Grimme, J. G. Brandenburg, C. Bannwarth, A. Hansen, "Consistent structures and interactions by density functional theory with small atomic orbital basis sets" *J. Chem. Phys.* **2015**, *143*, 054107.

[38] B. de Souza, "GOAT: A Global Optimization Algorithm for Molecules and Atomic Clusters" *Angew. Chem. Int. Ed.* **2025**, *64*, e202500393.

[39] C. Bannwarth, S. Ehlert, S. Grimme, "GFN2-xTB—An Accurate and Broadly Parametrized Self-Consistent Tight-Binding Quantum Chemical Method with Multipole Electrostatics and Density-Dependent Dispersion Contributions" *J. Chem. Theory Comput.* **2019**, *15*, 1652-1671.

[40] C. Bannwarth, E. Caldeweyher, S. Ehlert, A. Hansen, P. Pracht, J. Seibert, S. Spicher, S. Grimme, "Extended tight-binding quantum chemistry methods" *WIREs Comput. Mol. Sci.* **2021**, *11*, e1493.
[41] V. Barone, M. Cossi, "Quantum Calculation of Molecular Energies and Energy Gradients in Solution by a Conductor Solvent Model" *J. Phys. Chem. A* **1998**, *102*, 1995-2001.
[42] M. Cossi, N. Rega, G. Scalmani, V. Barone, "Energies, Structures, and Electronic Properties of Molecules in Solution with the C-PCM Solvation Model" *J. Comput. Chem.* **2003**, *24*, 669-681.
[43] J. Tao, J. P. Perdew, V. N. Staroverov, G. E. Scuseria, "Climbing the Density Functional Ladder: Nonempirical Meta–Generalized Gradient Approximation Designed for Molecules and Solids" *Phys. Rev. Lett.* **2003**, *91*, 146401.
[44] V. N. Staroverov, G. E. Scuseria, J. Tao, J. P. Perdew, "Comparative assessment of a new nonempirical density functional: Molecules and hydrogen-bonded complexes" *J. Chem. Phys.* **2003**, *119*, 12129-12137.
[45] S. Grimme, "Accurate Description of van der Waals Complexes by Density Functional Theory Including Empirical Corrections" *J. Comput. Chem.* **2004**, *25*, 1463-1473.
[46] S. Grimme, J. Antony, S. Ehrlich, H. Krieg, "A consistent and accurate *ab initio* parametrization of density functional dispersion correction (DFT-D) for the 94 elements H-Pu " *J. Chem. Phys.* **2010**, *132*, 154104.
[47] A. D. Becke, E. R. Johnson, "Exchange-hole dipole moment and the dispersion interaction" *J. Chem. Phys.* **2005**, *122*, 154104.
[48] E. R. Johnson, A. D. Becke, "A post-Hartree–Fock model of intermolecular interactions" *J. Chem. Phys.* **2005**, *123*, 024101.
[49] W. J. Hehre, R. Ditchfield, J. A. Pople, "Self—Consistent Molecular Orbital Methods. XII. Further Extensions of Gaussian—Type Basis Sets for Use in Molecular Orbital Studies of Organic Molecules" *J. Chem. Phys.* **1972**, *56*, 2257–2261.
[50] P. C. Hariharan, J. A. Pople, "The Influence of Polarization Functions on Molecular Orbital Hydrogenation Energies" *Theor. Chim. Acta* **1973**, *28*, 213-222.
[51] T. Bruckhoff, J. Ballmann, L. H. Gade, "Radicalizing CO by Mononuclear Palladium(I)" *Angew. Chem. Int. Ed.* **2024**, *63*, e202320064.
[52] A. D. Becke, "Density-functional thermochemistry. III. The role of exact exchange" *J. Chem. Phys.* **1993**, *98*, 5648–5652.
[53] C. Lee, W. Yang, R. G. Parr, "Development of the Colle-Salvetti correlation-energy formula into a functional of the electron density" *Phys. Rev. B* **1988**, *37*, 785-789.
[54] J. D. Rolfes, F. Neese, D. A. Pantazis, "All-electron scalar relativistic basis sets for the elements Rb–Xe" *J. Comput. Chem.* **2020**, *41*, 1842-1849.
[55] E. van Lenthe, E. J. Baerends, J. G. Snijders, "Relativistic regular two-component Hamiltonians" *J. Chem. Phys.* **1993**, *99*, 4597–4610.
[56] E. van Lenthe, E. J. Baerends, J. G. Snijders, "Relativistic total energy using regular approximations" *J. Chem. Phys.* **1994**, *101*, 9783–9792.
[57] C. van Wüllen, "Molecular density functional calculations in the regular relativistic approximation: Method, application to coinage metal diatomics, hydrides, fluorides and chlorides, and comparison with first-order relativistic calculations" *J. Chem. Phys.* **1998**, *109*, 392–399.
[58] F. Neese, "Efficient and accurate approximations to the molecular spin-orbit coupling operator and their use in molecular *g*-tensor calculations" *J. Chem. Phys.* **2005**, *122*, 034107.
[59] B. A. Heß, C. M. Marian, U. Walgren, O. Gropen, "A mean-field spin-orbit method applicable to correlated wavefunctions" *Chem. Phys. Lett.* **1996**, *251*, 365-371.
[60] J. P. Perdew, Y. Wang, "Accurate and simple analytic representation of the electron-gas correlation energy" *Phys. Rev. B* **1992**, *45*, 13244-13249.
[61] B. O. Roos, P. R. Taylor, P. E. M. Siegbahn, "A complete active space SCF method (CASSCF) using a density matrix formulated super-CI approach" *Chem. Phys.* **1980**, *48*, 157-173.
[62] P. Siegbahn, A. Heiberg, B. Roos, B. Levy, "A Comparison of the Super-CI and the Newton-Raphson Scheme in the Complete Active Space SCF Method" *Phys. Scr.* **1980**, *21*, 323-327.
[63] C. Angeli, R. Cimiraglia, S. Evangelisti, T. Leininger, J.-P. Malrieu, "Introduction of *n*-electron valence states for multireference perturbation theory" *J. Chem. Phys.* **2001**, *114*, 10252–10264.
[64] C. Angeli, R. Cimiraglia, J.-P. Malrieu, "*N*-electron valence state perturbation theory: a fast implementation of the strongly contracted variant" *Chem. Phys. Lett.* **2001**, *350*, 297-305.
[65] C. Angeli, R. Cimiraglia, J.-P. Malrieu, "*n*-electron valence state perturbation theory: A spinless formulation and an efficient implementation of the strongly contracted and of the partially contracted variants" *J. Chem. Phys.* **2002**, *117*, 9138–9153.
[66] A. Hellweg, C. Hättig, S. Höfener, W. Klopper, "Optimized accurate auxiliary basis sets for RI-MP2 and RI-CC2 calculations for the atoms Rb to Rn" *Theor. Chem. Acc.* **2007**, *117*, 587-597.
[67] G. L. Stoychev, A. A. Auer, F. Neese, "Automatic Generation of Auxiliary Basis Sets" *J. Chem. Theory Comput.* **2017**, *13*, 554-562.
[68] L. Lang, M. Atanasov, F. Neese, "Improvement of Ab Initio Ligand Field Theory by Means of Multistate Perturbation Theory" *J. Phys. Chem. A* **2020**, *124*, 1025-1037.
[69] T. Lu, F. Chen, "Multiwfn: A Multifunctional Wavefunction Analyzer" *J. Comput. Chem.* **2012**, *33*, 580-592.
[70] Z. Liu, T. Lu, Q. Chen, "An sp-hybridized all-carboatomic ring, cyclo[18]carbon: Electronic structure, electronic spectrum, and optical nonlinearity" *Carbon* **2020**, *165*, 461-467.
[71] T. Lu, "A comprehensive electron wavefunction analysis toolbox for chemists, Multiwfn" *J. Chem. Phys.* **2024**, *161*, 082503.
[72] R. F. W. Bader, "Atoms in molecules" *Acc. Chem. Res.* **1985**, *18*, 9-15.
[73] R. F. W. Bader, "A Quantum Theory of Molecular Structure and Its Applications" *Chem. Rev.* **1991**, *91*, 893-928.
[74] R. Ponec, "Electron Pairing and Chemical Bonds: Chemical Bonds from the Condition of Minimum Fluctuation of Electron Pair." *Int. J. Quantum Chem.* **1998**, *69*, 193-200.
[75] Chemcraft - graphical software for visualization of quantum chemistry computations (www.chemcraftprog.com).
[76] G. T. P. Charnock, I. Kuprov, "A partial differential equation for pseudocontact shift" *Phys. Chem. Chem. Phys.* **2014**, *16*, 20184-20189.
[77] J. C. Ott, E. A. Suturina, I. Kuprov, J. Nehrkorn, A. Schnegg, M. Enders, L. H. Gade, "Observability of Paramagnetic NMR Signals at over 10 000 ppm Chemical Shifts" *Angew. Chem. Int. Ed.* **2021**, *60*, 22856-22864.

[78] F. Braun, T. Bruckhoff, J. C. Ott, J. Ballmann, L. H. Gade, "Carbon-Carbon Bond Activation at Chromium(I): An 11-Electron Complex Cleaving Dialkynes" *Angew. Chem. Int. Ed.* **2024**, *63*, e202418646.
[79] D. Hartmann, H. Wadepohl, L. H. Gade, "Synthesis and Structural Characterization of Group 10 Metal Complexes Bearing an Amidodiphosphine Pincer Ligand" *Z. Anorg. Allg. Chem.* **2018**, *644*, 1011-1017.
[80] I. Bertini, C. Luchinat, G. Parigi, E. Ravera, *NMR of Paramagnetic Molecules - Applications to Metallobiomolecules and Models*, 2nd ed., **2016**.
[81] M. Enders, in *Comprehensive Inorganic Chemistry III (Third Edition)* (Eds.: J. Reedijk, K. R. Poeppelmeier), Elsevier, **2023**, pp. 209-229.
[82] G. Parigi, C. Luchinat, in *Paramagnetism in Experimental Biomolecular NMR*, The Royal Society of Chemistry, **2018**.
[83] G. t. Velde, F. M. Bickelhaupt, E. J. Baerends, C. F. Guerra, S. J. A. v. Gisbergen, J. G. Snijders, T. Ziegler, "Chemistry with ADF" *J. Comput. Chem.* **2001**, *22*, 931-967.
[84] ADF **2024.1**, SCM, Theoretical Chemistry, Vrije Universiteit, Amsterdam, The Netherlands, http://www.scm.com.
[85] F. L. Hirshfeld, "Bonded-Atom Fragments for Describing Molecular Charge Densities" *Theor. Chim. Acta* **1977**, *44*, 129-138.
[86] E. D. Glendening, J. K. Badenhoop, A. E. Reed, J. E. Carpenter, J. A. Bohmann, C. M. Morales, P. Karafiloglou, C. R. Landis, F. Weinhold, *NBO 7.0*, University of Wisconsin, Madison (WI).
[87] E. D. Glendening, C. R. Landis, F. Weinhold, "*NBO 7.0*: New Vistas in Localized and Delocalized Chemical Bonding Theory" *J. Comput. Chem.* **2019**, *40*, 2234-2241.
[88] C. M. Breneman, K. B. Wiberg, "Determining atom-centered monopoles from molecular electrostatic potentials. The need for high sampling density in formamide conformational analysis" *J. Comput. Chem.* **1990**, *11*, 361-373.
